# Discovery of 3-Amino-1*H*-pyrazole-Based Kinase Inhibitors to Illuminate the Understudied PCTAIRE Family

**DOI:** 10.3390/ijms232314834

**Published:** 2022-11-27

**Authors:** Jennifer Alisa Amrhein, Lena Marie Berger, Amelie Tjaden, Andreas Krämer, Lewis Elson, Tuomas Tolvanen, Daniel Martinez-Molina, Astrid Kaiser, Manfred Schubert-Zsilavecz, Susanne Müller, Stefan Knapp, Thomas Hanke

**Affiliations:** 1 Institute of Pharmaceutical Chemistry, Goethe-University Frankfurt, Max-von-Laue-Str. 9, 60438 Frankfurt am Main, Germany; 2 Structural Genomics Consortium, Buchmann Institute for Life Sciences, Goethe-University Frankfurt, Max-von-Laue-Str. 15, 60438 Frankfurt am Main, Germany; 3 German Cancer Consortium (DKTK), German Cancer Research Center (DKFZ), DKTK Site Frankfurt-Mainz, 69120 Heidelberg, Germany; 4 Division of Rheumatology, Department of Medicine Solna, Karolinska University Hospital and Karolinska Institute, Solnavägen 1, 17177 Solna, Sweden; 5 Pelago Bioscience AB, Scheeles Väg 1, 17165 Solna, Sweden

**Keywords:** CDK16, cell cycle, FUCCI, kinase, kinase inhibitor, NanoBRET, synthesis

## Abstract

The PCTAIRE subfamily belongs to the CDK (cyclin-dependent kinase) family and represents an understudied class of kinases of the dark kinome. They exhibit a highly conserved binding pocket and are activated by cyclin Y binding. CDK16 is targeted to the plasma membrane after binding to *N*-myristoylated cyclin Y and is highly expressed in post-mitotic tissues, such as the brain and testis. Dysregulation is associated with several diseases, including breast, prostate, and cervical cancer. Here, we used the *N*-(1*H*-pyrazol-3-yl)pyrimidin-4-amine moiety from the promiscuous inhibitor **1** to target CDK16, by varying different residues. Further optimization steps led to **43d**, which exhibited high cellular potency for CDK16 (EC_50_ = 33 nM) and the other members of the PCTAIRE and PFTAIRE family with 20–120 nM and 50–180 nM, respectively. A DSF screen against a representative panel of approximately 100 kinases exhibited a selective inhibition over the other kinases. In a viability assessment, **43d** decreased the cell count in a dose-dependent manner. A FUCCI cell cycle assay revealed a G2/M phase cell cycle arrest at all tested concentrations for **43d**, caused by inhibition of CDK16.

## 1. Introduction

**Figure 1:**
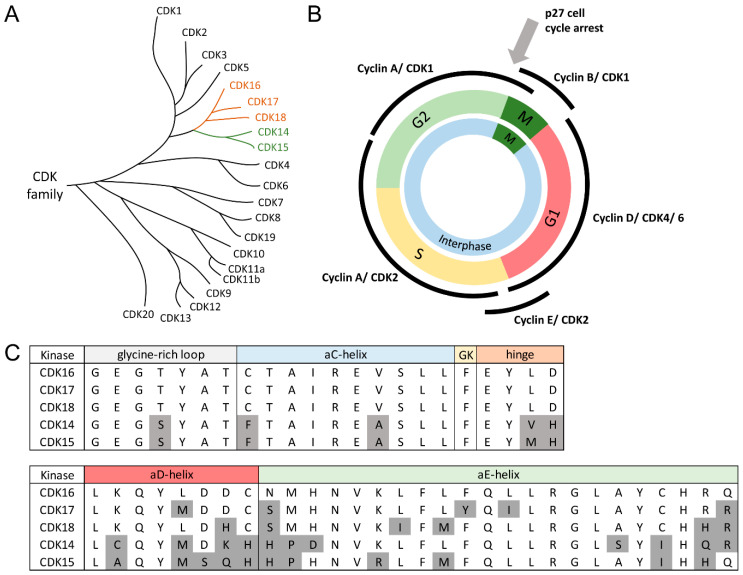
(**A**) Phylogenetic tree of the CDK family. The PFTAIRE subfamily is highlighted in green, the PCTAIRE subfamily in orange (Adapted from Wells et. al.,) [1]. (**B**) Overview of the cell cycle phases and the involvement of CDK’s 1,2,4 and 6 in the transitions through the different phases (Adapted from Suryadinata et.al.,) [10]. Dysregulation of p27 through CDK16 led to a G2/M phase cell cycle arrest. (**C**) Alignment of key residues of the ATP binding pocket of CDK14–18. Differences in comparison to CDK16 are highlighted in grey.

Cyclin-dependent kinases are serine-threonine kinases activated by activation loop phosphorylation and cyclins. The CDK family in eukaryotic cells consists of 21 serine/threonine kinases that are characterized by conserved structural motifs and sequence similarity (Figure 1A) [1]. They are characterized by a largely conserved ATP binding pocket, a PSTAIRE-like binding domain, and an activating T-loop motif. Activation of the kinase through cyclin binding shifts the T-loop to expose the substrate-binding site and primes the phospho-transfer reaction by aligning specific residues within the active site [2,3]. CDKs have key roles in regulating the cell cycle but they also perform diverse other cellular functions, such as regulation of mRNA processing and transcription [4,5,6]. Many CDKs have evolved as major drug targets which has led to recent approvals of CDK4/6 inhibitors for the treatment of cancer [7,8,9].

However, CDK14–18, also called “TAIRE” kinases based on a conserved sequence motif, remain poorly studied and no selective inhibitors have been developed so far, despite interesting links to disease development. CDK14, for instance, is implicated in the regulation of Wnt signaling by forming a CDK14-cyclin Y complex at the plasma membrane that phosphorylates the Wnt co-receptor Lrp6 [11]. CDK14 together with CDK15 forms a small subfamily also referred to as PFTK1 and PFTK2, based on the sequence of the cyclin-binding domain (PFTAIRE). Additionally, CDK15 regulates the Wnt pathway and β-catenin/MEK–ERK signaling by phosphorylation of PAK4, promoting cell proliferation and migration. CDK15 activation has therefore been linked to colorectal cancer progression [12]. CDK16–18 (PCTK1–3) form the PCTAIRE subfamily, which shares high sequence similarity with small differences within their N- and C-terminal domains [13]. CDK17 plays a role in regulating the glycerophospholipid metabolism and it shows high expression levels in Alzheimer’s disease where it promotes neurodegeneration [14]. Dysfunction of CDK18 has been associated with Alzheimer’s disease and other diseases, such as neurological disorders, cerebral ischemia, and cancer [15]. CDK16 is activated by cyclin Y at the plasma membrane and participates in many different pathways including Wnt-dependent signalling or signal transduction in the primary cilium [16]. In contrast to other CDKs, binding of cyclin Y to CDK16 requires a part of an N-terminal extension in addition to the kinase domain. This additional region contains serine 153, which can be phosphorylated. Phosphorylation at this site prevents the formation of the CDK16-cyclin Y complex preventing CDK16 activation [17]. CDK16 is highly expressed in postmitotic tissues such as the testis and the brain. Furthermore, CDK16 is highly expressed in postmeiotic spermatids, whereas it is absent in mature spermatozoa [18]. In the brain, CDK16 can be found in Purkinje and pyramidal cells of the hippocampus and in the neocortex and plays a role in regulation of intracellular vesicles and neurite outgrowth [19,20]. It is also involved in other processes such as glucose homeostasis [21], vesicle trafficking [22], and muscle differentiation [23]. Dysregulation of CDK16 has been described in many cancers such as breast, prostate, cervical cancers, and melanomas [24,25,26]. For example, CDK16 phosphorylates the tumor suppressor p27 at Ser10, which promotes ubiquitination, thereby attenuating apoptosis [27]. p27 regulates the cell cycle by inhibiting CDK4 and CDK6/cyclin complexes, as well as influencing the motility and apoptosis of cells [28]. Downregulation of p27 is associated with high-grade tumors and a poor survival prognosis [29,30]. Yanagi et. al. demonstrated that CDK16 knockout induces G2/M cell cycle arrest, apoptosis, and accumulation of p27 in cutaneous squamous cell carcinoma cells (SCC) (Figure 1B) [28]. In 2018, Wang et. al. found increased expression of CDK16 in NSCLC tumor tissues. Therefore, the oncogenic role of CDK16 is attributed to the p27 ubiquitination triggered by the phosphorylation [31]. Degradation of the tumor suppressor p53 via ubiquitination is also attributed to CDK16. It has been shown that CDK16 phosphorylates p53 at Ser315 and enhances the transcriptional activity which promotes radioresistance in lung cancer [32]. In addition, Dohmen et. al. identified the CDK16/cyclin Y complex as a substrate of AMPK in macroautophagy. Cyclin Y is phosphorylated by AMPK at Ser326, which promotes the activity of CDK16 by forming the CDK16-cyclin Y complex and a subsequent autophosphorylation at Ser336. These phosphorylation events induce autophagy in a ULK1 and Beclin1-dependent manner [33,34]. Despite their biological importance, CDK14–18 represents an understudied subclass of CDKs, whereas CDK1, 2, and 4, in particular, are well characterized [35]. This highlights the need to develop new inhibitors to gain a better understanding of the functions of these understudied kinases.

CDK inhibitors represent an important class of anti-tumor therapeutics. The development of CDK inhibitors extends to the 1990s. The first ones were pan-CDK inhibitors, such as Flavopiridol or Roscovitine, which affected cell proliferation. Due to the low selectivity and high toxicity of the so-called first generation of pan-CDK inhibitors, most of them failed in clinical trials. The second generation of pan-CDK inhibitors led to compounds such as dinaciclib or roniciclib with an improved selectivity profile and consequently fewer side effects. To date, approximately 40 pan-CDK inhibitors are in diverse stages of research or different phases of clinical trials and are used for the treatment of various tumors, such as leukaemia, melanoma, or breast cancer. Nevertheless, the development of specific CDK inhibitors represents an important research area in the medicinal chemistry. Palbociclib, ribociclib, and abemaciclib are FDA-approved CDK4/6 inhibitors for the treatment of HR^+^ advanced breast cancer. They interfere with the cell cycle by blocking the phosphorylation of Rb protein, resulting in cell cycle arrest from G1 to S phase. ICEC0942 is a phase I/II CDK7 inhibitor for the treatment of breast or prostate cancer. AZD-4573 inhibits CDK9, leading to a down-regulation of oncogenic genes such as MCL-1. Preclinical studies revealed anti-tumor effects in hematologic malignancies. The covalent CDK12/13 inhibitor THZ531 exhibits down-regulation effects of DNA damage response genes and is used together with sorafenib for the treatment of hepatocellular carcinoma [36,37]. To date, only a covalent pan-TAIRE inhibitor has been developed [38]. However, some PARP inhibitors have been recently described targeting kinases including CDK15 [39] and the structure of CDK16 with non-selective kinases inhibitors that may serve as starting points for chemical probe development has been disclosed [40]. The development of selective inhibitors for the PCTAIRE subfamily is still in the early stages and to date, no selective inhibitors are published. The high similarity of the kinase domain within the PCTAIRE subfamily with 73% identity makes it challenging to develop selective kinase inhibitors (Figure 1C) [26].

**Figure 2:**
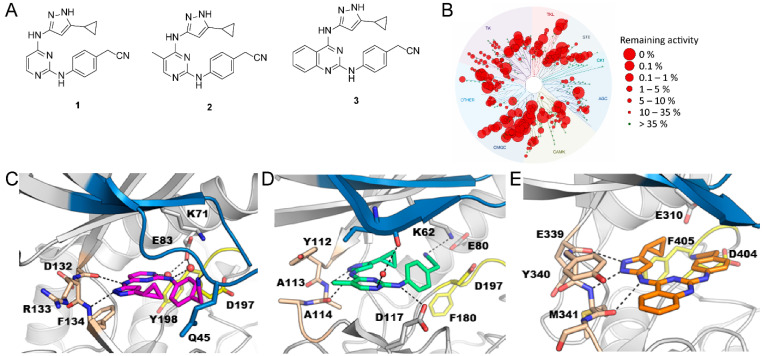
(**A**). Chemical structures of three previous published promiscuous kinase inhibitors **1**, **2** and **3**. (**B**). Selectivity profile of **1** against 359 recombinant human protein kinases at a screening concentration of 1 µM, using the KINOMEscan assay platform [41]. (**C**). Crystal structure of VRK1 in complex with **1** (PDB: 3OP5). (**D**). Crystal structure of STK17B in complex with **2** (PDB: 3LM0). (**E**). Crystal structure of c-Src in complex with **3** (PDB: 3F6X). The hinge region is highlighted in beige, the P-loop in blue, and the DFG motif (in VRK1 DYG motif) in yellow.

In 2008, Statsuk et. al. published the promiscuous kinase inhibitors **1, 2** and **3** (Figure 2A). A kinome-wide screen against 359 wild-type kinases emphasized their promiscuous behaviour by targeting 337 and 317 kinases for **1** and **3**, respectively. The selectivity profile of **1** at a screening concentration of 1 µM is shown in Figure 2B. Additionally, the K_D_ values of **1** against 40 different kinases have been reported. CDK2, CDK5, and JNK3 were the most potently inhibited kinases in this set with 4.6, 27.6, and 26.1 nM, respectively [41]. The crystal structures of all compounds (**1**–**3**) were each determined in complex with different kinases such as VRK1 with **1**, STK17B with **2**, and c-Src with **3** (Figure 2C–E). These different crystal structures indicated mainly two different binding modes. While for **1** the pyrimidine is facing towards the hydrophobic pocket, in **2** and **3** the molecule is reversed and the pyrimidine or quinazoline moiety targets the front pocket. The flexibility of the molecule in the binding pocket could be an explanation for the various targets and the promiscuous behaviour. The high on-target potency within the CDK family, particularly the PCTAIRE family [41], combined with the known crystal structures of the various derivatives makes the 3-aminopyrazole moiety an excellent starting point for the development of a structure activity relationship (SAR) study to obtain a selective kinase inhibitor for exploring the dark kinase CDK16.

The pyrazole hinge binding moiety represents a privileged scaffold in the medicinal chemistry for the development of kinase inhibitors. Previous publications underline the importance, as well as the anti-proliferative and anti-cancer potential of pyrazole-based molecules. They play a crucial role in the treatment of various diseases and cancer types, such as breast cancer, lymphoma, cervical cancer, or inflammation disorders [42,43].

**Figure 3:**
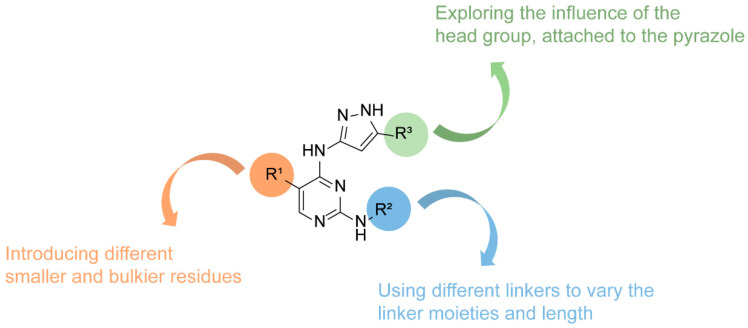
Summary of modifications, using **1**–**3** as the lead structure.

Here, the *N*^4^-(1*H*-pyrazol-3-yl)pyrimidine-2,4-diamine core of the promiscuous inhibitors (**1**–**3**) was chosen as pharmacophore for the structure-based modification on this scaffold (Figure 3). The *N*-(1*H*-pyrazol-3-yl)pyrimidin-4-amine core can be also found in the aurora kinase inhibitor tozasertib. It harbors a methyl moiety at the pyrazole, an additional residue at the C6 position of the pyrimidine, and an aromatic linker that is connected through a sulphur atom. It exhibited a better selectivity profile than the promiscuous inhibitor **1**, showed no affinity for CDK2 and CDK5, however, targeted CDK16 with a K_D_ value of 160 nM [44,45]. Those results underlined the hypothesis to gain selectivity by varying the different substituents at the pyrazole and the pyrimidine. Three different positions were selected to determine their influence on selectivity and potency against CDK16. Therefore, the head group on the pyrazole was varied. Different smaller and bulkier alkyl groups, ester, and amide groups were tested. Furthermore, the effect of an additional residue at the pyrimidine was explored. Based on the lead structure, a methyl-, a chloro-, and a phenyl-residue were used and different linkers were selected for the third position in our SAR analysis. Derived from the nitril of **1**, Boc-protected amines in different positions of the aromatic ring were explored first. In addition, the position of the aromatic ring was varied, alkyl linkers were used and the linker length was changed. The influence of the Boc group was also tested by replacing it with a smaller amide and free amines. 

## 2. Results

### 2.1. Synthesis of 3-Aminopyrazole-Based Kinase Inhibitors

**Scheme 1:**
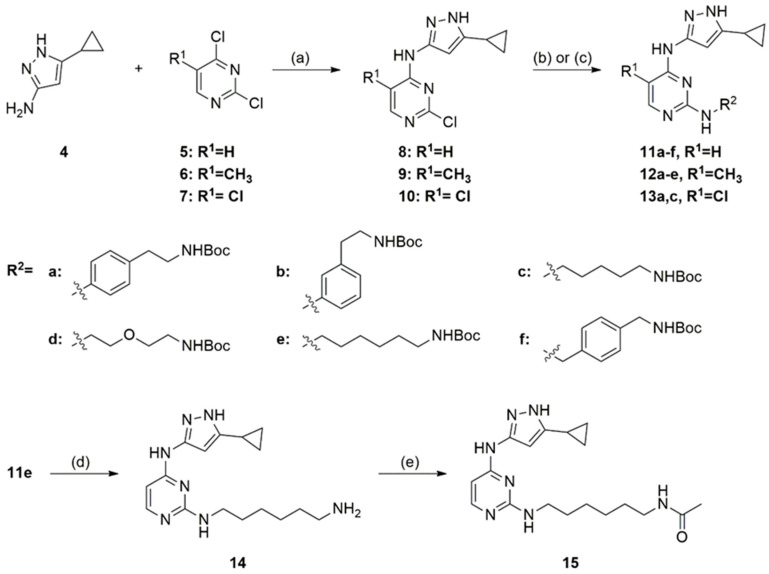
Synthesis of the first series of derivatives **11a**–**f**, **12a**–**e**, **13a**, **c**, and **15.** a. Reagents and conditions: (a) TEA and isopropanol, 48 h, 55–80 °C; (b) TEA and ethanol, microwave, 5–10 h, 80–90 °C; (c) HCl and ethanol, reflux, 4–18h; (d) TFA and DCM, 0 °C–rt, oN; (e) Acetic acid, HATU, DIPEA and DMF, rt, oN.

Based on the scaffold of **1**, the first set of molecules was synthesized. Therefore, 5-cyclopropyl-1*H*-pyrazole-3-amine (**4**) and a pyrimidine derivative (**5**–**7**) were used for a nucleophilic substitution under basic conditions, whereby different substituents such as a hydrogen, a methyl, and a chloro residue at position 5 of the pyrimidine were used. The yields were in the range of 35–89%. Various linkers were attached to the precursor via a second nucleophilic substitution under basic conditions under microwave irradiation or with a catalytic amount of 1M HCl. For this purpose, different aniline derivatives, benzylamine derivatives, and aliphatic linkers were used to obtain the final compounds **11a**–**f**, **12a**–**e**, and **13a, c**. The aniline derivatives could be obtained in moderate to good yields, ranging from 16 to 90%. However, the aliphatic linkers required quite harsh reaction conditions with long reaction times under microwave irradiation and ended in yields of 5–52%. To determine the influence of the Boc group in comparison to a smaller residue, the protecting group of **11e** was cleaved with TFA in DCM. A smaller residue was introduced via an amide coupling, using HATU and acetic acid under basic conditions to obtain **15** with a 30% yield (Scheme 1).

**Scheme 2:**
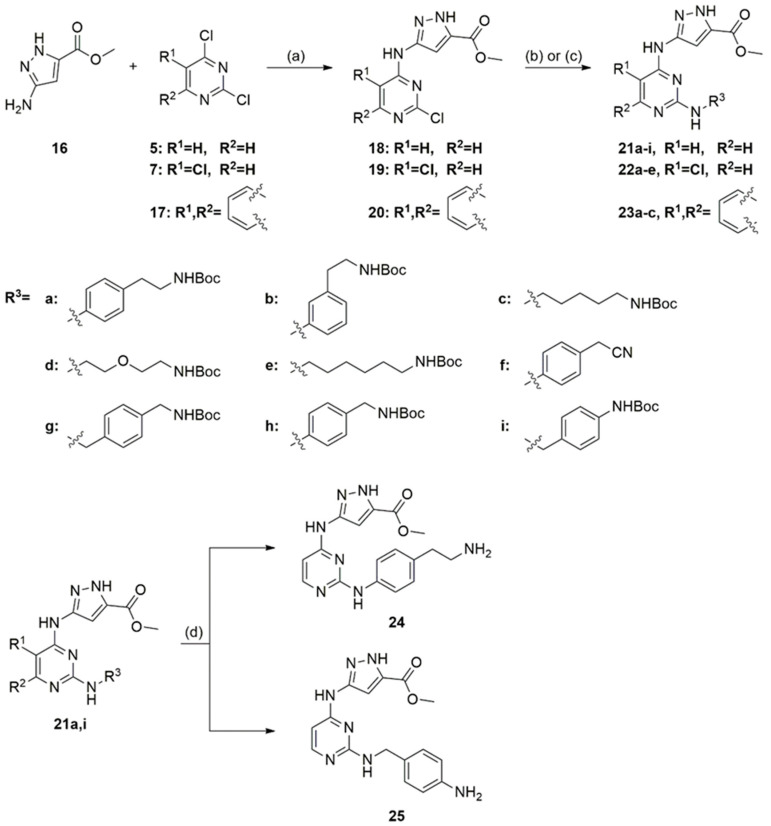
Synthesis of the second series of derivatives **21a**–**i**, **22a**–**e**, **23a**–**c**, and **24**–**25**. a. Reagents and conditions: (a) TEA and isopropanol, 24–72 h, 50–60 °C; (b) TEA and methanol or ethanol, microwave, 3–8 h, 80–120 °C; (c) HCl and ethanol, 70 °C–reflux, 18 h; (d) TFA and DCM, 0 °C–rt, oN.

**Scheme 3:**
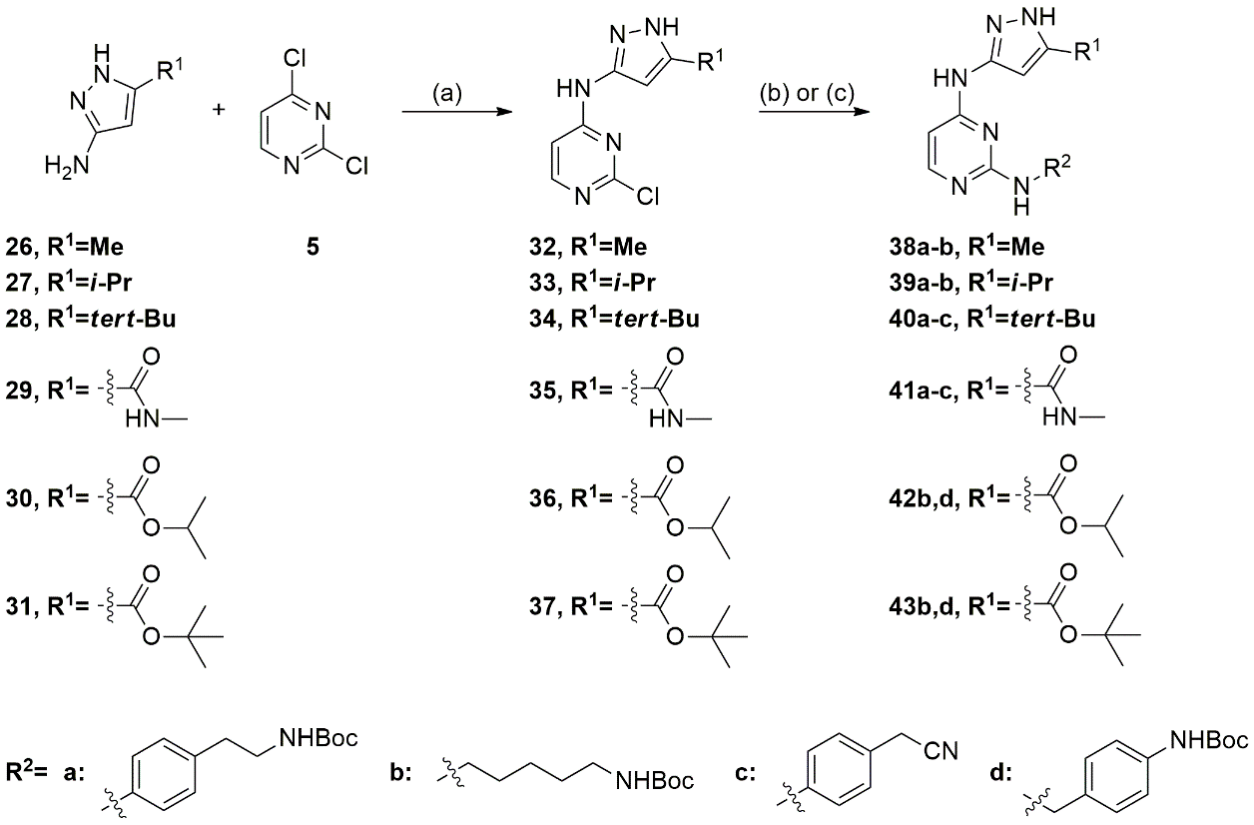
Synthesis of the third series of derivatives **38**–**39a**–**b**, **40**–**41a**–**c**, and **42**–**43b**, **d**. a. Reagents and conditions: (a) TEA and isopropanol, 18–120 h, 60 °C; (b) TEA and ethanol or isopropanol or *tert*-butanol, microwave, 8 h, 90–100 °C; (c) HCl and ethanol, 70 °C–reflux, 18 h.

For the second series, we replaced the cyclopropyl group on the pyrazole with a methyl ester. Again, we used different substituents such as a hydrogen or a chloro residue at position 5 of the pyrimidine, or a quinazoline moiety instead of the pyrimidine and different linkers to investigate their influence. Compounds **18**–**20** were received in yields between 16–84%. The final compounds **21a**–**i**, **22a**–**e**, and **23a**–**c** were synthesized as described above in a two-step synthesis route. The aniline derivatives could be obtained in moderate yields between 49–72% for **21** and **23.** However, a chloro residue at position 5 of the pyrimidine (**22**) decreased the yields dramatically to 5–9%. The yields for the introduction of the aliphatic linkers ranged from 17 to 64%. The Boc group was cleaved for **21a** and **21i** to determine the influence of the primary amines. **24** and **25** were received with 54% and 36%, respectively (Scheme 2). The moiety on the pyrazole was exchanged through various residues like a methyl, isopropyl, *tert*-butyl, methyl amide, isopropyl ester, or *tert*-butyl ester group in the third series. The alkyl groups (**32–34**) and the ester moieties (**36–37**) could be obtained in moderate yields ranging from 35–84%. However, the amide (**35**) was only achieved with 14%. One to three different linkers were attached for each of these functional groups to obtain the molecules **38**–**43**. For the second nucleophilic substitution, compounds **38–41** were gained with yields between 22 and 79%. The compounds harboring an ester moiety (**42–43**) reduced the turnover rate to 4–12% (Scheme 3).

### 2.2. Structure-Activity Relationship of 3-Aminopyrazole-Based Molecules

To investigate the selectivity profile of the different series, a differential scanning fluorimetry (DSF) assay was used [46]. This assay offers a rapid and sensitive screening method that determines the denaturation temperature of a protein in absence and presence of a compound. This assay format measures the fluorescence of a dye, which depends on the folding state of the protein. The difference between the melting temperature (ΔTm) in the absence and presence of the compound represents the binding strength to the protein. We used an in-house panel of 89–105 kinases to screen all synthesized inhibitors (Tables S1–S3). 

**Figure 4:**
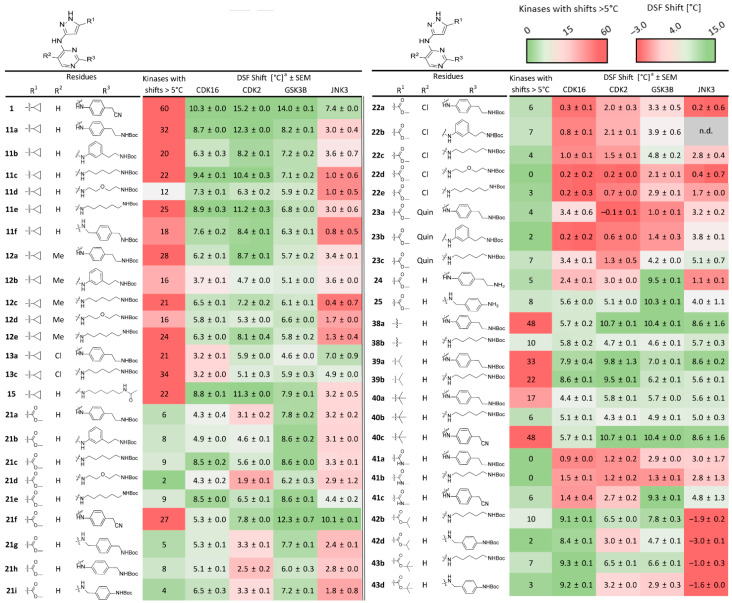
Heatmap of the DSF results of the *N*-(1*H*-pyrazole-3-yl)pyrimidine-4-amine-based inhibitors against CDK16, CDK2, GSK3B, and JNK3 (red means a low stabilization of the desired kinase, green a high stabilization). The number of kinases with shifts >5 °C provided an indication of the selectivity profile of the newly synthesized compounds (red indicates a high number of stabilized kinases and therefore a promiscuous behaviour, green a low number of stabilized kinases). ^a^ DSF measurements were performed in duplicates (±SEM).

The lead structure **1** was resynthesized, based on the synthetic route of Statsuk et. al. [41]. Using the DSF assay format, we identified several molecules, which showed a strong stabilization of CDK16 (Figure 4), including targets that were reported to be strongly inhibited by lead structure **1**. For example, CDK2 and JNK3, as well as GSK3B, which is a potential off-target of the newly synthesized compounds are shown together with the number of kinases with ΔTm shifts >5 °C, providing an indication of the overall selectivity. The compounds of the first series that lacked substitution at the pyrimidine (**11a**–**11f**) exhibited a high stabilization of CDK16. Especially **11c** showed a comparable ΔTm shift with 9.4 °C in comparison to **1** (10.3 °C). The stabilization of CDK2 was also quite high; however, the shifts of JNK3 were negligible. The introduction of a methyl or chloro residue at the pyrimidine was not tolerated. An additional methyl group (**12a**–**e**) reduced the ΔTm shifts by 2 °C on average in comparison to the corresponding molecule without the methyl group. An even stronger reduction of ΔTm shifts was observed by the introduction of a bulkier chloro residue (**13a**, **13c**) showing ΔTm shifts of only 3.2 °C. Additionally, the stabilization of CDK2 was decreased by introduction of the residues at R^1^; however, a chloro residue was tolerated screening JNK3 and the presence of this R^1^ group increased ΔTm shifts to 4.9–7.0 °C. The replacement of the Boc group to a smaller amide (**15**) had no influence on ΔTm shifts. 

The lead structure **1** was highly promiscuous as indicated by stabilizing 60 kinases with ΔTm shifts >5 °C. The compounds with the same precursor but an exchanged linker moiety **11a–f** and **15** were also nonselective with 12–32 significantly stabilized kinases. Additionally, the introduction of an additional residue, such as a methyl or chloro residue at the pyrimidine (**12a**–**12e**, **13a**, and **13c**), did not lead to an improved selectivity with 16–28 shifts >5 °C. The best selectivity within this series was observed for compounds **11d** and **12d** with the *tert-*butyl (2-(aminomethoxy)ethyl)carbamate linker, whereas the replacement from **11e** with the smaller amide in **15** did not impact selectivity. 

The introduction of the methyl ester at the pyrazole (**21a**–**i**) led to an overall lower stabilization of CDK16 in comparison to the lead structure **1**; however, **21c** and **21e** exhibited still similar ΔTm shifts compared to staurosporine (9.1 °C), which has a reported K_D_ value of 24 nM [47]. Additionally, **21i** showed significant ΔTm stabilization (6.5 °C). Surprisingly, the 4-aminobenzonitrile linker (**21f**) exhibited a high stabilization of GSK3B and JNK3, while CDK16 and CDK2 showed with 5.3 and 7.8 °C moderate ΔTm values. Additionally, here, modifications at R^1^ were not tolerated by CDK16. All molecules with the bulkier chloro residue at this position (**22a**–**22e**) or the exchange of the pyrimidine to a quinazoline moiety (**23a**–**23c**) showed no stabilization in the DSF assay. Furthermore, in this second series we were interested in the overall impact of the Boc group and cleaved these compounds to the primary amine in compounds **24** and **25**, which led to a decreased stabilization of CDK16. 

Gratifyingly, the second series showed in general a better selectivity profile and compounds of this series stabilized only between 0–9 kinases with a ΔTm shift > 5 °C. In analogy to the first series, substitution at R^1^ (**22a**–**22e**, **23a**–**23c**) had no influence compared to compounds that had a hydrogen at this position (**21a**–**21i**). Furthermore, the *tert-*butyl (2-(aminomethoxy)ethyl)-carbamate linker (**21d**, **22d**) improved selectivity and led also to a completely inactive compound **22d**. Interestingly, **21f** revealed strong ΔTm shifts of 27 kinases. It seemed that not only the cyclopropyl, but also the 4-aminobenzonitrile linker increase inhibitor promiscuity. The cleavage of the Boc group led to no significant influence on compound selectivity. **24** exhibited the same amount of potential targets, whereby the amount was even increased for **25**. 

The introduction of a methyl, *iso*-propyl, or *tert*-butyl group in the third series led to moderate (**38a**–**38b**, **40a**–**40c**) to good (**39a**–**39b**) stabilization of CDK16. Nevertheless, these modifications exhibited also high ΔTm shifts for CDK2, GSK3B, and JNK3. The replacement to a methyl amide moiety led surprisingly to inactive compounds with a ΔTm stabilization of 0.9–1.5 °C for CDK16. Only the 4-aminobenzonitrile linker (**40c**) resulted in ΔTm shift of GSK3B of 9.3 °C. Next, an *iso*-propyl- (**42b**, **42d**) and *tert*-butyl (**43b**, **43d**) ester were introduced at the pyrazole. These moieties can be found in various already approved drugs or those who are in clinical trials indicating that these groups might have valuable pharmacokinetic properties [48,49]. This led to increased ΔTm shifts between 8.4–9.3 °C for CDK16, while the stabilization for CDK2 and GSK3B was in a moderate range with 3.0–6.5 °C and 2.9–7.8 °C, respectively. JNK3 did not tolerate these variations with DSF shifts below or around 0 °C. 

**Figure 5:**
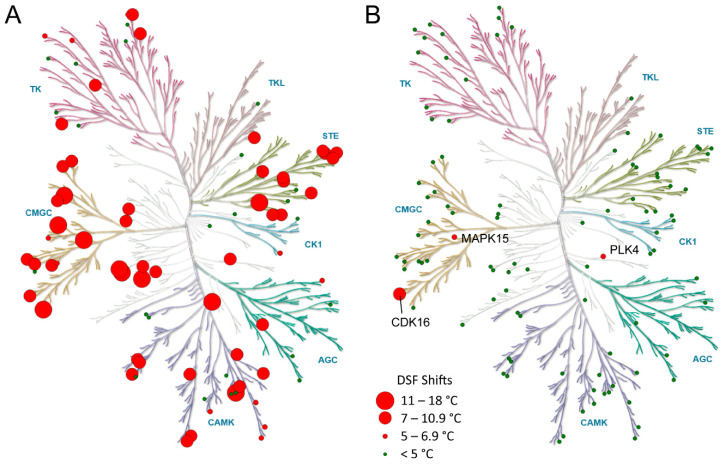
(**A**). Graphic representation of the selectivity data of **1** preserved from the DSF assay, illustrated with Cell Signaling Technology. (**B**). Graphic representation of the selectivity data of **43d** preserved from the DSF assay, illustrated with Cell Signaling Technology.

The introduction of a methyl, *iso*-propyl, or *tert*-butyl group again led to an increased number of ΔTm shifts >5 °C. The number of hits was between 6 and 48 with the trend that the bulkier *tert*-butyl group (**40a**–**40c**) was more selective than the smaller methyl- or *iso*-propyl groups (**38a**–**b**, **39a**–**b**). Remarkably, a methyl amide group was not tolerated in this position. **41a** and **41b** were completely inactive, only **41c** showed residual shifts due to the promiscuous 4-aminobenzonitrile linker. The replacement of the methyl ester to a more stable *iso*-propyl or *tert*-butyl ester decreased the number of potential off-targets. Only 1–2 kinases beside CDK16 exhibited a DSF shift >5 °C for **42d** and **43d** (Figure 5). However, these shifts were comparatively small compared to the staurosporine reference, suggesting that these compounds did not bind strongly to these kinases. **42b** and **43b** exhibited 7–10 ΔTm shifts >5 °C, which is in the same range as the corresponding compound **21c** which has a methyl ester at this position.

In conclusion, the introduction of the cyclopropyl group achieved the highest ΔTm shifts for CDK16 in the DSF assay; however, these compounds had a high number of other potential off-targets. A bulkier substituent at the pyrimidine, as well as a methyl amide group at the pyrazole, were not tolerated by CDK16. The most favorable combination of CDK16 stabilization and potential off-targets was observed for **21c**, **21e**, **21g**, **21h**, and **21i** which belonged to the second series, and **42d** and **43d** carrying an *iso*-propyl or *tert*-butyl ester.

### 2.3. 3-Aminopyrazole-Based Molecules Selectively Stabilized CDK16 in Cells

**Figure 6:**
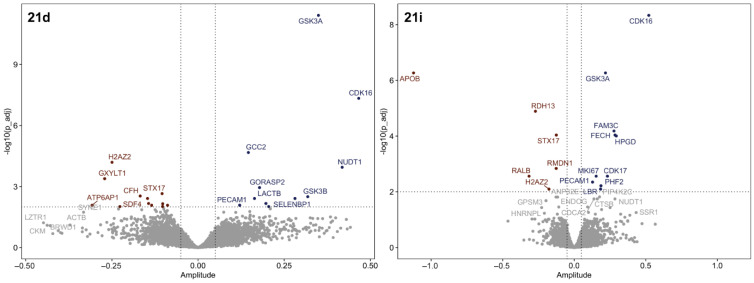
Volcano plots of stabilized (positive amplitude) and destabilized (negative amplitude) proteins of **21d** and **21i**.

To verify whether the substances also lead to stabilization of proteins in the cellular system, proteome-wide CETSA experiments were performed. Therefore, lead structure **1** and some compounds from the second series (**21b**, **21d**, **21e**, **21h**, and **21i**) were selected for this study (Figure 6, Figure S1). While the statistical significance is plotted on the Y-axis, the relative protein amount is shown on the X-axis. As expected, all selected compounds exhibited a stabilization of CDK16 and GSK3 in the CETSA experiment. **21b**, **21e**, and **21h** revealed a broader range of stabilized kinase off-targets, which also correlated with the DSF data. Based on excellent proteome-wide selectivity identifying CDK16 and GSK3 as the most prominently stabilized kinases, **21d** and **21i** were selected for further investigations. In particular, **21i** showed excellent selectivity and potency in CETSA MS (Figure 6). Due to the high stabilization of CDK16 in the cellular system and the great selectivity, we hypothesised that we would see similar properties also for compounds **42d** and **43d** which had even more potent and selective properties from our biochemical screens. We therefore decided to proceed with a CDK16-specific cellular assay.

### 2.4. NanoBRET Assay Revealed Highly Potent CDK Inhibitors in Cells

**Figure 7:**
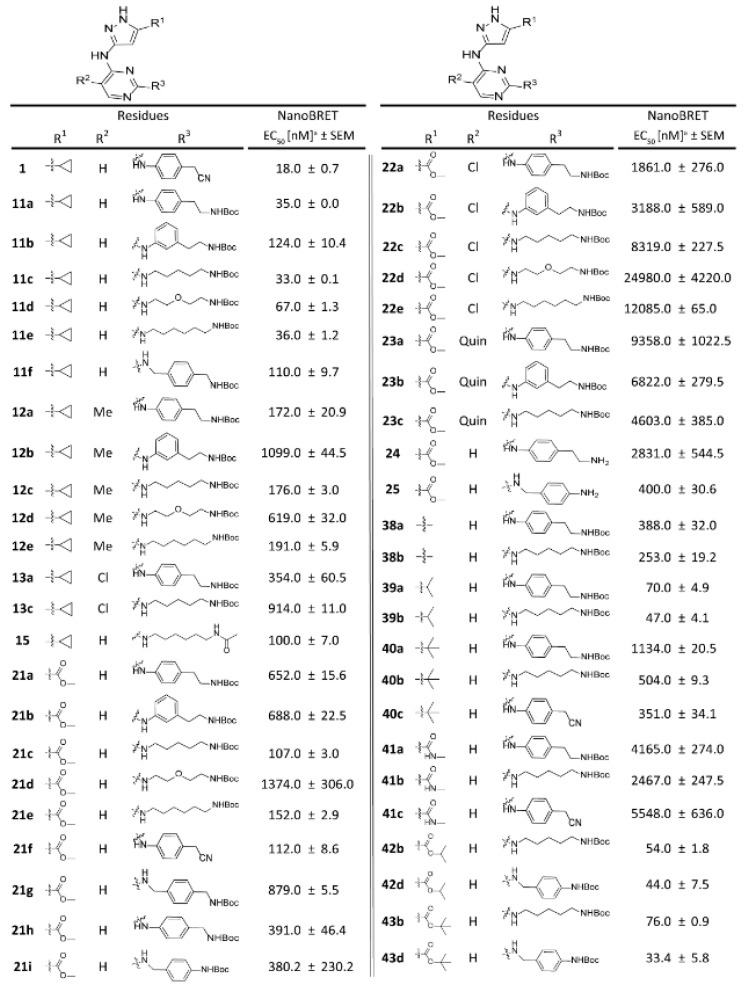
Overview of the binding affinities of the *N*-(1*H*-pyrazole-3-yl)pyrimidine-4-amine-based inhibitors against CDK16. ^a^ EC_50_ values were determined using the NanoBRET assay in a 11-point dose−response curve in duplicates.

Next, to specifically quantify the outcome of the cellular activity of the compounds, a NanoBRET cellular target engagement assay was performed. The determination of the cellular potency on CDK16 correlated well with the corresponding DSF assay data (Figure S2). The lead structure (**1**) exhibited the highest DSF shift with 10.3 °C and a corresponding EC_50_ value of 18.0 nM (Figure 7). Compounds **11a**–**f** bearing the cyclopropyl moiety on the pyrazole ring resulted in excellent cellular activity with EC_50_ values of 33.0–124.0 nM. The introduction of an additional residue on the pyrimidine ring resulted in less potent inhibitors (**12a**–**e**, **13a**, **13c**), associated with a high number of potential off-targets. The second series yielded in some interesting compounds **21c**, **21e**, **21h**, and **21i** with EC_50_ values of 107.0, 152.0, 391.0, and 380.2 nM, respectively. Although slightly less potent than the lead structure and the derivatives from the first series with the cyclopropyl residue, these modifications resulted in more selective compounds with only 4–9 kinases identified with ΔTm shifts >5 °C (Figure 4). The introduction of a chlorine residue at the 5 position of the pyrimidine (**22a**–**22e**) and the exchange of the pyrimidine ring to a quinazoline (**23a**–**23c**) led to inactive compounds with EC_50_ values >1 µM on CDK16. As mentioned before, the exchange from a methyl ester to an *iso*-propyl or *tert*-butyl ester led to a higher stabilization of the kinase in the DSF assay, which correlated with an increase in cellular potency as determined in the NanoBRET assay. **42d** and **43d** showed comparable DSF shifts to the lead structure and EC_50_ values of 44.0 and 33.4 nM, respectively. The number of potential off-targets was low with 1 and 2 kinases with ΔTm >5 °C (Figure 4), highlighting these inhibitors for more comprehensive selectivity studied within the CDK and particular the TAIRE subfamily. The potency of **24** was also assessed for GSK3A and GSK3B in the NanoBRET assay, whereby a shift of 9.5 °C correlated with an EC_50_ value of only 18 µM for GSK3B. **43d** exhibited a DSF shift of 2.9 °C and its activity against GSK3B was consequently negligible (Table S4).

**Figure 8:**
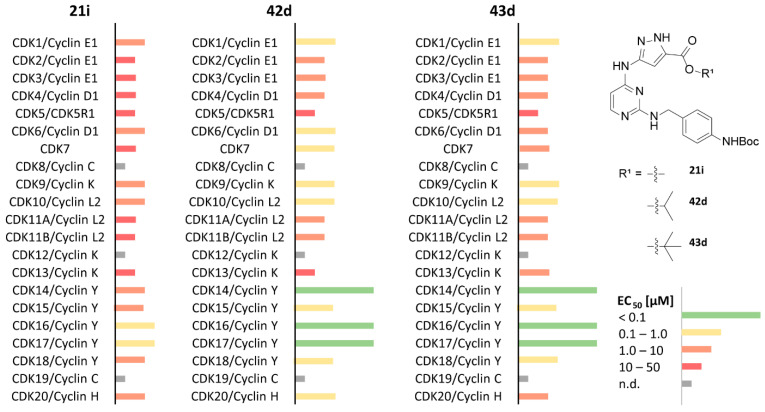
Cellular EC_50_ values of **21i**, **42d**, and **43d** against the CDK family were determined using NanoBRET technology in a 11-dose–response curve in duplicates.

To gain insights into CDK family-wide selectivity, we further profiled the most promising compounds **21i**, **42d**, and **43d**, as well as the lead structure **1** against the CDK family including co-expressed corresponding cyclins, using the NanoBRET technology. As expected, **1** was highly potent on all CDKs, but showed no selectivity within this kinase subfamily. **21i** exhibited an interesting profile, by targeting only CDK16 and CDK17 in the nanomolar range (380.2 and 673.1 nM). However, a much higher potency was observed for **42d** and **43d**, and **43d** exhibited the best selectivity profile within the CDK family. **43d** was highly potent for the PCTAIRE subfamily with 33.4 nM, 21.2 nM, and 120.6 nM for CDK16/cyclin Y, CDK17/cyclin Y, and CDK18/cyclin Y, respectively. The structurally highly related PFTAIRE subfamily was also targeted by **43d** with EC_50_ values ranging from 72.1 to 301.6 nM for CDK14/cyclin Y and CDK15/cyclin Y, respectively. Only CDK1, CDK9, and CDK10 were further off-targets inside the family but with weaker EC_50_ values of 581.9 nM, 993.4 nM, and 651.2 nM, respectively. **42d** showed additionally a nanomolar potency for CDK6, CDK7, and CDK20 (Figure 8, Table S5). 

### 2.5. Metabolic Stability of **21i** and **43d**

**Figure 9:**
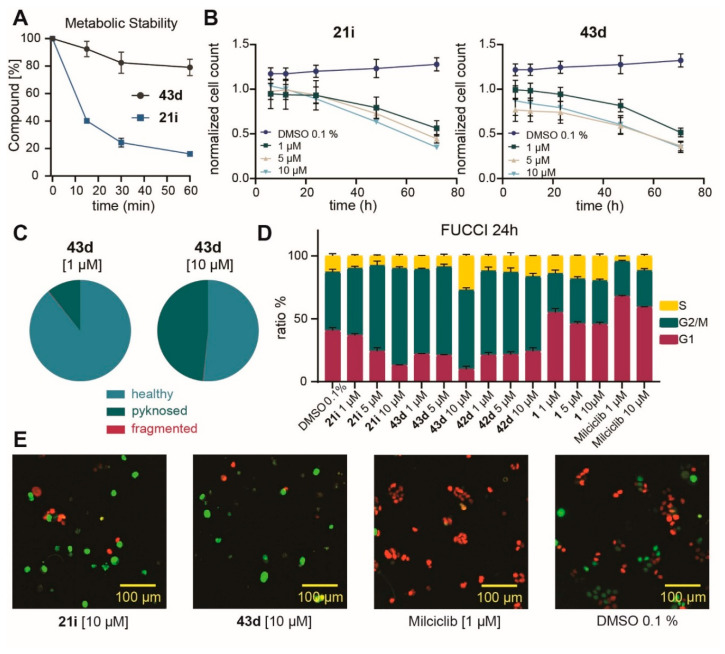
Metabolic stability and cell cycle analysis (FUCCI). (**A**). Metabolic stability of **21i** [10 µM] and **43d** [10 µM] after treatment of 60 min. The residual amount of compound in percent was plotted against time in minutes. (**B**). Normalized cell count of HCT116-FUCCI cells after 0, 6, 12, 24, 48 and 72 h of compound exposure (**21i** [1, 5, 10 µM], **43d** [1, 5, 10 µM]) in comparison to cells exposed to 0.1 % DMSO. Error bars show SEM of two biological replicates. (**C**). Fraction of healthy, fragmented and pyknosed nuclei after 24 h of 1 and 10 µM compound exposure (**43d**) in HCT116 cells. Average data of two biological duplicates are shown. (**D**). Fractions of red (G1), green (G2/M) or yellow (S) cells after 24 h of compound exposure (**21i** [1 µM, 5 µM, 10 µM], **43d** [1 µM, 5 µM, 10 µM], **42d** [1, 5, 10 µM], **1** [1, 5, 10 µM], milciclib [1, 10 µM]) in comparison to cells exposed to 0.1 % DMSO. Error bars show SEM of two biological replicates. (**E**). Fluorescence Image of HCT116-FUCCI cells after 48 h of compounds exposure (**21i** [10 µM], **43d** [10 µM]) in comparison to cells exposed to milciclib [1 µM] and cells exposed to 0.1 % DMSO.

Since the most potent and selective PCTAIRE inhibitors harbored an ester linkage, we assessed metabolic stability using liver microsomes. The lead structure **1** was not included in this assay due to its promiscuous behaviour. Since **1** is presumably not suitable as a tool compound to study the effect of selective kinase inhibition in an in vivo application, we focused on the newly synthesized compounds **21i** and **43d**. The compounds were tested against an activated microsome mix derived from the liver of Sprague-Dawley rats, as previously reported [50]. **21i** and **43d** were incubated at 37 °C for 60 min and the amount of unmetabolized compound was determined in 15 min steps, using high-performance liquid chromatography (HPLC). These experiments revealed an increased metabolic stability for **43d** in comparison to **21i**, thus replacing the methyl ester in **21i** with a *tert*-butyl ester (Figure 9A). After an incubation time of 60 min, only 16% of **21i** were detected; however, microsomal stability was significantly increased to 79% for the *tert*-butyl ester **43d**. 

### 2.6. Inhibition of CDK16 Led to Cell Cycle Arrest in G2/M

Expression of CDK16 has been shown to be cell cycle dependent with a peak in S and G2 phases [25]. Moreover, knockdown of CDK16 in different cancer cell lines like SCC has been shown to lead to late G2/M phase arrest followed by apoptosis, an effect that was mediated through regulation of the tumor suppressor p27 [28]. We therefore performed a cell-based assay in liver cells using the fluorescent ubiquitination-based cell cycle reporter (FUCCI) system [51,52]. This technology enables the detection of cell cycle states (G1, G2/M, or S phase) on a single cell level. Additionally, a viability assessment, as described by Tjaden et. al. [53], was performed using Hoechst33342 as a nuclear marker. Compounds **21i** and **43d** decreased the cell count at all concentrations tested (1, 5, 10 µM) in a dose-dependent manner, in comparison to cells treated with DMSO (0.1 %) shortly after treatment (Figure 9B), and with an increasing effect over time. After 72 h, both compounds showed less than 50% cells compared to the control suggesting attenuated cell growth. The nuclear gating, based on a machine learning algorithm in healthy, pyknosed, and fragmented nuclei [53], revealed for cells treated with 1 µM of **43d** on average 10% of pyknosed nuclei, an irreversible condensation of chromatin undergoing necrosis or apoptosis. At 10 µM of the same compound, the pyknosed nuclei rate was even higher, on average 45% (Figure 9C). An increase in cells including a pyknosed nucleus indicate apoptotic cell death, due to typical morphological changes like DNA condensation [54]. These results support the hypothesis of Yanagi et. al. that the inhibition of CDK16, which has been linked to up-regulation of p27, induces apoptosis. The compounds **21i**, **42d**, and **43d** led to the accumulation of cells in G2/M phases at all three concentrations tested (1 µM, 5 µM, 10 µM) (Figure 9D,E). The most potent and selective inhibitor of our series, **43d,** had the most significant impact on the cell cycle. Milciclib is a published CDK2 inhibitor and was used as a reference compound [55]. It led to G1 cell cycle arrest at both tested concentrations. In comparison, the lead structure compound **1**, which displays no family or kinome wide-selectivity, had no effect on the cell cycle at all three concentrations tested. At later time points, the G2/M phase arrest was no longer detectable (Table S7). This can be explained due to the increasing amount of apoptotic cells. This observation is also consistent with CDK16 knockout data that have been shown to lead to a G2/M phase arrest followed by apoptotic cell death. 

## 3. Discussion

We developed a new series of 3-amino-1*H*-pyrazole-based kinase inhibitors, based on the scaffold of the promiscuous inhibitor **1**. Selectivity screens against a representative set of 100 kinases revealed that small modifications, especially on the pyrazole ring, had significant effects on the selectivity of the new synthesized compounds. Alkyl residues on the pyrazole led to non-selective inhibitors, whereby the introduction of an amide moiety instead of the ester linkage was not tolerated and yielded in inactive compounds. The introduction of a methyl ester caused a dramatic reduction of potential hits while maintaining high potency on CDK16. The replacement of the methyl ester to an *iso*-propyl or *tert*-butyl ester increased the stabilization on CDK16 and led to a great selectivity profile in our in-house DSF panel, screening over one hundred kinases representing the human kinome. The cellular potency against CDK16 was determined for all new synthesized compounds, using the NanoBRET technology. The optimization of the promiscuous scaffold led to **42d** and **43d**, which were highly potent on CDK16 with a comparable potency to staurosporine or the lead structure **1**, which had reported low nM activity in enzyme-based assays. Further profiling against the whole CDK family revealed that **43d** only targeted the PCTAIRE and PFTAIRE subfamily with low nanomolar activity in cells. All members of this family belong to the dark kinome of understudied kinases. **43d** showed good metabolic stability at the compound induced cell cycle arrest and apoptosis in agreement with genetic knockout studies of CDK16. Thus, the reported synthesis and discovery of the tool compound **43d** provides a well characterized inhibitor for cell-based mechanistic studies on the PCTAIRE family of CDKs.

## 4. Materials and Methods

### 4.1. Differential Scanning Fluorimetry Assay

Recombinant protein kinase domains with a concentration of 2 μM were mixed with a 10 μM compound solution in DMSO, 20 mM HEPES, pH 7.5, and 500 mM NaCl. SYPRO Orange (5000×, Invitrogen) was added as a fluorescence probe (1 µl per mL). Subsequently, temperature-dependent protein unfolding profiles were measured, using the QuantStudio™ 5 realtime PCR machine (Thermo Fisher, Waltham, MA, USA). Excitation and emission filters were set to 465 and 590 nm. The temperature was raised with a step rate of 3 °C per minute. Data points were analyzed with the internal software (Thermal Shift SoftwareTM Version 1.4, Thermo Fisher) using the Boltzmann equation to determine the inflection point of the transition curve [56]. Differences in melting temperature are given as ΔTm values in °C. Measurements were performed in duplicates.

### 4.2. Cellular Thermal Shift Assay

Compressed CETSA MS assays, also known as PISA (Proteome Integral Stability Alteration assay), were performed as previously described by Chernobrovkin et. al., with some minor changes [57]. K562 cells were obtained from ATCC. K562 cells were mixed with an equal volume of compound solution, final concentartion of 30 µM, in a buffer (20 mM HEPES, 138 mM NaCl, 5 mM KCl, 2 mM CaCl_2_, 1 mM MgCl_2_, pH 7.4). One percent and DMSO was used as a control. Cells were incubated at +37 °C for 60 min. The cell suspension was split into 12 aliquots which were heated at a different temperature between 44 and 66 °C for 3 min. After the heating, the cells were lysed through freeze–thawing them 3 times and the lysed aliquots were pooled together. Cell debris and aggregates were removed through centrifugation (20 min at 30.000× *g*).

### 4.3. NanoBRET

The assay was performed as described previously [58]. In brief: Full-length kinases were obtained as plasmids cloned in frame with a terminal NanoLuc-fusion (Promega, Madison, WI, USA), as specified in table (Table S6). Plasmids were transfected into HEK293T cells using FuGENE HD (Promega, E2312) and proteins were allowed to express for 20h. Serially diluted inhibitor and NanoBRET Kinase Tracer (Promega) at a concentration determined previously as the K_D,app_ (Table S5) were pipetted into white 384-well plates (Greiner 781207) using an Echo acoustic dispenser (Labcyte, San Jose, CA, USA). The corresponding protein-transfected cells were added and reseeded at a density of 2.5 × 105 cells/mL after trypsinization and resuspending in Opti-MEM without phenol red (Life Technologies, Carlsbad, CA, USA). The system was allowed to equilibrate for 2 h at 37 °C/5% CO_2_ prior to BRET measurements. To measure BRET, NanoBRET NanoGlo Substrate + Extracellular NanoLuc Inhibitor (Promega, N2540) was added as per the manufacturer’s protocol, and filtered luminescence was measured on a PHERAstar plate reader (BMG Labtech, Ortenberg, Germany) equipped with a luminescence filter pair (450 nm BP filter (donor) and 610 nm LP filter (acceptor)). Competitive displacement data were then graphed using GraphPad Prism 9 software using a normalized 3-parameter curve fit with the following equation: Y = 100/(1 + 10^(X–LogEC50)).

### 4.4. Microsomal Stability Assay

The solubilized test compound (5 μL, final concentration 10 µM) was preincubated at 37 °C in 432 μL of phosphate buffer (0.1 M, pH 7.4) together with 50 μL NADPH regenerating system (30 mM glucose 6 phosphate, 4 U/mL glucose 6 phosphate dehydrogenase, 10 mM NADP, 30 mM MgCl_2_). After 5 min, the reaction was started by the addition of 13 μL of microsome mix from the liver of Sprague−Dawley rats (Invitrogen; 20 mg protein/mL in 0.1 M phosphate buffer) in a shaking water bath at 37 °C. The reaction was stopped by adding 500 μL of ice cold methanol at 0, 15, 30 and 60 min. The samples were centrifuged at 5000× *g* for 5 min at 4 °C, and the supernatants were analyzed and test compound was quantified by HPLC: The composition of the mobile phase is adapted to the test compound in a range of MeOH 40–90% and water (0.1% formic acid) 10–60%; flow rate: 1 mL/min; stationary phase: Purospher^®^ STAR, RP18, 5 μm, 125 × 4, precolumn: Purospher^®^ STAR, RP18, 5 μm, 4 × 4; detection wavelength: 254 and 280 nm; injection volume: 50 μL. Control samples were performed to check the test compound’s stability in the reaction mixture: first control was without NADPH, which is needed for the enzymatic activity of the microsomes, second control was with inactivated microsomes (incubated for 20 min at 90 °C), and third control was without test compound (to determine the baseline). The amounts of the test compound were quantified by an external calibration curve. Data are expressed as the mean ± SEM remaining compound from three independent experiments.

### 4.5. FUCCI Cell Cycle Assay

To validate the influence of the compounds on the cell cycle, a florescent ubiquitination-based cell cycle indicator (FUCCI) assay was performed as described previously [53]. In Brief, HCT116-FUCCI cells, stably expressing the FUCCI system, introduced by the Sleeping Beauty transposon system [53,59], were seeded at a density of 1250 cells per well in a 384 well plate (Cell culture microplate, PS, f-bottom, µClear^®^, 781091, Greiner, Frickenhausen, Germany) in culture medium (50 µL per well) and stained additionally with 60 nM Hoechst33342 (Thermo Scientific). Fluorescence and cellular shape were measured before and after compound treatment for 72 h every 12 h using the CQ1 high-content confocal microscope (Yokogawa, Tokyo, Japan). Compounds were added directly to the cells at three different concentrations (1 µM, 5 µM and 10 µM). The following parameters were used for image acquisition: Ex 405 nm/Em 447/60 nm, 500ms, 50%; Ex 561 nm/Em 617/73 nm, 100 ms, 40%; Ex 488/Em 525/50 nm, 50 ms, 40%; Ex 640 nm/Em 685/40, 50 ms, 20 %; bright field, 300ms, 100% transmission, one centered field per well, 7 z stacks per well with 55 µm spacing. Analysis of images was performed using the CellPathfinder software (Yokogawa). The cell count was normalized against the cell count of cells treated with 0.1% DMSO. All normal gated cells were further classified in cells containing healthy, fragmented or pyknosed nuclei. Cells that showed a healthy nucleus were gated in red, green or yellow based on 11 features of the cellbody and 4 features of the cell nuclei. Error bars show SEM of biological duplicates. All data can be found in Table S7.

### 4.6. Chemistry

The synthesis of compounds will be explained in the following and the analytical data for them can be found in the Supporting Information. All commercial chemicals were purchased from common suppliers with a purity ≥95% and were used without further purification. The solvents with an analytical grade were obtained from VWR Chemicals and Merck and all dry solvents from Acros Organics. All reactions were proceeded under an argon atmosphere. The thin layer chromatography was done with silica gel on aluminium foils (60 Å pore diameter) obtained from Macherey-Nagel and visualized with ultraviolet light (λ = 254 and 365 nm). The purification of the compounds was done by flash chromatography. A puriFlash XS 420 device with a UV-VIS multiwave detector (200−400 nm) from Interchim was used with pre-packed normal-phase PF-SIHP silica columns with particle sizes of 15 and 30 μm (Interchim). The nuclear magnetic resonance spectroscopy (NMR) was performed on a DPX250, AV300, AV400 or AV500 MHz spectrometers from Bruker. Chemical shifts (δ) are reported in parts per million (ppm). DMSO-d6 was used as a solvent, and the spectra were calibrated to the solvent signal: 2.50 ppm (^1^H NMR) or 39.52 ppm (^13^C NMR) for DMSO-d6. Coupling constants (*J*) were reported in hertz (Hz) and multiplicities were designated as followed: s (singlet), d (doublet), t (triplet), q (quartet), m (multiplet). Mass spectra were measured on a Surveyor MSQ device from ThermoFisher measuring in the positive- or negative-ion mode. Final compounds were additionally characterized by HRMS using a MALDI LTQ Orbitrap XL from ThermoScientific. The purity of the final compounds was determined by HPLC using method A: an Agilent 1260 Infinity II device with a 1260 DAD HS detector (G7117C; 254 nm, 280 nm, 310 nm) and a LC/MSD device (G6125B, ESI pos. 100–1000). The compounds were analyzed on a Poroshell 120 EC-C18 (Agilent, 3 × 150 mm, 2.7 µm) reversed phase column using 0.1% formic acid in water (A) and 0.1% formic acid in acetonitrile (B) as a mobile phase. The following gradient was used: 0 min 5% B–2 min 5% B–8 min 98% B–10 min 98% B (flow rate of 0.5 mL/min). Method B: an Agilent 1260 Infinity II device, with a 1260 MWD detector (G7165A; 254 nm, 280 nm) and a LC/MSD device (G6125B, ESI pos. 100–1000) was used. The compounds were analyzed on an Eclipse XDB-C18 (Agilent, 4.6 × 250 mm, 5 µm) reversed phase column using 0.1% TFA in water (A) and 0.1% TFA in acetonitrile (B) as a mobile phase. The following gradient was used: 0 min. 2% B–2 min. 2% B–10 min. 98% B–15 min. 98% B–17 min. 2% B–19 min. 2% B (flow rate of 1 mL/min.). UV-detection was performed at 254, 280 and 310 nm and all compounds used for further biological characterizations showed a purity ≥95%.

General procedure 1. The 3-aminopyrazole derivative (1.1 equiv) and the corresponding 2,4-dichloropyrimidine (1.0 equiv) were dissolved in anhydrous isopropanol (0.15 M). TEA (3.0 equiv) was added and the mixture was stirred at 50–80 °C for 18–120 h. The solvent was evaporated under reduced pressure and the crude product was purified by flash chromatography using DCM/methanol or *n*-hexane/EE as an eluent.

General procedure 2. The corresponding product of general procedure 1 (1.0 equiv) and the aniline derivative (1.0 equiv) were dissolved in anhydrous ethanol (0.07 M). A catalytic amount of 1 M HCl was added and the mixture was stirred at 70 °C–reflux for 4–18 h. A solid precipitated, which was filtered and washed with ethanol to obtain the title compound.

General procedure 3. The corresponding product of general procedure 1 (1.0 equiv) and the aniline derivative (1.0 equiv) were dissolved in anhydrous ethanol (0.07 M). A catalytic amount of 1 M HCl was added and the mixture was stirred at 70 °C–reflux for 18–24 h. The solvent was evaporated under reduced pressure and the crude product was purified by flash chromatography using DCM/methanol or *n*-hexane/EE as an eluent.

General procedure 4. The corresponding product of general procedure 1 (1.0 equiv) and the primary amine (1.0 equiv) were dissolved in anhydrous ethanol (0.07 M). TEA (3.0 equiv) was added and the mixture was stirred at 80–120 °C for 3–10 h under microwave irradiation. The solvent was evaporated under reduced pressure and the crude product was purified by flash chromatography using DCM/methanol or *n*-hexane/EE as an eluent.

2-bromo-*N*-(3-cyclopropyl-1*H*-pyrazol-5-yl)pyrimidin-4-amine (**8**). The title compound was prepared according to the general procedure 1, using 5-cyclopropyl-1*H*-pyrazol-3-amine and 2,4-dichloropyrimidine. The mixture was stirred at 55 °C for 48 h to obtain the product (133 mg, 35%) as a white solid. 1H NMR (250 MHz, DMSO-d6) δ 12.17 (s, 1H), 10.26 (s, 1H), 8.08 (d, J = 5.9 Hz, 1H), 7.26 (s, 1H), 5.96 (s, 1H), 1.95–1.81 (m, 1H), 0.98–0.88 (m, 2H), 0.73–0.62 (m, 2H). 13C NMR (126 MHz, DMSO) δ 160.36, 158.81, 151.52, 151.40, 147.27, 105.57, 92.64, 7.75, 6.69. MS-ESI *m*/*z* [M + H]^+^: calcd 281.1, found 280.0.

2-chloro-*N*-(3-cyclopropyl-1*H*-pyrazol-5-yl)-5-methylpyrimidin-4-amine (**9**). The title compound was prepared according to the general procedure 1, using 5-cyclopropyl-1*H*-pyrazol-3-amine and 2,4-dichloro-5-methylpyrimidine. The mixture was stirred at 80 °C for 48 h to obtain the product (366 mg, 40%) as a beige solid. ^1^H NMR (300 MHz, DMSO-d6) δ 12.17 (s, 1H), 9.27 (s, 1H), 7.97 (d, *J* = 1.0 Hz, 1H), 6.26 (s, 1H), 2.11 (d, *J* = 1.0 Hz, 3H), 1.97–1.85 (m, 1H), 0.98–0.89 (m, 2H), 0.74–0.66 (m, 2H). ^13^C NMR (75 MHz, DMSO-d6) δ 159.78, 156.99, 155.86, 113.90, 94.84, 13.37, 7.66, 6.97. MS-ESI *m*/*z* [M–H]^–^: calcd 248.7, found 248.0. 

2,5-dichloro-*N*-(3-cyclopropyl-1*H*-pyrazol-5-yl)pyrimidin-4-amine (**10**). The title compound was prepared according to the general procedure 1, using 5-cyclopropyl-1*H*-pyrazol-3-amine and 2,4,5-trichloropyrimidine. The mixture was stirred at 60 °C for 48 h to obtain the product (866 mg, 89%) as a white solid. 1H NMR (300 MHz, DMSO-d6) δ 12.33 (s, 1H), 9.65 (s, 1H), 8.32 (s, 1H), 6.19 (s, 1H), 1.99–1.82 (m, 1H), 0.99–0.89 (m, 2H), 0.74–0.66 (m, 2H). 13C NMR (75 MHz, DMSO) δ 157.05, 156.86, 155.14, 145.81, 145.55, 113.13, 95.57, 7.72, 6.79. MS-ESI *m*/*z* [M–H]^–^: calcd 269.1, found 268.1.

*tert*-butyl 4-((4-((3-cyclopropyl-1*H*-pyrazol-5-yl)amino)pyrimidin-2-yl)amino)phenethyl-carbamate (**11a**). The title compound was prepared according to the general procedure 2, using **5** and *tert*-butyl 4-aminophenethylcarbamate. The mixture was stirred for 4 h under reflux to obtain the product (155 mg, 67%) as a white solid. 1H NMR (300 MHz, DMSO-d6) δ 12.42 (s, 1H), 11.18 (s, 1H), 10.39 (s, 1H), 7.91 (d, J = 5.8 Hz, 1H), 7.40 (d, J = 7.8 Hz, 2H), 7.27 (d, J = 8.2 Hz, 2H), 6.91 (t, J = 5.4 Hz, 1H), 6.43 (s, 1H), 6.06 (s, 1H), 3.17 (q, J = 6.9 Hz, 2H), 2.74 (t, J = 7.5 Hz, 2H), 1.90–1.74 (m, 1H), 1.37 (s, 9H), 0.99–0.87 (m, 2H), 0.54 (s, 2H). 13C NMR (75 MHz, DMSO) δ 159.54, 155.52, 152.68, 152.56, 149.03, 145.91, 137.11, 134.25, 129.20, 123.96, 99.20, 93.29, 77.53, 41.44, 35.06, 28.24, 7.88, 6.70. MS-ESI *m*/*z* [M + H]^+^: calcd 436.5, found 436.4. HRMS *m*/*z* [M + H]^+^: calcd 436.2456, found 436.2446. HPLC: t_R_ = 7.57, purity ≥ 95% (UV: 254/280 nm).

*tert*-butyl 3-((4-((3-cyclopropyl-1*H*-pyrazol-5-yl)amino)pyrimidin-2-yl)amino)phenethyl-carbamate (**11b**). The title compound was prepared according to the general procedure 3, using **5** and *tert*-butyl 3-aminophenethylcarbamate. The mixture was stirred for 18 h under reflux to obtain the product (29 mg, 19%) as a colorless oil. 1H NMR (300 MHz, DMSO-d6) δ 11.97 (s, 1H), 9.48 (s, 1H), 8.97 (s, 1H), 7.98 (d, J = 5.8 Hz, 1H), 7.66 (d, J = 8.2 Hz, 1H), 7.50 (s, 1H), 7.16 (t, J = 7.8 Hz, 1H), 6.86 (t, J = 5.6 Hz, 1H), 6.75 (d, J = 7.5 Hz, 1H), 6.58–6.09 (m, 2H), 3.21–3.07 (m, 2H), 2.66 (t, J = 7.6 Hz, 2H), 1.93–1.78 (m, 1H), 1.37 (s, 9H), 0.89 (d, J = 8.2 Hz, 2H), 0.74–0.62 (m, 2H). 13C NMR (75 MHz, DMSO) δ 159.61, 155.90, 155.54, 140.98, 139.54, 128.28, 121.20, 119.14, 116.81, 98.14, 92.99, 77.51, 48.61, 41.66, 35.89, 28.27, 7.66. MS-ESI *m*/*z* [M + H]^+^: calcd 436.5, found 436.4. HRMS *m*/*z* [M + H]^+^: calcd 436.2456, found 436.2452. HPLC: t_R_ = 7.58, purity ≥ 95% (UV: 254/280 nm).

*tert*-butyl (5-((4-((3-cyclopropyl-1*H*-pyrazol-5-yl)amino)pyrimidin-2-yl)amino)pentyl) carbamate (**11c**). The title compound was prepared according to the general procedure 4, using **5** and *tert-*butyl (5-aminopentyl)carbamate. The mixture was stirred for 5 h at 90 °C to obtain the product (37 mg, 42%) as a colorless oil. 1H NMR (300 MHz, DMSO-d6) δ 11.92 (s, 1H), 9.31 (s, 1H), 7.77 (d, J = 5.7 Hz, 1H), 6.74 (t, J = 5.9 Hz, 1H), 6.65 (s, 1H), 6.30–5.97 (m, 2H), 3.21 (q, J = 6.7 Hz, 2H), 2.98–2.84 (m, 2H), 1.91–1.76 (m, 1H), 1.61–1.43 (m, 2H), 1.43–1.33 (m, 11H), 1.33–1.20 (m, 2H), 0.99–0.82 (m, 2H), 0.76–0.60 (m, 2H). 13C NMR (75 MHz, DMSO) δ 162.30, 162.02, 155.99, 155.57, 123.51, 95.71, 77.29, 40.66, 35.77, 30.76, 29.33, 28.98, 28.26, 23.93, 7.65. MS-ESI *m*/*z* [M + H]^+^: calcd 402.4, found 402.2. HRMS *m*/*z* [M + H]^+^: calcd 402.2612, found 402.2622. HPLC: t_R_ = 7.37, purity ≥ 95% (UV: 254/280 nm).

*tert*-butyl (2-(2-((4-((3-cyclopropyl-1*H*-pyrazol-5-yl)amino)pyrimidin-2-yl)amino)ethoxy)-ethyl)carbamate (**11d**). The title compound was prepared according to the general procedure 4, using **5** and *tert-*butyl (2-(2-aminoethoxy)ethyl)carbamate. The mixture was stirred for 7 h at 90 °C to obtain the product (43 mg, 30%) as a colorless oil. 1H NMR (300 MHz, DMSO-d6) δ 11.92 (s, 1H), 9.34 (s, 1H), 7.79 (d, J = 5.7 Hz, 1H), 6.92–6.50 (m, 2H), 6.30–6.00 (m, 2H), 3.56–3.46 (m, 2H), 3.45–3.36 (m, 4H), 3.09 (q, J = 5.9 Hz, 2H), 1.91–1.76 (m, 1H), 1.37 (s, 9H), 0.95–0.83 (m, 2H), 0.72–0.63 (m, 2H). 13C NMR (75 MHz, DMSO) δ 161.92, 159.58, 156.01, 155.61, 95.89, 92.13, 77.61, 69.12, 40.47, 28.23, 7.84, 7.68. MS-ESI *m*/*z* [M + H]^+^: calcd 404.5, found 404.4. HRMS *m*/*z* [M + H]^+^: calcd 404.2405, found 404.2405. HPLC: t_R_ = 5.46, purity ≥ 95% (UV: 254/280 nm).

*tert*-butyl (6-((4-((3-cyclopropyl-1*H*-pyrazol-5-yl)amino)pyrimidin-2-yl)amino)hexyl)-carbamate (**11e**). The title compound was prepared according to the general procedure 4, using **5** and *tert-*butyl (6-aminohexyl)carbamate. The mixture was stirred for 7 h at 90 °C to obtain the product (66 mg, 45%) as a colorless oil. 1H NMR (300 MHz, DMSO-d6) δ 11.87 (s, 1H), 9.29 (s, 1H), 7.77 (d, J = 5.6 Hz, 1H), 6.73 (t, J = 5.7 Hz, 1H), 6.62 (s, 1H), 6.35–6.00 (m, 2H), 3.44–3.28 (m, 2H), 3.28–3.14 (m, 2H), 2.89 (q, J = 6.5 Hz, 2H), 1.89–1.77 (m, 1H), 1.56–1.44 (m, 2H), 1.36 (s, 9H), 1.33–1.22 (m, 4H), 0.97–0.83 (m, 2H), 0.73–0.60 (m, 2H). 13C NMR (75 MHz, DMSO) δ 162.06, 159.61, 156.03, 155.59, 95.62, 77.28, 69.07, 41.18, 40.67, 29.57, 29.33, 28.26, 26.39, 26.20, 7.66. MS-ESI *m*/*z* [M + H]^+^: calcd 416.5, found 416.5. HRMS *m*/*z* [M + H]^+^: calcd 416.2769, found 416.2768. HPLC: t_R_ = 7.557, purity ≥ 95% (UV: 254/280 nm).

*tert*-butyl 4-(((4-((3-cyclopropyl-1*H*-pyrazol-5-yl)amino)pyrimidin-2-yl)amino)methyl)-benzylcarbamate (**11f**). The title compound was prepared according to the general procedure 4, using **5** and *tert-*butyl 4-(aminomethyl)benzylcarbamate. The mixture was stirred for 6 h at 90 °C to obtain the product (22 mg, 24%) as a colorless oil. 1H NMR (300 MHz, DMSO-d6) δ 11.97 (s, 1H), 9.35 (s, 1H), 7.79 (d, J = 5.7 Hz, 1H), 7.38–7.10 (m, 6H), 6.26–5.92 (m, 2H), 4.46 (d, J = 6.2 Hz, 2H), 4.08 (d, J = 6.2 Hz, 2H), 1.88–1.73 (m, 1H), 1.38 (s, 9H), 0.93–0.79 (m, 2H), 0.71–0.53 (m, 2H). 13C NMR (75 MHz, DMSO) δ 161.99, 159.65, 156.01, 155.77, 139.25, 138.28, 126.81, 96.08, 77.69, 43.72, 43.15, 28.24, 7.65. MS-ESI *m*/*z* [M + H]^+^: calcd 436.5, found 436.4. HRMS *m*/*z* [M + H]^+^: calcd 436.2456, found 436.2448. HPLC: t_R_ = 7.45, purity ≥ 95% (UV: 254/280 nm).

*tert*-butyl 4-((4-((3-cyclopropyl-1*H*-pyrazol-5-yl)amino)-5-methylpyrimidin-2-yl)amino)-phenethylcarbamate (**12a**). The title compound was prepared according to the general procedure 3, using **6** and *tert*-butyl 4-aminophenethylcarbamate. The mixture was stirred for 18 h under reflux to obtain the product (85 mg, 90%) as a colorless oil. 1H NMR (300 MHz, DMSO-d6) δ 12.56 (s, 1H), 10.61 (s, 1H), 10.16 (s, 1H), 7.88 (s, 1H), 7.38 (d, J = 7.2 Hz, 2H), 7.19 (d, J = 7.3 Hz, 2H), 6.90 (s, 1H), 6.07 (s, 1H), 3.21–3.07 (m, 2H), 2.77–2.63 (m, 2H), 2.11 (s, 3H), 1.94–1.81 (m, 1H), 0.99–0.87 (m, 2H), 0.64–0.51 (m, 2H). 13C NMR (75 MHz, DMSO) δ 160.12, 155.41, 151.27, 146.40, 145.12, 140.12, 136.01, 134.68, 128.98, 122.29, 107.06, 95.10, 77.40, 41.38, 34.96, 28.16, 13.26, 7.84, 6.78. MS-ESI *m*/*z* [M + H]^+^: calcd 450.6, found 450.4. HRMS *m*/*z* [M + H]^+^: calcd 450.2612, found 450.2603. HPLC: t_R_ = 7.54, purity ≥ 95% (UV: 254/280 nm).

*tert*-butyl 3-((4-((3-cyclopropyl-1*H*-pyrazol-5-yl)amino)-5-methylpyrimidin-2-yl)amino)-phenethylcarbamate (**12b**). The title compound was prepared according to the general procedure 3, using **6** and *tert*-butyl 3-aminophenethylcarbamate. The mixture was stirred for 18 h under reflux to obtain the product (34 mg, 36%) as a colourless oil. 1H NMR (300 MHz, DMSO-d6) δ 12.09 (s, 1H), 9.12 (s, 2H), 7.86 (s, 1H), 7.62 (d, J = 8.2 Hz, 1H), 7.45 (s, 1H), 7.14 (t, J = 7.8 Hz, 1H), 6.86 (t, J = 5.7 Hz, 1H), 6.72 (d, J = 7.5 Hz, 1H), 5.83 (s, 1H), 3.33 (s, 3H), 3.21–3.05 (m, 2H), 2.72–2.59 (m, 2H), 1.93–1.79 (m, 1H), 1.37 (s, 9H), 0.96–0.81 (m, 2H), 0.72–0.61 (m, 2H). 13C NMR (75 MHz, DMSO) δ 158.12, 155.52, 155.14, 141.21, 139.56, 128.35, 120.81, 118.44, 116.14, 105.46, 77.49, 41.65, 35.91, 28.26, 13.33, 7.68. MS-ESI *m*/*z* [M + H]^+^: calcd 450.6, found 450.4. HRMS *m*/*z* [M + H]^+^: calcd 450.2612, found 450.2607. HPLC: t_R_ = 7.63, purity ≥ 95% (UV: 254/280 nm).

*tert*-butyl (5-((4-((3-cyclopropyl-1*H*-pyrazol-5-yl)amino)-5-methylpyrimidin-2-yl)amino)-pentyl)carbamate (**12c**). The title compound was prepared according to the general procedure 4, using **6** and *tert-*butyl (5-aminopentyl)carbamate. The mixture was stirred for 6 h at 90 °C to obtain the product (5 mg, 5%) as a colorless oil. 1H NMR (300 MHz, DMSO-d6) δ 12.06 (s, 1H), 8.58 (s, 1H), 7.65 (s, 1H), 6.75 (t, J = 5.5 Hz, 1H), 6.32 (s, 1H), 3.18 (q, J = 6.7 Hz, 2H), 2.90 (q, J = 6.5 Hz, 2H), 1.96 (s, 3H), 1.90–1.78 (m, 1H), 1.55–1.45 (m, 2H), 1.36 (s, 9H), 1.32–1.21 (m, 4H), 0.97–0.81 (m, 2H), 0.69–0.61 (m, 2H). MS-ESI *m*/*z* [M + H]^+^: calcd 416.5, found 416.2. HRMS *m*/*z* [M + H]^+^: calcd 416.2769, found 416.2769. HPLC: t_R_ = 7.43, purity ≥ 95% (UV: 254/280 nm).

*tert*-butyl (2-(2-((4-((3-cyclopropyl-1*H*-pyrazol-5-yl)amino)-5-methylpyrimidin-2-yl)amino)ethoxy)ethyl)carbamate (**12d**). The title compound was prepared according to the general procedure 4, using **6** and *tert-*butyl (2-(2-aminoethoxy)ethyl)carbamate. The mixture was stirred for 10 h at 90 °C to obtain the product (7 mg, 7%) as a colorless oil. 1H NMR (300 MHz, DMSO-d6) δ 8.66 (s, 1H), 7.66 (s, 1H), 6.92–6.70 (m, 2H), 6.15 (s, 1H), 3.49 (t, J = 5.8 Hz, 2H), 3.44–3.37 (m, 2H), 3.08 (q, J = 5.9 Hz, 2H), 2.94–2.84 (m, 2H), 1.97 (s, 3H), 1.90–1.78 (m, 1H), 1.37 (s, 9H), 0.96–0.79 (m, 2H), 0.71–0.62 (m, 2H). 13C NMR (75 MHz, DMSO) δ 160.77, 155.55, 116.98, 102.86, 77.61, 69.28, 69.12, 41.33, 40.57, 28.22, 23.63, 13.19, 7.70. MS-ESI *m*/*z* [M + H]^+^: calcd 418.5, found 418.4. HRMS *m*/*z* [M + H]^+^: calcd 418.2561, found 418.2559. HPLC: t_R_ = 7.18, purity ≥ 95% (UV: 254/280 nm).

*tert*-butyl (6-((4-((3-cyclopropyl-1*H*-pyrazol-5-yl)amino)-5-methylpyrimidin-2-yl)amino)hexyl)carbamate (**12e**). The title compound was prepared according to the general procedure 4, using **6** and *tert-*butyl (6-aminohexyl)carbamate. The mixture was stirred for 6 h at 90 °C to obtain the product (5 mg, 5%) as a colourless oil. 1H NMR (300 MHz, DMSO-d6) δ 12.17 (s, 1H), 8.57 (s, 1H), 7.67 (s, 1H), 6.78 (t, J = 5.0 Hz, 1H), 6.44 (s, 1H), 3.21 (q, J = 6.7 Hz, 2H), 2.91 (q, J = 6.5 Hz, 2H), 1.98 (s, 3H), 1.92–1.80 (m, 1H), 1.60–1.47 (m, 2H), 1.38 (s, 9H), 1.33–1.24 (m, 4H), 0.96–0.82 (m, 2H), 0.71–0.60 (m, 2H). MS-ESI *m*/*z* [M + H]^+^: calcd 430.6, found 430.3. HRMS *m*/*z* [M + H]^+^: calcd 430.2925, found 430.2924. HPLC: t_R_ = 7.69, purity ≥ 95% (UV: 254/280 nm).

*tert*-butyl 4-((5-chloro-4-((3-cyclopropyl-1*H*-pyrazol-5-yl)amino)pyrimidin-2-yl)amino)phenethylcarbamate (**13a**). The title compound was prepared according to the general procedure 3, using **7** and *tert*-butyl 4-aminophenethylcarbamate. The mixture was stirred for 18 h under reflux to obtain the product (20 mg, 16%) as a yellow oil. 1H NMR (300 MHz, DMSO-d6) δ 12.25 (s, 1H), 9.31 (s, 1H), 8.58 (s, 1H), 8.09 (s, 1H), 7.54 (d, J = 8.4 Hz, 2H), 7.07 (d, J = 8.1 Hz, 2H), 6.85 (t, J = 5.6 Hz, 1H), 6.16 (s, 1H), 3.11 (q, J = 6.9 Hz, 2H), 2.63 (t, J = 7.6 Hz, 2H), 2.00–1.80 (m, 1H), 1.37 (s, 9H), 0.98–0.86 (m, 2H), 0.74–0.62 (m, 2H). 13C NMR (75 MHz, DMSO) δ 157.86, 155.52, 155.10, 154.26, 138.41, 132.39, 128.52, 119.32, 103.36, 77.46, 41.73, 34.99, 28.26, 7.73. MS-ESI *m*/*z* [M + H]^+^: calcd 471.0, found 470.4. HRMS *m*/*z* [M + H]^+^: calcd 470.2066, found 470.2061. HPLC: t_R_ = 8.70, purity ≥ 95% (UV: 254/280 nm).

*tert*-butyl (5-((5-chloro-4-((3-cyclopropyl-1*H*-pyrazol-5-yl)amino)pyrimidin-2-yl)amino)pentyl)carbamate (**13c**). The title compound was prepared according to the general procedure 4, using **7** and *tert-*butyl (5-aminopentyl)carbamate. The mixture was stirred for 8 h at 80 °C to obtain the product (50 mg, 52%) as a colorless oil. 1H NMR (300 MHz, DMSO-d6) δ 12.11 (s, 1H), 8.42 (s, 1H), 7.91 (s, 1H), 7.02 (s, 1H), 6.74 (t, J = 5.6 Hz, 1H), 6.31 (s, 1H), 3.27–3.13 (m, 2H), 2.90 (q, J = 6.5 Hz, 2H), 1.93–1.80 (m, 1H), 1.58–1.46 (m, 2H), 1.43–1.34 (m, 11H), 1.33–1.21 (m, 2H), 0.98–0.85 (m, 2H), 0.73–0.61 (m, 2H). 13C NMR (75 MHz, DMSO) δ 160.41, 158.17, 155.59, 154.56, 101.48, 93.37, 77.29, 40.99, 29.30, 28.77, 28.26, 23.87, 7.69. MS-ESI *m*/*z* [M + H]^+^: calcd 437.0, found 436.4. HRMS *m*/*z* [M + H]^+^: calcd 436.2222, found 436.2218. HPLC: t_R_ = 7.56, purity ≥ 95% (UV: 254/280 nm).

N^2^-(6-aminohexyl)-N^4^-(3-cyclopropyl-1*H*-pyrazol-5-yl)pyrimidine-2,4-diamine (**14**). **8e** (50 mg, 0.1 mmol) was dissolved in anhydrous DCM (4 mL). TFA (549 mg, 4.8 mmol) was added at 0°C and the reaction mixture was allowed to warm up to rt overnight. The solvent was evaporated under reduced pressure. The residue was dissolved in methanol and neutralized with saturated K_2_CO_3_ solution. The solvent was again evaporated under reduced pressure and the crude product was purified by flash chromatography using H_2_O/acetonitrile as an eluent to obtain the desired product with impurities. The product was used without further purification. MS-ESI *m*/*z* [M + H]^+^: calcd 316.4, found 316.2.

*N*-(6-((4-((3-cyclopropyl-1*H*-pyrazol-5-yl)amino)pyrimidin-2-yl)amino)hexyl)acetamide (**15**). Acetic acid (7 µL, 0.1 mmol) and HATU (55 mg, 0.1 mmol) were dissolved in anhydrous DMF (4 mL). DIPEA (37 mg 0.3 mmol) was added and the resulting mixture was stirred at rt for 1 h. **11** (38 mg, 0.1 mmol) was added and the reaction mixture was stirred at rt for further 18 h. The solvent was evaporated under reduced pressure and the crude product was purified by flash chromatography using DCM/ethanol as an eluent to obtain the desired product (13 mg, 30%) as a colorless oil. 1H NMR (300 MHz, DMSO-d6) δ 12.42 (s, 1H), 11.12 (s, 1H), 8.47 (s, 1H), 7.92–7.68 (m, 2H), 6.57–6.17 (m, 2H), 3.39–3.21 (m, 2H), 3.00 (q, J = 6.4 Hz, 2H), 1.97–1.82 (m, 1H), 1.77 (s, 3H), 1.67–1.50 (m, 2H), 1.47–1.23 (m, 6H), 1.02–0.90 (m, 2H), 0.83–0.62 (m, 2H). 13C NMR (75 MHz, DMSO) δ 168.91, 153.77, 150.30, 146.28, 142.21, 97.72, 93.29, 40.93, 38.41, 29.14, 28.36, 26.17, 22.60, 7.86, 6.76. MS-ESI *m*/*z* [M + H]^+^: calcd 358.5, found 358.3. HRMS *m*/*z* [M + H]^+^: calcd 358.2350, found 358.2352. HPLC: t_R_ = 6.51, purity ≥ 95% (UV: 254/280 nm).

methyl 5-((2-chloropyrimidin-4-yl)amino)-1*H*-pyrazole-3-carboxylate (**18**). The title compound was prepared according to the general procedure 1, using methyl 3-amino-1*H*-pyrazole-5-carboxylate and 2,4-dichloropyrimidine. The mixture was stirred at 50 °C for 72 h to obtain the product (132 mg, 16%) as a white solid. 1H NMR (250 MHz, DMSO-d6) δ 13.70 (s, 1H), 10.63 (s, 1H), 8.20 (d, J = 5.9 Hz, 1H), 7.19–6.81 (m, 2H), 3.86 (s, 3H). 13C NMR (151 MHz, DMSO) δ 160.56, 159.39, 147.77, 133.08, 128.15, 127.41, 99.45, 52.04. MS-ESI *m*/*z* [M + Na]^+^: calcd 276.7, found 276.1. 

methyl 5-((2,5-dichloropyrimidin-4-yl)amino)-1*H*-pyrazole-3-carboxylate (**19**). The title compound was prepared according to the general procedure 1, using methyl 3-amino-1*H*-pyrazole-5-carboxylate and 2,4,5-trichloropyrimidine. The mixture was stirred at 60 °C for 72 h to obtain the product (781 mg, 84%) as a beige solid. 1H NMR (250 MHz, DMSO-d6) δ 13.87 (s, 1H), 10.10 (s, 1H), 8.40 (s, 1H), 7.06 (s, 1H), 3.86 (s, 3H). 13C NMR (126 MHz, DMSO) δ 159.22, 156.95, 156.77, 155.60, 146.39, 133.11, 113.43, 102.23, 52.12. MS-ESI *m*/*z* [M + H]^+^: calcd 287.1, found 286.0. 

methyl 5-((2-chloroquinazolin-4-yl)amino)-1*H*-pyrazole-3-carboxylate (**20**). The title compound was prepared according to the general procedure 1, using methyl 3-amino-1*H*-pyrazole-5-carboxylate and 2,4-dichloroquinazoline. The mixture was stirred at 60 °C for 72 h to obtain the product (823 mg, 84%) as a white solid. 1H NMR (300 MHz, DMSO-d6) δ 13.88 (s, 1H), 11.12 (s, 1H), 8.67 (d, J = 8.3 Hz, 1H), 7.88 (t, J = 7.6 Hz, 1H), 7.73 (d, J = 8.3 Hz, 1H), 7.61 (t, J = 7.6 Hz, 1H), 7.35 (s, 1H), 3.89 (s, 3H). 13C NMR (75 MHz, DMSO) δ 159.32, 158.58, 156.07, 150.82, 147.41, 134.16, 132.93, 126.87, 126.77, 123.63, 113.45, 101.83, 52.11. MS-ESI *m*/*z* [M + H]^+^: calcd 304.7, found 304.1. 

methyl 5-((2-((4-(2-((*tert*-butoxycarbonyl)amino)ethyl)phenyl)amino)pyrimidin-4-yl)amino)-1*H*-pyrazole-3-carboxylate (**21a**). The title compound was prepared according to the general procedure 3, using **16** and *tert*-butyl 4-aminophenethylcarbamate. The mixture was stirred for 18 h under reflux to obtain the product (219 mg, 63%) as a light yellow solid. 1H NMR (250 MHz, DMSO-d6) δ 13.87 (s, 1H), 11.29 (s, 1H), 10.50 (s, 1H), 8.00 (d, J = 6.9 Hz, 1H), 7.47 (d, J = 8.0 Hz, 2H), 7.23 (d, J = 8.1 Hz, 2H), 7.06 (s, 1H), 6.90 (t, J = 5.3 Hz, 1H), 6.52 (s, 1H), 3.87 (s, 3H), 3.22–3.10 (m, 2H), 2.80–2.65 (m, 2H), 1.37 (s, 9H). 13C NMR (126 MHz, DMSO) δ 160.11, 155.54, 146.85, 136.35, 133.35, 129.22, 122.79, 100.87, 99.04, 77.52, 52.04, 41.52, 35.06, 28.24. MS-ESI *m*/*z* [M + H]^+^: calcd 454.5, found 454.1. HRMS *m*/*z* [M + Na]^+^: calcd 476.2017, found 476.2038. HPLC: t_R_ = 6.09, purity ≥ 95% (UV: 254/280 nm).

methyl 5-((2-((3-(2-((*tert*-butoxycarbonyl)amino)ethyl)phenyl)amino)pyrimidin-4-yl)amino)-1*H*-pyrazole-3-carboxylate (**21b**). The title compound was prepared according to the general procedure 3, using **16** and *tert-*butyl 3-aminophenethylcarbamate. The mixture was stirred for 18 h under reflux to obtain the product (178 mg, 49%) as a light yellow solid. 1H NMR (250 MHz, DMSO-d6) δ 13.85 (s, 1H), 11.33 (s, 1H), 10.54 (s, 1H), 8.01 (d, J = 6.7 Hz, 1H), 7.54–7.28 (m, 3H), 7.08 (d, J = 7.1 Hz, 1H), 6.98 (s, 1H), 6.85 (t, J = 5.6 Hz, 1H), 6.52 (s, 1H), 3.84 (s, 3H), 3.16–3.06 (m, 2H), 2.75–2.64 (m, 2H), 1.34 (s, 9H). 13C NMR (126 MHz, DMSO) δ 160.28, 159.49, 155.52, 153.45, 147.09, 140.65, 136.85, 133.51, 129.09, 125.61, 123.38, 121.02, 100.73, 99.19, 77.53, 51.93, 41.30, 35.37, 28.22. MS-ESI *m*/*z* [M + H]^+^: calcd 454.5, found 454.3. HRMS *m*/*z* [M + H]^+^: calcd 454.2197, found 454.2182. HPLC: t_R_ = 6.07, purity ≥ 95% (UV: 254/280 nm).

methyl 5-((2-((5-((*tert*-butoxycarbonyl)amino)pentyl)amino)pyrimidin-4-yl)amino)-1*H*-pyrazole-3-carboxylate (**21c**). The title compound was prepared according to the general procedure 4, using **16** and *tert-*butyl (5-aminopentyl)carbamate. The mixture was stirred for 5 h at 120 °C to obtain the product (142 mg, 45%) as a white solid. 1H NMR (400 MHz, DMSO-d6) δ 13.38 (s, 1H), 9.91 (d, J = 185.8 Hz, 1H), 7.83 (s, 1H), 7.21 (s, 1H), 6.75 (s, 2H), 6.04 (d, J = 53.4 Hz, 1H), 3.82 (s, 3H), 3.23 (q, J = 6.3 Hz, 2H), 2.91 (q, J = 6.4 Hz, 2H), 1.61–1.46 (m, 2H), 1.36 (s, 13H). 13C NMR (101 MHz, DMSO) δ 162.50, 160.04, 157.02, 156.06, 150.13, 142.02, 100.07, 96.24, 77.78, 52.27, 41.26, 40.66, 29.78, 29.39, 28.74, 24.33. MS-ESI *m*/*z* [M + H]^+^: calcd 420.5, found 420.9. HRMS *m*/*z* [M + H]^+^: calcd 420.2354, found 420.2350. HPLC: t_R_ = 5.57, purity ≥ 95% (UV: 254/280 nm).

methyl 5-((2-((2-(2-((*tert*-butoxycarbonyl)amino)ethoxy)ethyl)amino)pyrimidin-4-yl)amino)-1*H*-pyrazole-3-carboxylate (**21d**). The title compound was prepared according to the general procedure 4, using **16** and *tert*-butyl (2-(2-aminoethoxy)ethyl)carbamate. The mixture was stirred for 5 h at 120 °C to obtain the product (10 mg, 31%) as a colorless oil. 1H NMR (500 MHz, DMSO-d6) δ 13.39 (s, 1H), 9.91 (d, J = 245.4 Hz, 1H), 7.86 (s, 1H), 7.54–5.86 (m, 4H), 3.82 (s, 3H), 3.60–3.49 (m, 2H), 3.46–3.38 (m, 4H), 3.09 (q, J = 5.9 Hz, 2H), 1.36 (s, 9H). 13C NMR (126 MHz, DMSO) δ 162.14, 159.69, 156.87, 155.63, 149.28, 141.64, 132.78, 99.45, 96.01, 92.69, 77.62, 69.08, 51.72, 40.48, 28.22. MS-ESI *m*/*z* [M + H]^+^: calcd 422.5, found 422.9. HRMS *m*/*z* [M + H]^+^: calcd 422.2146, found 422.2135. HPLC: t_R_ = 5.18, purity ≥ 95% (UV: 254/280 nm).

methyl 5-((2-((6-((*tert*-butoxycarbonyl)amino)hexyl)amino)pyrimidin-4-yl)amino)-1*H*-pyrazole-3-carboxylate (**21e**). The title compound was prepared according to the general procedure 4, using **16** and *tert-*butyl (6-aminohexyl)carbamate. The mixture was stirred for 5 h at 120 °C to obtain the product (36 mg, 42%) as a colorless oil. 1H NMR (400 MHz, DMSO-d6) δ 13.37 (s, 1H), 9.90 (d, J = 169.7 Hz, 1H), 7.85 (s, 1H), 7.17 (s, 1H), 6.94–6.58 (m, 2H), 6.07 (s, 1H), 3.81 (s, 3H), 3.23 (q, J = 6.7 Hz, 2H), 2.98–2.83 (m, 2H), 1.57–1.45 (m, 2H), 1.40–1.23 (m, 15H). 13C NMR (101 MHz, DMSO) δ 162.04, 159.47, 156.64, 155.60, 149.06, 140.95, 132.46, 99.15, 95.72, 77.29, 51.72, 40.65, 29.53, 29.24, 28.27, 26.31, 26.18. MS-ESI *m*/*z* [M + H]^+^: calcd 434.5, found 435.0. HRMS *m*/*z* [M + H]^+^: calcd 434.2510, found 434.2500. HPLC: t_R_ = 5.86, purity ≥ 95% (UV: 254/280 nm).

methyl 5-((2-((4-(cyanomethyl)phenyl)amino)pyrimidin-4-yl)amino)-1*H*-pyrazole-3-carboxylate (**21f**). The title compound was prepared according to the general procedure 2, using **16** and 2-(4-aminophenyl)acetonitrile. The mixture was stirred for 18 h under reflux to obtain the product (46 mg, 61%) as a white solid. 1H NMR (300 MHz, DMSO-d6) δ 11.52 (s, 1H), 10.90 (s, 1H), 8.05 (d, J = 7.1 Hz, 1H), 7.58 (d, J = 8.1 Hz, 2H), 7.40 (d, J = 8.3 Hz, 2H), 7.01 (s, 1H), 6.56 (s, 1H), 4.08 (s, 2H), 3.88 (s, 3H). 13C NMR (75 MHz, DMSO) δ 160.20, 159.36, 152.75, 146.04, 143.59, 135.95, 133.88, 128.85, 127.98, 123.09, 119.23, 100.74, 99.31, 52.14, 21.99. MS-ESI *m*/*z* [M + H]^+^: calcd 350.4, found 350.2. HRMS *m*/*z* [M + H]^+^: calcd 350.1360, found 350.1363. HPLC: t_R_ = 6.79, purity ≥ 95% (UV: 254/280 nm).

methyl 5-((2-((4-(((*tert*-butoxycarbonyl)amino)methyl)benzyl)amino)pyrimidin-4-yl)amino)-1*H*-pyrazole-3-carboxylate (**21g**). The title compound was prepared according to the general procedure 4, using **16** and *tert-*butyl 4-(aminomethyl)benzylcarbamate. The mixture was stirred for 5 h at 120 °C to obtain the product (71 mg, 26%) as a white solid. 1H NMR (400 MHz, DMSO-d6) δ 13.39 (s, 1H), 9.95 (d, J = 203.0 Hz, 1H), 7.86 (s, 1H), 7.39–7.11 (m, 5H), 6.12 (s, 1H), 4.46 (d, J = 6.1 Hz, 2H), 4.08 (d, J = 6.1 Hz, 2H), 3.83 (s, 3H), 1.38 (s, 9H). 13C NMR (101 MHz, DMSO) δ 161.88, 159.45, 156.66, 155.78, 149.13, 141.49, 139.09, 138.33, 127.28, 126.96, 126.83, 96.28, 77.72, 51.72, 43.83, 43.15, 28.25. MS-ESI *m*/*z* [M + H]^+^: calcd 454.5, found 455.1 HRMS *m*/*z* [M + H]^+^: calcd 454.2197, found 454.2190. HPLC: t_R_ = 5.60, purity ≥ 95% (UV: 254/280 nm).

methyl 5-((2-((4-(((*tert*-butoxycarbonyl)amino)methyl)phenyl)amino)pyrimidin-4-yl)amino)-1*H*-pyrazole-3-carboxylate (**21h**). The title compound was prepared according to the general procedure 3, using **16** and *tert*-butyl 4-aminobenzylcarbamate. The mixture was stirred for 18 h under reflux to obtain the product (228 mg, 64%) as a white solid. 1H NMR (500 MHz, DMSO-d6) δ 13.18 (d, J = 722.0 Hz, 1H), 11.46 (s, 1H), 10.70 (s, 1H), 8.00 (s, 1H), 7.47 (d, J = 8.0 Hz, 2H), 7.41 (t, J = 6.2 Hz, 1H), 7.29 (d, J = 8.1 Hz, 2H), 7.21–6.97 (m, 1H), 6.50 (s, 1H), 4.16 (d, J = 6.0 Hz, 2H), 3.88 (s, 3H), 1.40 (s, 9H). 13C NMR (126 MHz, DMSO) δ 160.20, 155.83, 152.89, 143.48, 137.58, 135.01, 133.33, 127.77, 122.86, 100.87, 99.23, 77.86, 52.13, 43.05, 28.26. MS-ESI *m*/*z* [M + H]^+^: calcd 440.5, found 440.2. HRMS *m*/*z* [M + H]^+^: calcd 440.2041, found 440.2038. HPLC: t_R_ = 5.86, purity ≥ 95% (UV: 254/280 nm).

methyl 5-((2-((4-((*tert*-butoxycarbonyl)amino)benzyl)amino)pyrimidin-4-yl)amino)-1*H*-pyrazole-3-carboxylate (**21i**). The title compound was prepared according to the general procedure 4, using **16** and *tert-*butyl (4-(aminomethyl)phenyl)carbamate. The mixture was stirred for 3 h at 120 °C to obtain the product (45 mg, 17%) as a white solid. 1H NMR (400 MHz, DMSO-d6) δ 13.34 (s, 1H), 9.87 (d, J = 138.1 Hz, 1H), 9.24 (s, 1H), 7.87 (s, 1H), 7.59–6.81 (m, 6H), 6.09 (s, 1H), 4.41 (d, J = 6.1 Hz, 2H), 3.82 (s, 3H), 1.45 (s, 9H). 13C NMR (101 MHz, DMSO) δ 161.86, 159.46, 156.56, 152.80, 138.03, 134.23, 127.79, 127.51, 118.04, 96.14, 78.86, 51.71, 43.66, 28.14. MS-ESI *m*/*z* [M + H]^+^: calcd 440.5, found 441.0. HRMS *m*/*z* [M + H]^+^: calcd 440.2041, found 440.2040. HPLC: t_R_ = 5.79, purity ≥ 95% (UV: 254/280 nm).

methyl 5-((2-((4-(2-((*tert*-butoxycarbonyl)amino)ethyl)phenyl)amino)-5-chloropyrimidin-4-yl)amino)-1*H*-pyrazole-3-carboxylate (**22a**). The title compound was prepared according to the general procedure 3, using 17 and *tert*-butyl 4-aminophenethylcarbamate. The mixture was stirred for 18 h under reflux to obtain the product (14 mg, 5%) as a light yellow solid. 1H NMR (250 MHz, DMSO-d6) δ 9.92 (s, 1H), 9.88 (s, 1H), 8.23 (s, 1H), 7.50 (d, J = 7.4 Hz, 2H), 7.09 (d, J = 7.5 Hz, 2H), 7.00–6.77 (m, 2H), 3.85 (s, 3H), 3.18–3.01 (m, 2H), 2.72–2.58 (m, 2H), 1.36 (s, 9H). 13C NMR (75 MHz, DMSO) δ 160.79, 155.83, 155.40, 155.23, 151.51, 143.10, 137.10, 133.41, 128.67, 119.73, 103.59, 77.37, 51.73, 41.55, 34.90, 28.16. MS-ESI *m*/*z* [M + H]^+^: calcd 489.0, found 488.2. HRMS *m*/*z* [M + H]^+^: calcd 488.1808, found 488.1799. HPLC: t_R_ = 9.01, purity ≥ 95% (UV: 254/280 nm).

methyl 5-((2-((3-(2-((*tert*-butoxycarbonyl)amino)ethyl)phenyl)amino)-5-chloropyrimidin-4-yl)amino)-1*H*-pyrazole-3-carboxylate (**22b**). The title compound was prepared according to the general procedure 3, using 17 and *tert-*butyl 3-aminophenethylcarbamate. The mixture was stirred for 18 h under reflux to obtain the product (23 mg, 9%) as a light yellow solid. 1H NMR (300 MHz, DMSO-d6) δ 13.82–13.29 (m, 1H), 9.99 (s, 1H), 9.72 (s, 1H), 8.22 (s, 1H), 7.53 (d, J = 8.2 Hz, 1H), 7.42 (s, 1H), 7.17 (s, 1H), 6.88–6.73 (m, 2H), 6.62 (s, 1H), 3.83 (s, 3H), 3.22–3.04 (m, 2H), 2.70–2.56 (m, 2H), 1.37 (s, 9H). 13C NMR (75 MHz, DMSO) δ 157.65, 155.50, 154.62, 140.16, 139.83, 128.46, 126.57, 121.82, 118.74, 116.55, 103.68, 77.51, 51.59, 41.58, 35.77, 28.26. MS-ESI *m*/*z* [M + H]^+^: calcd 489.0, found 488.2. HRMS *m*/*z* [M + H]^+^: calcd 488.1808, found 488.1802. HPLC: t_R_ = 9.03, purity ≥ 95% (UV: 254/280 nm).

methyl 5-((2-((5-((*tert*-butoxycarbonyl)amino)pentyl)amino)-5-chloropyrimidin-4-yl)amino)-1*H*-pyrazole-3-carboxylate (**22c**). The title compound was prepared according to the general procedure 4, using **17** and *tert-*butyl (5-aminopentyl)carbamate. The mixture was stirred for 3 h at 80 °C to obtain the product (28 mg, 35%) as a white solid. 1H NMR (300 MHz, DMSO-d6) δ 13.50 (d, J = 68.2 Hz, 1H), 9.36 (d, J = 271.2 Hz, 1H), 7.99 (d, J = 26.2 Hz, 1H), 7.63–6.31 (m, 3H), 3.80 (s, 3H), 3.29–3.12 (m, 2H), 2.99–2.84 (m, 2H), 1.63–1.43 (m, 2H), 1.44–1.25 (m, 13H). 13C NMR (75 MHz, DMSO) δ 160.48, 160.03, 155.59, 155.20, 141.57, 140.72, 101.18, 95.10, 77.30, 51.49, 40.73, 29.27, 28.71, 28.25, 23.79. MS-ESI *m*/*z* [M + H]^+^: calcd 454.9, found 454.2. HRMS *m*/*z* [M + H]^+^: calcd 454.1964, found 454.1954. HPLC: t_R_ = 7.59, purity ≥ 95% (UV: 254/280 nm).

methyl 5-((2-((2-(2-((*tert*-butoxycarbonyl)amino)ethoxy)ethyl)amino)-5-chloropyrimidin-4-yl)amino)-1*H*-pyrazole-3-carboxylate (**22d**). The title compound was prepared according to the general procedure 4, using 17 and *tert*-butyl (2-(2-aminoethoxy)ethyl)carbamate. The mixture was stirred for 8 h at 80 °C to obtain the product (147 mg, 46%) as a white solid. 1H NMR (300 MHz, DMSO-d6) δ 13.53 (d, J = 61.3 Hz, 1H), 9.38 (d, J = 280.8 Hz, 1H), 8.02 (d, J = 22.6 Hz, 1H), 7.17 (d, J = 40.1 Hz, 1H), 6.77 (t, J = 5.5 Hz, 1H), 6.57 (s, 1H), 3.92–3.75 (m, 3H), 3.58–3.48 (m, 2H), 3.49–3.35 (m, 4H), 3.17–3.01 (m, 2H), 1.38 (s, 9H). 13C NMR (75 MHz, DMSO) δ 163.17, 160.84, 156.09, 155.59, 154.53, 142.03, 141.04, 101.91, 95.75, 78.10, 69.55, 69.41, 52.46, 51.95, 41.18, 28.69. MS-ESI *m*/*z* [M + H]^+^: calcd 456.9, found 456.3. HRMS *m*/*z* [M + H]^+^: calcd 457.1790, found 457.1785. HPLC: t_R_ = 7.35, purity ≥ 95% (UV: 254/280 nm).

methyl 5-((2-((6-((*tert*-butoxycarbonyl)amino)hexyl)amino)-5-chloropyrimidin-4-yl)amino)-1*H*-pyrazole-3-carboxylate (**22e**). The title compound was prepared according to the general procedure 4, using **17** and *tert-*butyl (6-aminohexyl)carbamate. The mixture was stirred for 8 h at 80 °C to obtain the product (177 mg, 54%) as a colorless oil. 1H NMR (300 MHz, DMSO-d6) δ 13.49 (d, J = 68.2 Hz, 1H), 9.33 (d, J = 284.3 Hz, 1H), 7.98 (d, J = 28.7 Hz, 1H), 7.17 (d, J = 43.4 Hz, 1H), 6.73 (t, J = 5.7 Hz, 1H), 6.65–6.41 (m, 1H), 3.90–3.76 (m, 3H), 3.29–3.14 (m, 2H), 2.95–2.83 (m, 2H), 1.60–1.44 (m, 2H), 1.39–1.20 (m, 15H). 13C NMR (75 MHz, DMSO) δ 162.58, 159.99, 155.54, 155.04, 153.78, 147.66, 132.63, 101.06, 95.10, 77.26, 51.91, 51.45, 40.72, 29.49, 29.00, 28.25, 26.23, 26.11. MS-ESI *m*/*z* [M + H]^+^: calcd 469.0, found 468.4. HRMS *m*/*z* [M + H]^+^: calcd 468.2121, found 468.2116. HPLC: t_R_ = 7.82, purity ≥ 95% (UV: 254/280 nm).

methyl 5-((2-((4-(2-((*tert*-butoxycarbonyl)amino)ethyl)phenyl)amino)quinazolin-4-yl)amino)-1*H*-pyrazole-3-carboxylate (**23a**). The title compound was prepared according to the general procedure 3, using **21** and *tert*-butyl 4-aminophenethylcarbamate. The mixture was stirred for 18 h at 70 °C to obtain the product (325 mg, 72%) as a yellow solid. 1H NMR (300 MHz, DMSO-d6) δ 13.32 (s, 1H), 11.84 (s, 1H), 10.76 (s, 1H), 8.73 (d, J = 8.2 Hz, 1H), 7.87 (t, J = 7.7 Hz, 1H), 7.61 (d, J = 8.2 Hz, 1H), 7.55–7.41 (m, 3H), 7.23 (d, J = 8.0 Hz, 2H), 7.13 (s, 1H), 6.92 (t, J = 5.4 Hz, 1H), 3.88 (s, 3H), 3.24–3.09 (m, 2H), 2.81–2.67 (m, 2H), 1.37 (s, 9H). 13C NMR (75 MHz, DMSO) δ 159.64, 158.44, 155.56, 151.92, 145.61, 139.54, 136.79, 135.78, 134.22, 129.28, 124.88, 124.78, 122.92, 117.59, 110.25, 102.96, 77.55, 52.06, 41.49, 35.13, 28.25. MS-ESI *m*/*z* [M + H]^+^: calcd 505.6, found 505.2. HRMS *m*/*z* [M + H]^+^: calcd 504.2354, found 504.2348. HPLC: t_R_ = 7.77, purity ≥ 95% (UV: 254/280 nm).

methyl 5-((2-((3-(2-((*tert*-butoxycarbonyl)amino)ethyl)phenyl)amino)quinazolin-4-yl)amino)-1*H*-pyrazole-3-carboxylate (**23b**). The title compound was prepared according to the general procedure 3, using **21** and *tert-*butyl 3-aminophenethylcarbamate. The mixture was stirred for 18 h at 70 °C to obtain the product (305 mg, 61%) as a yellow solid. 1H NMR (300 MHz, DMSO-d6) δ 13.65 (d, J = 246.4 Hz, 1H), 11.82 (s, 1H), 10.70 (s, 1H), 8.72 (d, J = 8.3 Hz, 1H), 7.88 (t, J = 7.8 Hz, 1H), 7.65 (d, J = 8.3 Hz, 1H), 7.50 (t, J = 7.7 Hz, 1H), 7.45–7.29 (m, 3H), 7.13 (d, J = 7.3 Hz, 1H), 7.06 (s, 1H), 6.85 (t, J = 5.9 Hz, 1H), 3.86 (s, 3H), 3.20–3.06 (m, 2H), 2.74–2.64 (m, 2H), 1.34 (s, 9H). 13C NMR (75 MHz, DMSO) δ 159.75, 158.31, 155.56, 152.14, 140.75, 139.90, 136.16, 135.74, 129.18, 126.19, 124.95, 124.70, 123.51, 121.24, 117.69, 110.35, 102.58, 77.60, 51.95, 41.28, 35.38, 28.24. MS-ESI *m*/*z* [M + H]^+^: calcd 505.6, found 505.4. HRMS *m*/*z* [M + H]^+^: calcd 504.2354, found 504.2347. HPLC: t_R_ = 8.11, purity ≥ 95% (UV: 254/280 nm).

methyl 5-((2-((5-((*tert*-butoxycarbonyl)amino)pentyl)amino)quinazolin-4-yl)amino)-1*H*-pyrazole-3-carboxylate (**23c**). The title compound was prepared according to the general procedure 4, using **21** and *tert-*butyl (5-aminopentyl)carbamate. The mixture was stirred for 8 h at 90 °C to obtain the product (233 mg, 50%) as a yellow solid. 1H NMR (250 MHz, DMSO-d6) δ 14.04 (s, 1H), 11.60 (s, 1H), 8.59 (d, J = 8.1 Hz, 1H), 8.10–7.65 (m, 2H), 7.62–7.24 (m, 2H), 6.76 (s, 1H), 3.87 (s, 3H), 3.53–3.42 (m, 2H), 3.03–2.87 (m, 2H), 1.75–1.54 (m, 2H), 1.46–1.25 (m, 13H). 13C NMR (75 MHz, DMSO) δ 156.10, 136.42, 135.13, 125.12, 124.67, 117.61, 110.68, 102.74, 77.82, 52.56, 41.71, 40.17, 29.66, 29.02, 28.73, 24.05. MS-ESI *m*/*z* [M + H]^+^: calcd 470.6, found 470.5. HRMS *m*/*z* [M + H]^+^: calcd 470.2510, found 470.2505. HPLC: t_R_ = 7.91, purity ≥ 95% (UV: 254/280 nm).

methyl 3-((2-((4-(2-aminoethyl)phenyl)amino)pyrimidin-4-yl)amino)-1*H*-pyrazole-5-carboxylate (**24**). **18a** (50 mg, 0.1 mmol) was dissolved in anhydrous DCM (4 mL). TFA (503 mg, 4.4 mmol) was added at 0°C and the reaction mixture was allowed to warm up to rt overnight. The solvent was evaporated under reduced pressure. The residue was dissolved in methanol and neutralized with saturated K_2_CO_3_ solution. The solvent was again evaporated under reduced pressure and the crude product was purified by flash chromatography using H_2_O/acetonitrile as an eluent to obtain the desired product (21 mg, 54%) as a yellow solid. 1H NMR (250 MHz, DMSO-d6) δ 9.27 (s, 1H), 8.03 (d, J = 5.7 Hz, 1H), 7.69–7.53 (m, 2H), 7.19–7.06 (m, 2H), 6.81 (s, 1H), 6.30 (d, J = 5.7 Hz, 1H), 3.84 (s, 3H), 2.84–2.70 (m, 2H), 2.68–2.54 (m, 2H). 13C NMR (126 MHz, DMSO) δ 160.93, 159.55, 159.08, 156.32, 151.48, 145.39, 138.58, 132.90, 128.62, 119.03, 98.07, 96.95, 51.72, 43.72, 39.02. MS-ESI *m*/*z* [M + H]^+^: calcd 354.4, found 354.7. HRMS *m*/*z* [M + Na]^+^: calcd 376.1492, found 376.1485. HPLC: t_R_ = 10.09, purity ≥ 95% (UV: 254/280 nm).

methyl 3-((2-((4-aminobenzyl)amino)pyrimidin-4-yl)amino)-1*H*-pyrazole-5-carboxylate (**25**). **18i** (20 mg, 0.05 mmol) was dissolved in anhydrous DCM (1 mL). TFA (208 mg, 1.8 mmol) was added at 0 °C and the reaction mixture was allowed to warm up to rt overnight. The solvent was evaporated under reduced pressure. The residue was dissolved in methanol and neutralized with saturated K_2_CO_3_ solution. The solvent was again evaporated under reduced pressure and the crude product was purified by flash chromatography using H_2_O/acetonitrile as an eluent to obtain the desired product (5 mg, 36%) as a yellow solid. 1H NMR (250 MHz, DMSO-d6) δ 13.38 (s, 1H), 9.81 (s, 1H), 7.88 (d, J = 5.5 Hz, 1H), 7.26 (s, 1H), 7.01 (d, J = 8.3 Hz, 2H), 6.50 (d, J = 8.3 Hz, 2H), 6.07 (d, J = 4.8 Hz, 1H), 4.89 (s, 2H), 4.30 (d, J = 5.9 Hz, 2H), 3.81 (s, 3H). MS-ESI *m*/*z* [M + H]^+^: calcd 340.4, found 340.6. HRMS *m*/*z* [M + H]^+^: calcd 340.1517, found 340.1529. HPLC: t_R_ = 10.13, purity ≥ 95% (UV: 254/280 nm).

2-chloro-*N*-(3-methyl-1*H*-pyrazol-5-yl)pyrimidin-4-amine (**32**). The title compound was prepared according to the general procedure 1, using 5-methyl-1*H*-pyrazol-3-amine and 2,4-dichloropyrimidine. The mixture was stirred at 60 °C for 72 h to obtain the product (466 mg, 48%) as a beige solid. 1H NMR (300 MHz, DMSO-d6) δ 12.13 (s, 1H), 10.28 (s, 1H), 8.15 (d, J = 5.9 Hz, 1H), 7.15 (s, 1H), 6.09 (s, 1H), 2.22 (s, 3H). 13C NMR (75 MHz, DMSO) δ 160.77, 159.37, 147.35, 142.17, 138.83, 104.97, 95.50, 10.61. MS-ESI *m*/*z* [M + H]^+^: calcd 210.6, found 210.2.

2-chloro-*N*-(3-isopropyl-1*H*-pyrazol-5-yl)pyrimidin-4-amine (**33**). The title compound was prepared according to the general procedure 1, using 5-isopropyl-1*H*-pyrazol-3-amine and 2,4-dichloropyrimidine. The mixture was stirred at 60 °C for 120 h to obtain the product (543 mg, 63%) as a beige solid. 1H NMR (300 MHz, DMSO-d6) δ 12.17 (s, 1H), 10.28 (s, 1H), 8.16 (d, J = 6.0 Hz, 1H), 7.20 (s, 1H), 6.07 (s, 1H), 2.93 (p, J = 6.9 Hz, 1H), 1.21 (d, J = 6.9 Hz, 6H). 13C NMR (75 MHz, DMSO) δ 161.09, 160.80, 159.35, 149.85, 147.06, 104.94, 92.75, 25.29, 22.21. MS-ESI *m*/*z* [M + H]^+^: calcd 238.7, found 238.2. 

*N*-(3-(*tert*-butyl)-1*H*-pyrazol-5-yl)-2-chloropyrimidin-4-amine (**34**). The title compound was prepared according to the general procedure 1, using 5-(*tert*-butyl)-1*H*-pyrazol-3-amine and 2,4-dichloropyrimidine. The mixture was stirred at 60 °C for 72 h to obtain the product (688 mg, 84%) as a beige solid. 1H NMR (300 MHz, DMSO-d6) δ 12.17 (s, 1H), 10.28 (s, 1H), 8.16 (d, J = 5.9 Hz, 1H), 7.24 (s, 1H), 6.04 (s, 1H), 1.26 (s, 9H). 13C NMR (75 MHz, DMSO) δ 160.80, 159.35, 157.60, 152.96, 146.72, 104.86, 92.35, 30.66, 29.89. MS-ESI *m*/*z* [M + H]^+^: calcd 252.7, found 252.2. 

5-((2-chloropyrimidin-4-yl)amino)-*N*-methyl-1*H*-pyrazole-3-carboxamide (**35**). The title compound was prepared according to the general procedure 1, using 3-amino-*N*-methyl-1*H*-pyrazole-5-carboxamide and 2,4-dichloropyrimidine. The mixture was stirred at 60 °C for 48 h to obtain the product (82 mg, 14%) as a white solid. 1H NMR (300 MHz, DMSO-d6) δ 13.18 (s, 1H), 10.49 (s, 1H), 8.52 (d, J = 5.2 Hz, 1H), 8.20 (d, J = 5.9 Hz, 1H), 7.18 (s, 1H), 6.85 (s, 1H), 2.76 (d, J = 4.5 Hz, 3H). 13C NMR (126 MHz, DMSO) δ 160.84, 160.77, 159.46, 158.95, 147.31, 137.29, 105.11, 95.89, 25.55. MS-ESI *m*/*z* [M + H]^+^: calcd 253.7, found 253.2.

isopropyl 3-((2-chloropyrimidin-4-yl)amino)-1*H*-pyrazole-5-carboxylate (**36**). The title compound was prepared according to the general procedure 1, using isopropyl 3-amino-1*H*-pyrazole-5-carboxylate and 2,4-dichloropyrimidine. The mixture was stirred at 60 °C for 48 h to obtain the product (363 mg, 43%) as a beige solid with impurities. MS-ESI *m*/*z* [M + H]^+^: calcd 282.7, found 282.1.

*tert*-butyl 3-((2-chloropyrimidin-4-yl)amino)-1*H*-pyrazole-5-carboxylate (**37**). The title compound was prepared according to the general procedure 1, using *tert*-butyl 3-amino-1*H*-pyrazole-5-carboxylate and 2,4-dichloropyrimidine. The mixture was stirred at 60 °C for 48 h to obtain the product (565 mg, 35%) as a white solid. 1H NMR (400 MHz, DMSO-d6) δ 13.49 (s, 1H), 10.61 (s, 1H), 8.20 (d, J = 5.9 Hz, 1H), 7.27–6.55 (m, 2H), 1.54 (s, 9H). 13C NMR (101 MHz, DMSO) δ 160.59, 159.40, 158.09, 147.59, 142.19, 134.61, 105.37, 99.20, 81.95, 27.79. MS-ESI *m*/*z* [M + H]^+^: calcd 296.7, found 296.1.

*tert*-butyl 4-((4-((3-methyl-1*H*-pyrazol-5-yl)amino)pyrimidin-2-yl)amino)phenethyl-carbamate (**38a**). The title compound was prepared according to the general procedure 2, using **28** and *tert*-butyl 4-aminophenethylcarbamate. The mixture was stirred for 18 h under reflux to obtain the product (66 mg, 67%) as a white solid. 1H NMR (300 MHz, DMSO-d6) δ 12.41 (s, 1H), 11.20 (s, 1H), 10.68 (s, 1H), 7.96 (s, 1H), 7.45 (d, J = 6.5 Hz, 2H), 7.25 (d, J = 8.0 Hz, 2H), 6.91 (t, J = 5.6 Hz, 1H), 6.45 (s, 1H), 6.24 (s, 1H), 3.16 (q, J = 6.9 Hz, 2H), 2.78–2.66 (m, 2H), 2.19 (s, 3H), 1.37 (s, 9H). 13C NMR (75 MHz, DMSO) δ 159.67, 155.54, 152.57, 145.99, 142.74, 139.27, 136.66, 134.69, 129.15, 123.31, 98.95, 97.03, 77.53, 41.57, 34.98, 28.25, 10.63. MS-ESI *m*/*z* [M + H]^+^: calcd 410.5, found 410.4. HRMS *m*/*z* [M + H]^+^: calcd 410.2299, found 410.2297. HPLC: t_R_ = 7.30, purity ≥ 95% (UV: 254/280 nm).

*tert*-butyl (5-((4-((3-methyl-1*H*-pyrazol-5-yl)amino)pyrimidin-2-yl)amino)pentyl)-carbamate (**38b**). The title compound was prepared according to the general procedure 4, using **28** and *tert-*butyl (5-aminopentyl)carbamate. The mixture was stirred for 8 h at 90 °C to obtain the product (31 mg, 29%) as a colorless oil. 1H NMR (300 MHz, DMSO-d6) δ 11.84 (s, 1H), 9.30 (s, 1H), 7.78 (d, J = 5.7 Hz, 1H), 6.75 (t, J = 5.7 Hz, 1H), 6.62 (s, 1H), 6.33–6.07 (m, 2H), 3.21 (q, J = 6.6 Hz, 2H), 2.91 (q, J = 6.5 Hz, 2H), 2.18 (s, 3H), 1.58–1.47 (m, 2H), 1.44–1.37 (m, 2H), 1.36 (s, 9H), 1.32–1.22 (m, 2H). 13C NMR (75 MHz, DMSO) δ 162.09, 159.66, 156.04, 155.60, 148.52, 138.40, 95.58, 95.15, 77.31, 41.30, 39.89, 29.36, 28.97, 28.28, 23.94, 11.07. MS-ESI *m*/*z* [M + H]^+^: calcd 376.5, found 376.4. HRMS *m*/*z* [M + H]^+^: calcd 376.2456, found 376.2458. HPLC: t_R_ = 7.08, purity ≥ 95% (UV: 254/280 nm).

*tert*-butyl 4-((4-((3-isopropyl-1*H*-pyrazol-5-yl)amino)pyrimidin-2-yl)amino)phenethyl-carbamate (**39a**). The title compound was prepared according to the general procedure 2, using **29** and *tert*-butyl 4-aminophenethylcarbamate. The mixture was stirred for 18 h at 70 °C to obtain the product (70 mg, 64%) as a white solid. 1H NMR (300 MHz, DMSO-d6) δ 12.41 (s, 1H), 11.22 (s, 1H), 10.62 (s, 1H), 7.96 (d, J = 5.8 Hz, 1H), 7.43 (d, J = 7.9 Hz, 2H), 7.24 (d, J = 8.2 Hz, 2H), 6.90 (t, J = 5.0 Hz, 1H), 6.45 (s, 1H), 6.22 (s, 1H), 3.21–3.07 (m, 2H), 2.98–2.81 (m, 1H), 2.74–2.66 (m, 2H), 1.37 (s, 9H), 1.15 (d, J = 6.9 Hz, 6H). 13C NMR (75 MHz, DMSO) δ 159.67, 155.52, 152.65, 149.96, 145.70, 142.19, 136.62, 134.50, 129.22, 123.45, 99.10, 94.05, 77.52, 41.45, 35.03, 28.25, 25.35, 22.21. MS-ESI *m*/*z* [M + H]^+^: calcd 438.6, found 438.5. HRMS *m*/*z* [M + H]^+^: calcd 438.2612, found 438.2609. HPLC: t_R_ = 7.92, purity ≥ 95% (UV: 254/280 nm).

*tert*-butyl (5-((4-((3-isopropyl-1*H*-pyrazol-5-yl)amino)pyrimidin-2-yl)amino)pentyl)-carbamate (**39b**). The title compound was prepared according to the general procedure 4, using **29** and *tert-*butyl (5-aminopentyl)carbamate. The mixture was stirred for 8 h at 90 °C to obtain the product (22 mg, 22%) as a yellow solid. 1H NMR (300 MHz, DMSO-d6) δ 11.97 (s, 1H), 9.71 (s, 1H), 7.78 (d, J = 6.0 Hz, 1H), 6.98 (s, 1H), 6.74 (t, J = 5.7 Hz, 1H), 6.45–6.04 (m, 2H), 3.25 (q, J = 6.9 Hz, 2H), 2.96–2.82 (m, 3H), 1.62–1.47 (m, 2H), 1.45–1.26 (m, 13H), 1.21 (d, J = 6.9 Hz, 6H). 13C NMR (75 MHz, DMSO) δ 160.54, 159.70, 155.61, 153.36, 149.65, 147.25, 96.01, 92.67, 77.34, 40.75, 29.32, 28.86, 28.29, 25.61, 23.90, 22.34. MS-ESI *m*/*z* [M + H]^+^: calcd 404.5, found 404.5. HRMS *m*/*z* [M + H]^+^: calcd 404.2769, found 404.2767. HPLC: t_R_ = 7.81, purity ≥ 95% (UV: 254/280 nm).

*tert*-butyl 4-((4-((3-(*tert*-butyl)-1*H*-pyrazol-5-yl)amino)pyrimidin-2-yl)amino)phenethyl-carbamate (**40a).** The title compound was prepared according to the general procedure 3, using **30** and *tert*-butyl 4-aminophenethylcarbamate. The mixture was stirred for 18 h under reflux to obtain the product (70 mg, 79%) as a yellow solid. 1H NMR (300 MHz, DMSO-d6) δ 12.38 (s, 1H), 11.24 (s, 1H), 10.65 (s, 1H), 7.96 (d, J = 4.5 Hz, 1H), 7.42 (d, J = 7.7 Hz, 2H), 7.23 (d, J = 8.0 Hz, 2H), 6.89 (t, J = 5.2 Hz, 1H), 6.45 (s, 1H), 6.21 (s, 1H), 3.22–3.08 (m, 2H), 2.76–2.62 (m, 2H), 1.37 (s, 9H), 1.20 (s, 9H). 13C NMR (75 MHz, DMSO) δ 159.59, 155.51, 152.98, 152.52, 145.46, 142.22, 136.67, 134.40, 129.26, 123.32, 99.15, 93.71, 77.51, 41.39, 35.02, 30.71, 29.87, 28.24. MS-ESI *m*/*z* [M + H]^+^: calcd 452.6, found 452.4. HRMS *m*/*z* [M + H]^+^: calcd 452.2769, found 452.2763. HPLC: t_R_ = 7.78, purity ≥ 95% (UV: 254/280 nm).

*tert*-butyl (5-((4-((3-(tert-butyl)-1*H*-pyrazol-5-yl)amino)pyrimidin-2-yl)amino)pentyl)-carbamate (**40b**). The title compound was prepared according to the general procedure 4, using **30** and *tert-*butyl (5-aminopentyl)carbamate. The mixture was stirred for 8 h at 90 °C to obtain the product (24 mg, 24%) as a colorless oil. 1H NMR (300 MHz, DMSO-d6) δ 11.86 (s, 1H), 9.27 (s, 1H), 7.77 (d, J = 5.7 Hz, 1H), 6.73 (t, J = 5.3 Hz, 1H), 6.59 (s, 1H), 6.38 (s, 1H), 6.14 (s, 1H), 3.23 (q, J = 6.7 Hz, 2H), 2.90 (q, J = 6.5 Hz, 2H), 1.58–1.46 (m, 2H), 1.44–1.22 (m, 22H). 13C NMR (75 MHz, DMSO) δ 162.10, 159.74, 156.02, 155.57, 151.97, 148.06, 95.55, 92.47, 77.29, 40.69, 30.73, 30.05, 29.32, 29.03, 28.27, 23.92. MS-ESI *m*/*z* [M + H]^+^: calcd 418.6, found 418.5. HRMS *m*/*z* [M + H]^+^: calcd 418.2925, found 418.2924. HPLC: t_R_ = 7.65, purity ≥ 95% (UV: 254/280 nm).

2-(4-((4-((3-(*tert*-butyl)-1*H*-pyrazol-5-yl)amino)pyrimidin-2-yl)amino)phenyl)acetonitrile (**40c**). The title compound was prepared according to the general procedure 3, using **30** and 2-(4-aminophenyl)acetonitrile. The mixture was stirred for 18 h under reflux to obtain the product (31 mg, 41%) as a white solid. 1H NMR (300 MHz, DMSO-d6) δ 12.43 (s, 1H), 11.26 (s, 1H), 10.81 (s, 1H), 7.99 (d, J = 6.9 Hz, 1H), 7.56 (d, J = 8.0 Hz, 2H), 7.39 (d, J = 8.3 Hz, 2H), 6.49 (s, 1H), 6.19 (s, 1H), 4.04 (s, 2H), 1.21 (s, 9H). 13C NMR (75 MHz, DMSO) δ 159.77, 153.14, 152.57, 145.39, 142.44, 136.06, 128.82, 127.96, 123.51, 119.02, 99.32, 93.71, 30.73, 29.90, 21.91. MS-ESI *m*/*z* [M + H]^+^: calcd 348.4, found 348.3. HRMS *m*/*z* [M + H]^+^: calcd 348.1931, found 348.1933. HPLC: t_R_ = 7.27, purity ≥ 95% (UV: 254/280 nm).

*tert*-butyl 4-((4-((3-(methylcarbamoyl)-1*H*-pyrazol-5-yl)amino)pyrimidin-2-yl)amino)-phenethylcarbamate (**41a**). The title compound was prepared according to the general procedure 3, using **31** and *tert*-butyl 4-aminophenethylcarbamate. The mixture was stirred for 24 h at 70 °C to obtain the product (20 mg, 40%) as a yellow solid. 1H NMR (300 MHz, DMSO-d6) δ 13.08 (s, 1H), 10.12 (d, J = 126.5 Hz, 1H), 9.40 (d, J = 98.9 Hz, 1H), 8.35 (s, 1H), 8.04 (s, 1H), 7.64 (d, J = 8.0 Hz, 2H), 7.10 (d, J = 8.1 Hz, 2H), 6.98–6.77 (m, 1H), 6.24 (s, 1H), 3.19–3.07 (m, 2H), 2.78 (d, J = 4.6 Hz, 3H), 2.68–2.59 (m, 2H), 1.37 (s, 9H). 13C NMR (75 MHz, DMSO) δ 164.36, 159.06, 155.55, 151.53, 142.19, 138.52, 132.28, 129.03, 128.71, 119.31, 114.78, 100.24, 98.02, 77.50, 41.76, 34.97, 28.28, 25.60. MS-ESI *m*/*z* [M + H]^+^: calcd 453.5, found 454.0. HRMS *m*/*z* [M + Na]^+^: calcd 475.2177, found 475.2173. HPLC: t_R_ = 7.04, purity ≥ 95% (UV: 254/280 nm).

*tert*-butyl (5-((4-((3-(methylcarbamoyl)-1*H*-pyrazol-5-yl)amino)pyrimidin-2-yl)amino)-pentyl)carbamate (**41b**). The title compound was prepared according to the general procedure 4, using **31** and *tert-*butyl (5-aminopentyl)carbamate. The mixture was stirred for 8 h at 90 °C to obtain the product (17 mg, 34%) as a white solid. 1H NMR (300 MHz, DMSO-d6) δ 12.95 (d, J = 48.6 Hz, 1H), 9.74 (d, J = 191.6 Hz, 1H), 8.36–7.75 (m, 2H), 7.33–6.86 (m, 1H), 6.75 (t, J = 4.9 Hz, 1H), 6.60–5.86 (m, 2H), 3.24 (q, J = 6.6 Hz, 2H), 2.90 (q, J = 6.5 Hz, 2H), 2.76 (d, J = 4.6 Hz, 3H), 1.59–1.45 (m, 2H), 1.45–1.21 (m, 13H). 13C NMR (75 MHz, DMSO) δ 164.36, 162.01, 159.74, 156.72, 155.62, 151.55, 142.21, 100.24, 95.70, 77.33, 40.58, 29.30, 28.93, 28.28, 25.59, 23.89. MS-ESI *m*/*z* [M + H]^+^: calcd 419.5, found 419.8. HRMS *m*/*z* [M + H]^+^: calcd 419.2514, found 419.2524. HPLC: t_R_ = 6.83, purity ≥ 95% (UV: 254/280 nm).

5-((2-((4-(cyanomethyl)phenyl)amino)pyrimidin-4-yl)amino)-N-methyl-1*H*-pyrazole-3-carboxamide (**41c**). The title compound was prepared according to the general procedure 2, using **31** and 2-(4-aminophenyl)acetonitrile. The mixture was stirred for 18 h under reflux to obtain the product (15 mg, 45%) as a yellow solid. 1H NMR (300 MHz, DMSO-d6) δ 11.28 (s, 1H), 10.90 (s, 1H), 8.56 (s, 1H), 8.14–7.92 (m, 1H), 7.61 (d, J = 8.1 Hz, 2H), 7.38 (d, J = 8.1 Hz, 2H), 7.19 (s, 1H), 6.57 (s, 1H), 4.04 (s, 2H), 2.79 (d, J = 4.5 Hz, 3H). 13C NMR (75 MHz, DMSO) δ 160.50, 159.22, 152.53, 145.57, 144.26, 138.51, 136.11, 128.96, 127.72, 122.60, 119.27, 99.11, 98.18, 25.60, 21.97. MS-ESI *m*/*z* [M + H]^+^: calcd 349.4, found 349.6. HRMS *m*/*z* [M + H]^+^: calcd 349.1520, found 349.1525. HPLC: t_R_ = 6.23, purity ≥ 95% (UV: 254/280 nm).

isopropyl 3-((2-((5-((*tert*-butoxycarbonyl)amino)pentyl)amino)pyrimidin-4-yl)amino)-1*H*-pyrazole-5-carboxylate (**42b**). The title compound was prepared according to the general procedure 4, using **32** and *tert-*butyl (5-aminopentyl)carbamate. The mixture was stirred for 6 h at 100 °C to obtain the product (8 mg, 4%) as a yellow solid. 1H NMR (400 MHz, DMSO-d6) δ 13.32 (s, 1H), 9.89 (d, J = 172.8 Hz, 1H), 7.83 (s, 1H), 7.15 (s, 1H), 6.74 (s, 2H), 6.10 (s, 1H), 5.11 (q, J = 12.9, 6.6 Hz, 1H), 3.24 (q, J = 6.6 Hz, 2H), 2.90 (q, J = 6.6 Hz, 2H), 1.58–1.47 (m, 2H), 1.44–1.28 (m, 19H). 13C NMR (101 MHz, DMSO) δ 156.59, 155.58, 96.06, 91.63, 77.29, 68.23, 29.29, 28.88, 28.26, 23.86, 21.66, 13.39, 12.99. MS-ESI *m*/*z* [M + H]^+^: calcd 448.5, found 448.5. HRMS *m*/*z* [M + H]^+^: calcd 448.2673, found 448.2667. HPLC: t_R_ = 6.16, purity ≥ 95% (UV: 254/280 nm).

isopropyl 3-((2-((4-((*tert*-butoxycarbonyl)amino)benzyl)amino)pyrimidin-4-yl)amino)-1*H*-pyrazole-5-carboxylate (**42d**). The title compound was prepared according to the general procedure 4, using **32** and *tert-*butyl (4-(aminomethyl)phenyl)carbamate. The mixture was stirred for 6 h at 100 °C to obtain the product (8 mg, 4%) as a beige solid. 1H NMR (400 MHz, DMSO-d6) δ 13.31 (s, 1H), 9.91 (d, J = 194.0 Hz, 1H), 9.23 (s, 1H), 7.83 (s, 1H), 7.65–6.93 (m, 6H), 6.12 (s, 1H), 5.11 (s, 1H), 4.42 (d, J = 6.2 Hz, 2H), 1.45 (s, 9H), 1.30 (d, J = 6.3 Hz, 6H). 13C NMR (101 MHz, DMSO) δ 162.00, 159.59, 158.67, 156.43, 152.78, 148.97, 137.95, 134.39, 127.73, 127.25, 117.99, 99.18, 96.05, 78.84, 68.27, 43.59, 28.13, 21.65. MS-ESI *m*/*z* [M + H]^+^: calcd 468.5, found 468.5. HRMS *m*/*z* [M + H]^+^: calcd 468.2351, found 468.2354. HPLC: t_R_ = 6.24, purity ≥ 95% (UV: 254/280 nm).

*tert*-butyl 3-((2-((5-((*tert*-butoxycarbonyl)amino)pentyl)amino)pyrimidin-4-yl)amino)-1*H*-pyrazole-5-carboxylate (**43b**). The title compound was prepared according to the general procedure 4, using **40** and *tert-*butyl (5-aminopentyl)carbamate. The mixture was stirred for 6 h at 100 °C to obtain the product (14 mg, 5%) as a yellow oil. 1H NMR (400 MHz, DMSO-d6) δ 13.25 (d, J = 59.3 Hz, 1H), 9.86 (d, J = 176.8 Hz, 1H), 7.81 (s, 1H), 7.10 (s, 1H), 6.73 (s, 2H), 6.10 (s, 1H), 3.24 (q, J = 6.6 Hz, 2H), 2.90 (q, J = 6.6 Hz, 2H), 1.56–1.47 (m, 11H), 1.42–1.26 (m, 13H). 13C NMR (101 MHz, DMSO) δ 162.18, 159.53, 158.61, 156.31, 155.57, 149.02, 134.08, 99.07, 95.80, 81.44, 77.29, 40.76, 29.31, 28.88, 28.27, 28.13, 27.85, 23.88. MS-ESI *m*/*z* [M + H]^+^: calcd 462.6, found 462.5. HRMS *m*/*z* [M + H]^+^: calcd 462.2819, found 462.2823. HPLC: t_R_ = 6.36, purity ≥ 95% (UV: 254/280 nm).

*tert*-butyl 3-((2-((4-((*tert*-butoxycarbonyl)amino)benzyl)amino)pyrimidin-4-yl)amino)-1*H*-pyrazole-5-carboxylate (**43d**). The title compound was prepared according to the general procedure 4, using **40** and *tert-*butyl (4-(aminomethyl)phenyl)carbamate. The mixture was stirred for 6 h at 100 °C to obtain the product (26 mg, 12%) as a white solid. 1H NMR (400 MHz, DMSO-d6) δ 13.26 (d, J = 60.0 Hz, 1H), 10.26–9.51 (m, 1H), 9.39–9.05 (m, 1H), 7.83 (s, 1H), 7.65–7.02 (m, 6H), 6.13 (s, 1H), 4.42 (d, J = 6.2 Hz, 2H), 1.52 (s, 9H), 1.45 (s, 9H). 13C NMR (101 MHz, DMSO) δ 162.05, 159.57, 158.46, 156.39, 152.79, 148.89, 137.99, 134.44, 127.79, 127.38, 117.99, 99.26, 96.07, 81.51, 78.85, 43.63, 28.14, 27.85. MS-ESI *m*/*z* [M + H]^+^: calcd 482.6, found 482.5. HRMS *m*/*z* [M + H]^+^: calcd 482.2507, found 482.2510. HPLC: t_R_ = 6.58, purity ≥ 95% (UV: 254/280 nm).

## Data Availability

Data is contained within the article or Supplementary Material.

## Supplementary Materials

The following supporting information can be downloaded at: https://www.mdpi.com/article/10.3390/ijms232314834/s1.

## Abbreviations

CDK: cyclin-dependent kinase, DCM, dichloromethane; DSF, differential scanning fluorimetry; EE, ethyl acetate; FUCCI, florescent ubiquitination-base cell cycle indicator; HATU, hexafluorophosphate azabenzotriazole tetramethyl uronium; n.d., not determined; NSCLC, non-small-cell lung carcinoma; oN, overnight; rt, room temperature; SCC, squamous cell carcinoma; TEA, triethylamine; TFA, trifluoroacetic acid.
