# Supplementary material for: Discovery of 3-Amino-1H-pyrazole-Based Kinase Inhibitors to Illuminate the Understudied PCTAIRE Family
Source: Int J Mol Sci. 2022 Nov 27;23(23):14834. doi: 10.3390/ijms232314834 (PMC9736855; doi:10.3390/ijms232314834)
Supplement: Supplementary file 1 [file ijms-23-14834-s001.zip › ijms-2056908-supplementary.pdf]

Supporting Information

Discovery of 3-amino-1*H*-pyrazole-based kinase inhibitors to illuminate the understudied PCTAIRE family

Jennifer Alisa Amrhein[1,2], Lena Marie Berger[1,2], Amelie Tjaden[1,2], Andreas Krämer[1,2,3], Lewis Elson[1,2], Tuomas Tolvanen[4], Daniel Martinez-Molina[5], Astrid Kaiser[1], Manfred Schubert-Zsilavecz[1], Susanne Müller[1,2], Stefan Knapp[1,2,3*], Thomas Hanke[1,2*]

[1]Institute of Pharmaceutical Chemistry, Goethe-University Frankfurt, Biozentrum, Max-von-Laue-Str. 9, 60438 Frankfurt am Main, Germany

[2]Structural Genomics Consortium, Goethe-University Frankfurt, Buchmann Institute for Life Sciences, Max-von-Laue-Str. 15, 60438 Frankfurt am Main, Germany

[3]German Cancer Consortium (DKTK), German Cancer Research Center (DKFZ), DKTK site Frankfurt-Mainz, 69120 Heidelberg, Germany

[4]Division of Rheumatology, Department of Medicine Solna, Karolinska University Hospital and Karolinska Institute, Solnavägen 1, 17177 Solna, Sweden

[5]Pelago Bioscience AB, Scheeles väg 1, 17165 Solna, Sweden

*correspondence:

Thomas Hanke: hanke@pharmchem.uni-frankfurt.de

or Stefan Knapp: knapp@pharmchem.uni-frankfurt.de

Table of Content:

Supplementary Table S1 – S7 S2

Supplementary Figure S1 – S2 S19

Analytical data of compounds **8 – 15, 18 – 25, 32 – 43** S20

Table S1: Values of the DSF results of the first series of 3-aminopyrazole based molecules. They were screened against an *in-house* panel of 104 kinases. Staurosporine was used as a reference.

	ΔT$_m$ [°C]														
	staurosporine	1	11a	11b	11c	11d	11e	11f	12a	12b	12c	12d	12e	13a	13c	15
R$_1$=		H	H	H	H	H	H	H	Me	Me	Me	Me	Me	Cl	Cl	H
CDK16	9.1	10.3	8.7	6.3	9.4	7.3	8.9	7.6	6.2	3.7	6.5	5.8	6.3	3.2	3.2	8.8
AAK1	15.6	13.8	14.3	8.5	13.0	9.3	11.8	9.1	12.6	8.9	11.4	9.7	10.6	11.2	13.1	10.3
ABL1	10.3	8.7	6.9	4.9	4.7	2.5	4.3	5.0	5.9	4.7	4.0	3.5	3.4	1.9	4.2	4.6
AKT3	6.6	1.4	-0.2	0.2	0.0	-0.1	0.1	-0.2	-0.2	-0.2	-0.1	0.0	-0.3	-0.6	0.0	0.2
AURKB	8.3	n.d.	0.8	1.0	1.0	0.2	1.2	0.5	0.2	0.0	0.6	0.3	0.3	-0.7	0.8	1.3
BMP2K	19.1	18.8	18.5	12.3	16.8	13.0	16.2	13.3	16.9	12.6	15.2	13.7	14.6	15.8	17.0	15.5
BMPR2	2.6	9.2	4.8	3.0	3.1	2.1	3.5	2.9	3.3	4.5	3.9	3.8	4.2	6.3	4.7	4.3
BMX	7.1	4.4	2.8	2.1	1.7	0.8	2.3	2.3	1.0	2.6	1.7	1.1	1.8	-3.5	0.0	2.0
BRAF	0.8	4.6	2.2	1.1	1.3	0.5	1.6	1.1	1.3	0.8	0.9	0.7	1.1	0.6	0.8	1.2
BRD4	1.1	1.3	2.1	0.5	0.7	1.0	0.6	0.2	3.5	2.9	0.6	0.8	1.2	1.5	1.8	1.2
BRPF1B	-0.1	n.d.	-0.2	0.4	0.2	0.5	0.7	-0.5	-1.2	-1.2	-0.4	-0.8	-0.1	-1.0	-1.0	-0.2
CAMK1D	10.0	5.4	1.6	0.1	0.3	-1.0	-0.6	-0.7	0.4	0.0	0.2	-0.7	-0.1	4.8	5.0	-0.2
CAMK1G	10.9	5.5	1.3	0.1	0.5	0.5	0.3	-0.5	0.6	0.1	0.4	-0.4	0.5	3.0	3.3	0.1
CAMK2B	13.3	4.4	1.5	0.5	0.9	0.1	0.9	0.7	1.4	0.9	1.2	1.1	1.5	1.1	3.2	1.3
CAMK2D	16.2	4.5	1.9	0.7	1.0	0.3	1.0	0.7	2.0	0.6	1.5	1.5	1.4	2.0	3.5	1.3
CAMK4	8.6	5.4	2.8	1.6	1.5	0.8	1.5	1.4	4.4	2.6	2.7	3.4	2.6	3.6	4.9	2.0
CAMKK2	24.6	12.0	5.8	5.0	4.9	3.1	4.1	4.2	8.3	6.8	6.5	5.7	6.2	7.4	8.8	5.6
CASK	4.8	2.0	1.3	0.2	0.5	-0.1	0.3	0.4	1.3	0.6	0.4	0.1	0.3	0.8	0.6	0.8
CDC42BPA	2.8	-0.1	-0.7	0.3	0.3	0.0	-0.4	-1.0	-1.1	-0.9	-0.2	-0.6	-0.6	0.5	0.2	-0.5
CDK2	15.5	15.2	12.3	8.2	10.4	6.3	11.2	8.4	8.7	4.7	7.2	5.3	8.1	5.9	5.1	11.3
CDKL1	3.1	6.8	2.3	1.8	2.0	1.5	1.2	0.9	-0.1	0.8	0.9	0.9	0.5	-0.4	0.6	2.5
CHEK2	17.1	8.6	5.9	2.2	2.3	1.5	3.7	3.1	6.9	3.6	3.6	3.7	5.0	3.8	4.3	2.5
CLK1	11.9	12.2	7.0	5.4	5.5	4.5	5.8	4.8	7.9	5.2	6.3	5.6	6.5	6.7	5.8	7.8
CLK3	5.6	11.3	2.4	1.7	1.6	1.0	2.2	1.5	2.3	1.4	1.7	1.8	2.2	2.1	1.4	3.6
CSNK1D	1.8	3.1	0.8	0.9	0.2	0.2	0.4	0.2	0.7	0.8	0.7	1.0	0.6	3.6	3.1	0.4
CSNK2A1	2.1	n.d.	2.5	2.0	1.0	0.7	1.1	1.4	3.5	2.3	1.3	1.6	1.2	0.7	2.5	1.8
CK2A2	5.6	8.5	4.3	4.1	3.1	2.2	2.5	2.8	3.8	3.8	2.3	2.8	2.1	5.6	4.3	4.5
DAPK1	9.8	4.9	1.9	1.2	1.5	0.5	1.8	1.7	3.0	2.1	2.3	2.5	2.7	1.9	3.8	2.1
DAPK3	15.8	9.7	4.6	3.0	4.5	3.2	4.8	3.6	4.7	3.3	4.7	4.9	5.1	6.4	8.0	5.2
DCAMKL1	12.4	8.9	4.7	4.8	6.9	4.2	6.8	3.9	5.3	4.1	6.4	5.0	6.4	4.2	7.6	5.4
DMPK1	8.7	3.2	3.7	2.6	2.8	1.7	2.4	1.5	2.9	1.8	2.5	1.6	1.7	1.5	2.0	2.1
DYRK1A	8.7	8.2	n.d.	1.9	0.5	1.4	0.5	-0.6	2.7	0.0	-0.3	-0.4	-0.4	3.9	2.3	2.7
DYRK2	7.0	11.0	3.3	2.5	3.2	2.3	3.6	3.6	1.4	1.5	1.6	2.1	1.8	4.2	5.0	6.1
EPHA2	8.3	8.4	3.9	3.3	1.7	0.8	1.8	2.1	2.6	3.7	1.7	1.6	1.8	0.4	2.0	2.8
EPHA4	6.2	n.d.	2.4	2.4	0.8	0.4	1.0	1.0	2.1	2.8	1.3	1.1	1.2	1.7	2.7	1.3
EPHA5	7.9	6.3	3.7	3.2	1.1	1.0	1.9	0.5	2.2	3.0	1.7	0.9	1.7	1.4	2.4	2.1
EPHA7	10.3	4.9	3.3	3.0	2.1	2.2	2.0	1.0	2.9	1.6	1.9	0.9	2.4	1.4	3.2	1.6
EPHB1	6.4	n.d.	3.1	1.9	0.8	0.2	1.2	1.2	2.5	2.9	1.2	1.3	1.5	1.6	2.0	1.7
EPHB3	5.0	5.1	n.d.	n.d.	0.5	0.0	0.8	0.8	1.7	n.d.	0.9	1.0	1.0	0.3	1.4	1.1
FES	6.2	4.5	1.4	1.5	0.7	0.7	0.8	0.9	1.8	1.7	1.2	1.2	1.4	1.0	2.6	1.8
FGFR1	5.9	4.5	3.2	3.0	1.8	1.5	2.7	2.2	3.0	2.8	2.0	1.9	3.0	2.9	2.7	2.8
FGFR2	9.0	5.9	4.0	3.8	2.7	2.0	3.5	3.4	4.5	4.3	3.4	3.2	4.1	3.9	4.0	3.3
FGFR3	13.3	9.8	6.4	5.7	4.1	2.7	5.7	5.2	6.1	5.8	5.1	4.6	6.1	4.3	6.0	5.0
FLT1	13.6	10.6	7.0	6.8	4.8	3.7	5.4	5.9	6.4	5.9	4.9	5.2	6.0	5.6	5.5	4.7
GAK	9.0	8.0	6.0	4.6	4.7	2.8	4.6	1.8	5.7	4.7	4.3	3.5	4.1	3.8	4.8	3.1
GPRK5	7.7	5.2	2.6	0.7	0.8	0.6	0.7	0.7	3.8	1.1	1.1	0.8	1.1	3.4	1.5	1.0
GSG2	7.2	2.9	-0.1	0.8	0.2	0.3	0.2	-0.2	-0.3	0.1	0.0	0.2	0.0	-0.2	1.2	0.7
GSK3B	11.8	14.0	8.2	7.2	7.1	5.9	6.8	6.3	5.7	5.1	6.1	6.6	5.8	4.6	5.9	7.9
HIPK2	4.4	n.d.	3.0	1.9	1.6	0.8	2.1	2.2	2.2	1.2	1.1	1.2	1.5	1.9	1.9	2.7
MAP2K1	1.3	1.4	0.9	0.5	0.6	0.4	0.6	0.6	1.2	0.7	0.6	0.6	0.7	n.d.	n.d.	0.6
MAP2K4	12.0	10.0	8.7	5.1	4.9	3.6	6.8	5.3	4.9	3.3	3.1	2.0	5.3	3.8	3.7	4.4
MAP2K6	12.3	10.4	8.0	5.6	7.3	5.6	6.8	6.7	4.4	2.5	3.1	2.6	3.0	3.3	3.6	6.2
MAP2K7	8.4	12.0	6.0	1.5	2.4	1.5	3.7	2.0	3.0	0.5	0.9	0.7	2.1	1.5	0.9	2.0
MAP3K5	18.5	n.d.	3.7	2.0	2.2	1.2	3.2	2.4	2.4	1.5	2.3	3.1	3.0	2.4	5.1	2.8
MAPK10	7.4	7.2	3.0	3.6	1.0	1.0	3.0	0.8	3.4	3.6	0.4	1.7	1.3	4.9	7.0	3.2
MAPK13	7.5	8.0	5.4	3.9	3.9	1.7	4.8	4.2	2.3	2.0	2.0	1.7	2.6	1.3	1.4	4.8
MAPK14	0.3	2.5	1.3	0.7	1.1	0.9	1.2	0.8	0.2	0.2	0.6	0.4	0.6	-0.7	-0.6	1.1
MAPK15	14.7	17.4	13.4	9.3	12.3	9.9	12.3	11.0	8.0	4.5	8.5	7.6	8.2	6.8	5.6	13.4
MAPK1	1.4	8.5	4.6	2.2	3.7	2.2	3.6	4.6	4.0	1.5	2.5	2.2	2.9	2.3	2.2	3.1
MAPK8	7.8	n.d.	6.8	5.7	5.1	3.8	5.4	4.4	5.5	5.1	4.1	3.6	4.6	6.5	5.6	5.5
MAPK9	3.7	6.5	4.4	2.9	3.1	2.1	3.3	3.2	4.3	3.5	2.4	2.3	2.6	6.1	5.3	3.6
MAPKAPK2	3.2	n.d.	-0.5	0.5	0.2	0.3	-0.3	-0.5	-0.3	-0.1	-0.4	-0.2	-0.1	-0.1	-0.2	-0.3
MARK3	19.0	8.1	7.0	5.4	7.1	5.0	5.7	3.9	6.0	4.3	6.3	3.5	6.0	6.7	6.5	3.8
MARK4	16.4	8.2	0.0	2.0	0.8	1.7	1.7	1.1	6.8	5.1	0.5	2.1	1.1	6.7	6.4	0.0
MELK	13.8	9.8	6.6	4.5	5.0	3.5	5.2	5.4	7.0	6.0	6.7	5.8	7.4	5.9	7.7	6.5
MERTK	6.6	4.4	3.2	1.9	2.0	1.4	2.5	1.1	3.5	2.6	2.7	2.7	2.8	2.0	2.9	2.6
MST3	7.3	9.3	4.3	1.8	3.0	1.4	2.9	1.5	2.9	1.6	4.2	2.2	5.0	3.2	1.9	3.9
MST4	6.1	7.1	4.3	2.1	2.6	1.6	2.9	1.9	3.2	1.6	3.7	2.2	4.1	3.1	2.5	3.7
NEK1	-0.6	1.1	0.2	0.5	0.2	0.2	-0.1	0.3	0.9	1.6	0.3	0.4	0.3	1.2	0.5	0.4
NEK2	3.3	n.d.	3.1	2.3	1.7	1.4	2.5	0.1	2.8	1.1	2.1	0.6	3.4	2.2	3.4	1.2
NEK7	1.0	n.d.	0.1	1.3	0.0	0.9	0.2	-0.3	0.1	0.6	0.0	-0.1	0.1	-0.6	-0.1	0.2
OSR1	7.7	5.6	5.9	3.5	5.2	3.8	4.4	2.2	4.9	3.0	4.6	4.0	3.4	4.5	5.4	2.9
PAK1	6.6	3.3	1.3	0.9	0.9	0.8	1.0	0.9	0.2	0.2	0.2	0.1	0.7	-0.3	-0.2	1.1
PAK4	12.2	7.7	5.7	3.5	4.6	3.3	4.0	3.0	3.8	4.1	2.3	2.2	2.2	2.6	2.9	3.7
PDK4	0.2	1.2	-0.8	-1.0	-0.4	-0.2	-0.8	-0.7	-0.8	-1.0	-0.4	-0.3	-0.5	-0.3	-0.9	-0.1
PHKG2	21.2	8.8	3.8	3.3	3.8	3.3	3.3	2.3	4.8	4.7	4.5	4.1	4.2	3.2	5.6	4.6
PIM1	12.5	3.3	0.2	0.2	0.0	-0.2	0.4	0.4	-0.7	-0.5	0.0	0.3	-0.4	0.2	0.0	2.2
PIM3	19.7	3.8	1.3	0.4	0.7	0.4	0.7	-0.1	0.1	0.5	0.3	0.2	0.3	1.6	1.8	1.7
PKMYT1	0.2	1.0	-0.7	-0.3	-0.4	0.4	-0.1	-0.5	-1.1	-1.4	-0.3	-1.1	-0.5	0.0	-0.2	0.1
PLK4	16.0	11.1	10.7	8.1	8.7	6.3	8.9	7.6	12.1	10.0	10.1	9.2	10.3	11.4	12.2	8.3
RIOK1	0.0	4.9	4.5	0.6	1.8	2.7	0.6	0.2	-1.1	-1.5	-0.2	-0.6	1.1	2.0	5.7	0.4
RIOK2	1.8	n.d.	n.d.	n.d.	n.d.	n.d.	n.d.	n.d.	n.d.	n.d.	n.d.	n.d.	n.d.	3.1	5.1	n.d.
RPS6KA1	3.9	5.1	0.9	0.3	0.4	0.2	0.3	0.2	0.9	0.5	0.3	0.3	0.2	0.0	0.4	0.8
RPS6KA5	13.7	n.d.	n.d.	n.d.	n.d.	n.d.	n.d.	n.d.	n.d.	n.d.	n.d.	n.d.	n.d.	4.8	7.5	n.d.
RPS6KA6	-0.5	0.4	4.7	6.3	7.4	5.0	6.5	3.0	2.4	5.0	7.4	5.7	7.5	0.0	-0.2	9.1
SLK	18.0	8.8	3.9	3.3	1.8	0.8	1.8	2.1	4.0	3.6	2.0	1.1	1.7	n.d.	n.d.	2.3
SPRK1	6.8	n.d.	0.8	1.0	0.7	0.2	0.5	0.5	1.3	2.0	1.3	1.3	0.7	1.4	2.3	0.7
SRC	5.9	4.4	3.4	2.2	2.0	1.0	2.7	1.9	2.7	3.2	2.5	2.2	2.9	2.4	2.4	3.2
STK10	23.3	10.1	4.9	3.9	3.0	1.6	3.7	2.7	5.4	4.1	3.7	2.0	4.4	4.7	4.4	4.1
STK17A	11.8	9.6	3.5	1.2	2.5	0.7	1.8	0.8	3.7	1.3	2.3	1.7	1.4	5.3	6.5	2.8
STK17B	11.5	9.7	4.0	n.d.	2.0	0.3	1.6	2.3	1.3	3.4	0.0	1.3	1.2	2.6	3.0	2.3
STK38L	12.6	8.7	12.1	7.7	10.3	6.1	10.7	8.4	8.2	4.7	7.1	5.0	8.0	4.4	5.1	11.1
STK39	9.6	7.6	7.4	4.7	6.6	5.6	5.7	3.0	5.7	3.9	6.2	4.9	5.7	4.3	5.6	4.5
STK3	16.6	11.6	8.4	5.7	7.2	4.5	7.2	8.6	8.9	7.2	8.0	7.2	7.9	6.5	7.7	9.8
STK4	14.9	9.6	6.7	4.9	6.1	4.2	6.7	6.0	6.6	5.2	6.3	5.8	6.4	7.1	7.3	8.1
STK6	17.1	14.3	15.2	12.8	13.3	11.0	13.3	13.8	12.7	13.3	13.2	12.1	13.3	13.5	14.2	13.6
TAF1	0.2	0.1	-2.0	-0.8	-1.2	-1.4	-1.8	-1.0	-0.1	0.3	0.3	-0.6	-0.4	-0.6	-0.4	0.0
TIF1	0.8	n.d.	1.4	1.1	1.0	1.1	0.4	-0.1	-0.1	0.1	0.5	0.2	0.6	0.2	-0.1	0.5
TLK1	9.4	n.d.	5.5	2.6	4.8	3.1	4.3	2.8	4.7	2.2	4.0	3.2	4.0	4.0	4.0	4.7
TTK	11.2	8.8	6.2	5.8	5.2	4.1	5.3	5.2	5.8	5.1	4.4	4.1	3.9	-0.3	6.4	5.2
ULK1	11.3	15.5	5.8	3.3	5.1	3.2	5.1	3.6	5.7	4.7	5.9	3.7	7.6	4.4	6.2	5.0
UKL3	19.6	8.3	12.1	7.4	10.9	8.4	10.3	8.3	12.3	8.8	10.9	10.2	11.1	10.3	11.4	9.9
VRK1	2.8	5.9	2.6	0.9	2.2	1.1	2.0	2.4	0.1	0.1	0.2	0.1	0.1	0.6	0.7	2.7
WNK1	0.9	1.1	0.2	1.5	0.3	0.5	0.4	0.1	0.6	0.9	0.1	0.1	0.6	0.4	0.7	0.4

Table S2: Values of the DSF results of the second series of 3-aminopyrazole based molecules. They were screened against an *in-house* panel of 104 kinases. Staurosporine was used as a reference.

	ΔT_m [°C]																			
	staurosporine	21a	21b	21c	21d	21e	21f	21g	21h	21i	22a	22b	22c	22d	22e	23a	23b	23c	24	25
R_1=		H	H	H	H	H	H	H	H	H	Cl	Cl	Cl	Cl	Cl	Quin	Quin	Quin	H	H
CDK16	9.1	4.3	4.9	8.5	4.3	8.5	5.3	5.3	5.1	6.5	0.3	0.8	1.0	0.2	0.2	-0.5	0.2	3.4	2.4	5.6
AAK1	15.6	6.7	5.8	8.2	4.1	7.8	7.1	3.7	7.2	2.0	4.4	5.1	6.8	2.2	4.0	5.8	3.0	9.0	6.4	4.0
ABL1	10.3	2.0	3.4	1.9	1.2	2.3	4.1	1.0	2.2	0.7	1.7	2.5	1.4	0.3	0.7	1.4	1.2	1.8	2.8	2.1
AKT3	6.6	-0.1	0.4	0.0	0.3	0.2	0.8	-0.2	0.0	-0.3	-0.4	-0.1	-0.1	-0.3	-0.5	-0.3	-0.1	-0.2	0.0	0.3
AURKB	8.3	n.d.	n.d.	n.d.	n.d.	n.d.	-0.6	n.d.	n.d.	n.d.	-1.2	-0.7	-0.6	-1.9	-0.6	-1.5	-0.9	-0.8	n.d.	n.d.
BMP2K	19.1	10.2	9.1	12.3	5.7	12.6	11.8	6.2	10.7	2.8	9.1	11.2	10.3	4.2	7.9	7.4	4.8	10.9	10.0	7.5
BMPR2	2.6	2.3	2.1	1.8	0.7	2.0	5.8	0.8	1.2	1.1	1.7	2.7	1.5	0.1	0.4	0.0	0.1	1.1	1.3	2.2
BMX	7.1	0.7	1.3	0.4	0.2	0.9	2.1	0.2	0.8	-0.1	-0.1	0.7	0.7	0.0	0.3	-1.8	-0.3	1.2	1.0	1.3
BRAF	0.8	1.3	1.8	1.6	0.9	1.5	1.8	0.9	0.8	1.0	1.1	1.2	1.0	0.7	0.6	0.2	0.6	0.8	0.9	1.1
BRD4	1.1	0.5	3.3	-0.4	0.7	2.3	1.2	0.8	1.6	1.0	4.1	6.5	1.9	0.8	2.3	1.7	3.6	1.4	0.5	-0.6
BRPF1B	-0.1	n.d.	n.d.	n.d.	n.d.	n.d.	0.8	n.d.	n.d.	n.d.	-0.5	-0.4	-0.7	0.3	0.1	0.4	1.2	-1.1	n.d.	n.d.
CAMK1D	10.0	0.2	3.2	0.3	1.4	0.1	3.5	0.0	2.1	-0.5	-0.7	0.0	-0.8	-1.6	-1.3	1.4	1.7	2.3	-0.1	1.2
CAMK1G	10.9	0.9	2.6	0.5	0.4	-0.3	2.1	-0.5	1.8	0.4	-0.3	0.1	-0.4	-1.3	-1.0	2.6	3.1	2.1	1.0	0.6
CAMK2B	13.3	0.6	0.8	0.6	0.2	1.9	1.8	0.8	1.1	0.6	-0.1	0.0	0.4	-0.3	-0.7	0.6	1.0	1.7	0.8	0.4
CAMK2D	16.2	0.5	1.2	1.4	0.8	1.2	1.4	1.1	0.5	0.6	0.0	0.2	1.2	0.1	0.7	0.5	0.7	1.2	0.6	1.3
CAMK4	8.6	0.5	0.5	0.2	-0.2	0.5	1.8	0.0	0.3	-0.4	0.2	0.6	0.5	0.0	-0.1	0.6	0.7	1.1	0.1	0.1
CAMKK2	24.6	0.9	2.3	1.8	0.5	1.2	5.6	0.5	1.0	1.1	4.1	5.0	3.4	1.0	3.0	1.1	1.7	1.6	1.6	2.1
CASK	4.8	0.5	0.4	0.2	0.4	0.8	1.0	0.2	0.7	0.2	0.5	-0.1	0.4	-0.1	0.6	-2.9	-6.1	0.6	0.4	0.3
CDC42BPA	2.8	-0.5	0.5	-0.8	0.5	-0.9	0.4	-0.9	0.4	-0.5	0.0	-0.2	-1.1	-0.5	-0.7	0.8	0.5	-1.2	-0.1	-0.4
CDK2	15.5	3.1	4.6	5.6	1.9	6.5	7.8	3.3	2.5	3.3	2.0	2.1	1.5	0.2	0.7	-0.1	0.6	1.3	3.0	5.1
CDKL1	3.1	0.3	0.4	0.3	0.1	0.6	3.8	-0.3	0.6	0.5	-1.3	0.5	0.8	0.0	0.1	0.0	-0.2	0.2	2.3	0.5
CHEK2	17.1	0.8	5.1	2.1	2.5	1.1	2.5	0.7	1.9	0.6	0.8	3.1	4.4	0.6	2.2	0.6	0.4	0.9	1.6	2.1
CLK1	11.9	2.5	3.1	2.9	1.8	2.9	6.6	1.9	2.2	1.3	1.9	2.8	2.7	2.0	1.8	3.2	2.7	3.9	2.9	4.2
CLK3	5.6	0.3	1.7	0.3	1.1	0.4	3.0	0.3	1.4	0.4	0.5	0.9	0.0	0.6	0.5	0.6	0.7	0.4	1.0	1.6
CSNK1D	1.8	0.6	1.0	0.5	0.2	0.8	3.4	0.5	0.5	0.4	0.1	1.1	1.5	0.3	0.6	0.6	1.0	0.9	1.0	0.6
CSNK2A1	2.1	n.d.	n.d.	n.d.	n.d.	n.d.	1.6	n.d.	n.d.	n.d.	3.5	2.9	1.9	1.1	1.1	0.1	0.4	1.1	n.d.	n.d.
CK2A2	5.6	3.2	4.0	3.0	1.8	3.0	6.0	1.9	2.4	1.3	5.4	5.0	2.8	2.3	2.1	1.9	2.7	2.9	4.7	3.5
DAPK1	9.8	0.9	1.9	1.3	-2.4	1.4	3.3	1.0	1.7	0.6	0.1	0.9	1.7	0.5	0.8	0.4	0.6	2.4	1.3	1.2
DAPK3	15.8	0.2	2.5	2.6	3.3	2.9	7.7	1.4	3.0	-0.6	1.0	2.4	3.0	1.2	2.0	2.3	3.3	5.5	2.4	3.2
DCAMKL1	12.4	1.0	2.1	1.7	1.5	2.3	4.3	0.8	1.5	-0.6	1.2	1.7	0.9	1.6	1.6	1.6	4.4	3.2	1.3	1.5
DMPK1	8.7	0.8	0.8	0.5	0.5	0.5	0.9	0.0	0.7	0.1	1.2	1.4	0.7	0.3	0.6	0.7	0.7	1.4	0.5	0.1
DYRK1A	8.7	2.5	0.9	1.7	1.2	0.8	7.1	1.4	2.3	3.4	-0.5	-0.1	-0.7	-0.6	0.5	0.8	0.6	-2.4	0.9	2.4
DYRK2	7.0	1.9	3.1	2.5	2.1	1.8	7.9	1.9	1.5	1.1	2.4	2.4	3.3	2.8	2.0	-0.2	0.0	0.6	2.8	4.6
EPHA2	8.3	1.0	2.4	0.4	0.2	0.9	2.0	0.5	0.6	0.4	-0.2	0.8	0.3	0.1	0.0	-0.1	0.4	0.1	1.0	1.3
EPHA4	6.2	n.d.	n.d.	n.d.	n.d.	n.d.	2.7	n.d.	n.d.	n.d.	0.0	0.6	0.3	-0.1	0.1	0.9	0.8	1.2	n.d.	n.d.
EPHA5	7.9	0.2	1.6	0.2	0.2	0.8	1.8	-0.1	0.9	0.2	0.1	1.0	-0.2	-0.8	-0.2	0.0	1.5	0.6	0.7	1.1
EPHA7	10.3	1.2	2.2	2.1	1.3	-0.9	3.0	-0.4	1.1	0.6	1.1	1.1	1.6	0.7	1.3	0.8	0.8	1.0	0.5	2.2
EPHB1	6.4	n.d.	n.d.	n.d.	n.d.	n.d.	2.3	n.d.	n.d.	n.d.	-0.2	0.6	0.5	-0.4	-0.4	0.8	0.9	1.5	n.d.	n.d.
EPHB3	5.0	0.8	2.2	0.8	0.8	0.8	1.9	0.5	1.9	0.4	-0.6	0.0	0.9	-0.4	-0.4	-1.0	-0.8	0.6	0.7	1.2
FES	6.2	0.6	1.9	0.4	1.5	0.9	1.1	0.7	0.7	0.0	0.7	1.4	0.2	0.2	-0.3	0.4	0.5	0.3	0.6	1.0
FGFR1	5.9	1.1	2.3	0.5	0.5	0.8	2.4	0.2	1.0	0.0	0.1	1.6	0.7	0.3	0.2	1.2	2.3	1.4	1.0	2.8
FGFR2	9.0	1.8	3.4	1.4	1.5	2.0	3.4	0.9	1.4	0.2	0.5	2.3	1.4	0.4	0.8	2.3	3.6	2.5	1.2	3.7
FGFR3	13.3	2.2	3.5	1.8	0.6	2.2	4.0	1.4	1.6	1.2	1.3	3.0	1.4	1.0	0.9	0.9	2.4	2.4	2.3	4.7
FLT1	13.6	3.3	5.2	2.8	2.0	3.0	6.0	2.4	3.2	2.2	2.5	4.1	1.0	0.8	1.3	1.9	4.0	2.7	5.0	4.9
GAK	9.0	4.1	4.8	4.1	2.1	4.7	5.3	1.5	5.5	1.3	1.8	2.7	2.8	0.2	1.8	1.8	1.5	2.3	2.7	1.8
GPRK5	7.7	0.7	0.4	0.3	0.2	0.8	0.9	0.7	0.8	0.7	0.2	0.5	0.2	0.3	0.4	0.4	0.6	0.4	0.8	1.2
GSG2	7.2	-0.1	0.6	0.1	0.2	0.1	1.1	0.0	0.2	-0.2	-0.1	0.5	1.1	0.4	0.5	0.0	-0.2	0.2	0.5	0.3
GSK3B	11.8	7.8	8.6	8.6	6.2	8.6	12.3	7.7	6.0	7.2	3.3	3.9	4.8	2.1	2.9	1.0	1.4	4.2	9.5	10.3
HIPK2	4.4	n.d.	n.d.	n.d.	n.d.	n.d.	4.7	n.d.	n.d.	n.d.	1.1	1.0	0.7	0.1	0.3	0.2	0.2	0.5	n.d.	n.d.
MAP2K1	1.3	-0.2	0.3	-0.1	0.7	-0.3	n.d.	-0.5	0.2	-0.5	0.3	0.1	0.1	0.0	0.1	n.d.	n.d.	n.d.	0.1	0.2
MAP2K4	12.0	2.8	3.4	2.5	0.9	2.5	4.7	1.7	4.0	2.4	3.2	3.8	1.4	0.6	0.9	0.4	0.4	0.5	1.4	2.1
MAP2K6	12.3	2.1	1.9	4.2	1.4	3.5	3.8	2.7	1.7	5.4	1.4	1.8	0.8	0.4	0.3	0.2	0.4	0.2	1.5	2.7
MAP2K7	8.4	0.8	0.9	0.9	0.7	1.1	1.7	0.4	0.8	0.5	0.3	0.2	0.7	0.0	-0.6	0.8	1.4	0.2	n.d.	n.d.
MAP3K5	18.5	n.d.	n.d.	n.d.	n.d.	n.d.	1.9	n.d.	n.d.	n.d.	0.8	0.9	2.7	2.3	0.0	0.7	0.8	0.7	0.5	1.1
MAPK10	7.4	3.2	3.1	3.3	2.9	4.4	10.1	2.4	2.8	1.8	0.2	n.d.	2.8	0.4	1.7	3.2	3.8	5.1	1.2	4.0
MAPK13	7.5	1.0	2.8	1.4	0.5	1.4	2.5	0.7	0.7	1.0	0.8	1.5	0.9	0.3	0.3	-0.5	-0.3	0.0	0.9	1.1
MAPK14	0.3	-0.4	-0.8	-0.3	-0.3	-0.3	0.7	-0.7	-0.2	-0.6	-0.2	0.2	0.0	0.4	-0.2	0.0	0.0	-0.4	0.0	-0.4
MAPK15	14.7	6.1	5.3	7.9	4.1	7.9	11.5	4.7	6.3	5.9	4.4	2.9	0.9	0.4	0.8	-0.2	0.2	2.6	7.4	6.6
MAPK1	1.4	0.6	0.6	0.6	0.3	0.8	1.8	1.0	0.7	0.7	0.4	0.7	0.8	0.6	0.4	-0.5	-0.3	0.0	0.6	0.3
MAPK8	7.8	n.d.	n.d.	n.d.	n.d.	n.d.	5.9	n.d.	n.d.	n.d.	5.2	5.8	5.0	2.8	4.3	1.4	1.4	1.4	n.d.	n.d.
MAPK9	3.7	2.5	3.2	2.2	1.1	2.9	5.8	1.8	3.0	1.7	2.9	3.2	3.0	1.2	2.0	1.0	1.3	1.7	1.2	2.1
MAPKAPK2	3.2	n.d.	n.d.	n.d.	n.d.	n.d.	-0.3	n.d.	n.d.	n.d.	-0.3	-0.2	-0.1	0.1	-0.2	0.1	0.2	0.0	n.d.	n.d.
MARK3	19.0	0.8	3.6	1.8	2.5	1.3	3.7	0.1	1.8	1.5	1.9	2.5	0.5	-0.3	0.7	1.4	2.0	3.6	2.0	3.3
MARK4	16.4	2.2	4.2	3.4	1.7	2.4	4.5	1.4	3.0	3.3	2.2	0.2	0.2	2.2	2.0	1.5	2.5	3.4	2.3	4.5
MELK	13.8	1.2	2.7	2.1	0.8	2.7	4.0	1.2	0.9	0.9	2.3	3.1	2.5	0.8	2.4	0.7	1.0	2.6	2.1	2.3
MERTK	6.6	1.3	3.4	1.7	1.9	0.8	2.0	0.1	2.9	-0.1	1.8	2.4	1.8	0.7	0.7	0.5	0.4	0.9	1.7	1.2
MST3	7.3	0.2	0.0	1.2	-0.3	0.2	4.2	-0.6	0.6	0.1	1.3	1.1	0.7	0.1	0.3	0.7	0.7	1.9	1.2	1.0
MST4	6.1	-0.3	0.0	0.3	0.7	0.1	4.1	-0.3	0.2	-0.7	0.8	1.2	-0.1	0.0	0.4	0.9	1.7	2.7	0.5	0.7
NEK1	-0.6	0.6	1.2	0.3	0.1	0.2	0.9	0.5	0.5	0.4	1.0	1.9	0.6	0.3	0.2	0.0	0.4	0.5	0.5	0.5
NEK2	3.3	n.d.	n.d.	n.d.	n.d.	n.d.	1.2	n.d.	n.d.	n.d.	3.5	2.5	1.3	0.7	1.6	0.7	0.9	-2.6	n.d.	n.d.
NEK7	1.0	n.d.	n.d.	n.d.	n.d.	n.d.	0.7	n.d.	n.d.	n.d.	1.8	0.7	0.4	0.7	0.8	1.1	1.7	0.1	n.d.	n.d.
OSR1	7.7	1.5	1.6	2.5	0.3	1.1	1.4	0.0	1.3	0.6	1.3	1.4	1.1	0.5	0.7	1.9	1.4	3.2	1.0	0.9
PAK1	6.6	-0.4	1.6	0.9	1.1	0.2	0.9	0.6	0.3	-0.4	0.2	0.2	-0.1	0.0	0.4	0.7	1.0	0.0	0.4	0.9
PAK4	12.2	0.9	1.9	0.6	1.0	0.9	3.2	-0.3	0.9	0.2	3.0	4.5	1.6	0.4	1.2	-0.3	0.3	0.8	2.3	1.2
PDK4	0.2	0.9	1.2	0.7	1.8	-1.0	-0.2	0.9	1.2	0.9	-0.9	-0.4	-0.1	-0.2	-0.3	-0.5	-0.5	-0.6	0.8	1.1
PHKG2	21.2	0.2	1.2	1.1	0.5	1.1	2.5	0.0	0.5	-0.3	0.7	2.5	1.3	0.2	0.7	0.5	1.0	0.6	1.0	1.8
PIM1	12.5	-0.3	2.4	-0.3	2.1	-0.5	2.6	-0.9	1.1	-0.6	-0.1	0.1	0.3	0.7	1.3	0.3	0.7	-0.5	0.9	0.5
PIM3	19.7	-0.2	0.7	0.3	0.0	0.4	4.3	-0.7	0.6	-0.4	0.2	1.0	0.6	0.4	0.8	2.0	2.2	0.6	1.2	1.0
PKMYT1	0.2	0.6	1.3	0.5	1.0	0.4	0.5	0.5	1.0	0.2	0.0	-0.2	-0.3	0.2	0.0	0.7	0.5	0.3	0.9	0.6
PLK4	16.0	6.3	6.1	6.9	3.0	7.2	7.7	5.1	6.0	4.1	5.5	6.8	7.6	4.7	7.3	5.1	6.4	9.1	3.8	6.8
RIOK1	0.0	0.3	0.3	-0.1	0.1	0.0	5.7	0.4	1.0	0.8	-0.5	-0.6	0.4	1.0	2.2	1.7	2.1	-0.4	0.0	0.7
RIOK2	1.8	n.d.	n.d.	n.d.	n.d.	n.d.	6.1	n.d.	n.d.	n.d.	n.d.	n.d.	n.d.	n.d.	n.d.	-0.4	-0.5	0.0	n.d.	n.d.
RPS6KA1	3.9	0.6	0.8	0.6	0.5	0.5	1.3	0.7	0.5	1.0	0.0	0.3	1.0	0.3	0.2	-0.5	-0.1	0.3	0.8	0.8
RPS6KA5	13.7	n.d.	n.d.	n.d.	n.d.	n.d.	11.0	n.d.	n.d.	n.d.	n.d.	n.d.	n.d.	n.d.	n.d.	2.1	2.8	2.5	n.d.	n.d.
RPS6KA6	-0.5	-0.6	0.3	-0.7	-0.3	-0.6	0.0	-0.8	-0.4	-0.3	0.6	3.4	1.5	-0.1	0.2	0.6	1.0	0.1	-0.2	0.2
SLK	18.0	1.9	3.5	1.5	1.0	1.2	n.d.	0.6	2.6	0.2	1.0	3.9	-0.2	-0.1	0.8	n.d.	n.d.	n.d.	3.9	2.7
SPRK1	6.8	n.d.	n.d.	n.d.	n.d.	n.d.	0.8	n.d.	n.d.	n.d.	-0.4	0.6	0.3	-0.1	-0.2	-0.3	0.1	0.7	-0.1	0.7
SRC	5.9	2.1	2.1	1.6	0.5	2.6	2.6	0.8	2.0	0.6	-0.8	1.4	0.7	-0.1	0.3	1.1	1.1	1.8	1.9	2.1
STK10	23.3	0.8	3.3	0.3	-0.7	1.2	4.7	-0.4	1.7	-0.7	1.6	4.3	2.2	-0.3	1.0	0.9	1.3	0.4	2.9	2.6
STK17A	11.8	1.2	2.5	3.0	2.2	1.9	3.5	0.5	3.3	-0.1	0.0	0.5	0.3	0.0	0.3	2.8	2.8	0.6	1.9	1.3
STK17B	11.5	3.5	3.6	1.4	1.6	2.1	5.6	2.7	3.0	3.4	-0.8	0.4	0.3	-2.3	-2.0	1.3	1.5	3.0	1.6	3.0
STK38L	12.6	2.5	2.8	1.8	1.2	2.5	8.1	1.1	1.5	0.9	1.7	1.9	0.7	0.2	0.3	0.5	0.9	1.7	1.6	1.3
STK39	9.6	2.6	3.0	4.0	1.8	2.4	2.0	1.5	2.5	2.1	1.2	1.6	0.9	1.3	1.6	1.5	1.4	4.2	2.5	1.6
STK3	16.6	2.4	2.8	3.1	1.6	4.3	5.4	2.7	2.8	1.2	2.4	2.6	1.4	0.5	1.6	1.7	1.8	1.5	4.5	5.3
STK4	14.9	1.7	2.4	1.9	1.6	2.8	5.0	2.0	2.3	2.2	1.1	2.7	0.6	1.1	2.3	1.6	1.8	2.2	2.1	3.7
STK6	17.1	6.3	7.9	8.1	3.2	9.2	10.3	7.0	7.2	4.6	7.5	10.5	5.5	3.7	6.7	5.4	7.7	10.9	3.0	7.5
TAF1	0.2	0.3	0.2	0.1	0.0	-0.2	1.0	0.1	0.5	0.2	-1.7	-1.1	0.6	-0.7	-0.5	-2.1	-2.5	-0.3	0.2	0.2
TIF1	0.8	n.d.	n.d.	n.d.	n.d.	n.d.	0.3	n.d.	n.d.	n.d.	0.8	1.3	0.5	0.3	0.2	1.1	1.0	-0.5	n.d.	n.d.
TLK1	9.4	n.d.	n.d.	n.d.	n.d.	n.d.	4.9	n.d.	n.d.	n.d.	0.4	0.8	1.1	0.3	0.5	1.2	1.1	2.9	n.d.	n.d.
TTK	11.2	2.0	3.4	0.8	1.6	1.0	4.8	0.4	2.3	0.0	3.1	2.7	2.6	2.2	2.1	1.4	2.1	1.1	0.5	0.3
ULK1	11.3	4.3	4.5	6.5	2.3	5.9	2.8	2.0	4.6	2.7	2.0	2.8	2.5	1.2	0.4	0.7	1.3	2.2	0.8	0.4
UKL3	19.6	0.6	1.2	2.8	0.9	1.5	8.2	0.7	1.6	0.3	5.8	6.9	3.3	1.7	3.1	3.4	2.3	9.3	2.6	3.5
VRK1	2.8	0.5	1.5	1.6	1.0	1.9	2.0	1.1	0.6	1.1	-0.1	0.2	-0.1	0.1	0.2	0.2	0.3	0.4	1.0	3.1
WNK1	0.9	0.6	1.5	0.7	0.9	0.6	0.8	0.6	1.2	0.6	0.2	1.3	0.6	0.7	0.3	-0.4	0.1	0.4	0.6	0.5

Table S3: Values of the DSF results of the third series of 3-aminopyrazole based molecules. They were screened against an *in-house* panel of 104 kinases. Staurosporine was used as a reference.

	ΔT$_m$ [°C]														
	staurosporine	38a	38b	39a	39b	40a	40b	40c	41a	41b	41c	42b	42d	43b	43d
R$_1$=		Me	Me	*i*-Prop	*i*-Prop	*t*-Bu	*t*-Bu	*t*-Bu	Amide	Amide	Amide	*i*-Pr ester	*i*-Pr ester	*t*-Bu ester	*t*-Bu ester
CDK16	9.1	5.7	5.8	7.9	8.6	4.4	5.1	5.7	0.9	1.5	1.4	9.1	8.4	9.3	9.2
AAK1	15.6	9.0	9.3	11.2	10.2	8.0	6.5	9.0	4.6	3.1	5.2	5.3	0.5	3.4	1.0
ABL1	10.3	5.8	4.2	6.2	4.3	4.1	1.6	5.8	1.0	0.8	2.5	2.6	1.0	2.6	1.6
AKT3	6.6	0.7	0.1	-0.5	-0.1	-0.4	-0.2	0.7	0.5	0.4	0.6	0.4	0.6	0.3	0.5
AURKB	8.3	0.1	-0.2	-0.9	-1.0	-1.6	-2.0	0.1	-2.2	-3.7	-2.4	n.d.	n.d.	n.d.	n.d.
BMP2K	19.1	14.0	12.9	16.5	15.0	12.8	11.3	14.0	3.8	2.6	5.4	10.1	1.4	7.3	1.7
BMPR2	2.6	7.4	1.8	5.0	4.0	4.8	2.5	7.4	1.0	0.3	1.7	2.2	0.9	1.8	1.4
BMX	7.1	3.4	1.6	-0.5	1.4	0.1	0.1	3.4	0.7	0.3	0.5	0.3	-0.1	0.3	-0.4
BRAF	0.8	3.4	1.0	1.6	0.7	1.4	0.7	3.4	0.4	1.0	1.2	0.7	0.4	0.9	0.7
BRD4	1.1	0.3	-0.4	0.0	-0.1	0.4	-0.9	0.3	2.0	2.3	2.5	-1.6	-2.1	-1.4	-1.5
BRPF1B	-0.1	0.3	-0.6	-0.7	-0.2	-0.9	-0.9	0.3	0.8	2.5	0.5	-1.8	-1.4	-0.5	-0.9
CAMK1D	10.0	4.7	2.3	4.0	2.8	2.9	1.8	4.7	1.9	2.6	1.6	-2.5	-2.4	-2.0	-2.9
CAMK1G	10.9	3.4	1.4	2.6	0.9	1.6	0.6	3.4	1.3	0.1	1.1	0.1	-0.2	0.0	-0.7
CAMK2B	13.3	2.7	1.2	2.5	1.5	1.4	0.8	2.7	0.3	0.4	0.4	-0.8	-1.2	-0.3	-0.5
CAMK2D	16.2	3.9	0.6	2.7	1.5	2.6	1.2	3.9	1.2	1.0	1.3	0.3	-0.4	0.5	-0.1
CAMK4	8.6	6.0	0.8	4.0	2.5	3.1	1.3	6.0	1.0	0.4	0.8	0.1	0.4	0.0	1.2
CAMKK2	24.6	8.8	1.1	6.9	6.1	4.3	2.3	8.8	1.8	1.4	2.5	2.0	-0.6	2.2	0.1
CASK	4.8	1.5	0.2	1.5	0.7	0.8	0.2	1.5	1.7	0.4	0.6	0.6	0.4	1.0	0.4
CDC42BPA	2.8	0.7	0.1	-0.9	0.0	-0.8	-0.7	0.7	0.7	0.2	0.5	-1.3	-0.7	-1.9	-0.8
CDK2	15.5	10.7	4.7	9.8	9.5	5.8	4.3	10.7	1.2	1.2	2.7	6.5	3.0	6.5	3.2
CDKL1	3.1	3.2	0.4	2.0	1.5	0.8	0.2	3.2	0.4	0.3	0.8	1.0	1.7	1.3	2.1
CHEK2	17.1	5.1	2.0	3.2	1.9	1.7	1.1	5.1	0.9	1.7	1.1	1.0	0.6	0.6	1.3
CLK1	11.9	9.3	2.0	7.4	6.8	4.6	4.2	9.3	1.9	1.2	3.1	1.8	0.0	1.7	-0.5
CLK3	5.6	8.3	0.2	2.6	2.4	1.6	1.3	8.3	0.9	1.0	0.9	-0.2	-0.4	0.9	0.0
CSNK1D	1.8	3.1	0.6	2.5	1.0	1.7	0.2	3.1	0.7	0.2	1.4	0.7	0.8	0.8	1.0
CSNK2A1	2.1	3.2	0.3	1.6	1.0	1.2	0.8	3.2	0.9	0.5	0.6	n.d.	n.d.	n.d.	n.d.
CK2A2	5.6	4.6	1.6	3.6	2.4	2.3	0.7	4.6	1.6	0.6	3.1	1.5	1.1	0.1	1.1
DAPK1	9.8	3.4	2.3	2.7	1.6	1.8	0.8	3.4	0.8	0.3	1.1	-0.3	-0.3	-0.5	-0.4
DAPK3	15.8	7.8	6.6	5.3	5.7	2.8	2.9	7.8	1.2	1.2	3.0	1.0	0.1	0.7	0.3
DCAMKL1	12.4	5.3	4.0	4.9	7.1	2.4	2.6	5.3	1.0	0.3	1.0	-1.6	-2.4	-1.8	-2.1
DMPK1	8.7	2.2	1.0	3.2	2.2	2.3	0.9	2.2	0.6	-0.2	-0.2	0.3	-0.1	0.1	-0.1
DYRK1A	8.7	7.2	-0.1	2.8	-0.3	0.6	-0.5	7.2	0.9	0.2	1.8	1.1	0.0	2.9	1.8
DYRK2	7.0	6.5	1.3	3.8	2.5	3.6	1.3	6.5	0.4	0.6	3.2	1.6	0.5	1.5	0.8
EPHA2	8.3	5.3	0.9	3.2	1.2	2.0	0.4	5.3	0.2	0.1	1.1	1.1	0.6	1.7	1.3
EPHA4	6.2	3.9	1.3	2.6	1.3	2.6	1.3	3.9	1.0	0.9	1.3	-0.1	-0.1	0.5	0.0
EPHA5	7.9	4.5	0.9	2.5	0.7	1.9	0.4	4.5	0.6	0.1	1.0	0.2	-0.6	0.8	-2.7
EPHA7	10.3	4.2	0.7	2.4	1.2	0.9	0.0	4.2	1.1	1.1	0.9	-3.5	-3.1	-2.8	-3.4
EPHB1	6.4	4.1	0.8	2.5	1.0	2.3	0.4	4.1	1.6	0.9	1.4	0.7	0.9	1.3	1.0
EPHB3	5.0	3.0	0.2	2.1	0.6	1.5	0.2	3.0	0.6	0.1	0.4	0.6	1.3	1.0	1.7
FES	6.2	3.3	0.9	1.4	1.5	1.3	0.7	3.3	0.5	1.0	1.0	0.1	0.1	0.3	1.1
FGFR1	5.9	3.6	2.3	2.8	1.6	2.6	1.2	3.6	0.8	0.7	0.7	0.8	0.4	1.2	0.6
FGFR2	9.0	3.5	2.8	3.9	2.6	2.9	1.1	3.5	0.7	0.3	0.9	0.9	0.1	1.3	0.3
FGFR3	13.3	5.3	3.8	6.1	3.6	3.7	1.4	5.3	0.6	0.3	1.0	n.d.	n.d.	n.d.	n.d.
FLT1	13.6	5.1	3.9	5.6	3.2	3.3	1.6	5.1	1.8	-1.8	2.8	1.5	0.6	2.3	0.9
GAK	9.0	8.3	2.2	5.9	4.4	5.9	4.0	8.3	3.0	1.8	3.8	3.3	-0.9	3.1	-4.1
GPRK5	7.7	4.6	0.3	3.7	1.0	3.2	0.8	4.6	0.3	0.2	0.5	-1.4	-1.3	-1.2	-1.4
GSG2	7.2	2.2	0.2	0.6	0.4	0.5	0.3	2.2	0.3	0.3	0.5	0.3	0.5	0.0	-0.3
GSK3B	11.8	10.4	4.6	7.0	6.2	5.7	4.9	10.4	2.9	1.3	9.3	7.8	4.7	6.6	2.9
HIPK2	4.4	6.4	1.0	4.1	2.6	2.3	1.4	6.4	0.7	0.9	0.9	0.2	-0.2	0.3	0.1
MAP2K1	1.3	n.d.	n.d.	n.d.	n.d.	n.d.	n.d.	n.d.	n.d.	n.d.	n.d.	n.d.	n.d.	n.d.	n.d.
MAP2K4	12.0	6.5	3.0	6.5	4.8	4.6	2.9	6.5	1.9	0.5	2.7	1.8	0.4	1.1	1.0
MAP2K6	12.3	6.7	4.3	6.7	5.8	4.6	3.0	6.7	0.4	0.3	1.0	5.9	5.0	4.4	3.0
MAP2K7	8.4	4.0	1.3	4.6	1.8	1.7	0.6	4.0	1.5	0.7	1.2	-0.2	-0.3	-0.3	-3.0
MAP3K5	18.5	4.2	1.3	3.2	1.6	2.8	1.3	4.2	0.6	0.7	0.5	0.1	0.1	0.3	0.1
MAPK10	7.4	8.6	5.7	8.6	5.6	5.6	5.0	8.6	3.0	2.8	4.8	-1.9	-3.0	-1.0	-1.6
MAPK13	7.5	5.7	1.1	3.3	1.8	2.7	1.0	5.7	0.1	-0.4	0.7	1.4	1.0	1.3	1.3
MAPK14	0.3	2.9	0.2	0.1	-0.2	0.0	-0.1	2.9	0.5	0.6	1.3	-0.6	-0.5	-0.6	-0.3
MAPK15	14.7	11.0	6.8	12.6	11.8	7.0	5.2	11.0	1.9	0.7	3.9	8.6	7.0	7.4	5.8
MAPK1	1.4	5.1	1.7	3.2	2.6	2.8	1.8	5.1	0.5	0.2	0.7	0.1	-0.1	0.0	0.0
MAPK8	7.8	9.8	4.1	6.0	3.2	6.7	3.4	9.8	4.8	3.9	5.6	1.9	-0.8	2.9	-0.1
MAPK9	3.7	7.0	3.1	4.7	2.8	5.4	3.1	7.0	2.7	2.1	3.4	0.0	-0.7	0.3	-1.4
MAPKAPK2	3.2	0.3	-0.1	0.0	-0.1	-0.1	-0.1	0.3	0.3	0.3	0.4	-0.4	-0.3	-0.3	0.2
MARK3	19.0	5.7	3.2	5.4	4.9	3.2	3.0	5.7	1.3	-0.1	0.9	0.5	-0.4	0.4	-0.5
MARK4	16.4	6.7	2.1	5.1	4.6	3.8	3.2	6.7	2.1	1.6	1.4	1.2	0.3	1.8	-0.5
MELK	13.8	8.3	2.7	6.6	5.1	4.8	3.8	8.3	0.6	0.8	1.8	0.4	-0.8	0.8	0.2
MERTK	6.6	2.8	1.0	2.4	1.7	1.4	0.6	2.8	1.4	1.0	2.1	0.6	-0.8	0.4	-0.8
MST3	7.3	5.9	0.6	3.0	2.3	2.3	1.0	5.9	1.0	0.4	1.1	1.4	0.8	2.0	-0.3
MST4	6.1	6.2	1.0	2.4	2.5	3.0	2.3	6.2	2.7	1.9	2.0	0.8	0.1	1.6	-0.1
NEK1	-0.6	0.3	0.2	0.1	0.1	0.4	0.1	0.3	0.2	0.4	0.6	0.0	0.2	0.2	0.1
NEK2	3.3	2.4	0.7	2.0	0.9	1.9	1.3	2.4	1.9	1.6	1.1	0.7	-1.4	0.8	2.0
NEK7	1.0	2.8	-0.5	0.0	-0.3	0.3	-0.5	2.8	2.4	2.2	2.3	-2.4	-1.7	-2.1	-1.0
OSR1	7.7	4.9	2.3	5.0	4.1	3.5	2.5	4.9	1.7	3.5	2.7	0.1	-0.8	0.0	-0.4
PAK1	6.6	2.4	-0.2	0.5	0.1	-0.2	-0.4	2.4	1.0	0.4	0.7	-0.9	-1.1	-0.4	-0.6
PAK4	12.2	5.0	1.2	3.9	2.8	2.8	1.2	5.0	1.0	0.8	1.1	0.8	-0.2	0.5	-0.9
PDK4	0.2	-0.1	-0.1	-0.4	-0.5	-0.3	-0.3	-0.1	-0.3	-0.2	-0.2	n.d.	n.d.	n.d.	n.d.
PHKG2	21.2	4.8	1.0	3.1	3.4	1.1	0.8	4.8	0.9	0.6	1.4	0.7	-0.2	1.1	0.5
PIM1	12.5	2.7	-0.2	-0.5	-0.3	-0.1	-0.9	2.7	1.5	0.7	1.6	0.1	0.2	-0.4	-0.7
PIM3	19.7	3.0	1.1	1.4	1.4	0.9	0.8	3.0	2.1	1.8	2.7	-1.2	-1.1	-1.2	-1.6
PKMYT1	0.2	0.1	0.5	-0.4	-0.2	0.0	0.2	0.1	0.6	0.6	0.5	-2.8	-3.2	-3.0	-2.0
PLK4	16.0	6.5	7.9	7.1	5.4	4.6	1.8	6.5	2.3	1.2	3.5	7.2	3.7	7.2	5.5
RIOK1	0.0	5.0	1.8	3.8	0.6	2.6	1.1	5.0	2.4	3.0	4.2	n.d.	n.d.	n.d.	n.d.
RIOK2	1.8	9.1	4.4	8.2	3.2	8.1	3.4	9.1	1.8	1.2	1.8	n.d.	n.d.	n.d.	n.d.
RPS6KA1	3.9	1.6	0.1	0.4	0.3	0.1	0.0	1.6	0.5	0.6	1.4	-0.2	-0.3	-0.2	0.2
RPS6KA5	13.7	12.8	6.5	6.0	7.0	4.5	4.5	12.8	2.6	1.8	7.2	-0.1	0.8	0.5	1.7
RPS6KA6	-0.5	1.1	0.1	-0.2	0.0	0.0	-0.2	1.1	0.3	1.2	1.7	n.d.	n.d.	n.d.	n.d.
SLK	18.0	n.d.	n.d.	n.d.	n.d.	n.d.	n.d.	n.d.	n.d.	n.d.	n.d.	0.1	-0.6	1.5	0.1
SPRK1	6.8	3.2	0.2	1.7	1.3	1.5	0.7	3.2	0.2	0.4	0.5	-0.1	-0.2	0.0	0.4
SRC	5.9	3.4	1.9	2.8	1.7	2.3	0.4	3.4	0.5	0.3	1.1	0.1	0.4	0.4	0.6
STK10	23.3	5.1	1.3	3.7	2.5	1.8	-0.3	5.1	1.2	0.2	0.6	0.6	-0.7	0.8	-0.8
STK17A	11.8	10.6	1.9	5.7	4.6	6.0	5.2	10.6	3.5	3.7	3.8	0.1	-0.4	-0.2	-0.6
STK17B	11.5	4.0	1.9	2.6	1.9	0.8	0.8	4.0	1.0	0.8	2.7	-3.2	-2.8	-2.4	-2.5
STK38L	12.6	10.3	4.7	10.8	9.3	5.3	4.3	10.3	0.6	0.7	1.9	0.0	0.0	-0.3	0.0
STK39	9.6	4.7	3.4	5.6	6.0	3.1	2.9	4.7	1.3	0.7	0.3	0.1	-0.6	-0.1	-0.9
STK3	16.6	9.0	3.8	7.3	6.0	5.7	3.8	9.0	0.7	0.5	1.3	0.1	-1.0	2.9	0.3
STK4	14.9	8.7	4.4	6.2	5.8	5.1	4.2	8.7	2.0	1.1	1.7	0.9	-0.5	2.1	-0.2
STK6	17.1	10.6	12.4	12.7	10.2	9.0	5.3	10.6	4.0	2.3	6.3	7.5	3.4	7.3	4.0
TAF1	0.2	0.6	-0.1	0.0	-0.1	0.3	-0.3	0.6	0.0	-0.2	0.6	0.2	0.5	0.2	0.0
TIF1	0.8	0.9	0.1	-0.7	-1.6	0.5	-0.5	0.9	2.4	2.4	1.6	n.d.	n.d.	n.d.	n.d.
TLK1	9.4	4.5	4.0	4.1	3.4	2.6	1.9	4.5	2.0	1.8	2.4	1.2	-1.2	-1.3	-1.4
TTK	11.2	8.4	1.2	6.6	6.0	6.0	4.9	8.4	4.4	1.5	2.6	0.1	-0.1	-0.3	-0.2
ULK1	11.3	6.3	2.1	4.6	3.7	3.2	2.4	6.3	1.8	2.2	1.7	n.d.	n.d.	n.d.	n.d.
UKL3	19.6	10.3	6.0	9.6	8.1	6.9	3.9	10.3	1.7	2.0	3.0	6.0	2.3	5.0	3.3
VRK1	2.8	5.0	1.6	3.0	2.6	2.4	2.8	5.0	0.8	1.0	1.2	2.1	0.5	2.8	0.9
WNK1	0.9	1.1	0.1	0.8	0.4	0.9	0.4	1.1	0.3	0.6	0.7	-0.4	0.1	-0.3	-0.1

Table S4: DSF shifts for GSK3B and cellular EC_{50} values of GSK3A and GSK3B for **1**, **21a**, **21c**, and **24**.

	ΔTm [°C]	NanoBRET EC_{50} [µM]	
Compound	GSK3B	GSK3A	GSK3B
**1**	14.0	4 nM	4 nM
**21a**	7.8	10 µM	> 45 µM
**21c**	8.6	13 µM	> 45 µM
**24**	9.5	5 µM	18 µM

Table S5: Cellular EC_{50} values of **1**, **21i**, **42d**, and **43d** against the CDK family. Values were determined in a 11-point dose−response curve in duplicate measurements.

	NanoBRET EC_{50} [nM] ± SEM			
	**1**	**21i**	**42d**	**43d**
CDK1/Cyclin B1	63.1 ± 10.4	> 50000	9301.0 ± 5789.0	> 50000
CDK1/Cyclin E1	5.2 ± 2.5	6415.0 ± 3456.9	200.1 ± 42.8	581.9 ± 360.7
CDK2/Cyclin A1	60.0 ± 41.1	36735.0 ± 3315.0	32475.0 ± 13895.0	1670.5 ± 419.5
CDK2/Cyclin A2	28.1 ± 7.5	11657.7 ± 2334.1	2843.0 ± 1350.8	1524.0 ± 448.8
CDK2/Cyclin D1	38.6 ± 17.3	33520.0 ± 1520.0	4860.5 ± 2389.6	3074.1 ± 1859.4
CDK2/Cyclin E1	26.7 ± 6.3	20950.0 ± 5690.2	2598.8 ± 913.6	1864.8 ± 582.2
CDK3/Cyclin E1	11.8 ± 4.5	12413.3 ± 4611.5	1274.7 ± 521.9	2749.0 ± 958.8
CDK3/Cyclin E2	38.4 ± 30.4	38830.0 ± 4640.0	26862.5 ± 13394.8	16085.7 ± 7622.5
CDK4/Cyclin D1	93.6 ± 23.6	10931.0 ± 3918.6	1598.3 ± 409.1	2140.8 ± 749.8
CDK4/Cyclin D3	32.1 ± 3.1	6782.8 ± 1457.4	508.0 ± 86.6	908.3 ± 112.6
CDK5/CDK5R1	30.3 ± 10.3	> 50000	> 50000	28550.0 ± 4650.0
CDK5/CDK5R2	11.9 ± 2.8	> 50000	> 50000	38570.0 ± 5850.0
CDK6/Cyclin D1	10.5 ± 3.8	7452.5 ± 1744.2	959.0 ± 390.8	1737.5 ± 206.1
CDK6/Cyclin D3	45.3 ± 35.6	6548.3 ± 3476.4	2080.3 ± 934.8	2909.0 ± 1572.4
CDK7	102.6 ± 43.2	10381.8 ± 4765.2	1141.9 ± 658.1	1459.9 ± 551.9
CDK9/Cyclin K	17.4 ± 5.4	7004.3 ± 2117.2	574.6 ± 157.4	993.4 ± 306.8
CDK9/Cyclin T1	86.5 ± 10.9	6872.5 ± 2188.8	408.3 ± 140.0	804.6 ± 240.3
CDK10/Cyclin L2	324.7 ± 36.4	7930.0 ± 2932.2	333.6 ± 0.2	651.2 ± 34.8
CDK11A/Cyclin K	999.3 ± 475.6	21085.0 ± 10975.1	3096.5 ± 1352.0	7976.5 ± 3738.4
CDK11A/Cyclin L2	759.7 ± 460.2	20465.3 ± 11780.9	2093.0 ± 598.9	3455.0 ± 1843.5
CDK11B/Cyclin K	926.4 ± 649.2	22900.0 ± 3740.0	3862.8 ± 1054.7	7962.3 ± 3288.7
CDK11B/Cyclin L2	668.5 ± 405.9	16731.3 ± 7892.1	2993.5 ± 1327.5	3582.8 ± 1726.4
CDK13/Cyclin K	66.1 ± 19.9	24200.0 ± 5880.0	> 50000	3833.0 ± 2281.0
CDK14/Cyclin Y	33.7 ± 8.2	1371.0 ± 374.4	88.2 ± 48.8	72.1 ± 23.2
CDK15/Cyclin Y	93.4 ± 41.7	2088.5 ± 495.8	167.4 ± 14.9	301.6 ± 34.1
CDK16/Cyclin Y	14.6 ± 5.5	380.2 ± 230.2	44.0 ± 7.5	33.4 ± 5.8
CDK17/Cyclin Y	9.9 ± 1.2	673.1 ± 41.3	33.7 ± 2.5	21.2 ± 2.4
CDK18/Cyclin Y	25.0 ± 8.9	1624.8 ± 287.4	127.7 ± 10.4	120.6 ± 5.9
CDK20/Cyclin H	2657.8 ± 567.7	8416.5 ± 628.6	744.5 ± 350.4	1706.8 ± 222.0

Table S6: Further information about the NanoBRET assay setup.

Construct CDK	Catalog #/ CAS #	NanoLuc orientation	Construct Cyclin	Catalog #/ CAS #	Tracer	Tracer $K_{D,app}$ [µM]	Tracer, used [µM]
CDK1	CS1810C163	C	CCNB1	CS1810C170	K9	1.23	1.00
CDK1	CS1810C163	C	CCNE1	CS1810C171	K9	0.66	0.70
CDK2	CS1810C164	C	CCNA1	CS1810C172	K10	0.31	0.30
CDK2	CS1810C164	C	CCNA2	Promega	K10	0.26	0.30
CDK2	CS1810C164	C	CCND1	CS1810C118	K10	0.26	0.30
CDK2	CS1810C164	C	CCNE1	CS1810C171	K10	0.29	0.30
CDK3	CS1810C197	C	CCNE1	CS1810C171	K10	0.29	0.30
CDK3	CS1810C197	C	CCNE2	Promega	K12	2.14	1.00
CDK4	CS1810C86	N	CCND1	CS1810C118	K10	0.31	0.40
CDK4	CS1810C86	N	CCND3	CS1810C87	K10	0.24	0.30
CDK5	NV1121	C	CDK5R1	CS1810C173	K10	0.28	0.30
CDK5	NV1121	C	CDK5R2	CS1810C174	K10	0.31	0.30
CDK6	CS1810C117	N	CCND1	CS1810C118	K10	0.17	0.20
CDK6	CS1810C117	N	CCND3	CS1810C87	K7	0.14	0.20
CDK7	CS1810C117	N			K10	0.25	0.30
CDK8	CS1810C115	N	CCNC	CS1810C242	K8	0.13	0.20
CDK9	CS1810C92	N	CCNK	CS1810C93	K8	0.20	0.20
CDK9	CS1810C92	N	CCNT1	CS1810C177	K8	0.51	0.55
CDK10	CS1810C187	C	CCNL2	CS1810C189	K10	0.29	0.30
CDK11A	CS1810C188	C	CCNK	CS1810C93	K12	0.67	0.70
CDK11A	CS1810C188	C	CCNL2	CS1810C189	K12	0.69	0.70
CDK11B	Promega	C	CCNK	CS1810C93	K12	0.39	0.40
CDK11B	Promega	C	CCNL2	CS1810C189	K12	0.59	0.60
CDK12	CS1810C49	C	CCNK	CS1810C93	K12	0.25	0.30
CDK13	CS1810C51	C	CCNK	CS1810C93	K12	0.40	0.40
CDK14	CS1810C190	C	CCNY	CS1810C178	K10	0.28	0.30
CDK15	CS1810C191	N	CCNY	CS1810C178	K10	0.25	0.30
CDK16	CS1810C192	C	CCNY	CS1810C178	K10	0.17	0.20
CDK17	CS1810C193	C	CCNY	CS1810C178	K10	0.16	0.30
CDK18	CS1810C194	C	CCNY	CS1810C178	K10	0.19	0.30
CDK19	CS1810C195	N	CCNC	CS1810C242	K8	0.14	0.20
CDK20	CS1810C196	N	CCNH	CS1810C175	K10	0.27	0.20

Table S7. FUCCI cell cycle assay data of **1**, **21i**, **42d**, **and 43d**. Milciclib was used as a reference.

Compound ID	Conc [μM]	Time [min]	Cell Count	Ratio Hoechst High Cell count	Ratio Normal Cells	Ratio Healthy Cell Count	Ratio Fragmented Cell Count	Ratio Pyknosed Cell Count	Ratio Red Cell Count	Ratio Green Cell Count	Ratio Yellow Cell Count	normalized normal cell count
DMSO	10	0	105	0.00%	100.00%	97.14%	0.00%	2.86%	73.53%	12.75%	13.73%	0.44
DMSO	10	0	129	0.78%	99.22%	96.88%	3.13%	0.00%	76.61%	12.10%	11.29%	0.54
DMSO	10	0	164	0.00%	100.00%	94.51%	1.83%	3.66%	74.84%	16.13%	9.03%	0.69
DMSO	10	0	261	0.00%	100.00%	95.02%	1.92%	3.07%	78.23%	12.10%	9.68%	1.10
DMSO	10	0	275	0.00%	100.00%	97.09%	2.18%	0.73%	78.28%	10.86%	10.86%	1.16
DMSO	10	0	265	0.00%	100.00%	96.60%	1.51%	1.89%	76.56%	12.11%	11.33%	1.12
DMSO	10	0	206	0.00%	100.00%	93.20%	2.91%	3.88%	71.35%	18.23%	10.42%	0.87
DMSO	10	0	275	0.00%	100.00%	96.00%	1.45%	2.55%	70.08%	13.26%	16.67%	1.16
DMSO	10	0	229	0.00%	100.00%	96.07%	1.31%	2.62%	78.64%	11.82%	9.55%	0.97
DMSO	10	0	260	0.00%	100.00%	97.69%	0.77%	1.54%	72.44%	11.42%	16.14%	1.10
DMSO	10	0	261	0.38%	99.62%	96.54%	1.92%	1.54%	70.92%	13.94%	15.14%	1.10
DMSO	10	0	300	0.00%	100.00%	97.00%	2.00%	1.00%	70.45%	15.81%	13.75%	1.27
DMSO	10	0	328	0.30%	99.70%	96.02%	1.53%	2.45%	72.61%	13.06%	14.33%	1.38
DMSO	10	0	256	0.00%	100.00%	94.53%	3.52%	1.95%	67.36%	18.18%	14.46%	1.08
DMSO	10	6	102	4.90%	95.10%	95.88%	0.00%	4.12%	10.75%	65.59%	23.66%	0.48
DMSO	10	6	119	1.68%	98.32%	94.87%	0.00%	5.13%	10.81%	63.06%	26.13%	0.57
DMSO	10	6	143	2.80%	97.20%	93.53%	0.00%	6.47%	10.00%	66.92%	23.08%	0.68
DMSO	10	6	232	1.72%	98.28%	94.30%	0.00%	5.70%	8.37%	65.58%	26.05%	1.12
DMSO	10	6	251	2.79%	97.21%	95.90%	0.00%	4.10%	7.69%	70.51%	21.79%	1.20
DMSO	10	6	223	4.04%	95.96%	93.46%	0.00%	6.54%	6.00%	74.00%	20.00%	1.05
DMSO	10	6	196	4.59%	95.41%	96.26%	0.00%	3.74%	6.67%	72.78%	20.56%	0.92
DMSO	10	6	236	1.69%	98.31%	96.12%	0.00%	3.88%	5.83%	65.47%	28.70%	1.14
DMSO	10	6	218	4.13%	95.87%	97.61%	0.00%	2.39%	7.35%	71.08%	21.57%	1.03
DMSO	10	6	235	2.13%	97.87%	95.65%	0.00%	4.35%	5.00%	73.64%	21.36%	1.13
DMSO	10	6	231	4.76%	95.24%	97.73%	0.00%	2.27%	4.65%	66.98%	28.37%	1.08
DMSO	10	6	260	1.92%	98.08%	95.29%	0.00%	4.71%	10.70%	71.19%	18.11%	1.25
DMSO	10	6	278	2.88%	97.12%	94.07%	0.00%	5.93%	6.30%	73.23%	20.47%	1.32
DMSO	10	6	219	3.65%	96.35%	93.84%	0.00%	6.16%	9.09%	70.20%	20.71%	1.04
DMSO	10	12	110	3.64%	96.36%	95.28%	0.00%	4.72%	5.94%	82.18%	11.88%	0.49
DMSO	10	12	129	0.78%	99.22%	96.09%	0.00%	3.91%	12.20%	73.17%	14.63%	0.59

DMSO	10	12	155	1.29%	98.71%	94.12%	0.00%	5.88%	10.42%	77.78%	11.81%	0.71
DMSO	10	12	233	2.15%	97.85%	93.86%	0.00%	6.14%	7.48%	73.83%	18.69%	1.06
DMSO	10	12	259	1.16%	98.84%	94.92%	0.00%	5.08%	7.41%	79.01%	13.58%	1.19
DMSO	10	12	243	4.12%	95.88%	94.42%	0.00%	5.58%	9.55%	83.64%	6.82%	1.08
DMSO	10	12	205	2.93%	97.07%	94.47%	0.00%	5.53%	7.45%	74.47%	18.09%	0.92
DMSO	10	12	250	1.60%	98.40%	94.72%	0.00%	5.28%	6.01%	77.68%	16.31%	1.14
DMSO	10	12	224	2.68%	97.32%	95.87%	0.00%	4.13%	8.61%	79.90%	11.48%	1.01
DMSO	10	12	242	1.24%	98.76%	96.23%	0.00%	3.77%	6.96%	80.87%	12.17%	1.11
DMSO	10	12	243	1.65%	98.35%	93.72%	0.00%	6.28%	8.93%	73.21%	17.86%	1.11
DMSO	10	12	268	1.49%	98.51%	96.59%	0.00%	3.41%	9.80%	78.04%	12.16%	1.23
DMSO	10	12	292	2.74%	97.26%	94.37%	0.00%	5.63%	8.21%	80.22%	11.57%	1.32
DMSO	10	12	228	2.63%	97.37%	94.14%	0.00%	5.86%	10.05%	77.51%	12.44%	1.03
DMSO	10	24	121	0.83%	99.17%	93.33%	0.00%	6.67%	37.50%	56.25%	6.25%	0.50
DMSO	10	24	133	0.75%	99.25%	95.45%	0.00%	4.55%	45.24%	41.27%	13.49%	0.56
DMSO	10	24	167	2.99%	97.01%	91.98%	0.00%	8.02%	44.30%	47.65%	8.05%	0.68
DMSO	10	24	253	3.16%	96.84%	92.65%	0.00%	7.35%	37.89%	45.81%	16.30%	1.03
DMSO	10	24	283	2.47%	97.53%	93.84%	0.00%	6.16%	35.14%	51.74%	13.13%	1.16
DMSO	10	24	262	3.44%	96.56%	93.68%	0.00%	6.32%	45.15%	41.77%	13.08%	1.06
DMSO	10	24	229	3.93%	96.07%	95.45%	0.00%	4.55%	40.00%	48.10%	11.90%	0.93
DMSO	10	24	288	2.08%	97.92%	94.68%	0.00%	5.32%	44.94%	39.33%	15.73%	1.19
DMSO	10	24	248	0.81%	99.19%	93.50%	0.00%	6.50%	41.74%	48.26%	10.00%	1.03
DMSO	10	24	273	1.83%	98.17%	97.39%	0.00%	2.61%	41.00%	47.89%	11.11%	1.13
DMSO	10	24	260	2.69%	97.31%	90.51%	0.00%	9.49%	37.55%	46.29%	16.16%	1.06
DMSO	10	24	302	2.32%	97.68%	95.59%	0.00%	4.41%	39.72%	45.74%	14.54%	1.24
DMSO	10	24	335	2.69%	97.31%	94.48%	0.00%	5.52%	44.81%	47.08%	8.12%	1.37
DMSO	10	24	255	1.57%	98.43%	92.83%	0.00%	7.17%	46.35%	43.35%	10.30%	1.06
DMSO	10	48	151	0.66%	99.34%	98.00%	0.00%	2.00%	53.74%	38.10%	8.16%	0.49
DMSO	10	48	179	0.56%	99.44%	97.75%	0.00%	2.25%	51.72%	39.08%	9.20%	0.58
DMSO	10	48	199	1.51%	98.49%	97.45%	0.00%	2.55%	50.26%	37.70%	12.04%	0.64
DMSO	10	48	329	0.61%	99.39%	96.02%	0.00%	3.98%	49.04%	38.54%	12.42%	1.07
DMSO	10	48	353	1.98%	98.02%	96.53%	0.00%	3.47%	45.81%	40.72%	13.47%	1.13
DMSO	10	48	326	0.61%	99.39%	96.60%	0.00%	3.40%	53.04%	36.42%	10.54%	1.06
DMSO	10	48	268	1.12%	98.88%	96.60%	0.00%	3.40%	50.39%	40.63%	8.98%	0.87
DMSO	10	48	359	0.28%	99.72%	96.37%	0.00%	3.63%	56.23%	35.65%	8.12%	1.17
DMSO	10	48	300	0.00%	100.00%	95.67%	0.00%	4.33%	49.13%	38.33%	12.54%	0.98
DMSO	10	48	333	1.50%	98.50%	98.17%	0.00%	1.83%	50.93%	36.96%	12.11%	1.07

DMSO	10	48	320	2.19%	97.81%	96.49%	0.00%	3.51%	48.34%	39.40%	12.25%	1.02
DMSO	10	48	364	1.37%	98.63%	96.38%	0.00%	3.62%	47.40%	39.88%	12.72%	1.17
DMSO	10	48	467	1.50%	98.50%	96.74%	0.00%	3.26%	45.62%	40.67%	13.71%	1.51
DMSO	10	48	376	0.27%	99.73%	97.33%	0.00%	2.67%	45.21%	44.38%	10.41%	1.23
DMSO	10	72	268	0.00%	100.00%	98.13%	0.00%	1.87%	44.49%	43.35%	12.17%	0.52
DMSO	10	72	240	0.42%	99.58%	98.74%	0.00%	1.26%	52.54%	29.24%	18.22%	0.46
DMSO	10	72	349	0.00%	100.00%	97.99%	0.00%	2.01%	52.34%	30.12%	17.54%	0.68
DMSO	10	72	568	0.53%	99.47%	98.41%	0.00%	1.59%	54.68%	34.71%	10.61%	1.10
DMSO	10	72	550	0.18%	99.82%	99.45%	0.00%	0.55%	48.35%	36.26%	15.38%	1.07
DMSO	10	72	553	0.18%	99.82%	99.64%	0.00%	0.36%	48.73%	39.64%	11.64%	1.07
DMSO	10	72	421	0.24%	99.76%	99.05%	0.00%	0.95%	56.49%	30.53%	12.98%	0.82
DMSO	10	72	585	0.68%	99.32%	98.80%	0.00%	1.20%	56.10%	32.93%	10.98%	1.13
DMSO	10	72	531	0.94%	99.06%	98.29%	0.00%	1.71%	57.83%	28.63%	13.54%	1.02
DMSO	10	72	534	1.12%	98.88%	98.67%	0.00%	1.33%	45.30%	41.27%	13.44%	1.03
DMSO	10	72	578	0.87%	99.13%	98.60%	0.00%	1.40%	49.91%	35.58%	14.51%	1.11
DMSO	10	72	631	0.32%	99.68%	99.36%	0.00%	0.64%	54.08%	33.28%	12.64%	1.22
DMSO	10	72	759	1.19%	98.81%	99.60%	0.00%	0.40%	42.57%	42.70%	14.73%	1.46
DMSO	10	72	682	0.59%	99.41%	98.67%	0.00%	1.33%	47.68%	39.16%	13.15%	1.32
21i	1	0	155	0.65%	99.35%	95.45%	0.65%	3.90%	77.55%	17.69%	4.76%	0.65
21i	1	0	152	0.00%	100.00%	95.39%	1.32%	3.29%	75.86%	15.17%	8.97%	0.64
21i	1	0	298	0.00%	100.00%	95.97%	0.34%	3.69%	75.17%	12.59%	12.24%	1.26
21i	1	0	291	0.69%	99.31%	97.92%	0.69%	1.38%	81.63%	9.19%	9.19%	1.22
21i	10	0	247	0.00%	100.00%	98.79%	0.40%	0.81%	73.36%	11.89%	14.75%	1.04
21i	10	0	235	0.00%	100.00%	94.47%	3.40%	2.13%	80.18%	8.56%	11.26%	0.99
21i	10	0	306	0.00%	100.00%	94.44%	1.96%	3.59%	72.32%	15.57%	12.11%	1.29
21i	10	0	281	0.00%	100.00%	96.44%	1.78%	1.78%	70.11%	13.65%	16.24%	1.19
21i	5	0	177	0.00%	100.00%	96.05%	1.13%	2.82%	73.53%	17.06%	9.41%	0.75
21i	5	0	221	0.00%	100.00%	94.12%	2.26%	3.62%	75.48%	12.02%	12.50%	0.93
21i	5	0	294	0.00%	100.00%	94.56%	4.08%	1.36%	75.18%	14.03%	10.79%	1.24
21i	5	0	263	0.00%	100.00%	94.30%	3.42%	2.28%	66.94%	19.76%	13.31%	1.11
21i	1	6	145	2.07%	97.93%	93.66%	0.00%	6.34%	12.03%	61.65%	26.32%	0.70
21i	1	6	138	6.52%	93.48%	96.90%	0.00%	3.10%	8.00%	58.40%	33.60%	0.63
21i	1	6	263	2.66%	97.34%	94.53%	0.00%	5.47%	10.33%	68.60%	21.07%	1.26
21i	1	6	256	4.30%	95.70%	94.29%	0.00%	5.71%	10.39%	66.67%	22.94%	1.20
21i	10	6	228	4.82%	95.18%	93.09%	0.46%	6.45%	6.93%	77.72%	15.35%	1.06
21i	10	6	202	3.47%	96.53%	90.77%	0.51%	8.72%	7.91%	67.80%	24.29%	0.96

21i	10	6	241	7.88%	92.12%	83.78%	0.45%	15.77%	9.68%	76.88%	13.44%	1.09
21i	10	6	226	7.52%	92.48%	84.21%	0.96%	14.83%	10.80%	73.30%	15.91%	1.03
21i	5	6	165	5.45%	94.55%	91.03%	0.00%	8.97%	10.56%	74.65%	14.79%	0.77
21i	5	6	192	6.77%	93.23%	89.39%	0.00%	10.61%	6.88%	73.13%	20.00%	0.88
21i	5	6	240	2.08%	97.92%	91.49%	0.00%	8.51%	6.51%	73.02%	20.47%	1.15
21i	5	6	234	4.27%	95.73%	91.96%	0.45%	7.59%	9.22%	70.87%	19.90%	1.10
21i	1	12	150	0.67%	99.33%	91.95%	0.67%	7.38%	10.95%	78.10%	10.95%	0.69
21i	1	12	141	3.55%	96.45%	95.59%	0.00%	4.41%	9.23%	74.62%	16.15%	0.63
21i	1	12	276	2.17%	97.83%	95.56%	0.00%	4.44%	8.14%	79.84%	12.02%	1.25
21i	1	12	259	1.54%	98.46%	92.16%	0.00%	7.84%	10.21%	68.09%	21.70%	1.18
21i	10	12	230	3.48%	96.52%	92.79%	0.45%	6.76%	5.83%	85.44%	8.74%	1.03
21i	10	12	203	8.37%	91.63%	90.86%	0.00%	9.14%	10.65%	73.96%	15.38%	0.86
21i	10	12	261	8.43%	91.57%	82.85%	0.00%	17.15%	9.09%	78.28%	12.63%	1.11
21i	10	12	232	7.33%	92.67%	82.33%	0.93%	16.74%	13.56%	74.01%	12.43%	1.00
21i	5	12	167	3.59%	96.41%	87.58%	0.00%	12.42%	9.22%	78.01%	12.77%	0.75
21i	5	12	194	2.58%	97.42%	91.01%	0.00%	8.99%	6.40%	83.72%	9.88%	0.88
21i	5	12	264	1.89%	98.11%	90.73%	0.00%	9.27%	6.81%	85.11%	8.09%	1.20
21i	5	12	254	3.15%	96.85%	94.31%	0.00%	5.69%	9.91%	81.90%	8.19%	1.14
21i	1	24	167	2.40%	97.60%	90.80%	0.00%	9.20%	35.81%	51.35%	12.84%	0.69
21i	1	24	150	3.33%	96.67%	88.28%	0.00%	11.72%	37.50%	53.13%	9.38%	0.61
21i	1	24	303	0.99%	99.01%	90.33%	0.00%	9.67%	40.22%	51.29%	8.49%	1.26
21i	1	24	288	3.13%	96.88%	89.61%	0.00%	10.39%	34.00%	57.60%	8.40%	1.17
21i	10	24	222	6.31%	93.69%	78.37%	0.00%	21.63%	12.88%	79.75%	7.36%	0.87
21i	10	24	209	11.00%	89.00%	82.26%	0.00%	17.74%	11.76%	77.78%	10.46%	0.78
21i	10	24	271	8.86%	91.14%	74.90%	0.40%	24.70%	13.51%	73.51%	12.97%	1.04
21i	10	24	254	17.32%	82.68%	77.62%	0.00%	22.38%	14.11%	77.30%	8.59%	0.88
21i	5	24	183	3.28%	96.72%	84.75%	0.00%	15.25%	24.00%	66.67%	9.33%	0.74
21i	5	24	207	5.31%	94.69%	85.71%	0.00%	14.29%	17.86%	76.79%	5.36%	0.82
21i	5	24	270	4.44%	95.56%	86.43%	0.00%	13.57%	23.77%	69.06%	7.17%	1.09
21i	5	24	270	4.44%	95.56%	87.21%	0.00%	12.79%	31.56%	60.44%	8.00%	1.09
21i	1	48	200	0.50%	99.50%	95.98%	0.00%	4.02%	63.87%	29.84%	6.28%	0.65
21i	1	48	164	1.83%	98.17%	93.79%	0.00%	6.21%	59.60%	31.79%	8.61%	0.53
21i	1	48	320	2.19%	97.81%	93.93%	0.00%	6.07%	59.86%	32.31%	7.82%	1.02
21i	1	48	301	1.99%	98.01%	91.53%	0.00%	8.47%	59.26%	32.96%	7.78%	0.97
21i	10	48	206	10.19%	89.81%	68.65%	0.54%	30.81%	33.07%	55.12%	11.81%	0.61
21i	10	48	206	15.53%	84.47%	71.84%	0.00%	28.16%	28.00%	56.00%	16.00%	0.57

21i	10	48	247	14.17%	85.83%	62.26%	1.89%	35.85%	28.03%	59.09%	12.88%	0.69
21i	10	48	243	16.05%	83.95%	62.75%	0.00%	37.25%	31.25%	56.25%	12.50%	0.67
21i	5	48	188	4.26%	95.74%	91.11%	0.00%	8.89%	54.88%	34.15%	10.98%	0.59
21i	5	48	226	3.54%	96.46%	89.91%	0.00%	10.09%	52.55%	36.22%	11.22%	0.71
21i	5	48	272	2.94%	97.06%	85.61%	0.00%	14.39%	55.75%	35.40%	8.85%	0.86
21i	5	48	240	4.17%	95.83%	89.13%	0.00%	10.87%	60.00%	33.66%	6.34%	0.75
21i	1	72	229	0.87%	99.13%	96.04%	0.00%	3.96%	68.35%	22.48%	9.17%	0.44
21i	1	72	202	0.99%	99.01%	95.50%	0.00%	4.50%	61.78%	27.75%	10.47%	0.39
21i	1	72	380	0.53%	99.47%	97.88%	0.00%	2.12%	67.57%	19.46%	12.97%	0.73
21i	1	72	363	2.20%	97.80%	96.06%	0.00%	3.94%	65.69%	23.46%	10.85%	0.69
21i	10	72	212	7.55%	92.45%	70.92%	2.55%	26.53%	42.45%	46.76%	10.79%	0.38
21i	10	72	183	14.75%	85.25%	66.03%	2.56%	31.41%	29.13%	57.28%	13.59%	0.30
21i	10	72	215	15.81%	84.19%	61.33%	1.10%	37.57%	36.04%	46.85%	17.12%	0.35
21i	10	72	229	16.59%	83.41%	61.78%	1.57%	36.65%	29.66%	54.24%	16.10%	0.37
21i	5	72	173	3.47%	96.53%	85.03%	0.60%	14.37%	67.61%	26.76%	5.63%	0.32
21i	5	72	222	2.70%	97.30%	84.72%	0.00%	15.28%	70.49%	22.40%	7.10%	0.42
21i	5	72	270	2.59%	97.41%	92.78%	0.00%	7.22%	66.80%	23.77%	9.43%	0.51
21i	5	72	281	2.49%	97.51%	93.07%	0.36%	6.57%	64.31%	25.49%	10.20%	0.53
43d	1	0	189	0.00%	100.00%	95.24%	3.17%	1.59%	77.78%	11.67%	10.56%	0.80
43d	1	0	203	0.00%	100.00%	94.09%	2.46%	3.45%	74.87%	13.61%	11.52%	0.86
43d	1	0	217	0.00%	100.00%	96.77%	0.92%	2.30%	76.67%	14.29%	9.05%	0.92
43d	1	0	284	0.00%	100.00%	97.18%	1.76%	1.06%	76.81%	10.87%	12.32%	1.20
43d	10	0	191	0.00%	100.00%	96.86%	1.05%	2.09%	77.84%	11.35%	10.81%	0.81
43d	10	0	114	0.00%	100.00%	94.74%	1.75%	3.51%	74.07%	12.04%	13.89%	0.48
43d	10	0	293	0.34%	99.66%	97.26%	1.03%	1.71%	74.30%	10.92%	14.79%	1.23
43d	10	0	258	0.39%	99.61%	95.33%	1.56%	3.11%	74.69%	13.88%	11.43%	1.09
43d	5	0	195	0.51%	99.49%	95.36%	2.58%	2.06%	78.38%	11.89%	9.73%	0.82
43d	5	0	94	0.00%	100.00%	92.55%	2.13%	5.32%	68.97%	18.39%	12.64%	0.40
43d	5	0	190	0.00%	100.00%	97.89%	0.53%	1.58%	74.19%	15.59%	10.22%	0.80
43d	5	0	246	0.00%	100.00%	96.75%	2.03%	1.22%	72.27%	15.55%	12.18%	1.04
43d	1	6	170	5.29%	94.71%	96.89%	0.62%	2.48%	7.69%	71.79%	20.51%	0.79
43d	1	6	167	2.40%	97.60%	93.87%	0.00%	6.13%	12.42%	62.75%	24.84%	0.80
43d	1	6	205	5.37%	94.63%	91.24%	0.00%	8.76%	11.86%	68.93%	19.21%	0.95
43d	1	6	265	4.15%	95.85%	94.88%	0.00%	5.12%	15.77%	63.07%	21.16%	1.25
43d	10	6	167	13.17%	86.83%	69.66%	0.00%	30.34%	14.85%	58.42%	26.73%	0.71
43d	10	6	102	17.65%	82.35%	63.10%	0.00%	36.90%	7.55%	56.60%	35.85%	0.41

43d	10	6	241	3.32%	96.68%	77.25%	0.43%	22.32%	12.78%	67.22%	20.00%	1.14
43d	10	6	231	7.79%	92.21%	71.83%	0.94%	27.23%	6.54%	64.05%	29.41%	1.05
43d	5	6	163	6.75%	93.25%	86.18%	0.66%	13.16%	10.69%	58.02%	31.30%	0.75
43d	5	6	83	9.64%	90.36%	85.33%	0.00%	14.67%	7.81%	70.31%	21.88%	0.37
43d	5	6	168	5.36%	94.64%	86.16%	0.00%	13.84%	15.33%	64.23%	20.44%	0.78
43d	5	6	214	6.07%	93.93%	87.06%	0.00%	12.94%	12.57%	73.14%	14.29%	0.99
43d	1	12	187	2.14%	97.86%	93.99%	0.00%	6.01%	4.65%	80.81%	14.53%	0.85
43d	1	12	171	2.34%	97.66%	91.62%	0.00%	8.38%	9.15%	77.12%	13.73%	0.78
43d	1	12	210	2.86%	97.14%	92.65%	0.00%	7.35%	13.23%	70.90%	15.87%	0.95
43d	1	12	266	4.89%	95.11%	94.07%	0.00%	5.93%	13.03%	72.27%	14.71%	1.17
43d	10	12	164	12.80%	87.20%	59.44%	0.00%	40.56%	11.76%	60.00%	28.24%	0.66
43d	10	12	112	8.93%	91.07%	56.86%	0.00%	43.14%	8.62%	62.07%	29.31%	0.47
43d	10	12	251	6.37%	93.63%	75.74%	0.43%	23.83%	15.73%	66.29%	17.98%	1.09
43d	10	12	239	13.39%	86.61%	70.53%	0.48%	28.99%	10.96%	61.64%	27.40%	0.96
43d	5	12	172	6.98%	93.02%	89.38%	0.00%	10.63%	12.59%	71.33%	16.08%	0.74
43d	5	12	86	8.14%	91.86%	79.75%	0.00%	20.25%	15.87%	76.19%	7.94%	0.37
43d	5	12	176	4.55%	95.45%	84.52%	0.00%	15.48%	12.68%	62.68%	24.65%	0.78
43d	5	12	215	5.12%	94.88%	84.31%	0.00%	15.69%	14.53%	66.28%	19.19%	0.95
43d	1	24	189	3.70%	96.30%	89.01%	0.00%	10.99%	22.84%	65.43%	11.73%	0.77
43d	1	24	193	2.59%	97.41%	88.30%	0.00%	11.70%	22.89%	67.47%	9.64%	0.79
43d	1	24	224	2.68%	97.32%	88.99%	0.00%	11.01%	21.13%	68.04%	10.82%	0.92
43d	1	24	280	6.07%	93.93%	90.87%	0.00%	9.13%	20.92%	69.04%	10.04%	1.11
43d	10	24	170	10.59%	89.41%	50.66%	0.00%	49.34%	10.39%	61.04%	28.57%	0.64
43d	10	24	110	10.91%	89.09%	44.90%	0.00%	55.10%	2.27%	68.18%	29.55%	0.41
43d	10	24	264	9.47%	90.53%	56.90%	0.00%	43.10%	13.24%	62.50%	24.26%	1.01
43d	10	24	245	10.20%	89.80%	54.09%	0.00%	45.91%	13.45%	60.50%	26.05%	0.93
43d	5	24	187	5.35%	94.65%	81.36%	0.00%	18.64%	20.14%	70.14%	9.72%	0.74
43d	5	24	92	7.61%	92.39%	78.82%	0.00%	21.18%	20.90%	73.13%	5.97%	0.36
43d	5	24	184	5.98%	94.02%	85.55%	0.00%	14.45%	22.97%	64.86%	12.16%	0.73
43d	5	24	238	5.46%	94.54%	77.78%	0.00%	22.22%	20.00%	73.71%	6.29%	0.95
43d	1	48	214	1.87%	98.13%	93.81%	0.00%	6.19%	57.87%	32.99%	9.14%	0.69
43d	1	48	202	3.47%	96.53%	88.72%	0.00%	11.28%	47.98%	37.57%	14.45%	0.64
43d	1	48	244	1.64%	98.36%	91.25%	0.00%	8.75%	62.10%	29.68%	8.22%	0.79
43d	1	48	297	1.68%	98.32%	92.81%	0.00%	7.19%	59.04%	32.10%	8.86%	0.96
43d	10	48	171	14.62%	85.38%	36.99%	0.00%	63.01%	11.11%	62.96%	25.93%	0.48
43d	10	48	108	12.96%	87.04%	36.17%	0.00%	63.83%	0.00%	85.29%	14.71%	0.31

43d	10	48	254	7.87%	92.13%	41.88%	0.00%	58.12%	20.41%	54.08%	25.51%	0.77
43d	10	48	228	10.53%	89.47%	46.08%	0.00%	53.92%	9.57%	63.83%	26.60%	0.67
43d	5	48	188	6.38%	93.62%	76.14%	1.14%	22.73%	40.30%	45.52%	14.18%	0.58
43d	5	48	91	3.30%	96.70%	71.59%	0.00%	28.41%	46.03%	39.68%	14.29%	0.29
43d	5	48	175	4.57%	95.43%	73.65%	0.00%	26.35%	39.02%	40.65%	20.33%	0.55
43d	5	48	244	6.56%	93.44%	85.09%	0.00%	14.91%	40.72%	43.81%	15.46%	0.75
43d	1	72	211	1.42%	98.58%	97.12%	0.00%	2.88%	67.82%	18.81%	13.37%	0.40
43d	1	72	203	0.99%	99.01%	94.53%	0.00%	5.47%	61.58%	27.37%	11.05%	0.39
43d	1	72	257	1.17%	98.83%	96.06%	0.00%	3.94%	70.08%	18.03%	11.89%	0.49
43d	1	72	312	1.28%	98.72%	96.43%	0.00%	3.57%	65.99%	20.88%	13.13%	0.60
43d	10	72	160	21.25%	78.75%	30.16%	0.00%	69.84%	5.26%	73.68%	21.05%	0.24
43d	10	72	109	19.27%	80.73%	35.23%	0.00%	64.77%	0.00%	90.32%	9.68%	0.17
43d	10	72	256	16.80%	83.20%	39.44%	0.00%	60.56%	10.71%	63.10%	26.19%	0.41
43d	10	72	229	11.35%	88.65%	45.81%	0.00%	54.19%	4.30%	61.29%	34.41%	0.39
43d	5	72	196	7.65%	92.35%	73.48%	0.00%	26.52%	43.61%	37.59%	18.80%	0.35
43d	5	72	92	7.61%	92.39%	65.88%	0.00%	34.12%	48.21%	39.29%	12.50%	0.17
43d	5	72	178	6.74%	93.26%	81.33%	0.00%	18.67%	48.15%	37.04%	14.81%	0.32
43d	5	72	230	3.48%	96.52%	81.53%	0.00%	18.47%	43.65%	40.88%	15.47%	0.43
42d	1	0	260	0.00%	100.00%	96.54%	1.15%	2.31%	70.92%	17.13%	11.95%	1.10
42d	1	0	223	0.00%	100.00%	97.76%	1.79%	0.45%	70.18%	17.43%	12.39%	0.94
42d	1	0	197	0.00%	100.00%	93.91%	0.00%	6.09%	78.38%	12.43%	9.19%	0.83
42d	1	0	223	0.45%	99.55%	95.95%	1.35%	2.70%	73.71%	13.62%	12.68%	0.94
42d	10	0	243	0.00%	100.00%	96.30%	1.23%	2.47%	73.50%	16.24%	10.26%	1.03
42d	10	0	155	0.65%	99.35%	96.75%	0.65%	2.60%	71.81%	14.09%	14.09%	0.65
42d	10	0	234	0.00%	100.00%	95.73%	2.56%	1.71%	75.89%	13.84%	10.27%	0.99
42d	10	0	226	0.00%	100.00%	94.69%	2.65%	2.65%	71.50%	16.82%	11.68%	0.96
42d	5	0	190	0.00%	100.00%	96.32%	1.58%	2.11%	74.86%	13.66%	11.48%	0.80
42d	5	0	142	0.00%	100.00%	92.96%	3.52%	3.52%	76.52%	12.12%	11.36%	0.60
42d	5	0	229	0.00%	100.00%	96.94%	0.44%	2.62%	76.13%	12.16%	11.71%	0.97
42d	5	0	211	0.00%	100.00%	97.16%	1.42%	1.42%	77.07%	15.12%	7.80%	0.89
42d	1	6	230	3.04%	96.96%	91.93%	0.00%	8.07%	11.22%	65.85%	22.93%	1.09
42d	1	6	195	3.08%	96.92%	92.06%	0.00%	7.94%	7.47%	64.94%	27.59%	0.93
42d	1	6	178	2.81%	97.19%	93.64%	0.00%	6.36%	9.26%	72.84%	17.90%	0.85
42d	1	6	204	5.88%	94.12%	94.27%	0.00%	5.73%	7.18%	69.61%	23.20%	0.94
42d	10	6	198	12.12%	87.88%	72.99%	0.57%	26.44%	10.24%	64.57%	25.20%	0.85
42d	10	6	129	17.83%	82.17%	68.87%	0.94%	30.19%	5.48%	69.86%	24.66%	0.52

42d	10	6	198	11.11%	88.89%	74.43%	0.57%	25.00%	8.40%	71.76%	19.85%	0.86
42d	10	6	197	15.23%	84.77%	70.66%	0.00%	29.34%	11.02%	66.10%	22.88%	0.82
42d	5	6	151	9.93%	90.07%	84.56%	0.00%	15.44%	13.04%	66.09%	20.87%	0.67
42d	5	6	124	7.26%	92.74%	83.48%	0.00%	16.52%	10.42%	67.71%	21.88%	0.56
42d	5	6	200	8.00%	92.00%	86.41%	0.54%	13.04%	15.72%	59.75%	24.53%	0.90
42d	5	6	182	7.14%	92.86%	81.07%	0.00%	18.93%	8.03%	65.69%	26.28%	0.83
42d	1	12	245	1.63%	98.37%	90.87%	0.00%	9.13%	7.76%	79.45%	12.79%	1.12
42d	1	12	196	1.53%	98.47%	94.82%	0.00%	5.18%	8.20%	73.22%	18.58%	0.90
42d	1	12	195	1.03%	98.97%	94.82%	0.00%	5.18%	7.65%	80.33%	12.02%	0.90
42d	1	12	221	3.17%	96.83%	95.33%	0.00%	4.67%	9.80%	76.96%	13.24%	0.99
42d	10	12	209	11.00%	89.00%	70.97%	0.00%	29.03%	23.48%	59.85%	16.67%	0.86
42d	10	12	135	17.04%	82.96%	60.71%	0.00%	39.29%	14.71%	63.24%	22.06%	0.52
42d	10	12	201	7.46%	92.54%	70.43%	0.54%	29.03%	20.61%	61.83%	17.56%	0.86
42d	10	12	207	15.46%	84.54%	62.29%	0.57%	37.14%	13.76%	69.72%	16.51%	0.81
42d	5	12	157	9.55%	90.45%	82.39%	0.00%	17.61%	15.38%	69.23%	15.38%	0.66
42d	5	12	128	5.47%	94.53%	75.21%	0.00%	24.79%	16.48%	65.93%	17.58%	0.56
42d	5	12	210	4.29%	95.71%	85.07%	0.00%	14.93%	18.71%	64.91%	16.37%	0.93
42d	5	12	183	8.20%	91.80%	86.31%	0.00%	13.69%	15.86%	68.97%	15.17%	0.78
42d	1	24	264	3.79%	96.21%	90.55%	0.00%	9.45%	23.04%	65.65%	11.30%	1.07
42d	1	24	219	3.20%	96.80%	89.62%	0.00%	10.38%	17.89%	73.68%	8.42%	0.89
42d	1	24	200	2.50%	97.50%	90.26%	0.00%	9.74%	17.05%	69.32%	13.64%	0.82
42d	1	24	232	5.60%	94.40%	91.32%	0.00%	8.68%	26.50%	60.00%	13.50%	0.92
42d	10	24	213	12.21%	87.79%	57.75%	0.00%	42.25%	26.85%	56.48%	16.67%	0.79
42d	10	24	155	13.55%	86.45%	59.70%	0.00%	40.30%	23.75%	60.00%	16.25%	0.56
42d	10	24	207	8.70%	91.30%	65.61%	0.53%	33.86%	29.84%	54.84%	15.32%	0.79
42d	10	24	212	15.57%	84.43%	51.96%	0.00%	48.04%	16.13%	66.67%	17.20%	0.75
42d	5	24	162	9.26%	90.74%	80.27%	0.00%	19.73%	18.64%	73.73%	7.63%	0.62
42d	5	24	132	3.03%	96.97%	75.00%	0.00%	25.00%	19.79%	60.42%	19.79%	0.54
42d	5	24	221	4.52%	95.48%	84.36%	0.00%	15.64%	28.65%	58.99%	12.36%	0.89
42d	5	24	198	4.04%	95.96%	77.89%	0.00%	22.11%	18.92%	68.92%	12.16%	0.80
42d	1	48	320	1.25%	98.75%	90.82%	0.00%	9.18%	48.43%	37.63%	13.94%	1.03
42d	1	48	252	1.59%	98.41%	93.95%	0.00%	6.05%	51.50%	39.91%	8.58%	0.81
42d	1	48	222	0.90%	99.10%	92.27%	0.00%	7.73%	44.83%	41.38%	13.79%	0.72
42d	1	48	245	2.45%	97.55%	93.72%	0.00%	6.28%	59.82%	30.80%	9.38%	0.78
42d	10	48	209	13.88%	86.12%	46.11%	0.56%	53.33%	26.51%	54.22%	19.28%	0.59
42d	10	48	153	12.42%	87.58%	41.04%	0.00%	58.96%	27.27%	54.55%	18.18%	0.44

42d	10	48	209	11.96%	88.04%	57.61%	0.54%	41.85%	32.08%	49.06%	18.87%	0.60
42d	10	48	210	20.48%	79.52%	52.10%	0.00%	47.90%	24.14%	59.77%	16.09%	0.55
42d	5	48	174	9.77%	90.23%	80.25%	0.00%	19.75%	35.71%	54.76%	9.52%	0.51
42d	5	48	134	5.97%	94.03%	77.78%	0.00%	22.22%	27.55%	56.12%	16.33%	0.41
42d	5	48	224	5.36%	94.64%	77.36%	0.00%	22.64%	35.98%	48.78%	15.24%	0.69
42d	5	48	206	4.85%	95.15%	78.57%	0.00%	21.43%	37.01%	51.95%	11.04%	0.64
42d	1	72	357	0.84%	99.16%	97.18%	0.00%	2.82%	64.24%	19.48%	16.28%	0.69
42d	1	72	291	0.69%	99.31%	97.23%	0.00%	2.77%	66.19%	24.20%	9.61%	0.56
42d	1	72	271	1.85%	98.15%	95.49%	0.00%	4.51%	60.63%	23.23%	16.14%	0.52
42d	1	72	299	1.00%	99.00%	97.64%	0.00%	2.36%	66.09%	15.92%	17.99%	0.57
42d	10	72	202	14.85%	85.15%	36.63%	0.58%	62.79%	33.33%	47.62%	19.05%	0.33
42d	10	72	151	11.92%	88.08%	42.11%	0.00%	57.89%	21.43%	57.14%	21.43%	0.26
42d	10	72	200	9.00%	91.00%	56.04%	0.55%	43.41%	31.37%	47.06%	21.57%	0.35
42d	10	72	210	12.86%	87.14%	48.09%	0.00%	51.91%	21.59%	64.77%	13.64%	0.36
42d	5	72	180	5.56%	94.44%	81.18%	0.00%	18.82%	42.03%	47.10%	10.87%	0.33
42d	5	72	130	4.62%	95.38%	73.39%	0.00%	26.61%	37.36%	41.76%	20.88%	0.24
42d	5	72	223	3.59%	96.41%	80.93%	0.00%	19.07%	41.38%	43.10%	15.52%	0.42
42d	5	72	207	3.86%	96.14%	76.88%	0.00%	23.12%	48.37%	37.91%	13.73%	0.39
1	1	0	189	0.53%	99.47%	95.74%	1.60%	2.66%	75.56%	14.44%	10.00%	0.79
1	1	0	185	0.00%	100.00%	95.68%	3.24%	1.08%	75.71%	13.56%	10.73%	0.78
1	1	0	234	0.00%	100.00%	97.44%	0.43%	2.14%	74.12%	14.04%	11.84%	0.99
1	1	0	251	0.00%	100.00%	96.02%	1.59%	2.39%	73.44%	15.35%	11.20%	1.06
1	10	0	160	0.00%	100.00%	95.63%	1.25%	3.13%	72.55%	15.03%	12.42%	0.68
1	10	0	168	0.00%	100.00%	98.81%	0.60%	0.60%	75.90%	13.86%	10.24%	0.71
1	10	0	201	0.00%	100.00%	98.51%	0.50%	1.00%	70.20%	16.16%	13.64%	0.85
1	10	0	255	0.78%	99.22%	95.26%	4.35%	0.40%	73.44%	15.77%	10.79%	1.07
1	5	0	132	0.00%	100.00%	96.97%	2.27%	0.76%	75.78%	13.28%	10.94%	0.56
1	5	0	150	0.00%	100.00%	94.67%	3.33%	2.00%	66.20%	16.90%	16.90%	0.63
1	5	0	229	0.00%	100.00%	97.38%	1.31%	1.31%	77.58%	13.45%	8.97%	0.97
1	5	0	235	0.00%	100.00%	94.04%	2.13%	3.83%	74.66%	13.57%	11.76%	0.99
1	1	6	155	3.23%	96.77%	89.33%	0.00%	10.67%	17.91%	50.75%	31.34%	0.74
1	1	6	157	2.55%	97.45%	92.16%	0.00%	7.84%	17.73%	53.19%	29.08%	0.75
1	1	6	188	3.72%	96.28%	91.16%	0.55%	8.29%	23.03%	47.27%	29.70%	0.89
1	1	6	209	5.26%	94.74%	92.42%	0.00%	7.58%	18.03%	50.82%	31.15%	0.97
1	10	6	130	2.31%	97.69%	88.19%	0.00%	11.81%	14.29%	58.93%	26.79%	0.62
1	10	6	140	3.57%	96.43%	93.33%	0.00%	6.67%	15.87%	58.73%	25.40%	0.66

1	10	6	151	3.97%	96.03%	86.21%	0.00%	13.79%	16.80%	46.40%	36.80%	0.71
1	10	6	198	5.56%	94.44%	87.70%	0.00%	12.30%	12.20%	57.32%	30.49%	0.92
1	5	6	115	4.35%	95.65%	88.18%	0.91%	10.91%	18.56%	60.82%	20.62%	0.54
1	5	6	133	5.26%	94.74%	89.68%	0.00%	10.32%	11.50%	68.14%	20.35%	0.62
1	5	6	168	4.17%	95.83%	85.09%	0.00%	14.91%	12.41%	54.74%	32.85%	0.79
1	5	6	190	4.74%	95.26%	83.43%	0.55%	16.02%	19.87%	57.62%	22.52%	0.89
1	1	12	165	5.45%	94.55%	83.97%	0.00%	16.03%	45.04%	36.64%	18.32%	0.72
1	1	12	163	3.07%	96.93%	84.81%	0.00%	15.19%	36.57%	42.54%	20.90%	0.73
1	1	12	203	3.45%	96.55%	89.80%	0.00%	10.20%	46.02%	35.80%	18.18%	0.91
1	1	12	220	2.73%	97.27%	87.38%	0.00%	12.62%	41.71%	43.32%	14.97%	0.99
1	10	12	144	2.78%	97.22%	67.86%	0.00%	32.14%	27.37%	48.42%	24.21%	0.65
1	10	12	151	5.30%	94.70%	76.22%	0.00%	23.78%	30.28%	42.20%	27.52%	0.66
1	10	12	167	6.59%	93.41%	67.95%	0.00%	32.05%	32.08%	40.57%	27.36%	0.72
1	10	12	215	7.44%	92.56%	73.37%	0.00%	26.63%	34.93%	45.89%	19.18%	0.92
1	5	12	126	2.38%	97.62%	81.30%	0.81%	17.89%	38.00%	47.00%	15.00%	0.57
1	5	12	138	8.70%	91.30%	79.37%	0.00%	20.63%	33.00%	48.00%	19.00%	0.59
1	5	12	194	3.61%	96.39%	78.07%	0.00%	21.93%	39.04%	43.84%	17.12%	0.87
1	5	12	204	6.37%	93.63%	75.92%	0.00%	24.08%	40.69%	41.38%	17.93%	0.89
1	1	24	191	2.62%	97.38%	70.43%	0.00%	29.57%	51.91%	33.59%	14.50%	0.78
1	1	24	182	4.95%	95.05%	75.72%	0.00%	24.28%	50.38%	34.35%	15.27%	0.73
1	1	24	223	5.38%	94.62%	77.25%	0.00%	22.75%	64.42%	23.31%	12.27%	0.89
1	1	24	238	7.98%	92.02%	74.89%	0.00%	25.11%	53.05%	33.54%	13.41%	0.92
1	10	24	152	5.92%	94.08%	58.04%	0.00%	41.96%	40.96%	38.55%	20.48%	0.60
1	10	24	158	10.13%	89.87%	56.34%	0.00%	43.66%	45.00%	32.50%	22.50%	0.60
1	10	24	186	6.99%	93.01%	60.12%	0.00%	39.88%	50.00%	34.62%	15.38%	0.73
1	10	24	232	7.33%	92.67%	59.53%	0.00%	40.47%	46.09%	33.59%	20.31%	0.90
1	5	24	132	6.82%	93.18%	71.54%	0.00%	28.46%	42.05%	39.77%	18.18%	0.52
1	5	24	135	8.15%	91.85%	64.52%	0.00%	35.48%	50.00%	31.25%	18.75%	0.52
1	5	24	196	5.10%	94.90%	68.28%	0.00%	31.72%	48.03%	35.43%	16.54%	0.78
1	5	24	225	8.89%	91.11%	58.05%	0.00%	41.95%	43.70%	36.97%	19.33%	0.86
1	1	48	189	6.35%	93.65%	67.23%	0.00%	32.77%	54.62%	31.09%	14.29%	0.58
1	1	48	174	6.32%	93.68%	64.42%	0.00%	35.58%	48.57%	27.62%	23.81%	0.53
1	1	48	229	10.04%	89.96%	76.70%	0.00%	23.30%	52.53%	31.01%	16.46%	0.67
1	1	48	244	4.51%	95.49%	70.39%	0.00%	29.61%	50.61%	28.66%	20.73%	0.76
1	10	48	149	14.77%	85.23%	44.88%	0.00%	55.12%	47.37%	42.11%	10.53%	0.42
1	10	48	156	12.18%	87.82%	50.36%	0.00%	49.64%	39.13%	44.93%	15.94%	0.45

1	10	48	181	7.18%	92.82%	40.48%	0.00%	59.52%	42.65%	42.65%	14.71%	0.55
1	10	48	239	9.21%	90.79%	52.07%	0.00%	47.93%	40.71%	37.17%	22.12%	0.71
1	5	48	129	9.30%	90.70%	64.96%	0.00%	35.04%	43.42%	38.16%	18.42%	0.38
1	5	48	136	10.29%	89.71%	57.38%	0.82%	41.80%	47.14%	35.71%	17.14%	0.40
1	5	48	195	8.21%	91.79%	62.01%	0.00%	37.99%	51.35%	40.54%	8.11%	0.59
1	5	48	213	9.39%	90.61%	52.33%	0.52%	47.15%	38.61%	43.56%	17.82%	0.63
1	1	72	188	5.85%	94.15%	64.97%	0.00%	35.03%	51.30%	33.04%	15.65%	0.34
1	1	72	175	7.43%	92.57%	67.90%	0.00%	32.10%	60.00%	27.27%	12.73%	0.31
1	1	72	215	7.91%	92.09%	72.73%	0.00%	27.27%	55.56%	31.25%	13.19%	0.38
1	1	72	230	7.83%	92.17%	62.26%	0.00%	37.74%	48.48%	34.09%	17.42%	0.41
1	10	72	151	10.60%	89.40%	43.70%	0.00%	56.30%	28.81%	55.93%	15.25%	0.26
1	10	72	157	7.01%	92.99%	41.78%	0.00%	58.22%	37.70%	52.46%	9.84%	0.28
1	10	72	182	8.24%	91.76%	41.32%	0.00%	58.68%	43.48%	46.38%	10.14%	0.32
1	10	72	222	9.46%	90.54%	35.32%	0.00%	64.68%	39.44%	40.85%	19.72%	0.39
1	5	72	135	10.37%	89.63%	57.02%	0.00%	42.98%	39.13%	43.48%	17.39%	0.24
1	5	72	132	7.58%	92.42%	54.10%	0.82%	45.08%	50.00%	30.30%	19.70%	0.24
1	5	72	188	8.51%	91.49%	53.49%	0.00%	46.51%	48.91%	36.96%	14.13%	0.33
1	5	72	222	8.56%	91.44%	48.28%	0.00%	51.72%	31.63%	48.98%	19.39%	0.39
milciclib	1	0	201	0.00%	100.00%	96.02%	1.00%	2.99%	73.58%	15.54%	10.88%	0.85
milciclib	1	0	261	0.00%	100.00%	95.02%	2.30%	2.68%	76.61%	10.89%	12.50%	1.10
milciclib	1	0	302	0.00%	100.00%	96.03%	2.32%	1.66%	73.10%	15.86%	11.03%	1.28
milciclib	10	0	237	0.42%	99.58%	96.19%	1.27%	2.54%	76.65%	11.01%	12.33%	1.00
milciclib	10	0	297	0.00%	100.00%	94.28%	1.68%	4.04%	73.93%	14.64%	11.43%	1.26
milciclib	10	0	258	0.39%	99.61%	95.72%	2.33%	1.95%	71.14%	17.07%	11.79%	1.09
milciclib	1	6	181	2.21%	97.79%	92.66%	0.00%	7.34%	8.54%	73.78%	17.68%	0.87
milciclib	1	6	214	2.34%	97.66%	91.87%	0.48%	7.66%	7.81%	70.83%	21.35%	1.03
milciclib	1	6	251	2.39%	97.61%	92.65%	0.00%	7.35%	9.25%	83.26%	7.49%	1.20
milciclib	10	6	179	2.23%	97.77%	93.71%	0.00%	6.29%	10.98%	72.56%	16.46%	0.86
milciclib	10	6	233	3.00%	97.00%	88.94%	0.00%	11.06%	19.40%	68.66%	11.94%	1.11
milciclib	10	6	195	3.08%	96.92%	91.01%	0.53%	8.47%	20.93%	66.86%	12.21%	0.93
milciclib	1	12	191	3.14%	96.86%	92.97%	0.00%	7.03%	13.37%	75.00%	11.63%	0.86
milciclib	1	12	236	3.81%	96.19%	90.75%	0.00%	9.25%	12.14%	75.73%	12.14%	1.05
milciclib	1	12	267	1.12%	98.88%	90.15%	0.00%	9.85%	15.97%	75.63%	8.40%	1.23
milciclib	10	12	187	3.21%	96.79%	90.06%	0.00%	9.94%	38.04%	49.69%	12.27%	0.84
milciclib	10	12	226	3.10%	96.90%	84.02%	0.00%	15.98%	35.87%	51.63%	12.50%	1.02
milciclib	10	12	198	3.03%	96.97%	85.94%	0.00%	14.06%	39.39%	47.27%	13.33%	0.89

milciclib	1	24	183	4.37%	95.63%	88.57%	0.00%	11.43%	69.68%	26.45%	3.87%	0.74
milciclib	1	24	228	3.51%	96.49%	87.27%	0.00%	12.73%	65.10%	30.21%	4.69%	0.93
milciclib	1	24	254	2.76%	97.24%	89.88%	0.00%	10.12%	68.02%	27.48%	4.50%	1.04
milciclib	10	24	197	7.11%	92.89%	79.23%	0.00%	20.77%	58.62%	31.03%	10.34%	0.77
milciclib	10	24	231	3.03%	96.97%	71.88%	0.00%	28.13%	59.01%	32.30%	8.70%	0.94
milciclib	10	24	191	3.66%	96.34%	79.89%	0.54%	19.57%	59.86%	26.53%	13.61%	0.77
milciclib	1	48	160	0.63%	99.38%	89.94%	0.00%	10.06%	71.33%	19.58%	9.09%	0.52
milciclib	1	48	178	2.25%	97.75%	91.38%	0.57%	8.05%	76.73%	15.09%	8.18%	0.57
milciclib	1	48	184	2.72%	97.28%	94.41%	0.00%	5.59%	69.82%	21.89%	8.28%	0.59
milciclib	10	48	190	5.26%	94.74%	72.78%	0.00%	27.22%	60.31%	23.66%	16.03%	0.59
milciclib	10	48	231	6.93%	93.07%	74.88%	0.00%	25.12%	60.87%	21.74%	17.39%	0.70
milciclib	10	48	199	3.02%	96.98%	75.13%	0.52%	24.35%	65.52%	19.31%	15.17%	0.63
milciclib	1	72	150	1.33%	98.67%	92.57%	0.00%	7.43%	64.96%	26.28%	8.76%	0.29
milciclib	1	72	164	2.44%	97.56%	93.13%	0.00%	6.88%	65.77%	22.15%	12.08%	0.31
milciclib	1	72	170	0.59%	99.41%	97.63%	0.00%	2.37%	64.24%	24.24%	11.52%	0.33
milciclib	10	72	180	5.56%	94.44%	61.76%	0.00%	38.24%	54.29%	29.52%	16.19%	0.33
milciclib	10	72	244	6.15%	93.85%	67.69%	0.00%	32.31%	53.55%	29.68%	16.77%	0.44
milciclib	10	72	199	5.03%	94.97%	70.37%	0.00%	29.63%	52.63%	31.58%	15.79%	0.37

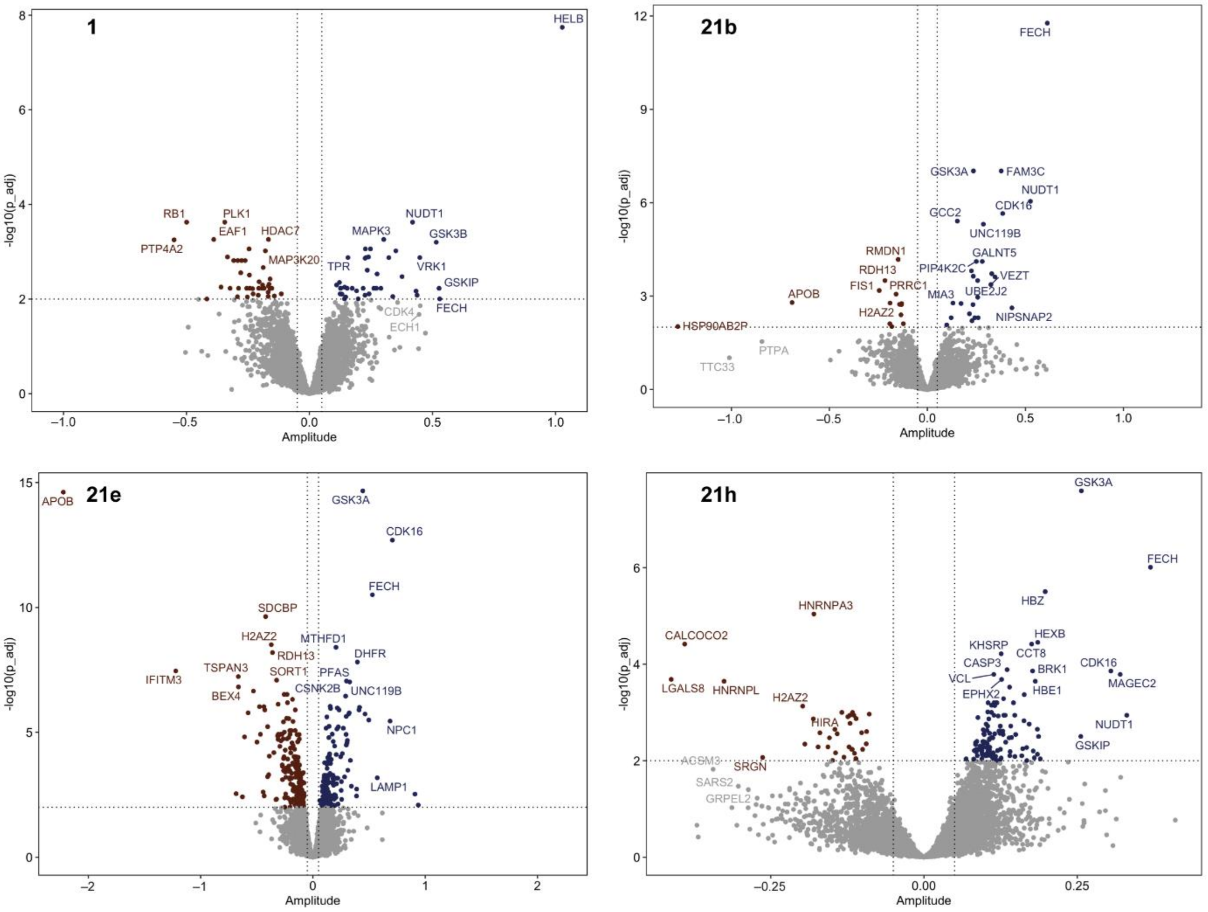

Figure S1: Vulcano plots of stabilized (positive amplitude) and destabilized (negative amplitude) proteins of **1**, **21b**, **21e**, and **21h**.

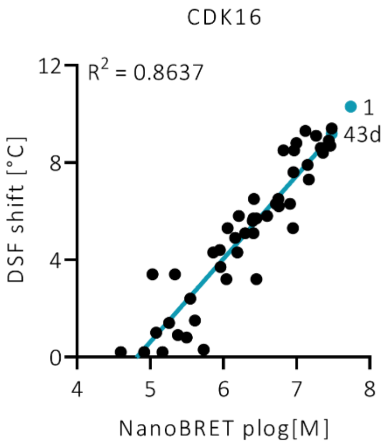

Figure S2: Correlation of DSF shifts with the accossiated NanoBRET data.

Analytical data of compounds **8 – 15, 18 – 25, 32 – 43**.

^{1}H and ^{13}C NMR data of compound **8.**

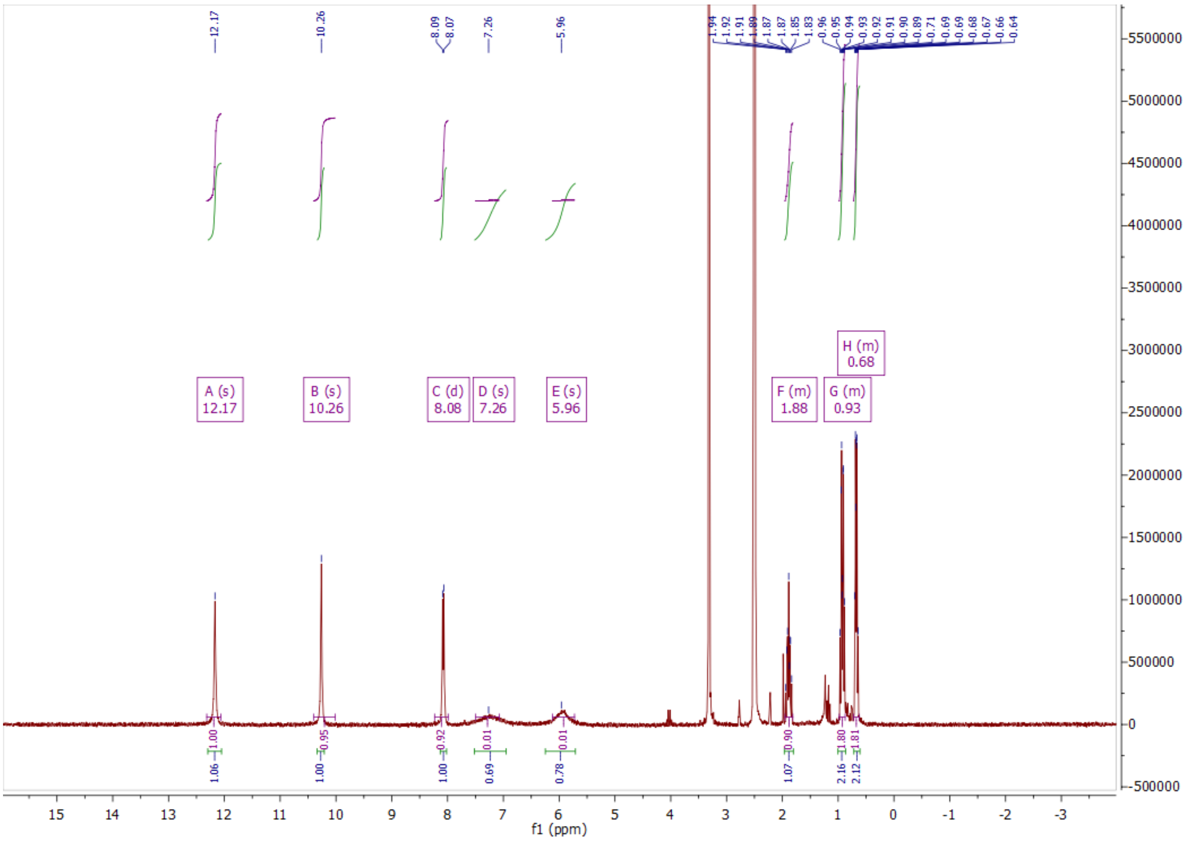

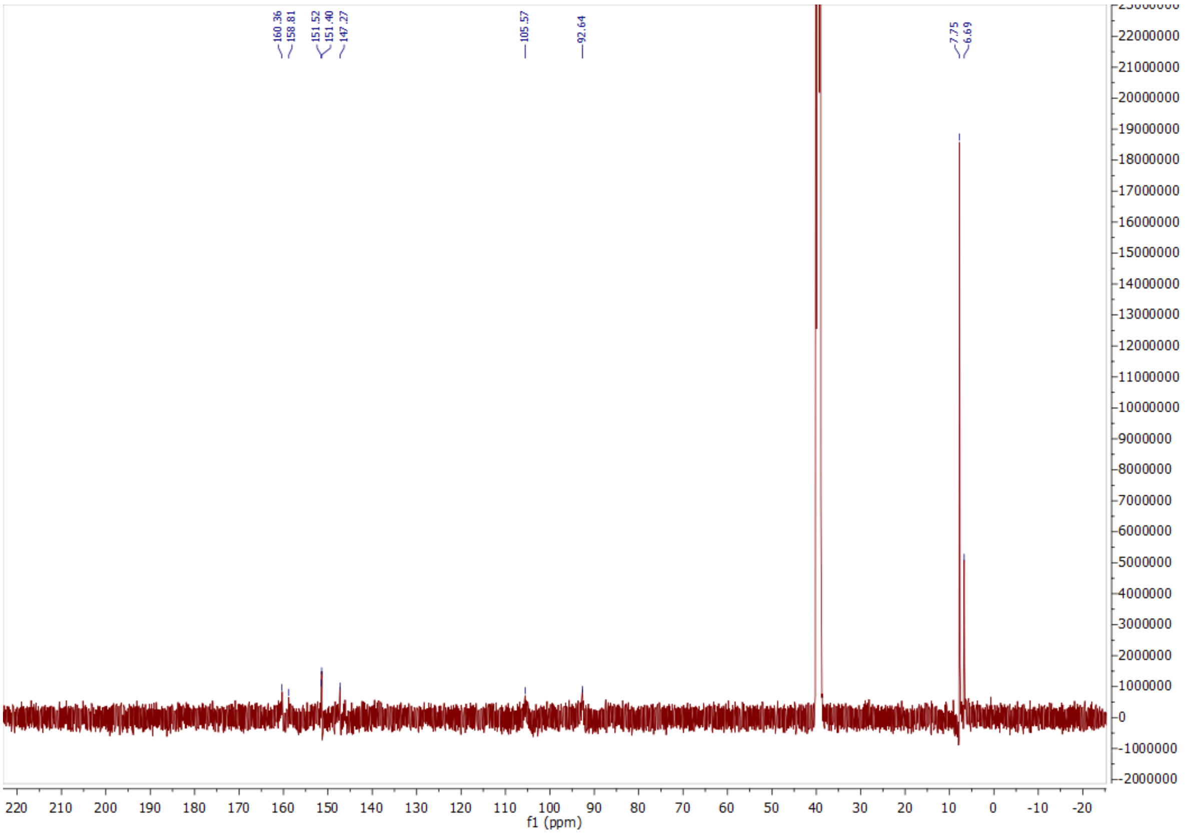

^{1}H and ^{13}C NMR data of compound **9**.

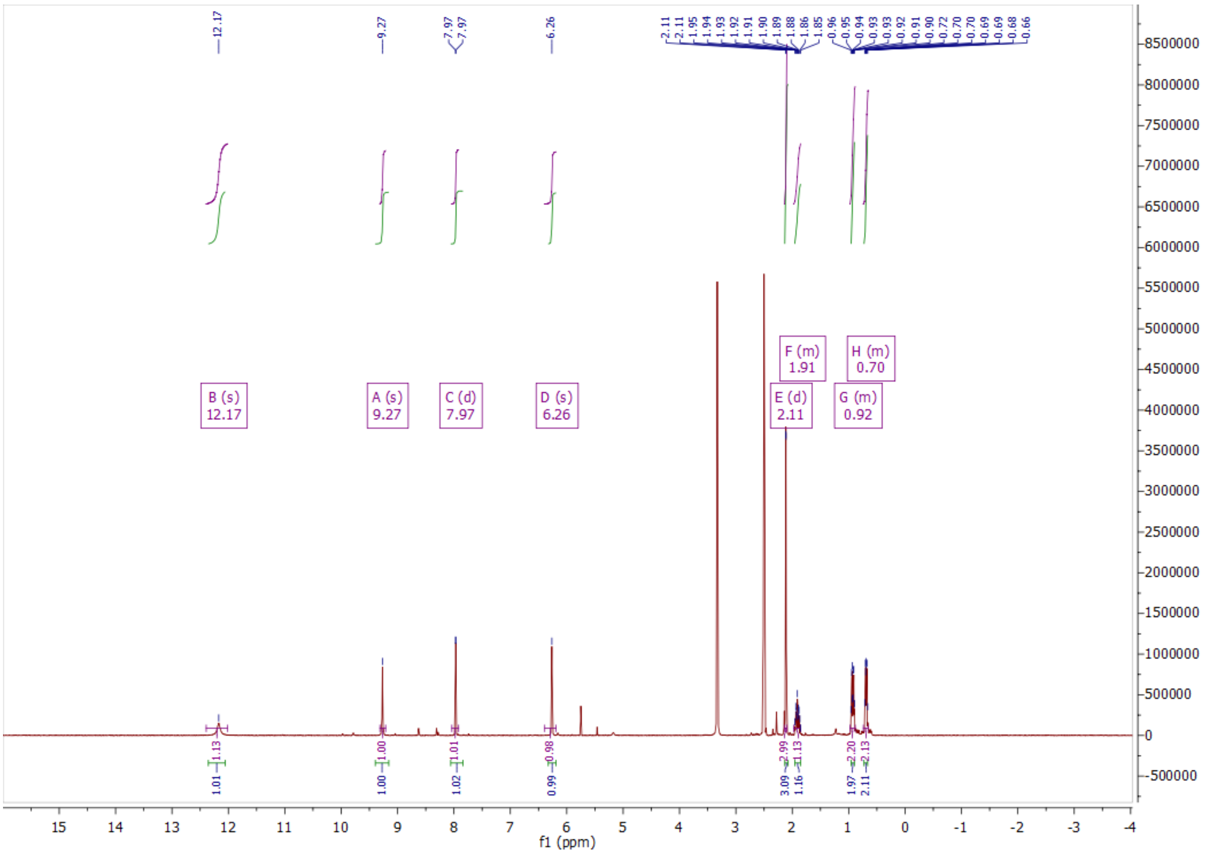

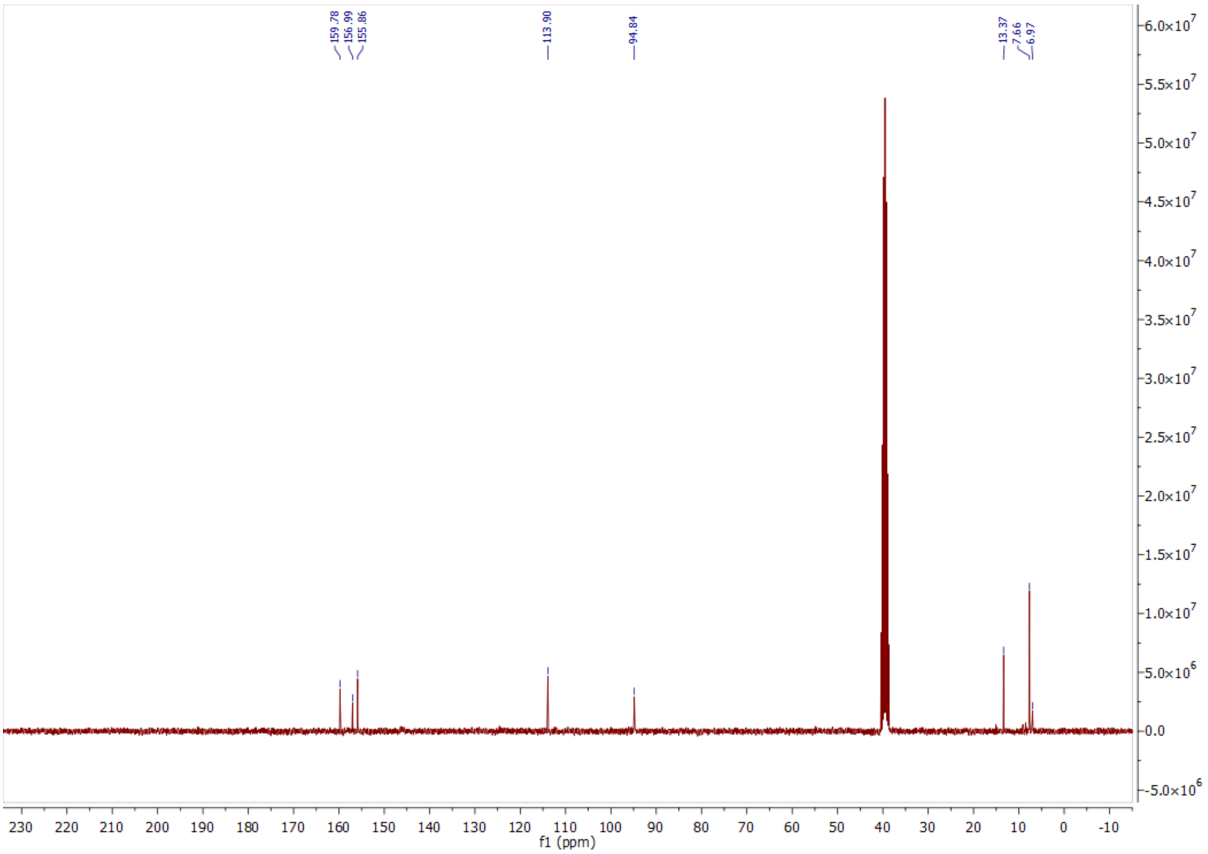

^{1}H and ^{13}C NMR data of compound **10**.

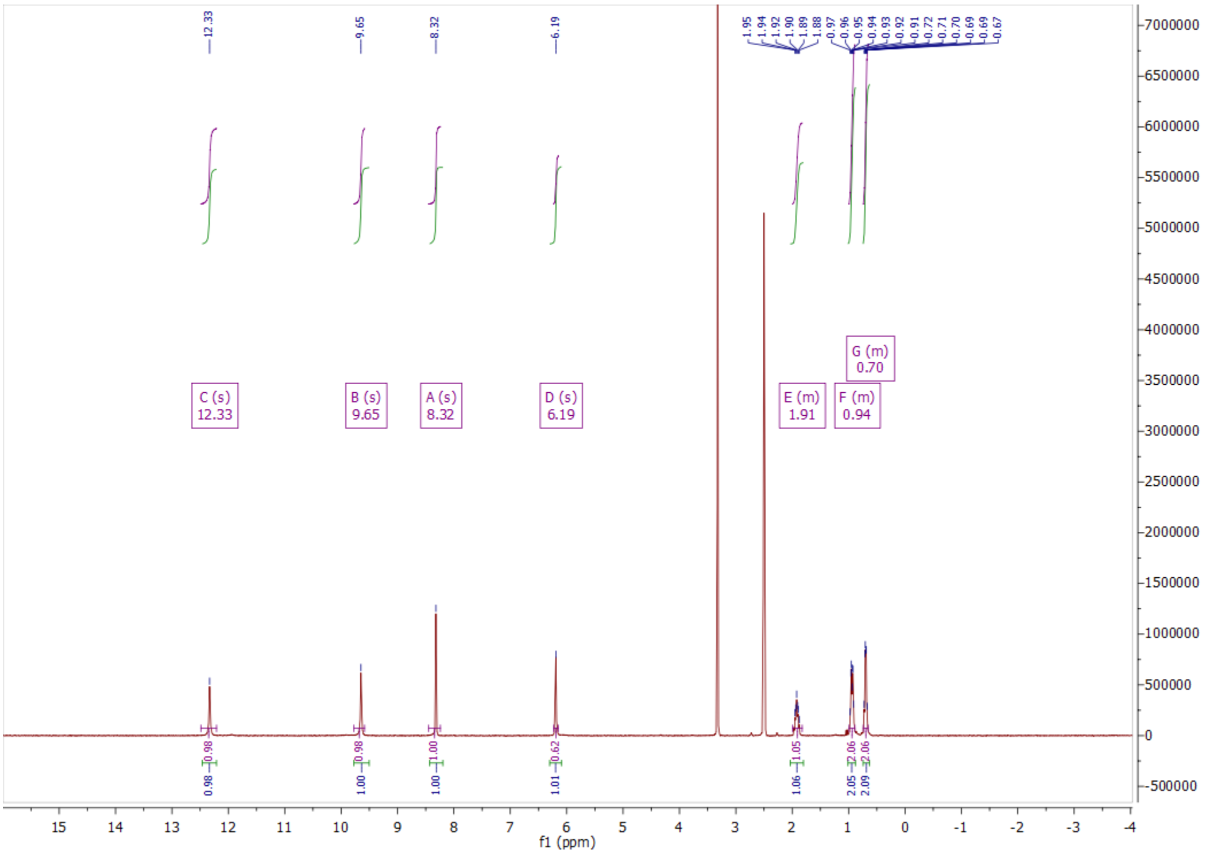

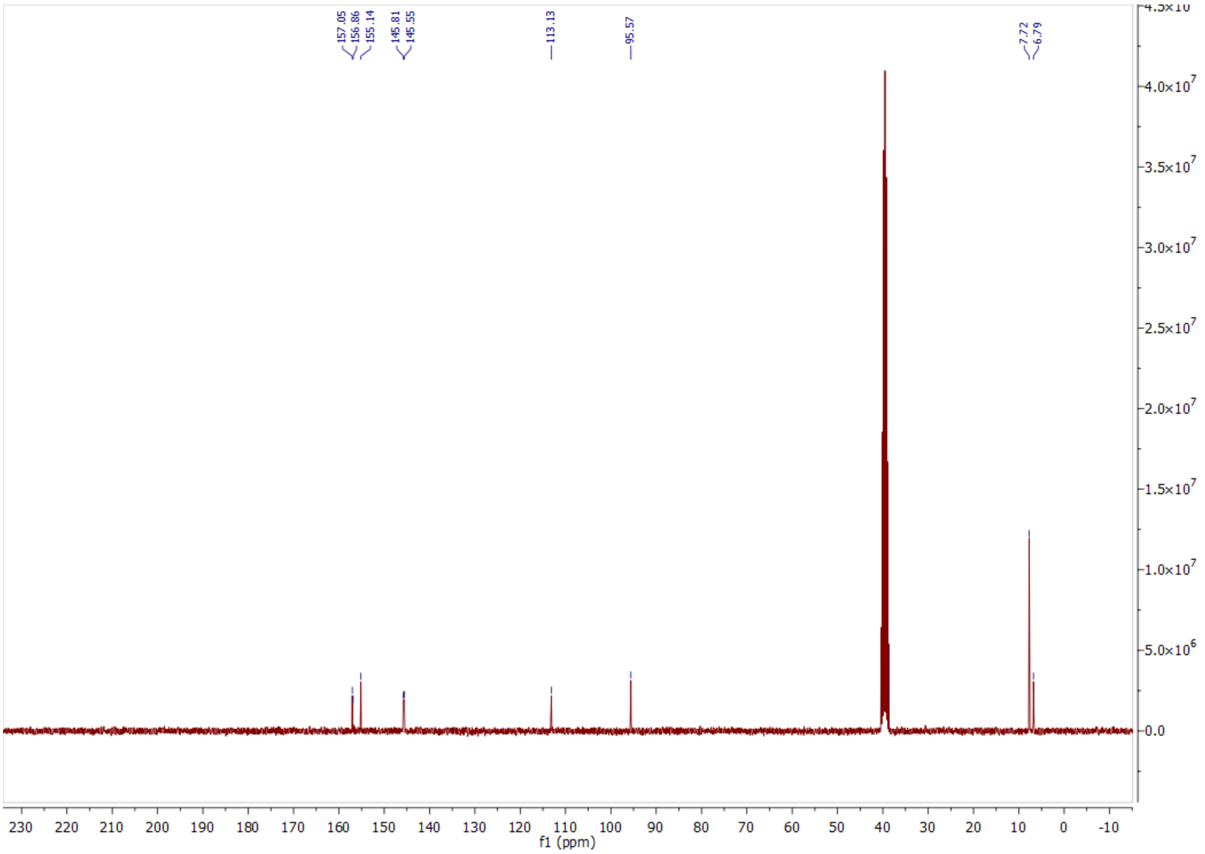

^{1}H, ^{13}C NMR and HPLC data of compound **11a**.

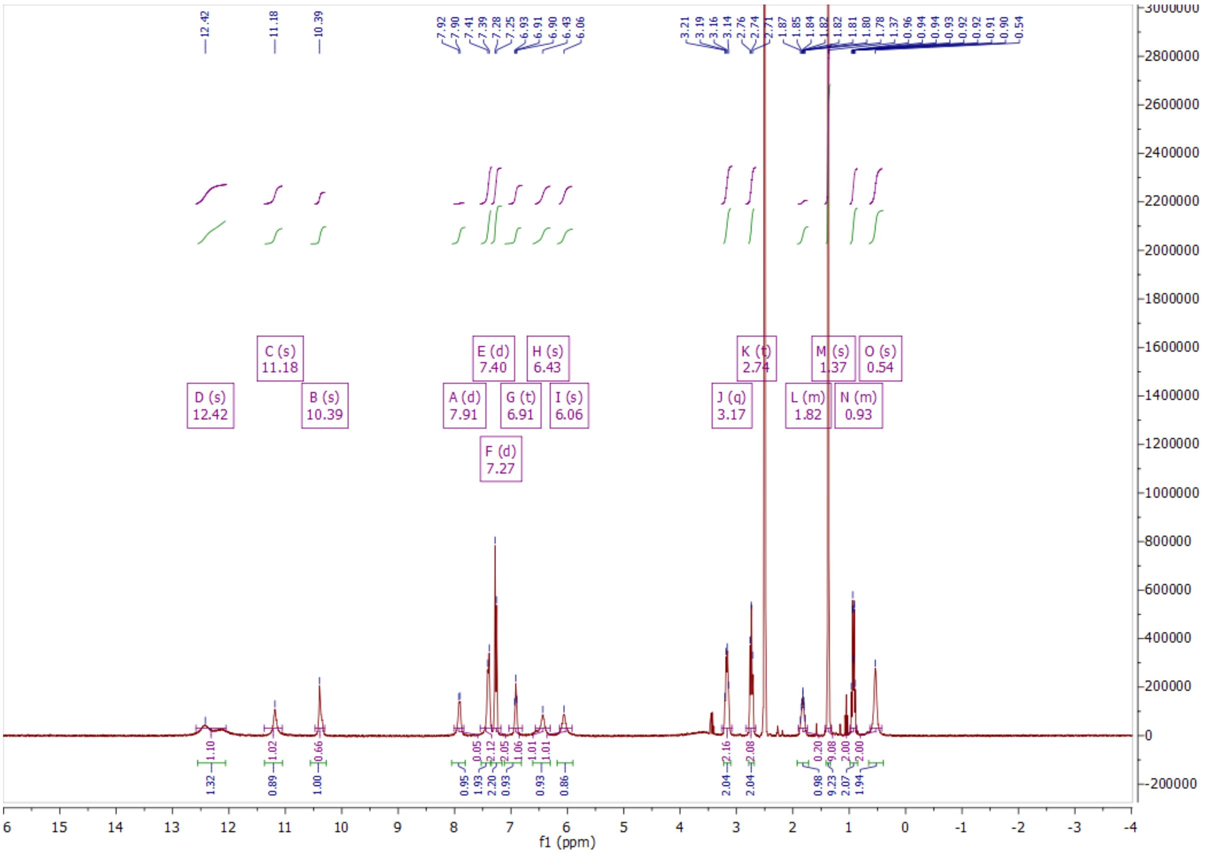

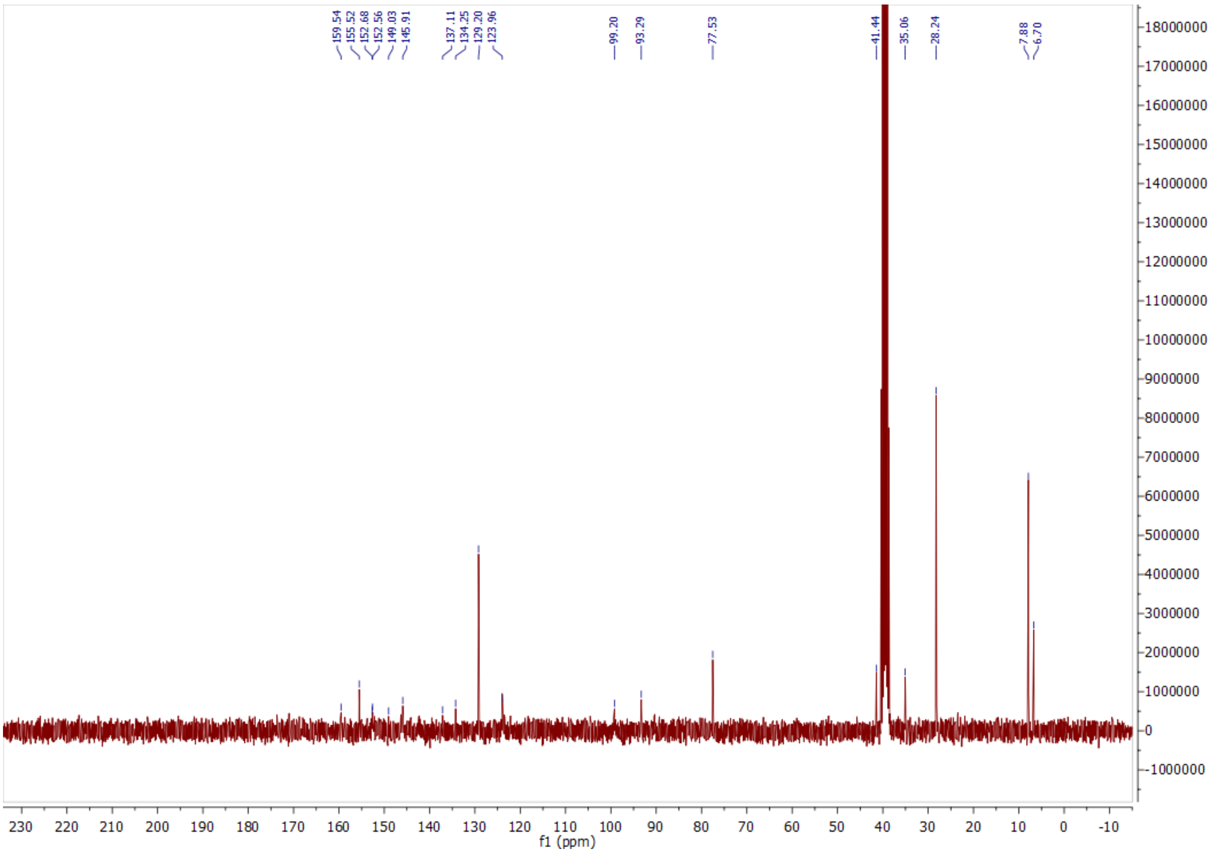

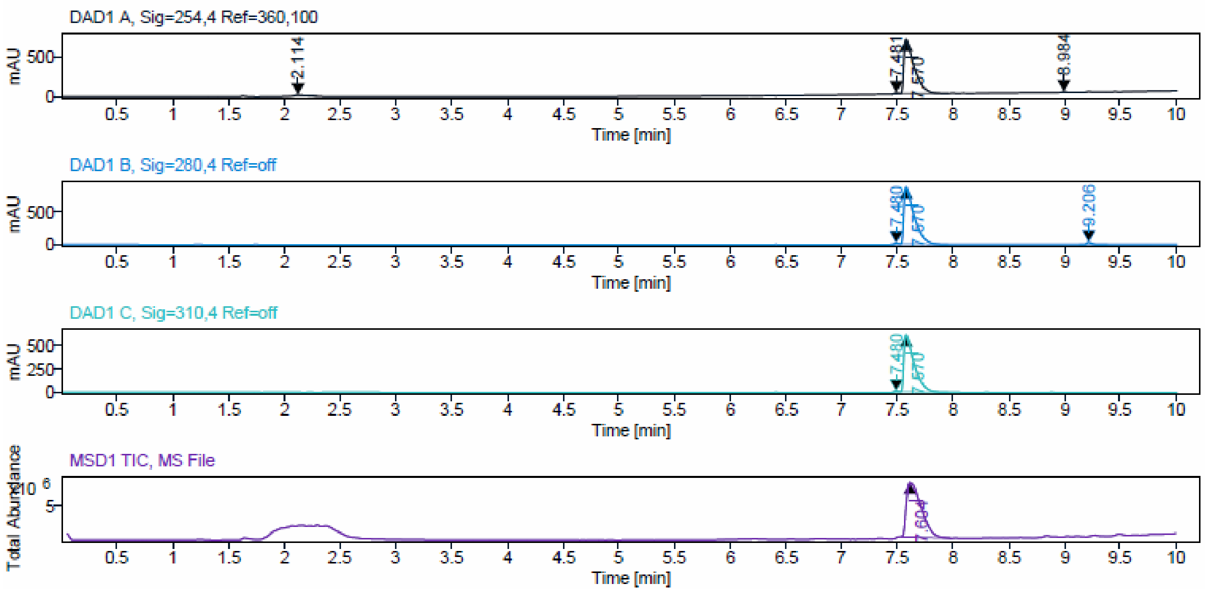

Sample Purity

Signal Description DAD1 A, Sig=254,4 Ref=360,100

Sample Name	Name	RT	Width	Area	Area%	Height
JA221_Feststoff		2.114	0.080	107.9313	2.33	14.3630
JA221_Feststoff		7.481	0.038	27.5142	0.59	12.3713
JA221_Feststoff		7.570	0.093	4476.0967	96.52	709.4230
JA221_Feststoff		8.984	0.040	26.0634	0.56	11.3094

Max Area% 96.517

UV Signal Purity>95% Pass

Signal Description DAD1 B, Sig=280,4 Ref=off

Sample Name	Name	RT	Width	Area	Area%	Height
JA221_Feststoff		7.480	0.037	34.6017	0.59	15.6127
JA221_Feststoff		7.570	0.093	5691.2979	97.50	897.4998
JA221_Feststoff		9.206	0.042	111.6275	1.91	36.8102

Max Area% 97.495

UV Signal Purity>95% Pass

Signal Description DAD1 C, Sig=310,4 Ref=off

Sample Name	Name	RT	Width	Area	Area%	Height
JA221_Feststoff		7.480	0.036	21.6304	0.54	9.8318
JA221_Feststoff		7.570	0.093	3963.1770	99.46	623.3157

Max Area% 99.457

UV Signal Purity>95% Pass

^{1}H, ^{13}C NMR and HPLC data of compound **11b**.

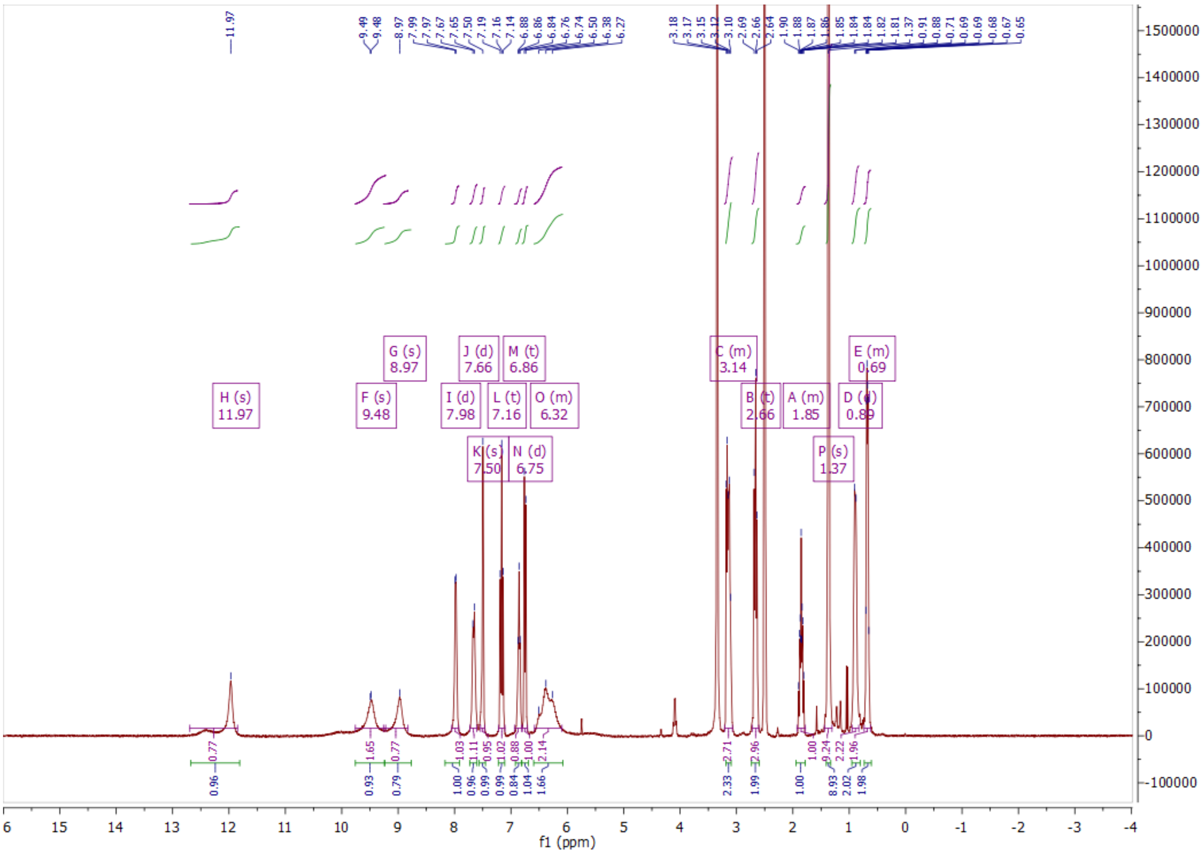

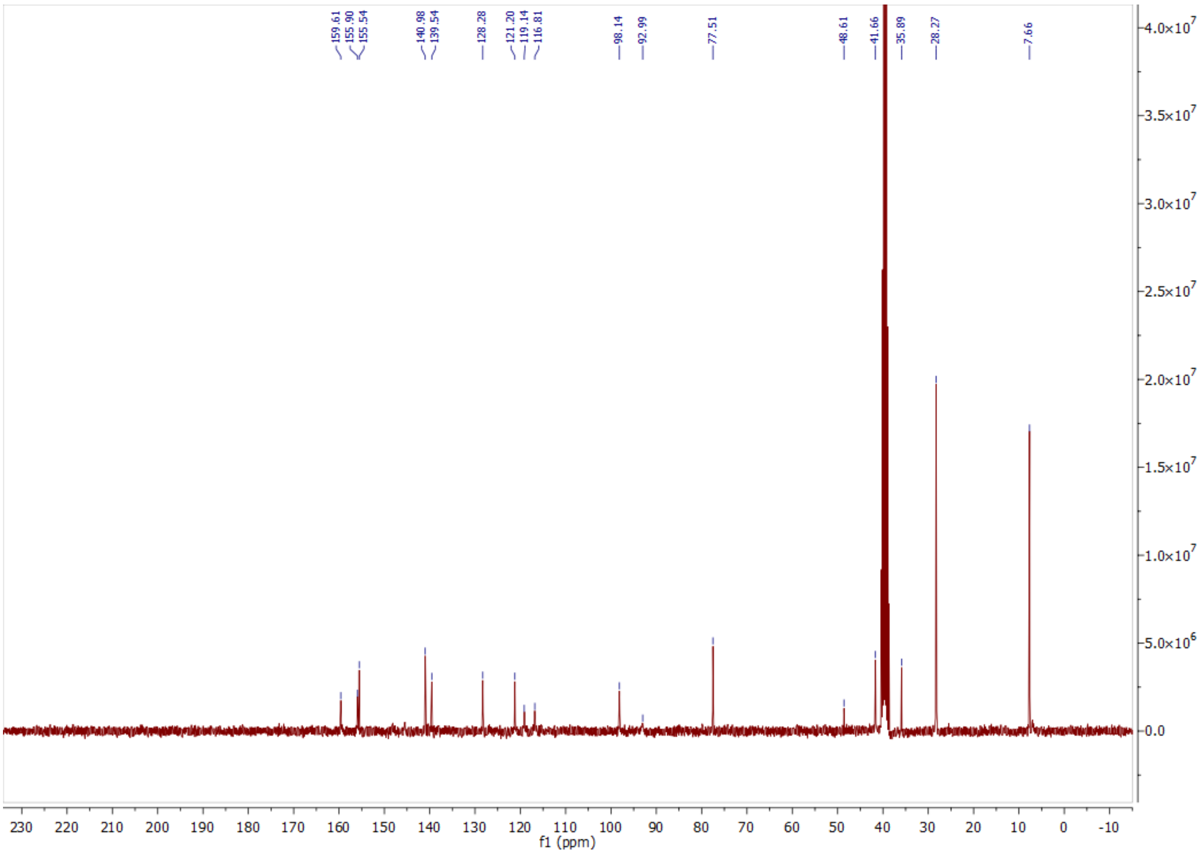

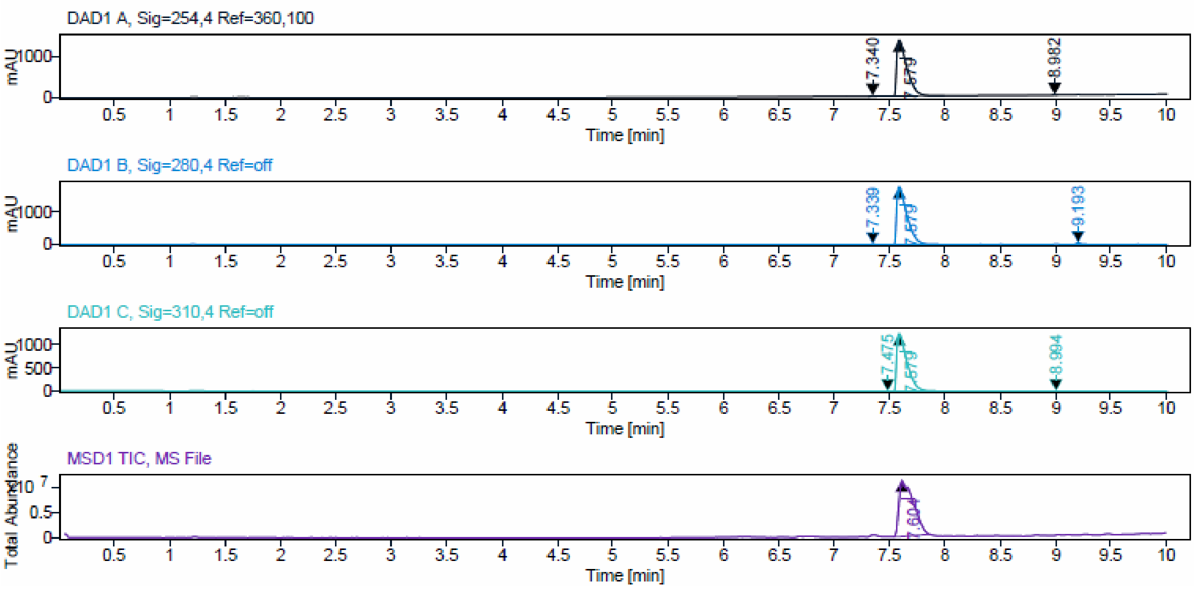

Sample Purity

Signal Description DAD1 A, Sig=254,4 Ref=360,100

Sample Name	Name	RT	Width	Area	Area%	Height
JA227_F6-8		7.340	0.033	35.2133	0.41	14.9857
JA227_F6-8		7.579	0.095	8617.5693	99.29	1378.2892
JA227_F6-8		8.982	0.042	26.8322	0.31	13.1462

Max Area% 99.285

UV Signal Purity>95% Pass

Signal Description DAD1 B, Sig=280,4 Ref=off

Sample Name	Name	RT	Width	Area	Area%	Height
JA227_F6-8		7.339	0.032	22.3977	0.20	9.0327
JA227_F6-8		7.579	0.095	11093.67 97	98.65	1769.9623
JA227_F6-8		9.193	0.040	129.6207	1.15	42.8550

Max Area% 98.648

UV Signal Purity>95% Pass

Signal Description DAD1 C, Sig=310,4 Ref=off

Sample Name	Name	RT	Width	Area	Area%	Height
JA227_F6-8		7.475	0.033	5.2779	0.07	2.5973
JA227_F6-8		7.579	0.094	7957.2271	99.86	1267.8508
JA227_F6-8		8.994	0.038	5.5085	0.07	2.0620

Max Area% 99.865

UV Signal Purity>95% Pass

^{1}H, ^{13}C NMR and HPLC data of compound **11c**.

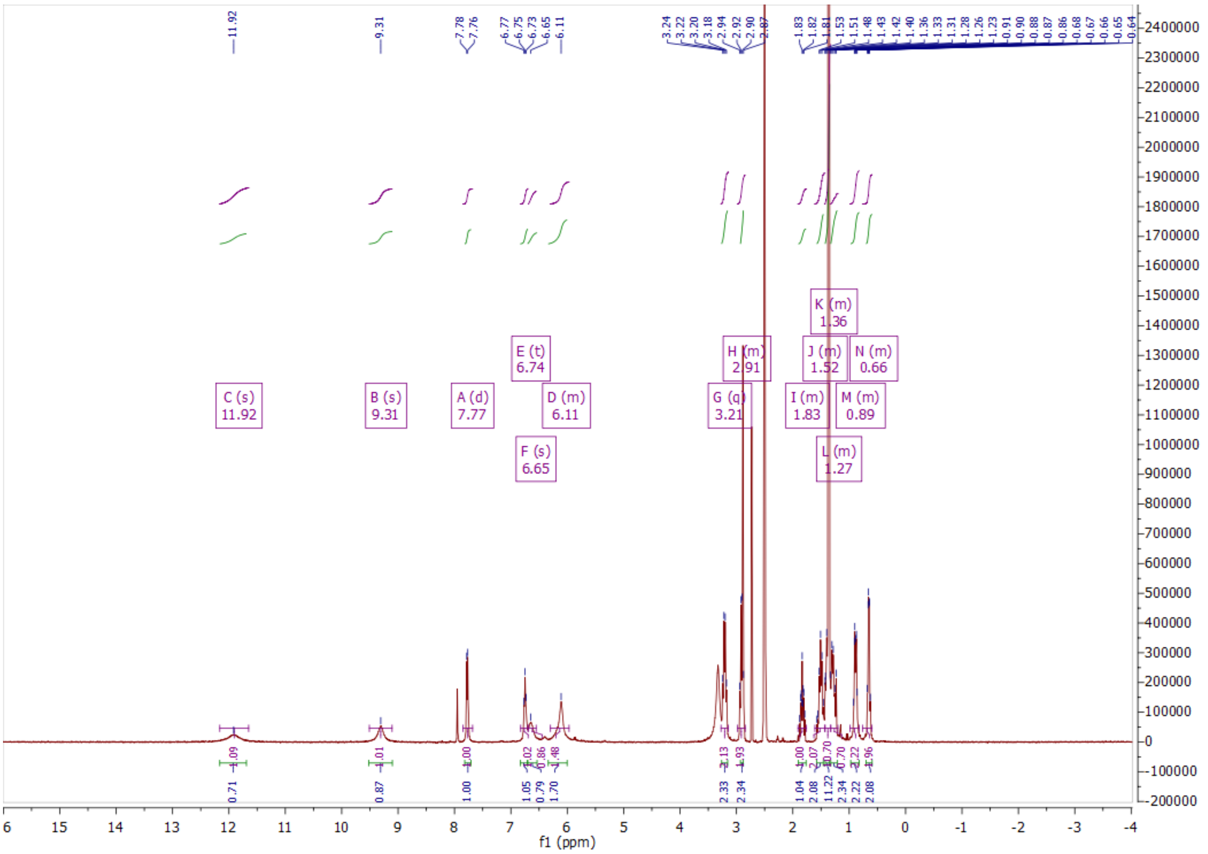

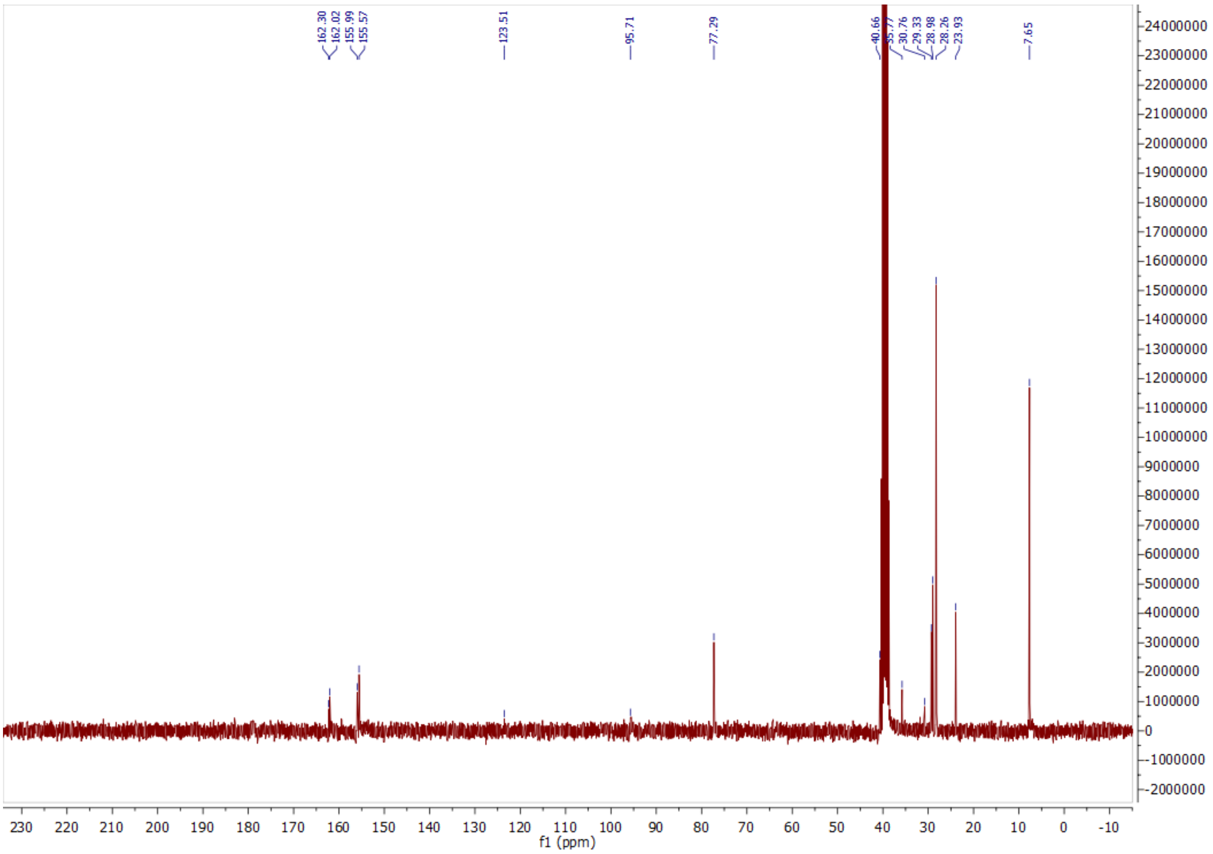

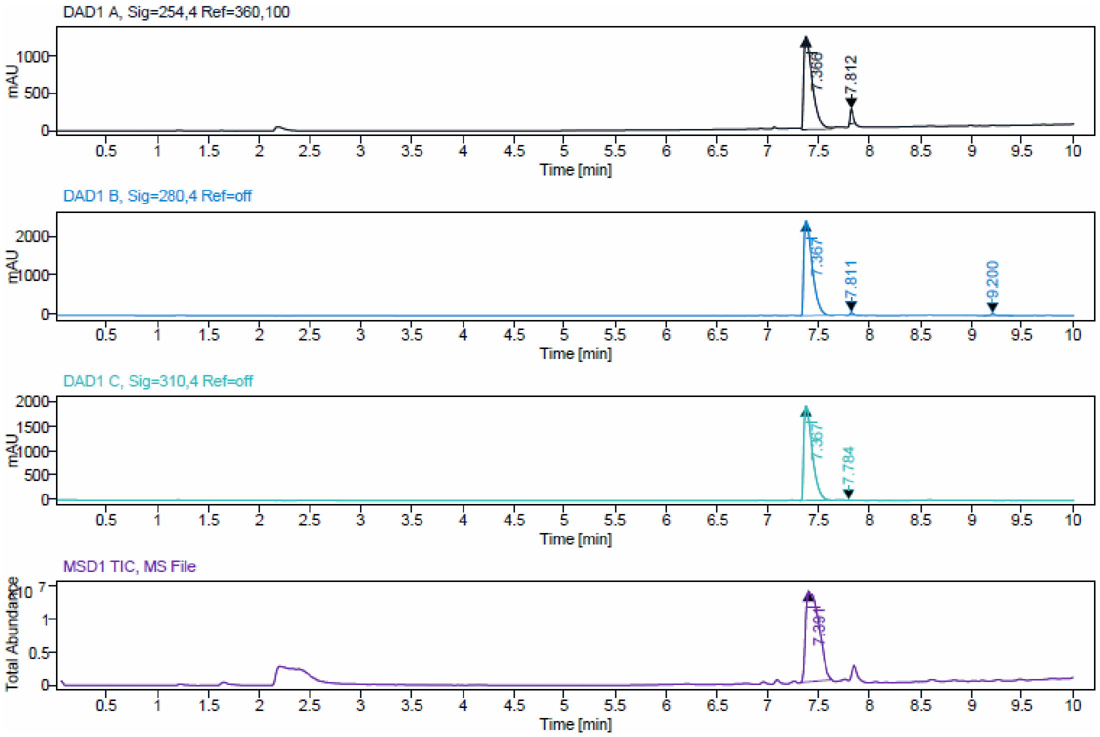

Sample Purity

Signal Description DAD1 A, Sig=254,4 Ref=360,100

Sample Name	Name	RT	Width	Area	Area%	Height
JA223_F6-11		7.366	0.089	7414.4194	95.10	1265.1262
JA223_F6-11		7.812	0.032	381.7301	4.90	198.3908

Max Area% 95.104

UV Signal Purity>95% Pass

Signal Description DAD1 B, Sig=280,4 Ref=off

Sample Name	Name	RT	Width	Area	Area%	Height
JA223_F6-11		7.367	0.089	13837.0684	97.76	2397.6631
JA223_F6-11		7.811	0.036	180.3365	1.27	71.7074
JA223_F6-11		9.200	0.040	136.9513	0.97	46.3551

Max Area% 97.758

UV Signal Purity>95% Pass

Signal Description DAD1 C, Sig=310,4 Ref=off

Sample Name	Name	RT	Width	Area	Area%	Height
JA223_F6-11		7.367	0.089	11148.0615	99.95	1938.9113
JA223_F6-11		7.784	0.042	6.0632	0.05	2.0418

Max Area% 99.946

UV Signal Purity>95% Pass

^{1}H, ^{13}C NMR and HPLC data of compound **11d**.

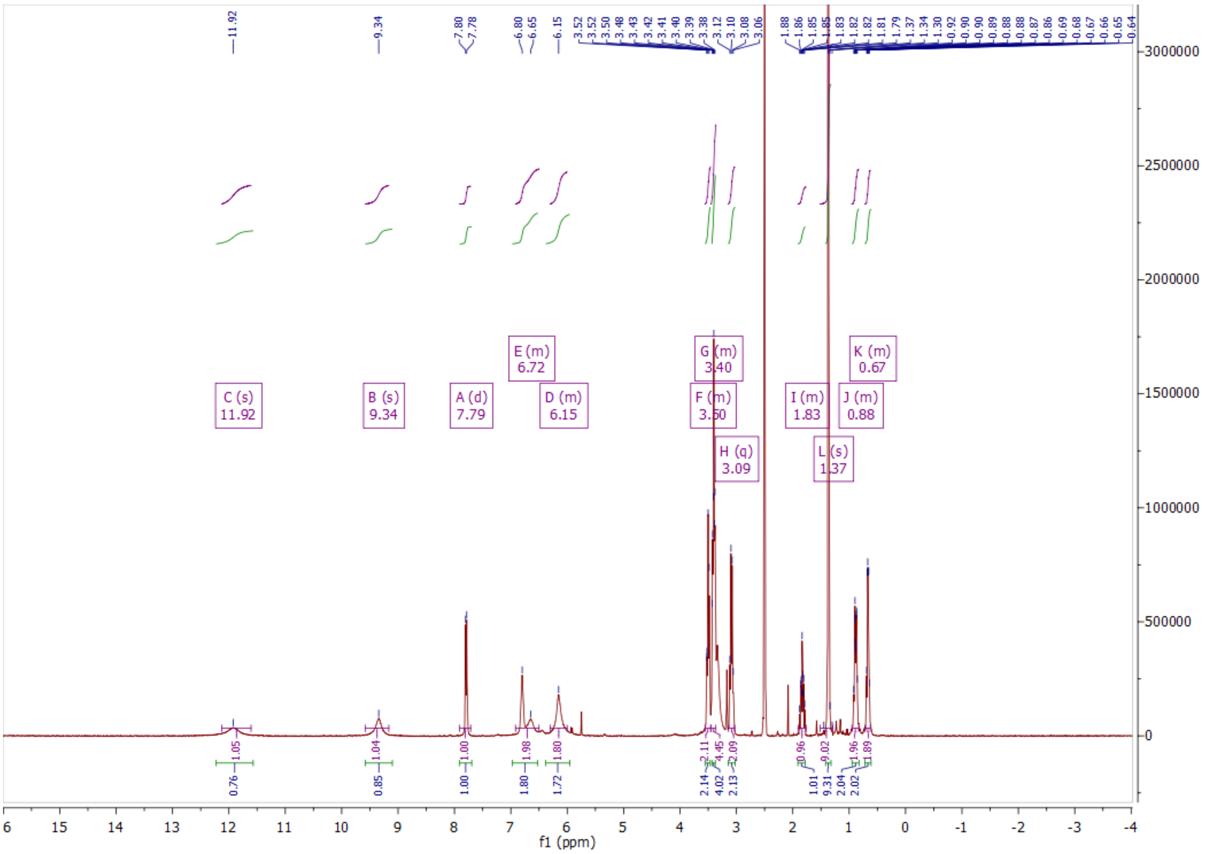

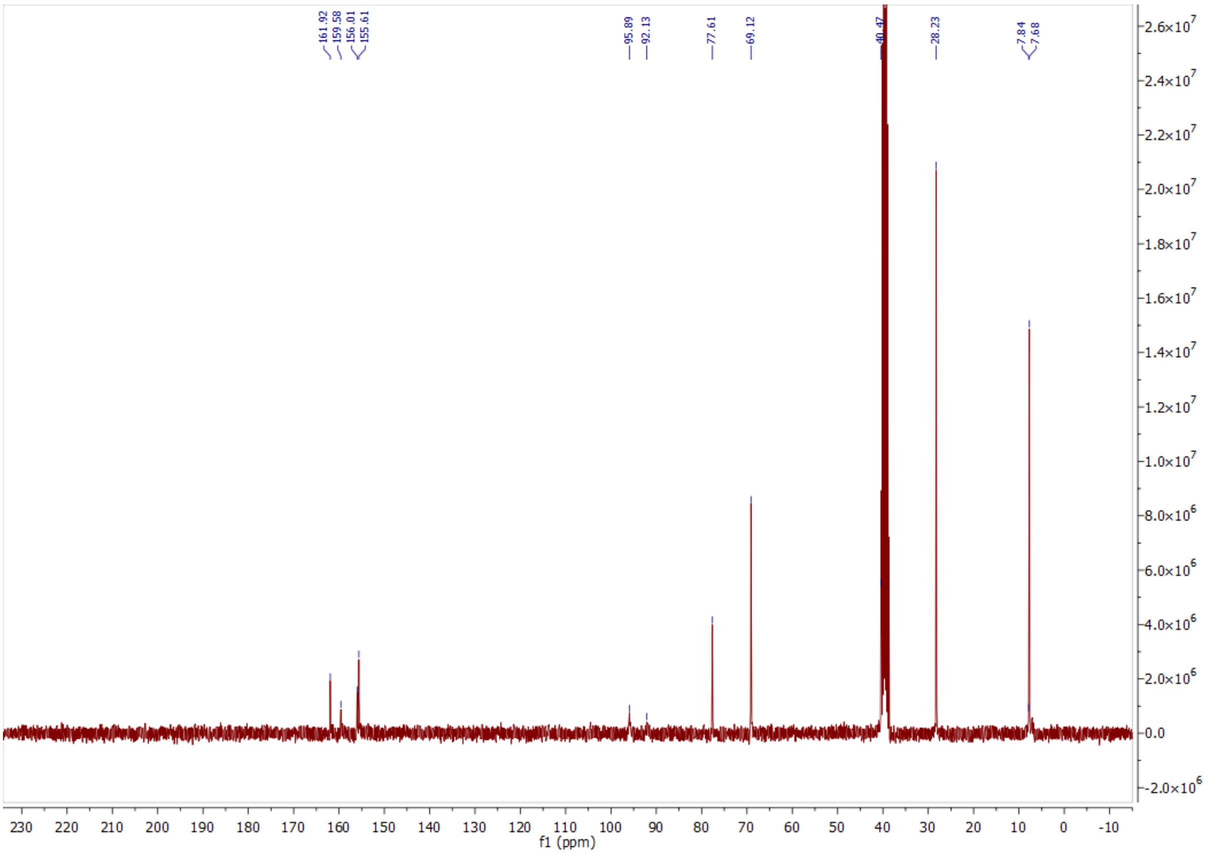

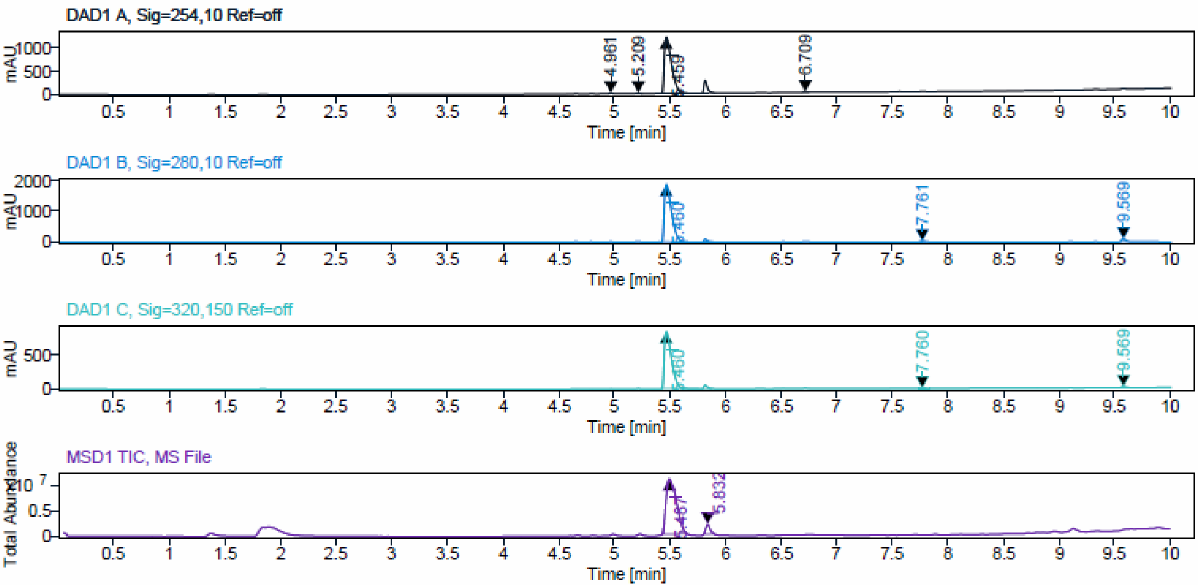

Sample Purity

Signal Description DAD1 A, Sig=254,10 Ref=off

Sample Name	Name	RT	Width	Area	Area%	Height
JA225		4.961	0.022	20.4267	0.33	15.1634
JA225		5.209	0.037	27.2926	0.44	14.6227
JA225		5.459	0.072	6123.4048	98.64	1226.3052
JA225		6.709	0.054	36.7559	0.59	14.5543

Max Area% 98.639

UV Signal Purity>95% Pass

Signal Description DAD1 B, Sig=280,10 Ref=off

Sample Name	Name	RT	Width	Area	Area%	Height
JA225		5.460	0.071	8898.8682	97.74	1913.5195
JA225		7.761	0.039	59.7091	0.66	27.8733
JA225		9.569	0.034	145.7289	1.60	76.6095

Max Area% 97.744

UV Signal Purity>95% Pass

Signal Description DAD1 C, Sig=320,150 Ref=off

Sample Name	Name	RT	Width	Area	Area%	Height
JA225		5.460	0.071	3927.7476	98.11	851.2654
JA225		7.760	0.073	33.9937	0.85	8.1633
JA225		9.569	0.040	41.6723	1.04	15.9330

Max Area% 98.110

UV Signal Purity>95% Pass

^{1}H, ^{13}C NMR and HPLC data of compound **11e**.

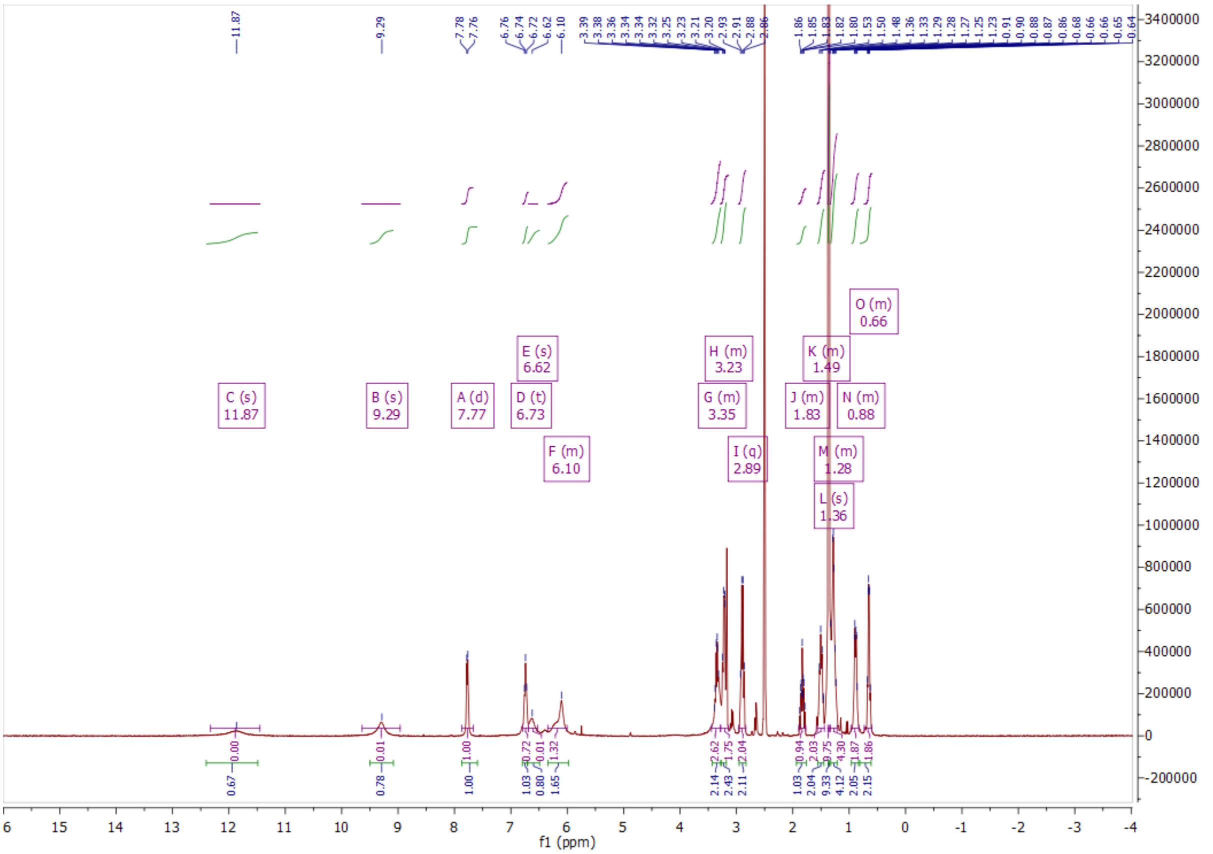

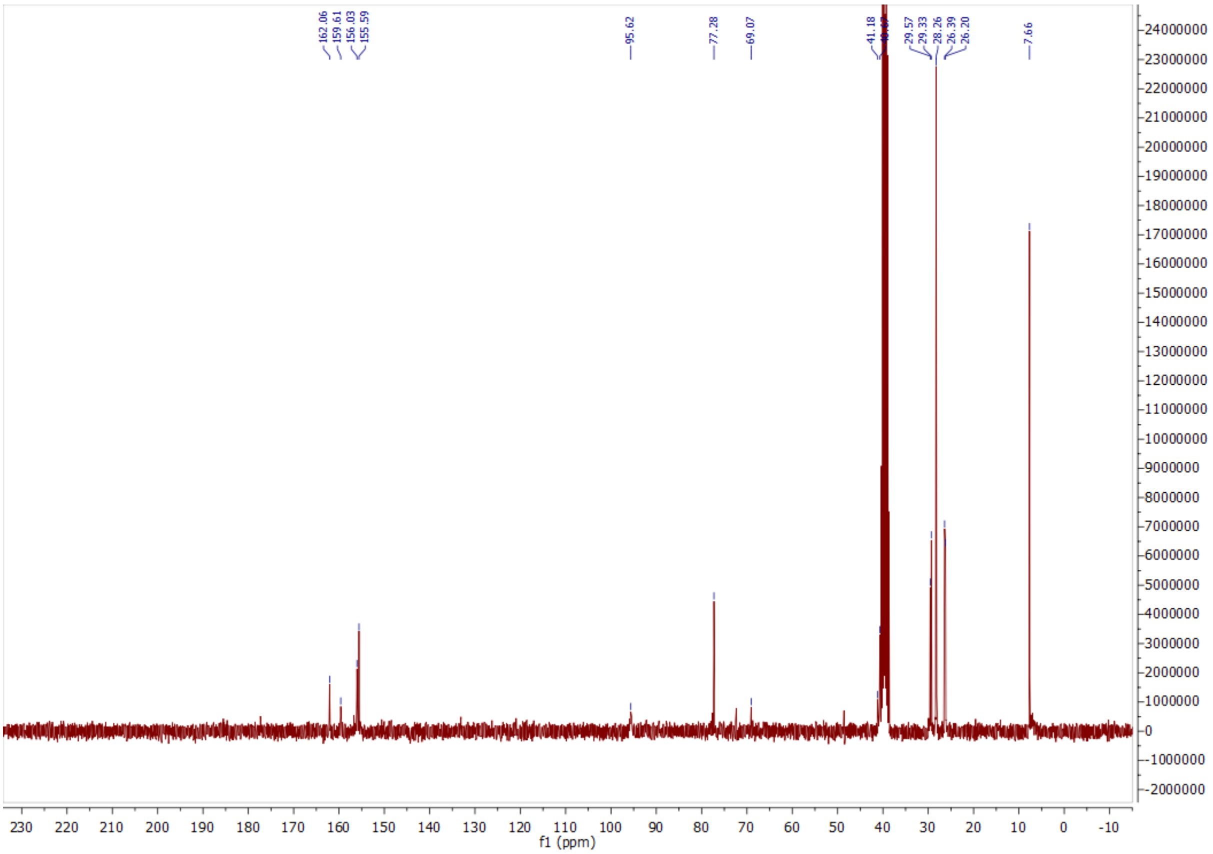

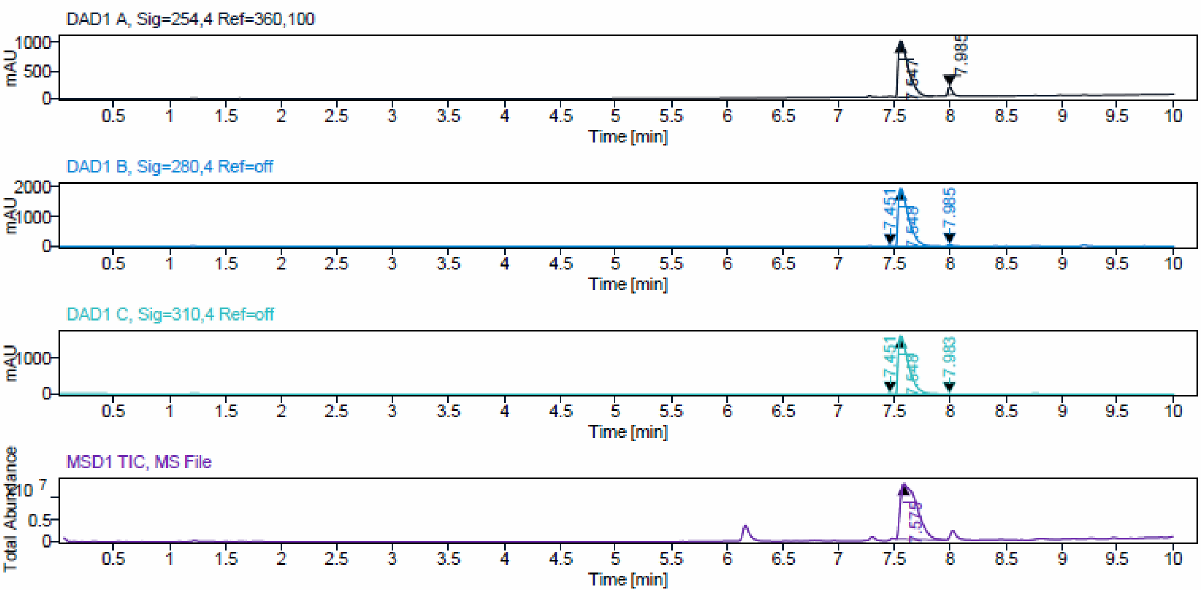

Sample Purity

Signal Description DAD1 A, Sig=254,4 Ref=360,100

Sample Name	Name	RT	Width	Area	Area%	Height
JA226		7.547	0.097	6507.7070	95.41	1024.2211
JA226		7.985	0.037	313.2963	4.59	144.7537

Max Area% 95.407

UV Signal Purity>95% Pass

Signal Description DAD1 B, Sig=280,4 Ref=off

Sample Name	Name	RT	Width	Area	Area%	Height
JA226		7.451	0.038	42.4846	0.33	18.2119
JA226		7.548	0.097	12667.23 73	98.51	1984.4272
JA226		7.985	0.041	148.8745	1.16	51.5651

Max Area% 98.512

UV Signal Purity>95% Pass

Signal Description DAD1 C, Sig=310,4 Ref=off

Sample Name	Name	RT	Width	Area	Area%	Height
JA226		7.451	0.038	31.9454	0.31	13.7988
JA226		7.548	0.097	10220.36 04	99.63	1598.6370
JA226		7.983	0.041	5.8305	0.06	1.9151

Max Area% 99.632

UV Signal Purity>95% Pass

^{1}H, ^{13}C NMR and HPLC data of compound **11f**.

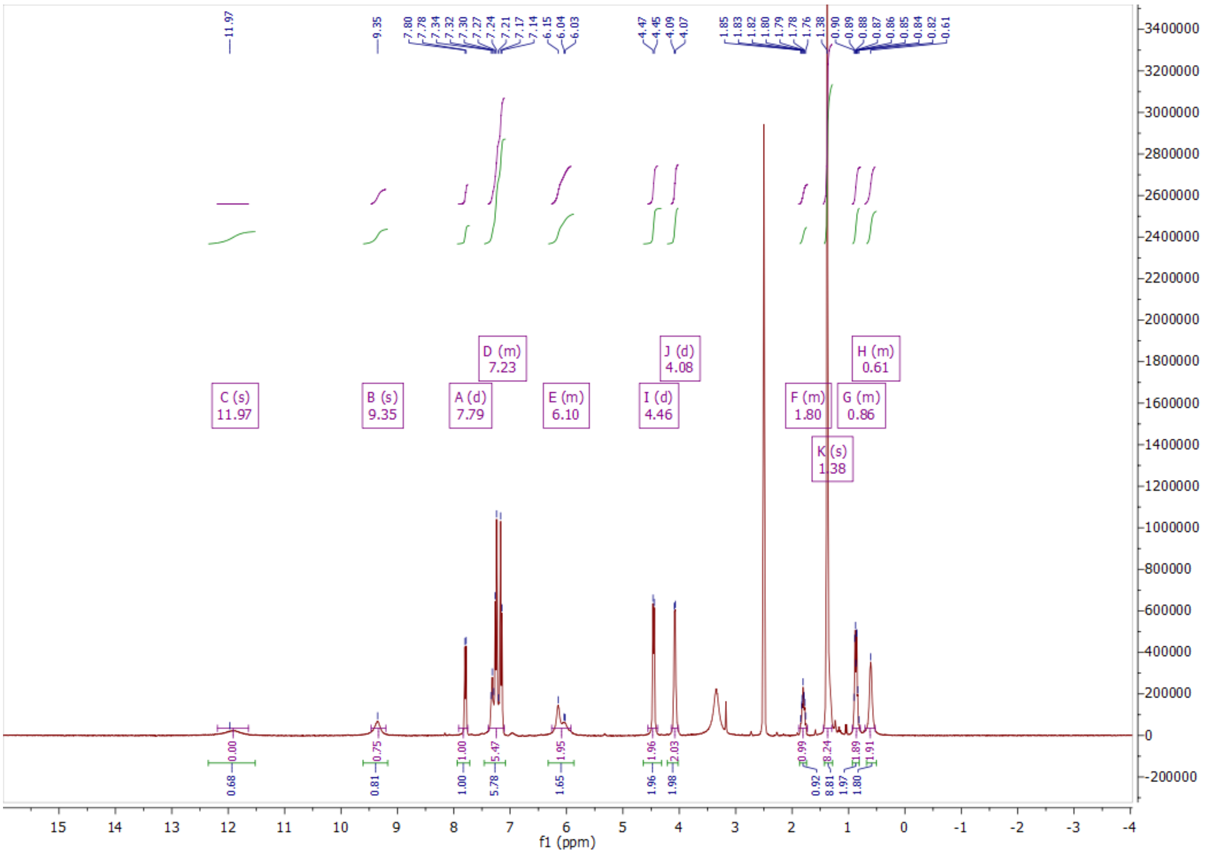

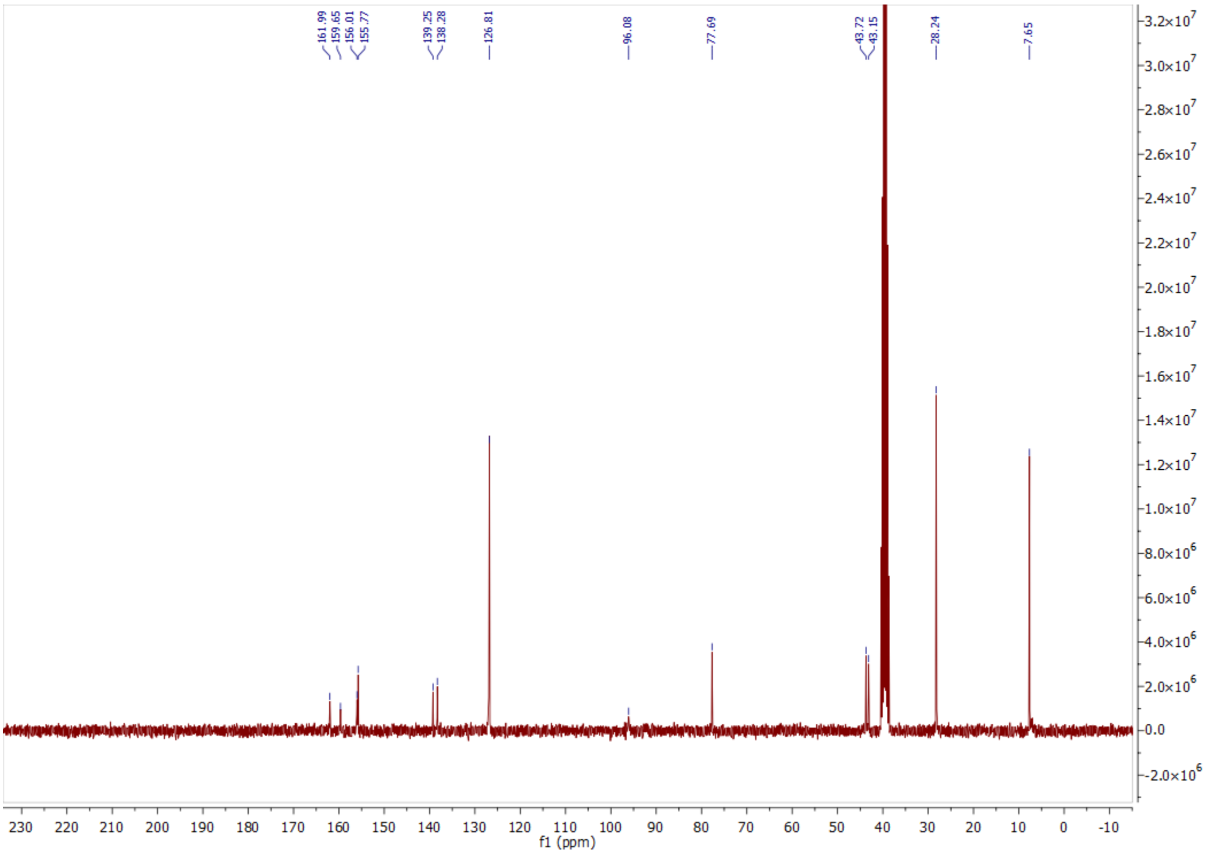

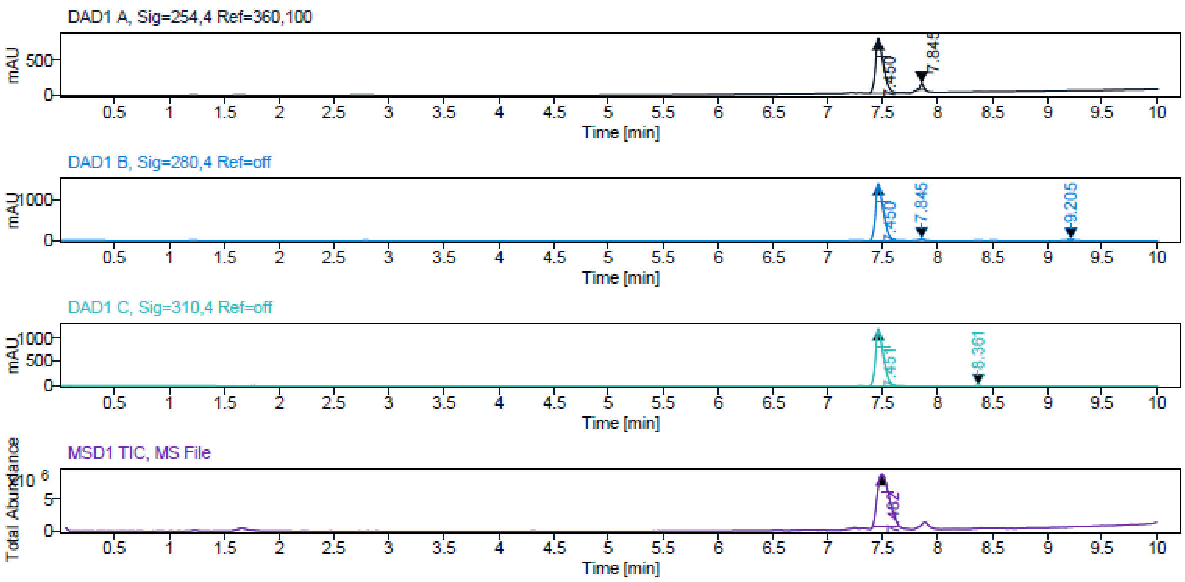

Sample Purity

Signal Description	DAD1 A, Sig=254,4 Ref=360,100

Sample Name	Name	RT	Width	Area	Area%	Height
JA243_F15-16		7.450	0.075	3961.0994	95.77	781.0707
JA243_F15-16		7.845	0.031	174.9273	4.23	90.4024

Max Area%	95.771
UV Signal Purity>95%	Pass
Signal Description	DAD1 B, Sig=280,4 Ref=off

Sample Name	Name	RT	Width	Area	Area%	Height
JA243_F15-16		7.450	0.075	7077.0752	98.11	1402.4868
JA243_F15-16		7.845	0.038	101.0171	1.40	38.2763
JA243_F15-16		9.205	0.035	35.2847	0.49	19.2278

Max Area%	98.110
UV Signal Purity>95%	Pass

Signal Description	DAD1 C, Sig=310,4 Ref=off

Sample Name	Name	RT	Width	Area	Area%	Height
JA243_F15-16		7.451	0.075	6019.2920	99.00	1202.3838
JA243_F15-16		8.361	0.121	61.0018	1.00	10.4350

Max Area%	98.997
UV Signal Purity>95%	Pass

^{1}H, ^{13}C NMR and HPLC data of compound **12a**.

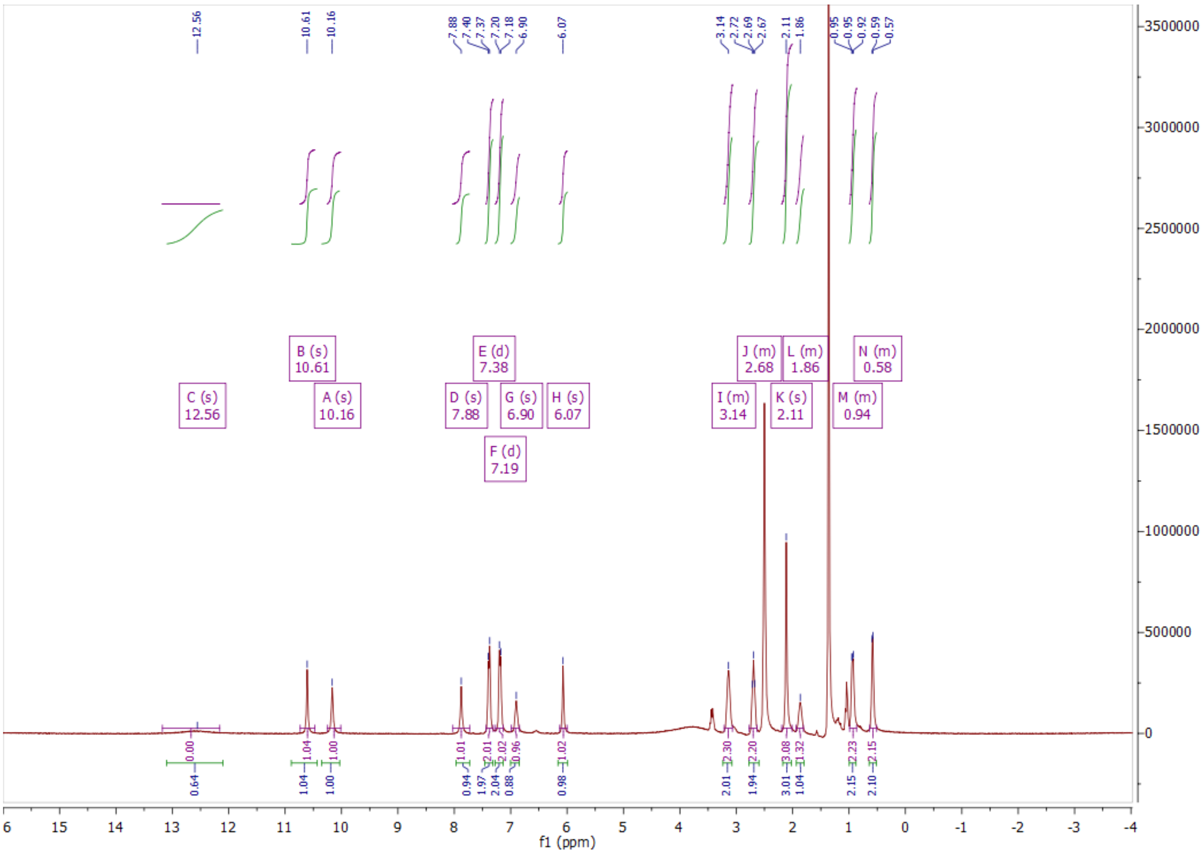

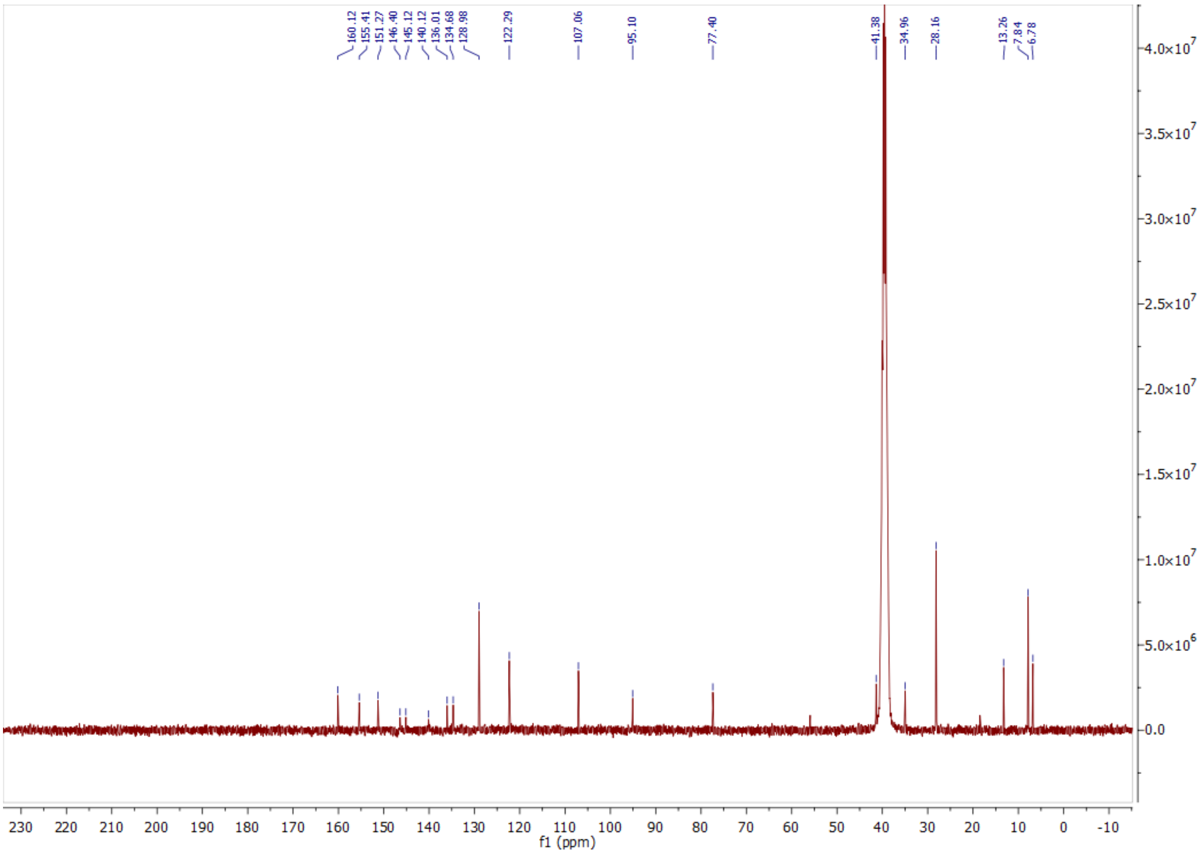

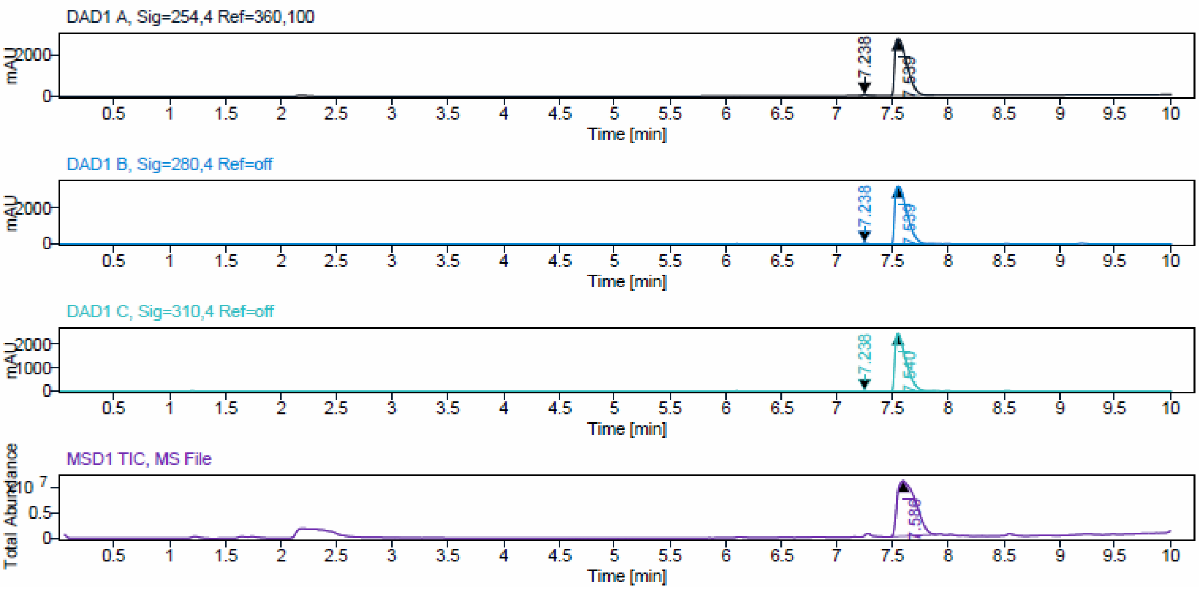

Sample Purity

Signal Description	DAD1 A, Sig=254,4 Ref=360,100

Sample Name	Name	RT	Width	Area	Area%	Height
JA238_F12-13		7.238	0.034	169.2494	0.81	69.2998
JA238_F12-13		7.539	0.118	20648.13 48	99.19	2747.5686

Max Area%	99.187
UV Signal Purity>95%	Pass
Signal Description	DAD1 B, Sig=280,4 Ref=off

Sample Name	Name	RT	Width	Area	Area%	Height
JA238_F12-13		7.238	0.033	119.0443	0.49	48.8686
JA238_F12-13		7.539	0.117	24093.73 44	99.51	3230.1929

Max Area%	99.508
UV Signal Purity>95%	Pass

Signal Description	DAD1 C, Sig=310,4 Ref=off

Sample Name	Name	RT	Width	Area	Area%	Height
JA238_F12-13		7.238	0.033	28.0562	0.16	11.7167
JA238_F12-13		7.540	0.106	17101.58 59	99.84	2500.2917

Max Area%	99.836
UV Signal Purity>95%	Pass

^{1}H, ^{13}C NMR and HPLC data of compound **12b**.

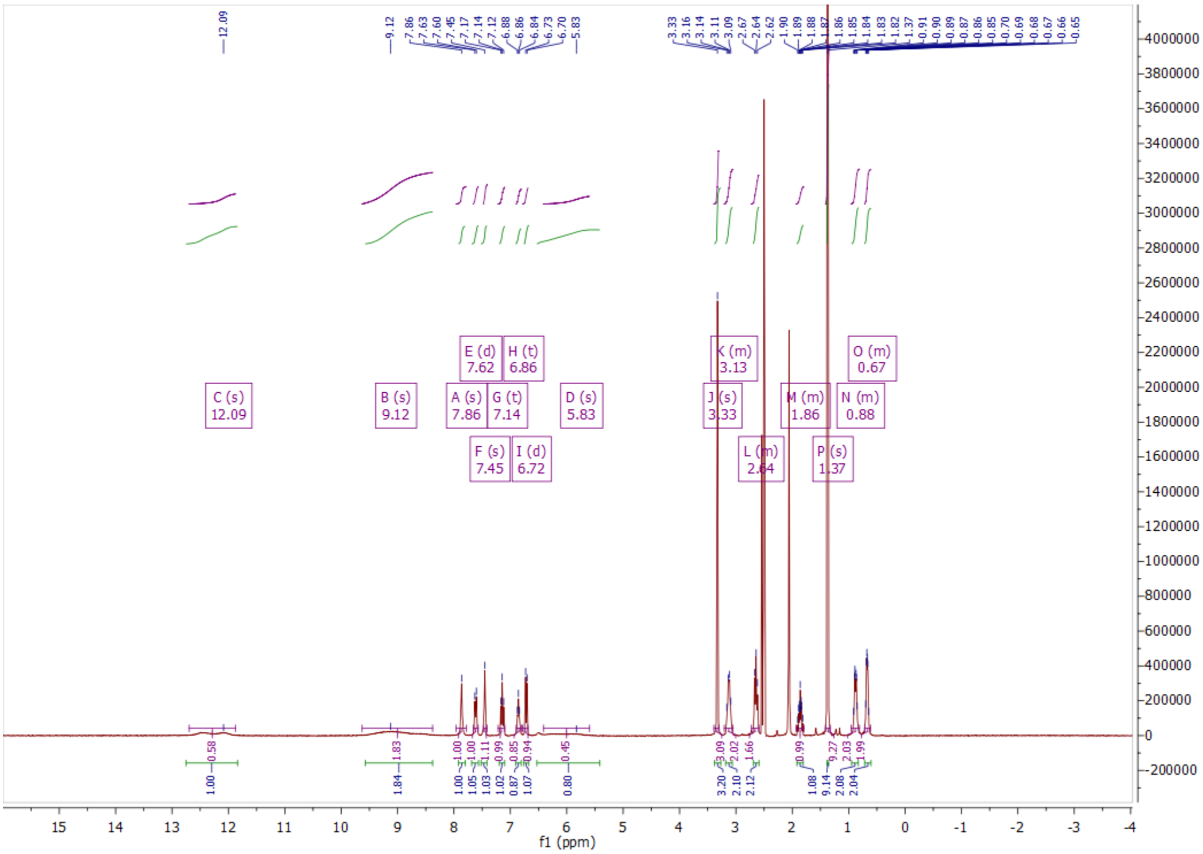

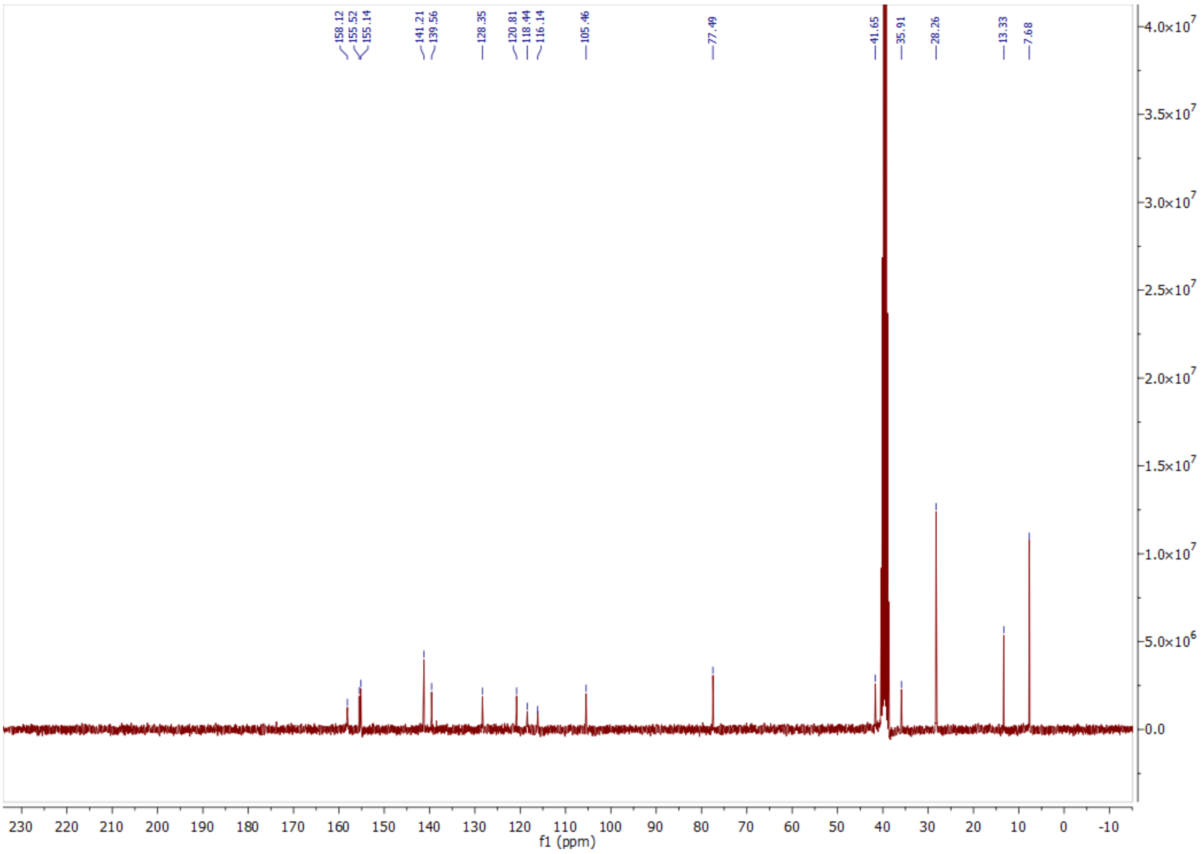

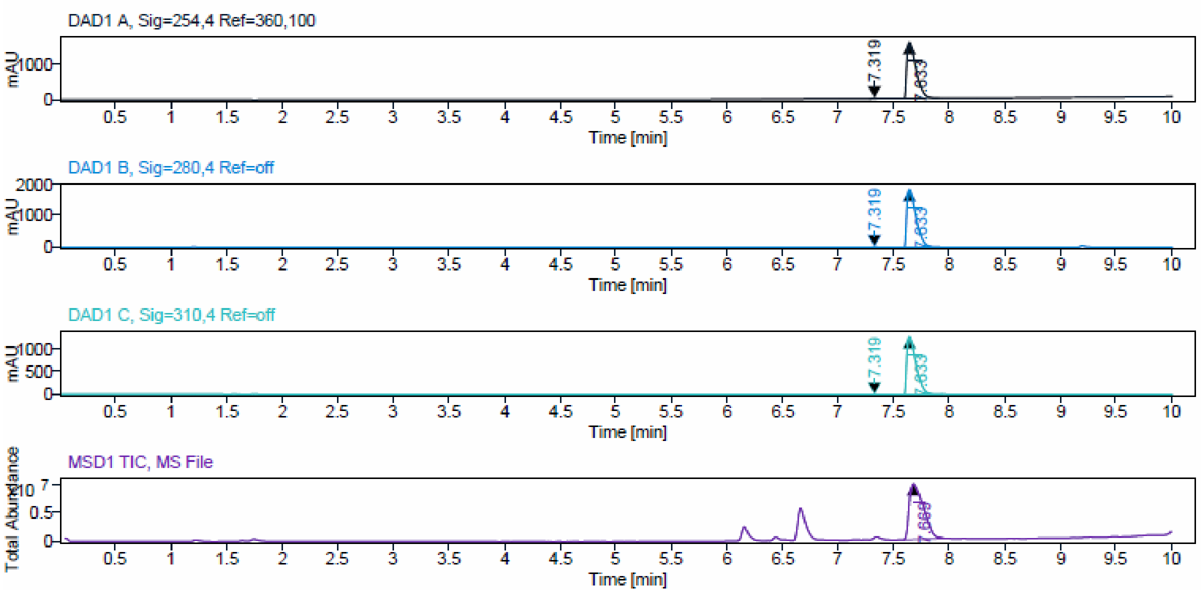

Sample Purity

Signal Description DAD1 A, Sig=254,4 Ref=360,100

Sample Name	Name	RT	Width	Area	Area%	Height
JA239_F29		7.319	0.035	50.5336	0.55	20.1977
JA239_F29		7.633	0.089	9114.3486	99.45	1607.0404

Max Area% 99.449

UV Signal Purity>95% Pass

Signal Description DAD1 B, Sig=280,4 Ref=off

Sample Name	Name	RT	Width	Area	Area%	Height
JA239_F29		7.319	0.034	33.9213	0.32	12.5852
JA239_F29		7.633	0.088	10594.57 32	99.68	1860.9395

Max Area% 99.681

UV Signal Purity>95% Pass

Signal Description DAD1 C, Sig=310,4 Ref=off

Sample Name	Name	RT	Width	Area	Area%	Height
JA239_F29		7.319	0.036	11.3008	0.15	3.2576
JA239_F29		7.633	0.088	7456.0742	99.85	1309.5631

Max Area% 99.849

UV Signal Purity>95% Pass

[1]H and HPLC data of compound **12c**.

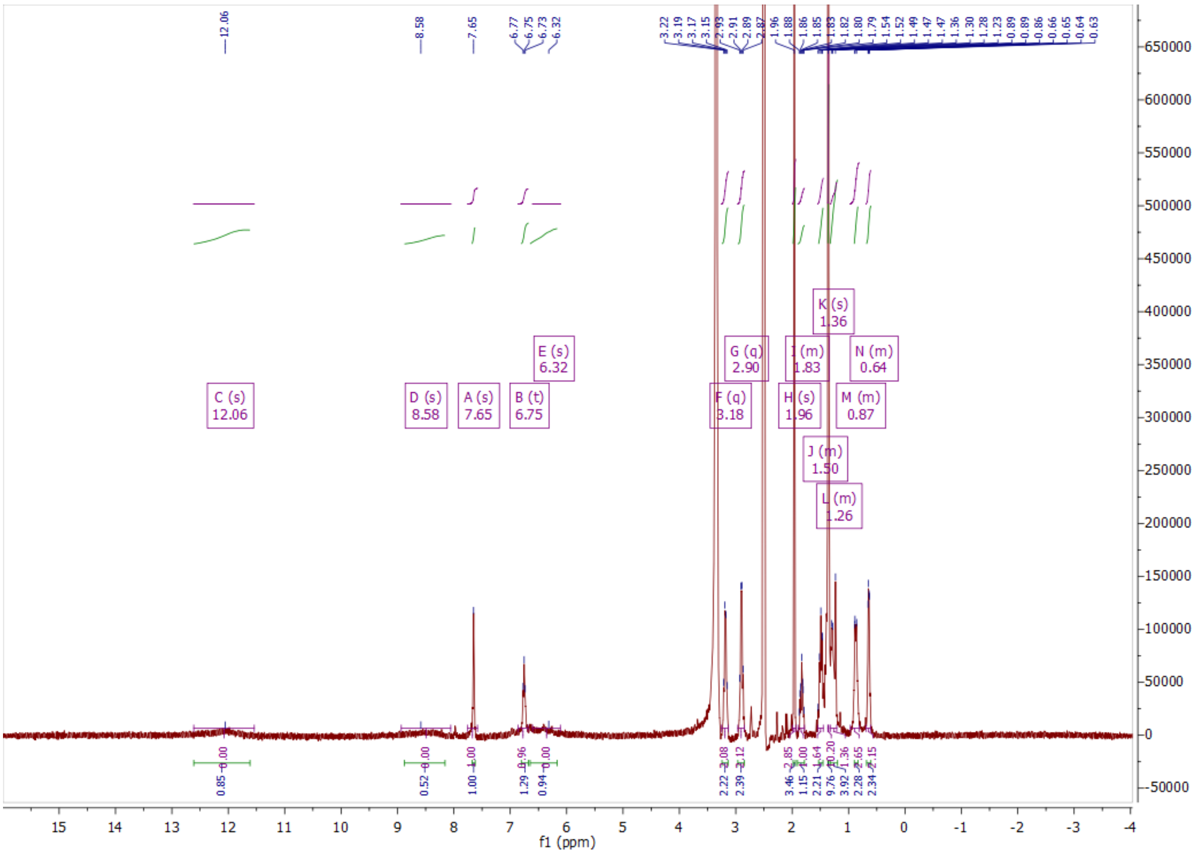

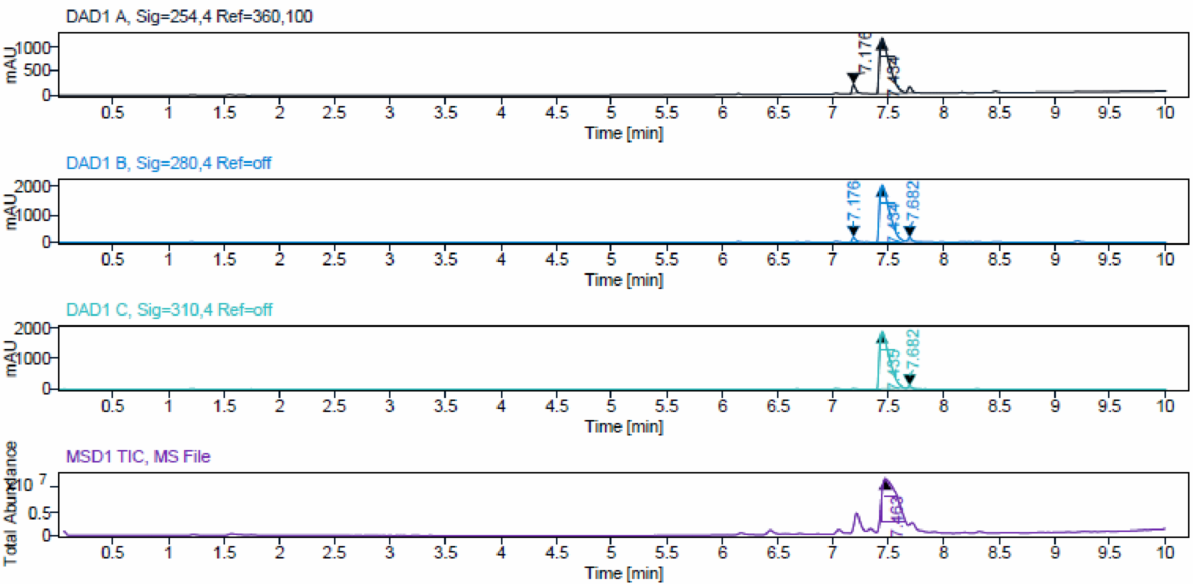

Sample Purity

Signal Description DAD1 A, Sig=254,4 Ref=360,100

Sample Name	Name	RT	Width	Area	Area%	Height
JA236_F6-9		7.176	0.036	393.9118	4.98	180.7378
JA236_F6-9		7.434	0.097	7508.4521	95.02	1158.0663

Max Area% 95.015

UV Signal Purity>95% Pass

Signal Description DAD1 B, Sig=280,4 Ref=off

Sample Name	Name	RT	Width	Area	Area%	Height
JA236_F6-9		7.176	0.035	345.7982	2.48	160.5132
JA236_F6-9		7.434	0.097	13307.28 22	95.26	2124.7468
JA236_F6-9		7.682	0.034	316.4581	2.27	140.7838

Max Area% 95.259

UV Signal Purity>95% Pass

Signal Description DAD1 C, Sig=310,4 Ref=off

Sample Name	Name	RT	Width	Area	Area%	Height
JA236_F6-9		7.435	0.097	11826.64 84	98.71	1913.0609
JA236_F6-9		7.682	0.032	154.1769	1.29	71.9098

Max Area% 98.713

UV Signal Purity>95% Pass

^{1}H, ^{13}C NMR and HPLC data of compound **12d**.

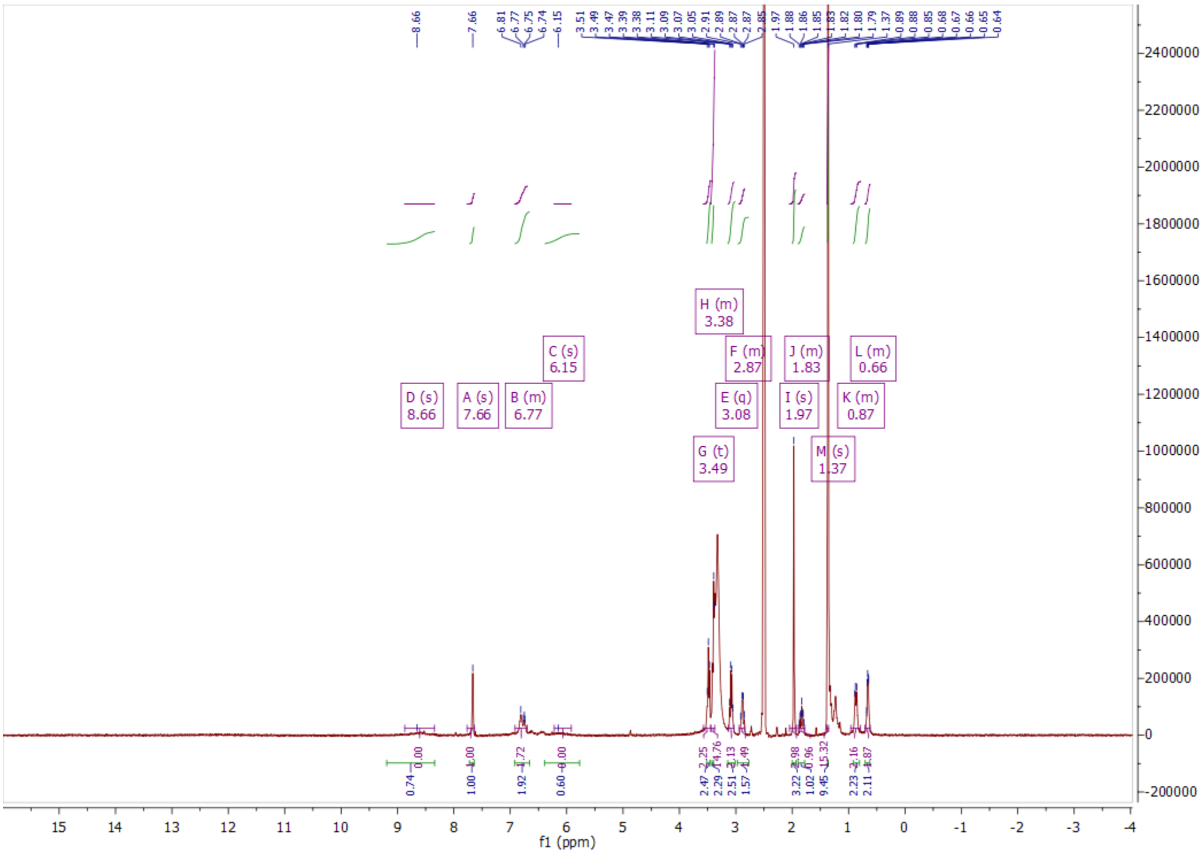

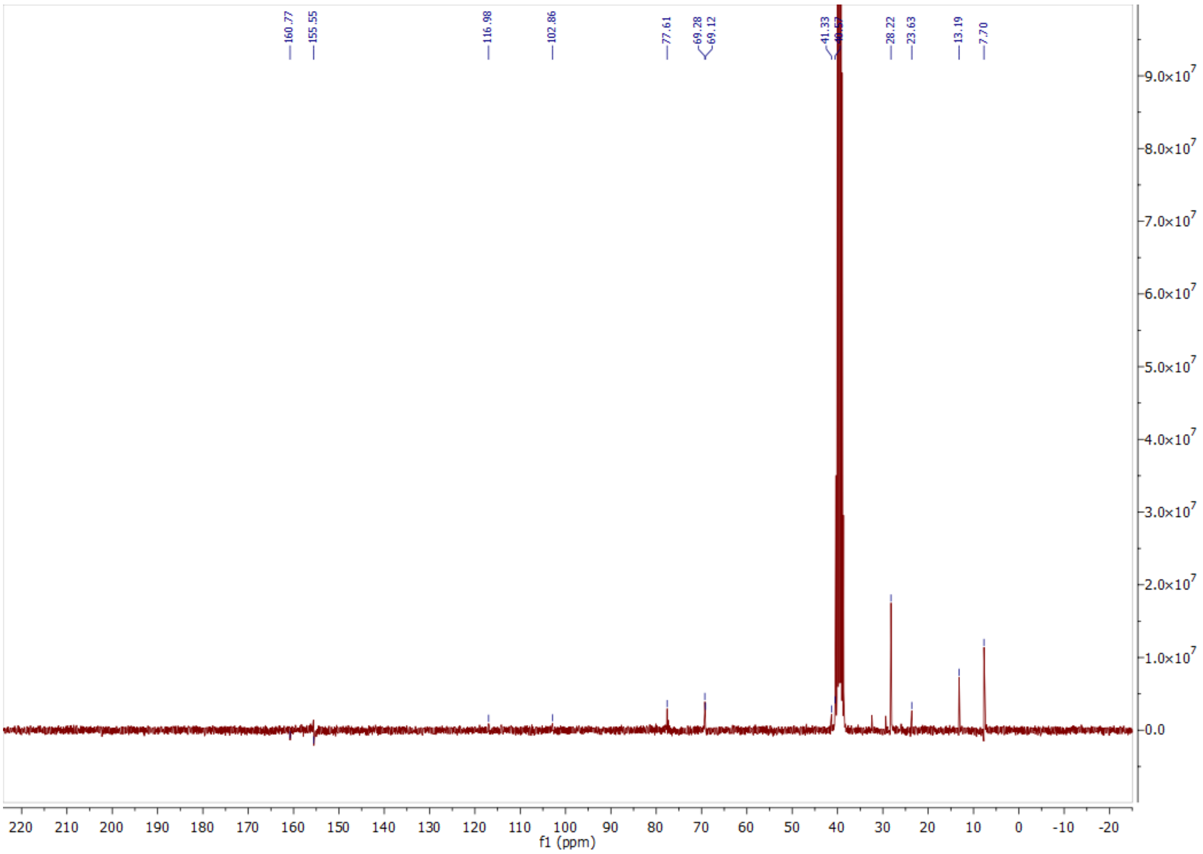

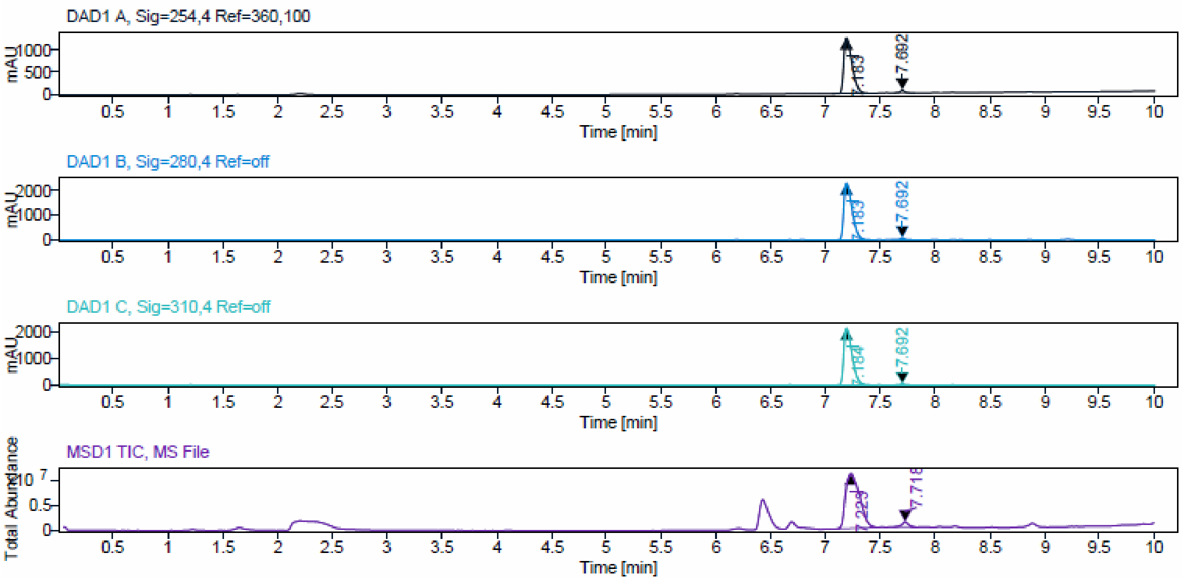

Sample Purity

Signal Description DAD1 A, Sig=254,4 Ref=360,100

Sample Name	Name	RT	Width	Area	Area%	Height
JA240		7.183	0.080	6479.6777	97.30	1263.5648
JA240		7.692	0.037	179.6208	2.70	63.2616

Max Area% 97.303

UV Signal Purity>95% Pass

Signal Description DAD1 B, Sig=280,4 Ref=off

Sample Name	Name	RT	Width	Area	Area%	Height
JA240		7.183	0.080	11870.00 59	98.01	2287.4702
JA240		7.692	0.037	240.6320	1.99	79.1497

Max Area% 98.013

UV Signal Purity>95% Pass

Signal Description DAD1 C, Sig=310,4 Ref=off

Sample Name	Name	RT	Width	Area	Area%	Height
JA240		7.184	0.080	11056.12 50	98.80	2131.0474
JA240		7.692	0.038	134.4070	1.20	43.1608

Max Area% 98.799

UV Signal Purity>95% Pass

[1]H, and HPLC data of compound **12e**.

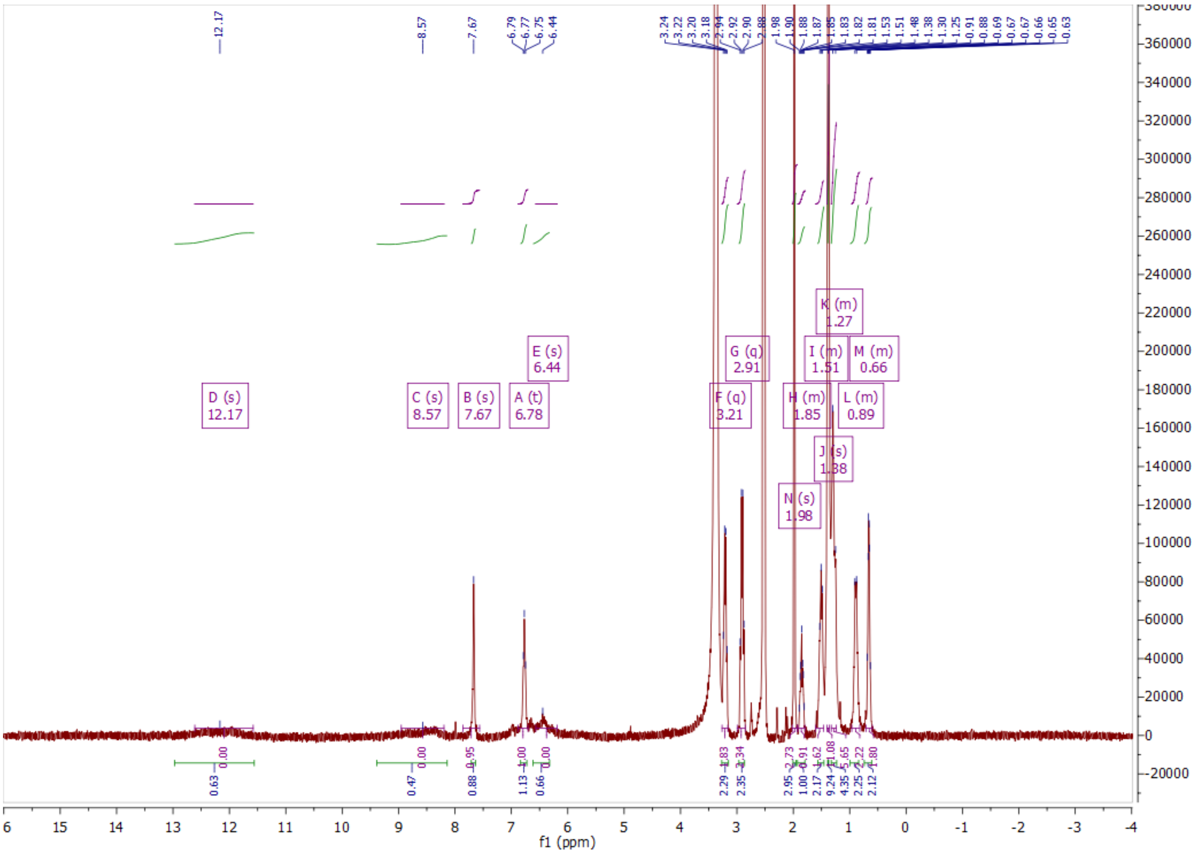

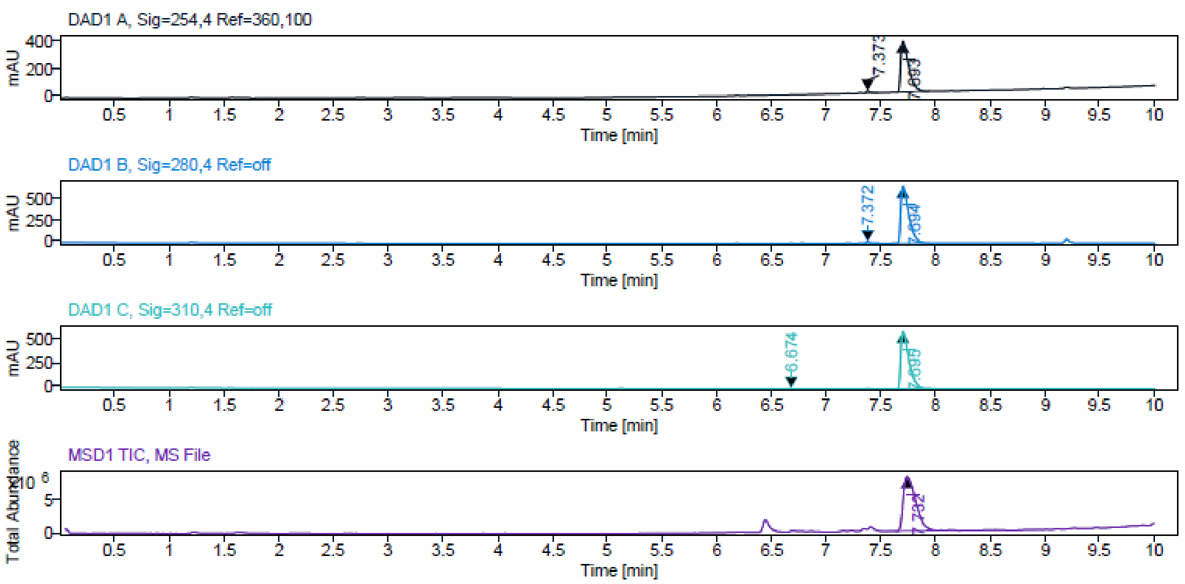

Sample Purity

Signal Description DAD1 A, Sig=254,4 Ref=360,100

Sample Name	Name	RT	Width	Area	Area%	Height
JA237_F8-10		7.373	0.031	34.2503	1.86	15.1012
JA237_F8-10		7.693	0.077	1808.7574	98.14	362.0468

Max Area% 98.142

UV Signal Purity>95% Pass

Signal Description DAD1 B, Sig=280,4 Ref=off

Sample Name	Name	RT	Width	Area	Area%	Height
JA237_F8-10		7.372	0.030	32.9190	0.96	14.1122
JA237_F8-10		7.694	0.077	3390.3953	99.04	670.4847

Max Area% 99.038

UV Signal Purity>95% Pass

Signal Description DAD1 C, Sig=310,4 Ref=off

Sample Name	Name	RT	Width	Area	Area%	Height
JA237_F8-10		6.674	0.036	6.9030	0.22	3.6943
JA237_F8-10		7.695	0.078	3088.6848	99.78	603.7136

Max Area% 99.777

UV Signal Purity>95% Pass

^{1}H, ^{13}C NMR and HPLC data of compound **13a**.

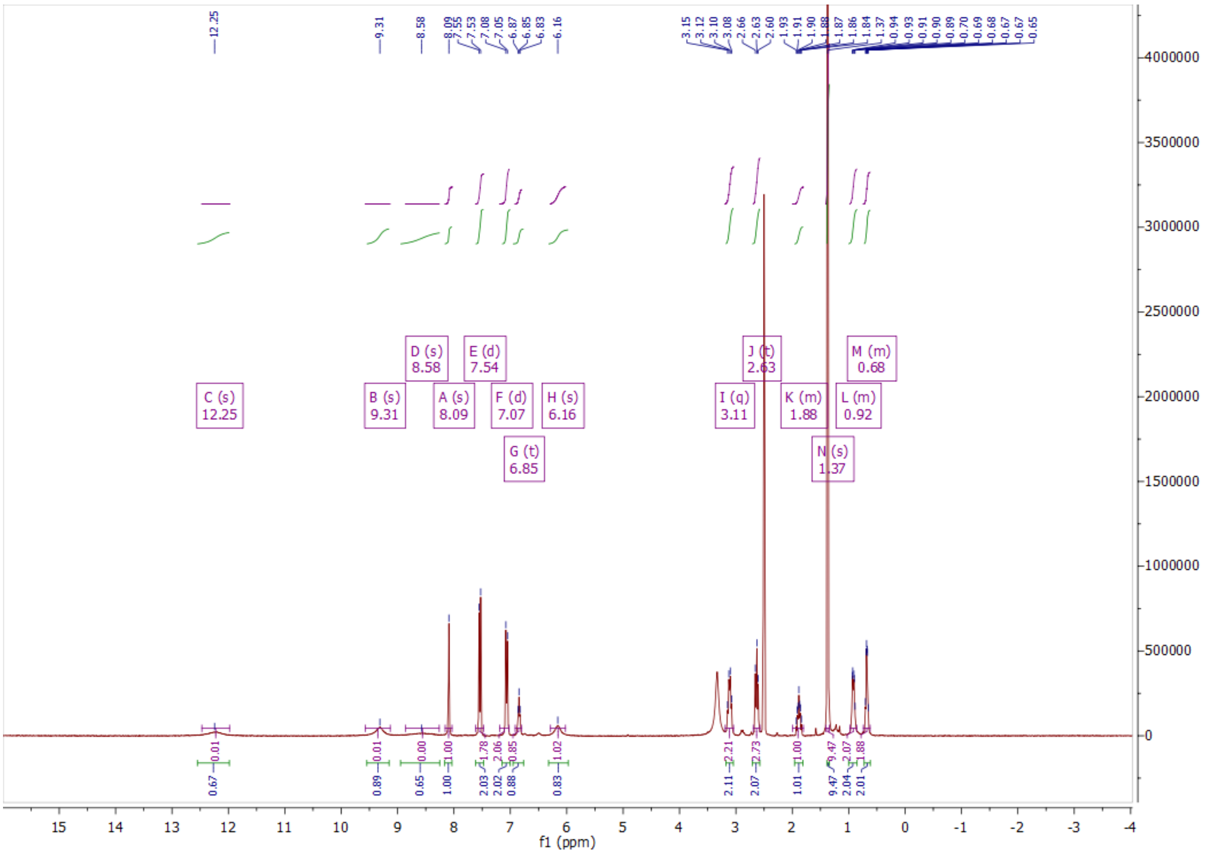

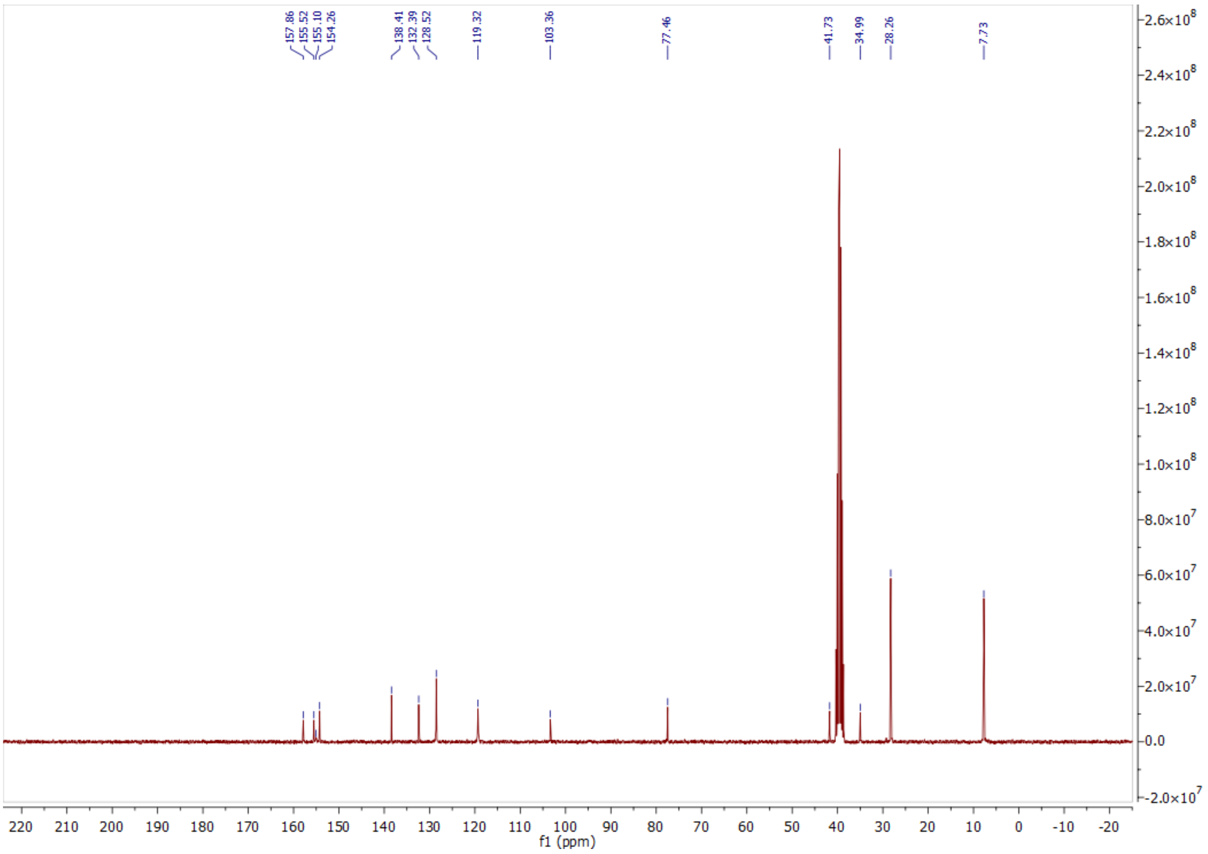

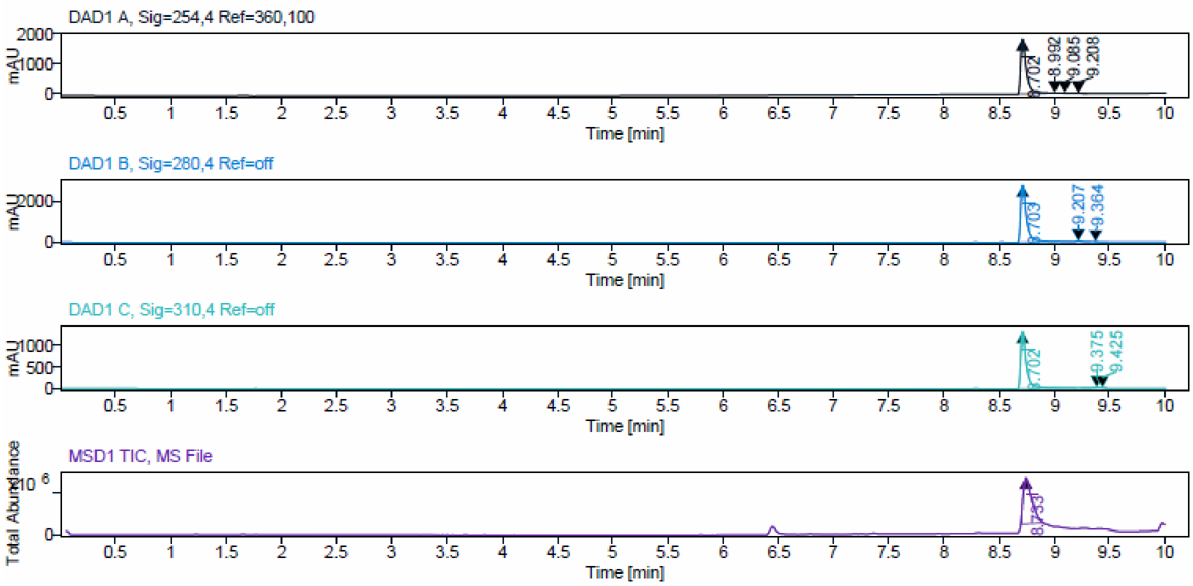

Sample Purity

Signal Description DAD1 A, Sig=254,4 Ref=360,100

Sample Name	Name	RT	Width	Area	Area%	Height
JA271_F5		8.702	0.046	6139.7017	97.55	1819.5193
JA271_F5		8.992	0.032	10.6366	0.17	5.7538
JA271_F5		9.085	0.071	64.6120	1.03	13.6139
JA271_F5		9.208	0.090	78.7719	1.25	14.8513

Max Area% 97.553

UV Signal Purity>95% Pass

Signal Description DAD1 B, Sig=280,4 Ref=off

Sample Name	Name	RT	Width	Area	Area%	Height
JA271_F5		8.703	0.048	10216.98 73	98.66	2804.9519
JA271_F5		9.207	0.043	124.6276	1.20	42.9633
JA271_F5		9.364	0.034	14.6464	0.14	1.9193

Max Area% 98.655

UV Signal Purity>95% Pass

Signal Description DAD1 C, Sig=310,4 Ref=off

Sample Name	Name	RT	Width	Area	Area%	Height
JA271_F5		8.702	0.045	4629.5195	99.43	1332.4393
JA271_F5		9.375	0.042	19.9881	0.43	6.0711
JA271_F5		9.425	0.026	6.6135	0.14	2.5773

Max Area% 99.429

UV Signal Purity>95% Pass

^{1}H, ^{13}C NMR and HPLC data of compound **13c**.

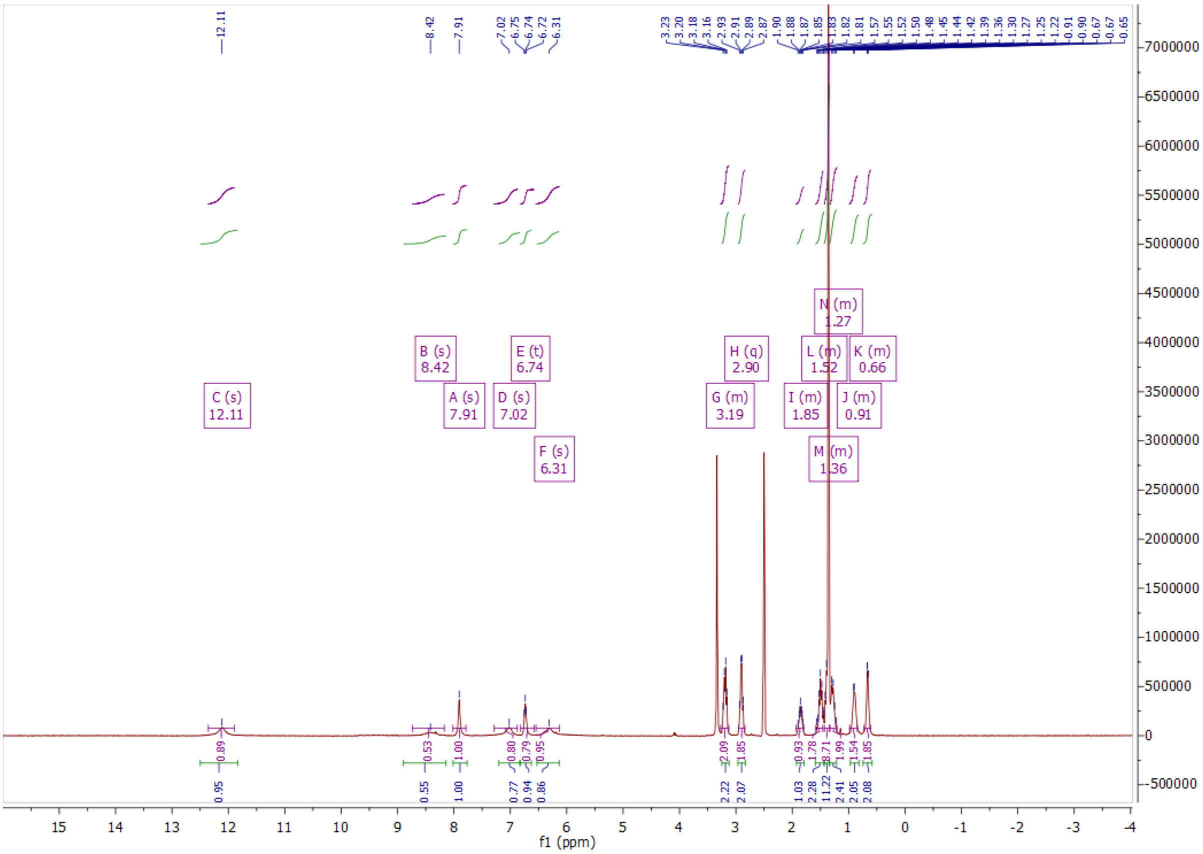

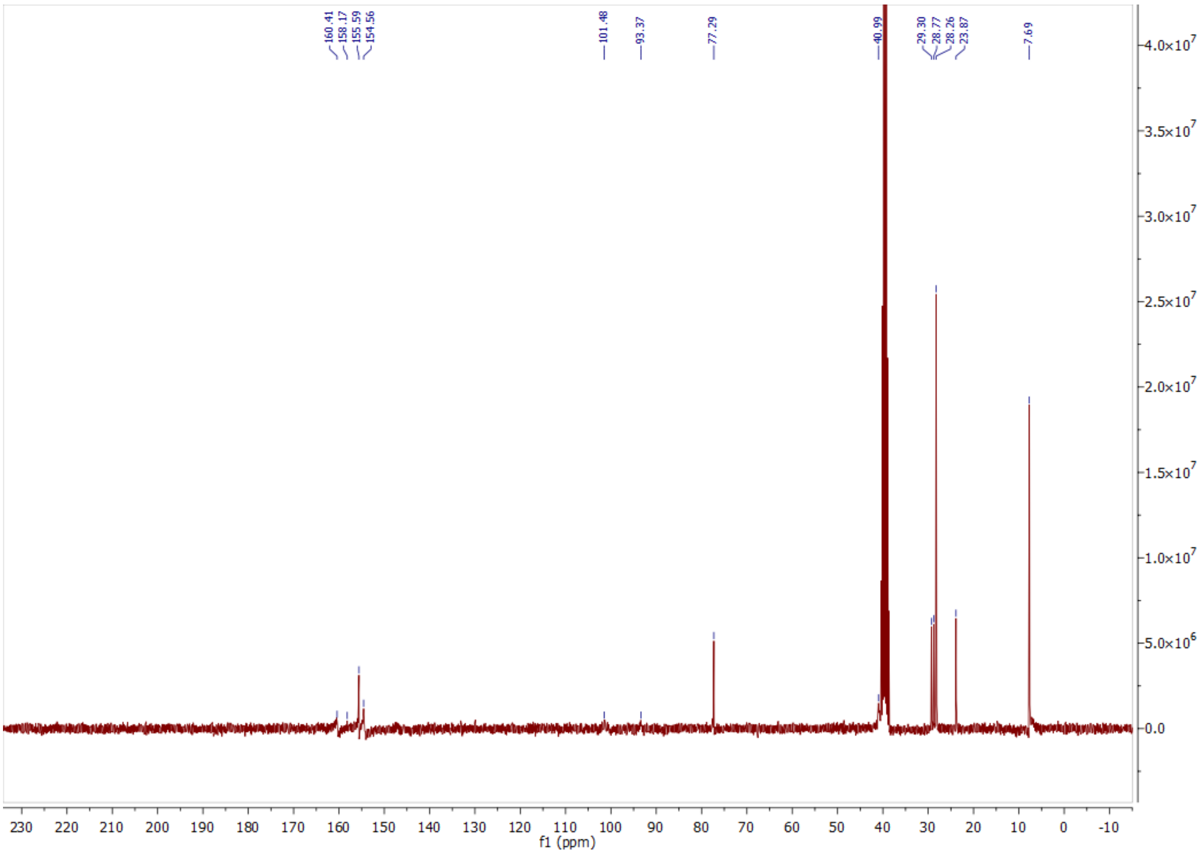

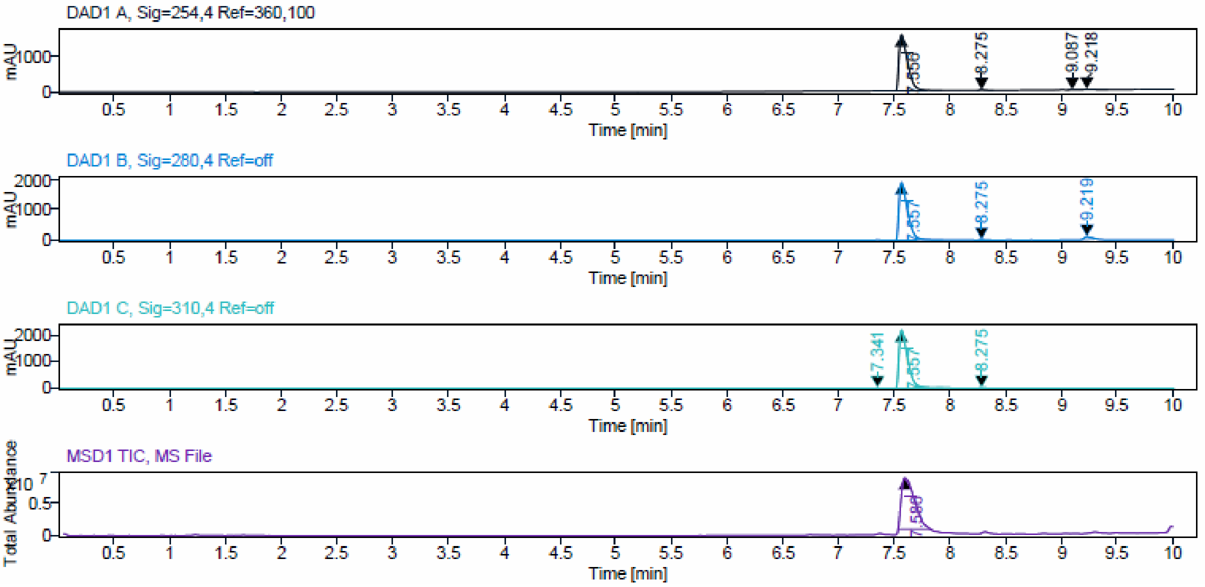

Sample Purity

Signal Description DAD1 A, Sig=254,4 Ref=360,100

Sample Name	Name	RT	Width	Area	Area%	Height
JA273_neu_F5		7.556	0.082	8402.1455	97.72	1566.9601
JA273_neu_F5		8.275	0.041	63.1049	0.73	21.8376
JA273_neu_F5		9.087	0.056	29.3713	0.34	9.6526
JA273_neu_F5		9.218	0.097	103.4959	1.20	17.0535

Max Area% 97.721

UV Signal Purity>95% Pass

Signal Description DAD1 B, Sig=280,4 Ref=off

Sample Name	Name	RT	Width	Area	Area%	Height
JA273_neu_F5		7.557	0.081	10511.3721	95.68	1972.3652
JA273_neu_F5		8.275	0.040	71.9030	0.65	25.0407
JA273_neu_F5		9.219	0.086	402.7542	3.67	91.0337

Max Area% 95.679

UV Signal Purity>95% Pass

Signal Description DAD1 C, Sig=310,4 Ref=off

Sample Name	Name	RT	Width	Area	Area%	Height
JA273_neu_F5		7.341	0.043	16.0842	0.13	4.9029
JA273_neu_F5		7.557	0.081	11918.6289	99.47	2235.0386
JA273_neu_F5		8.275	0.040	47.6917	0.40	16.5351

Max Area% 99.468

UV Signal Purity>95% Pass

^{1}H, ^{13}C NMR and HPLC data of compound **15**.

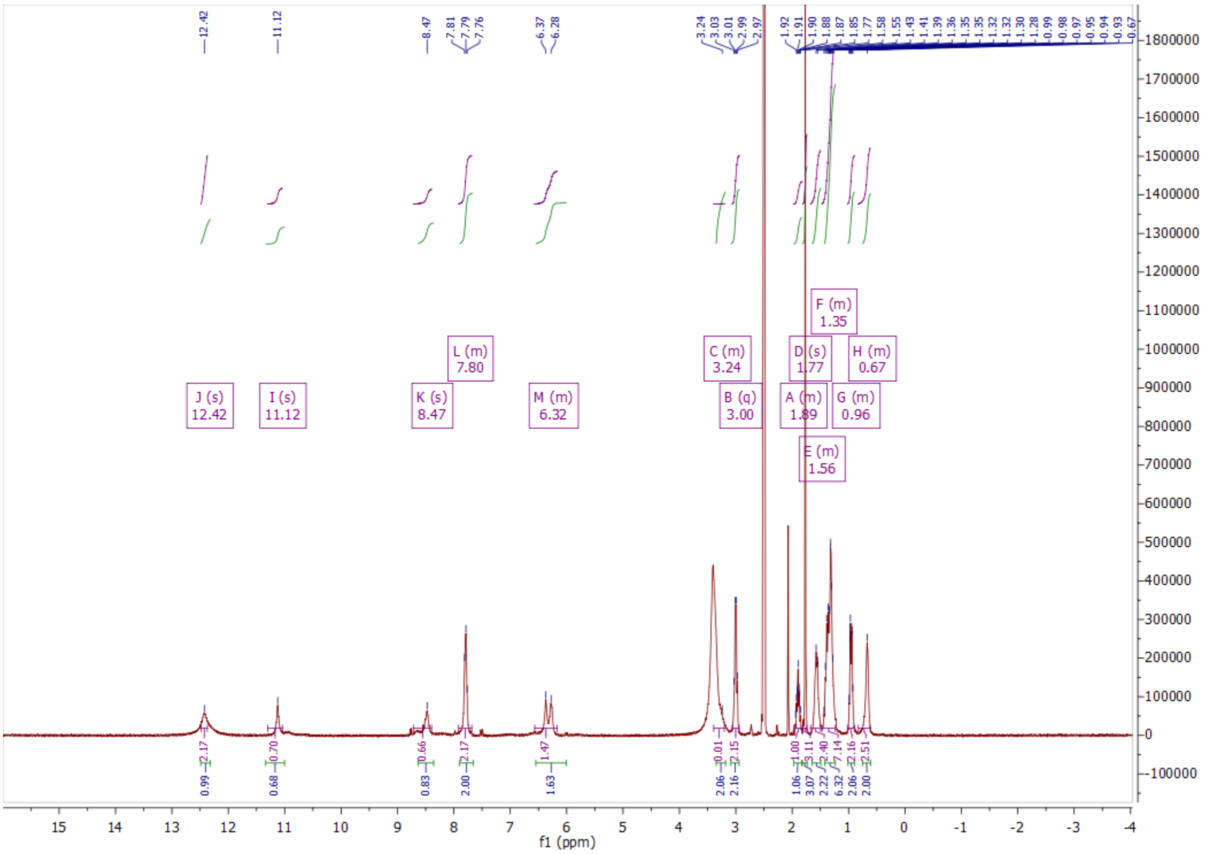

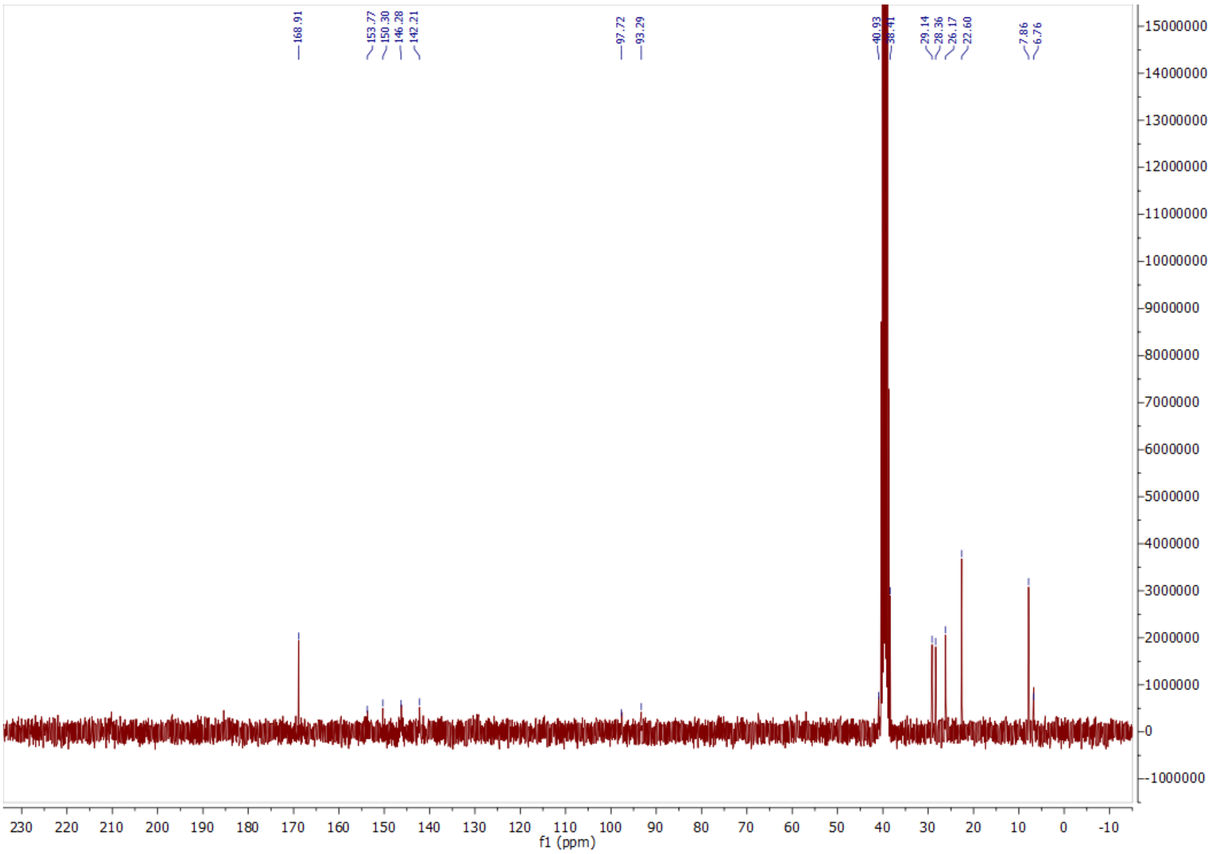

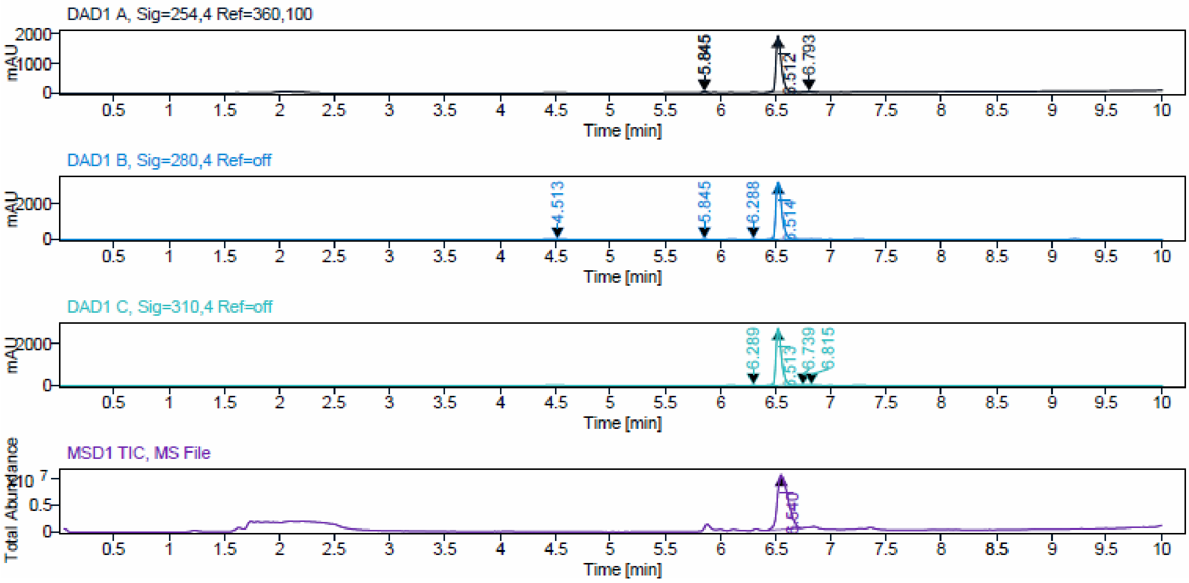

Sample Purity

Signal Description DAD1 A, Sig=254,4 Ref=360,100

Sample Name	Name	RT	Width	Area	Area%	Height
JA241_gew		5.845	0.040	112.1081	1.57	45.0589
JA241_gew		6.512	0.053	6940.5903	97.25	1950.1249
JA241_gew		6.793	0.049	84.2915	1.18	24.4807

Max Area% 97.248

UV Signal Purity>95% Pass

Signal Description DAD1 B, Sig=280,4 Ref=off

Sample Name	Name	RT	Width	Area	Area%	Height
JA241_gew		4.513	0.227	299.3524	2.29	19.8673
JA241_gew		5.845	0.040	73.9105	0.57	28.0744
JA241_gew		6.288	0.036	61.8452	0.47	24.5020
JA241_gew		6.514	0.059	12628.2021	96.67	3238.2986

Max Area% 96.669

UV Signal Purity>95% Pass

Signal Description DAD1 C, Sig=310,4 Ref=off

Sample Name	Name	RT	Width	Area	Area%	Height
JA241_gew		6.289	0.034	40.1139	0.39	16.6009
JA241_gew		6.513	0.054	10206.3232	99.30	2823.3350
JA241_gew		6.739	0.027	10.3400	0.10	6.1019
JA241_gew		6.815	0.029	21.3774	0.21	10.8509

Max Area% 99.301

UV Signal Purity>95% Pass

^{1}H and ^{13}C NMR data of compound **18**.

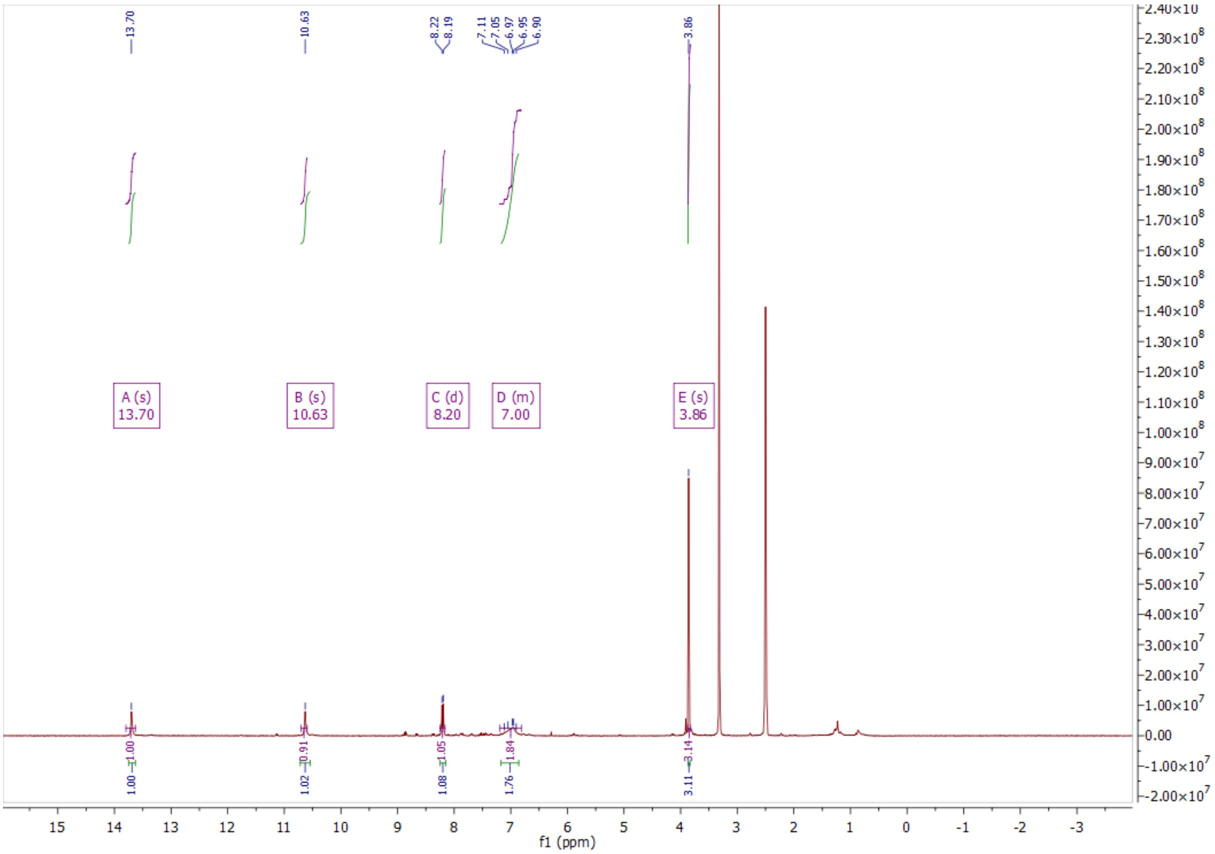

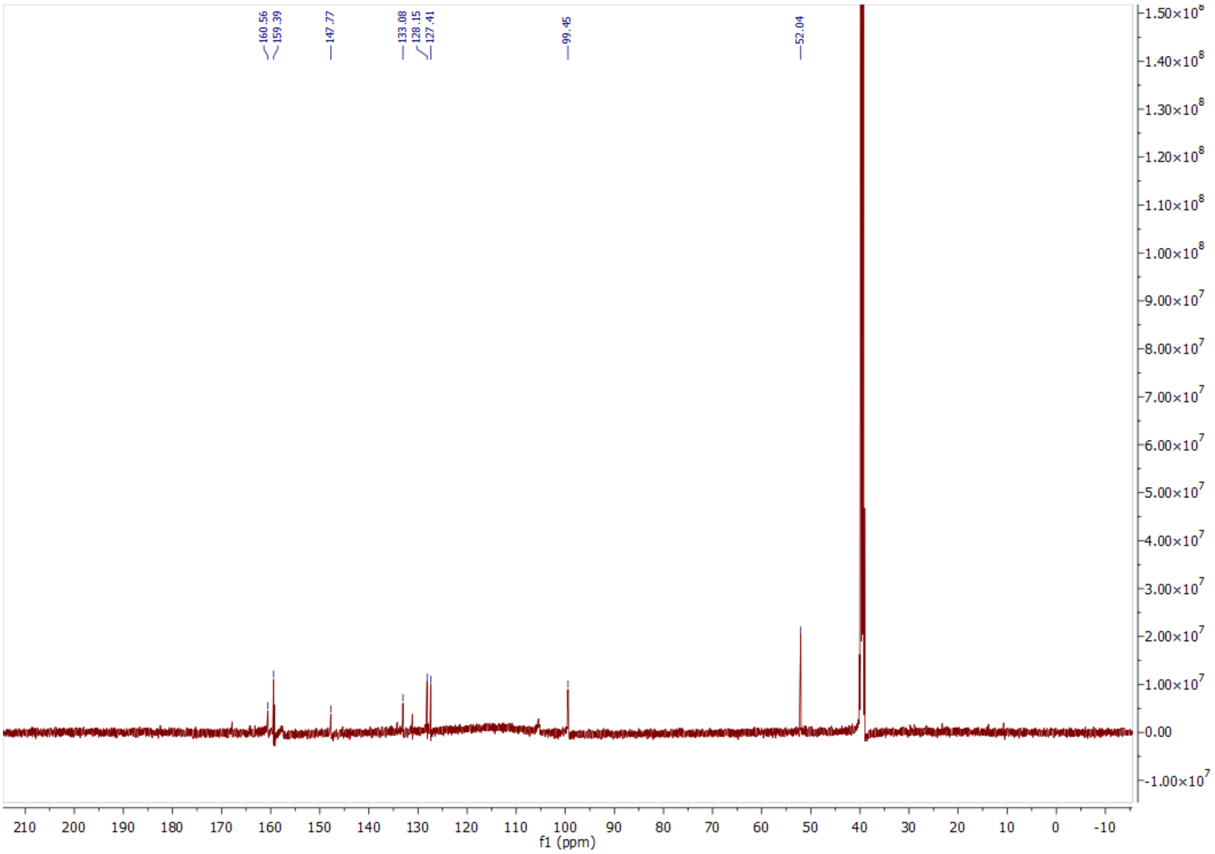

^{1}H and ^{13}C NMR data of compound **19**.

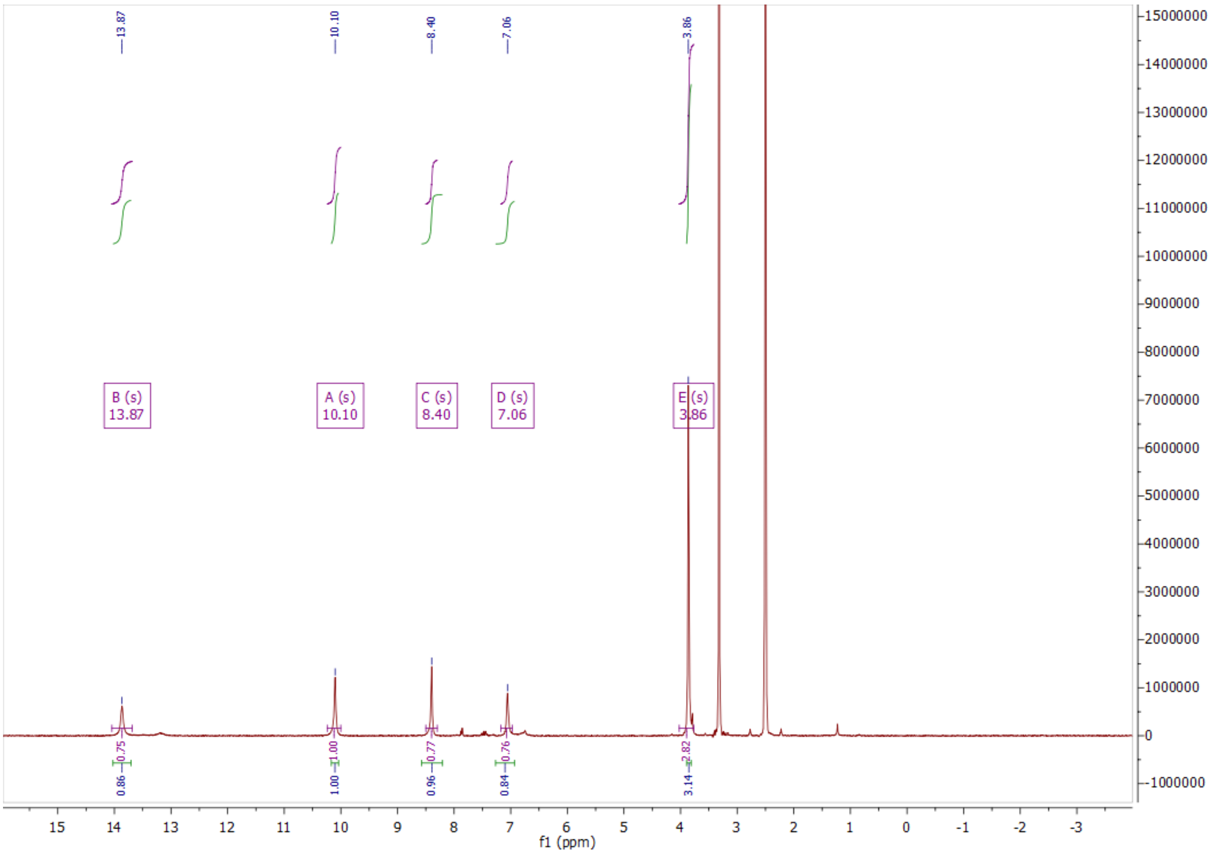

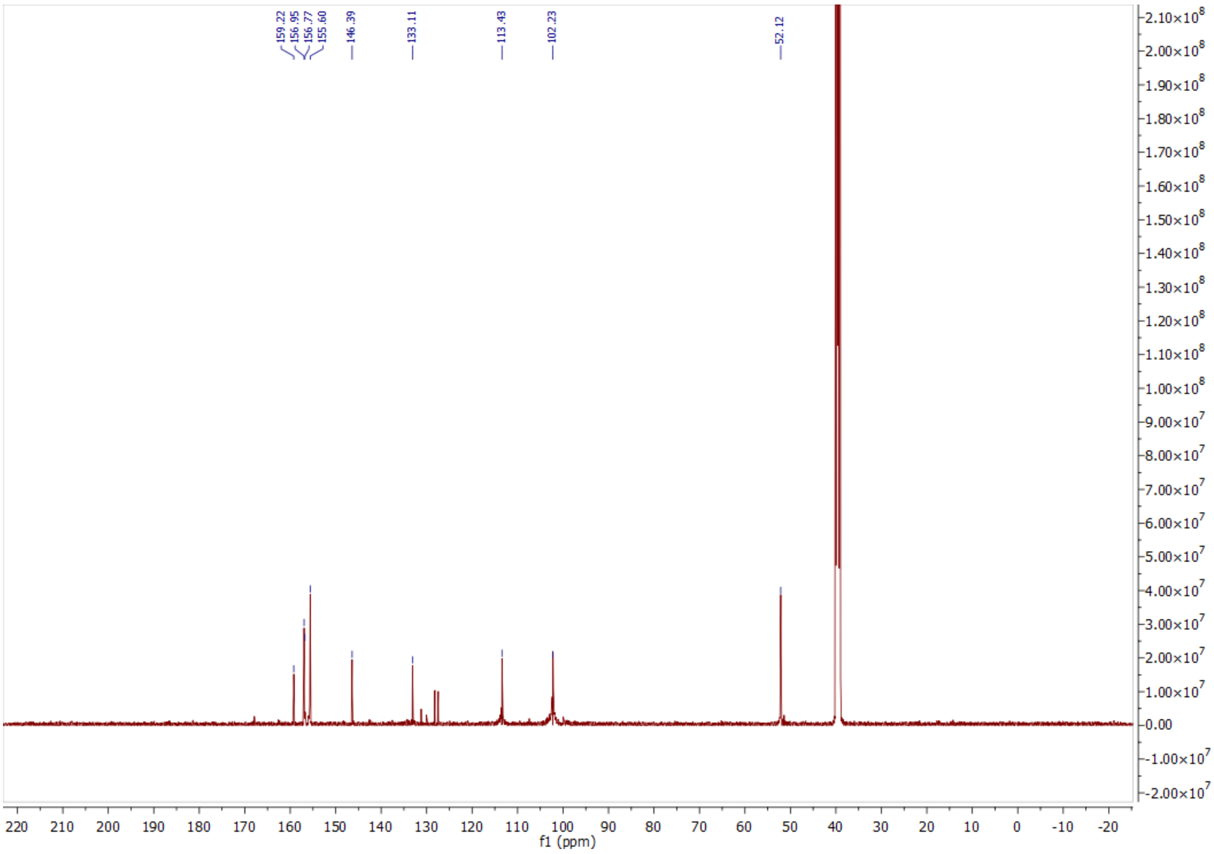

^{1}H and ^{13}C NMR data of compound **20**.

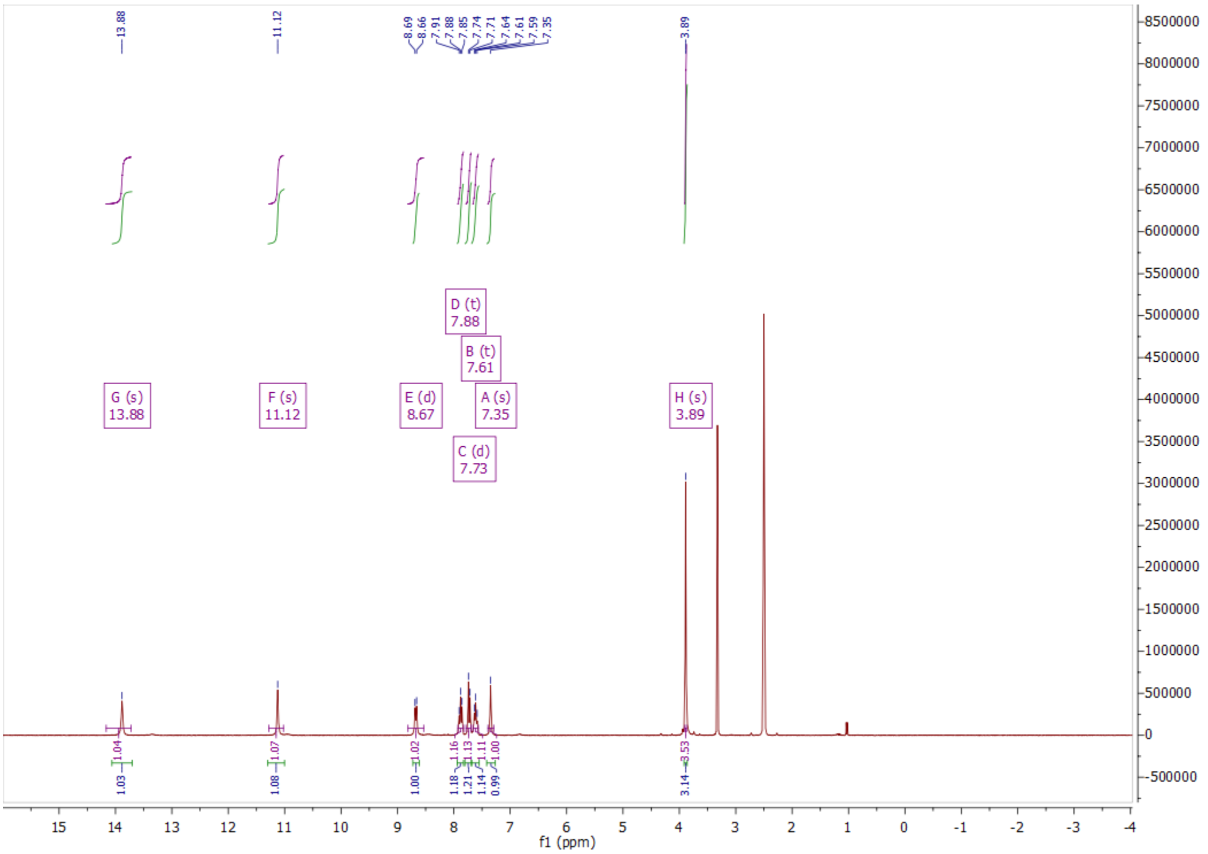

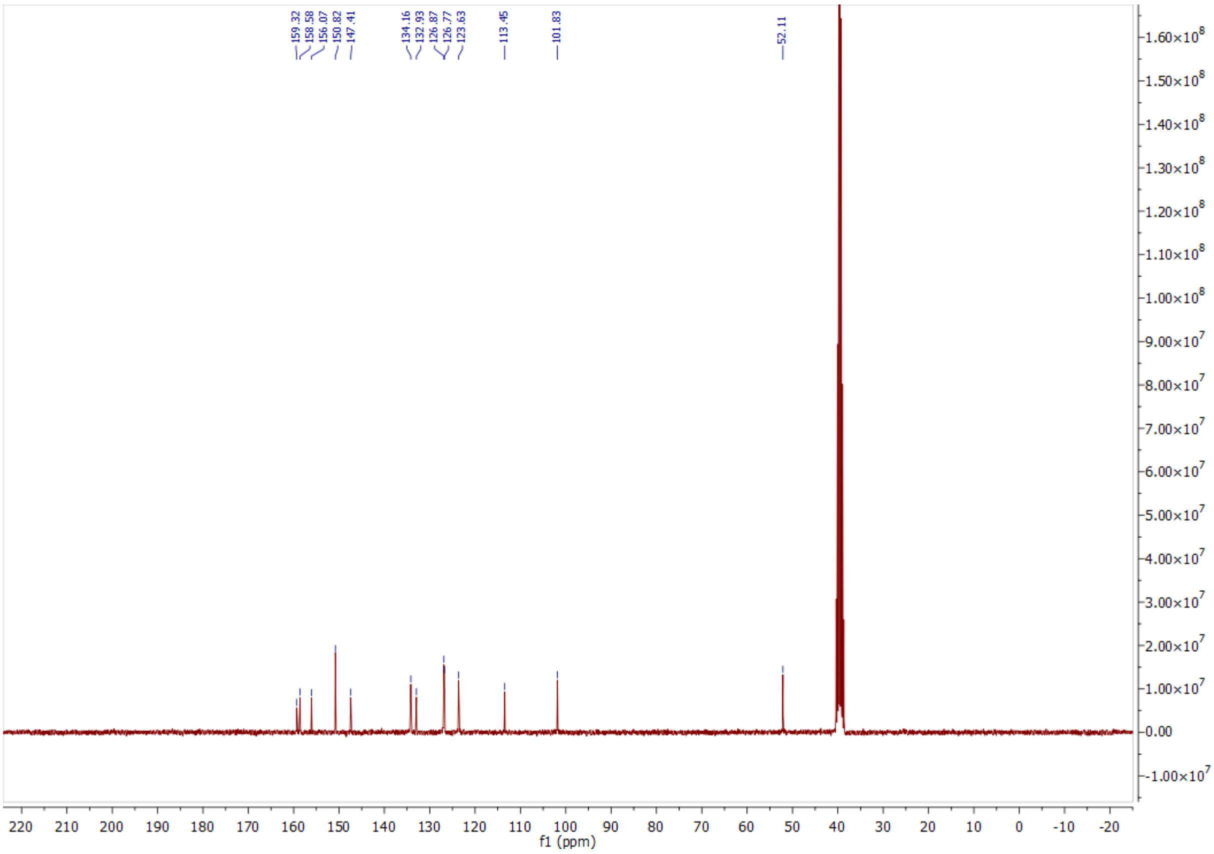

^{1}H, ^{13}C NMR and HPLC data of compound **21a**.

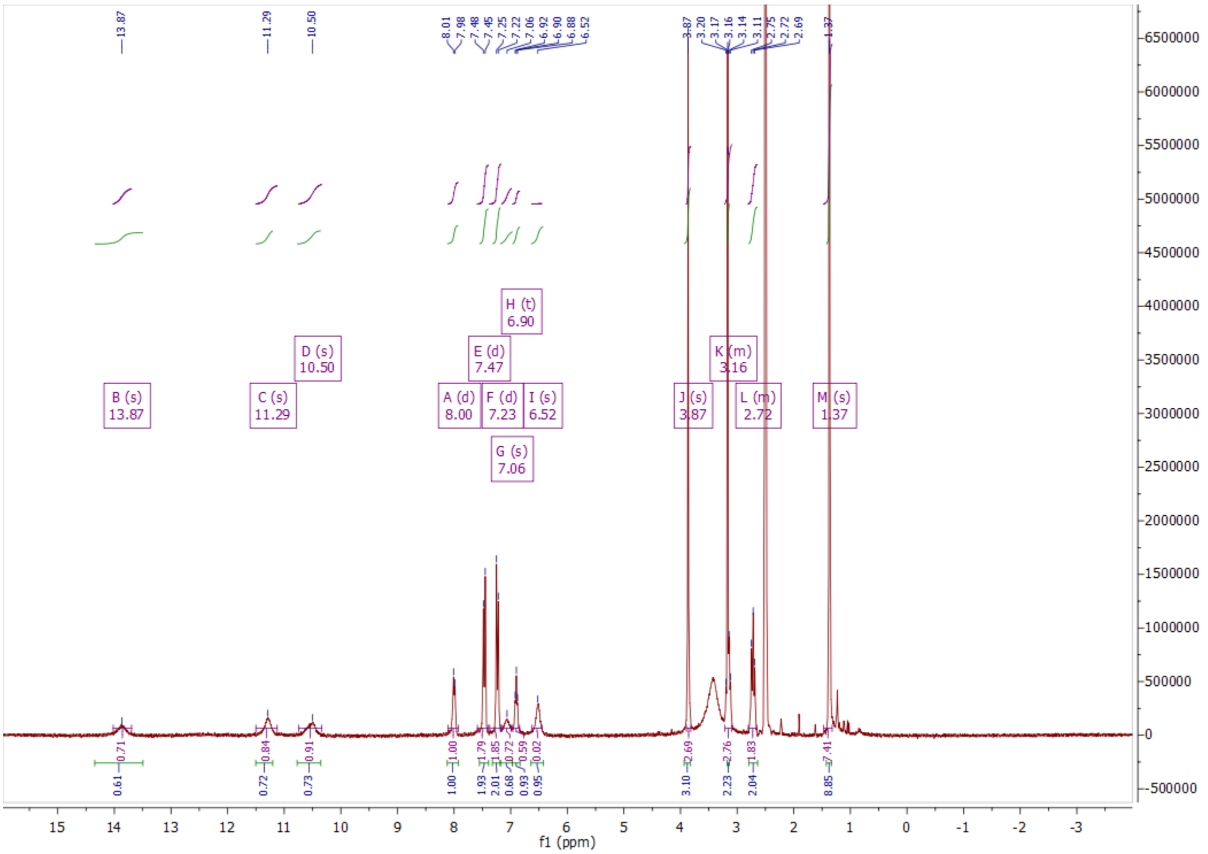

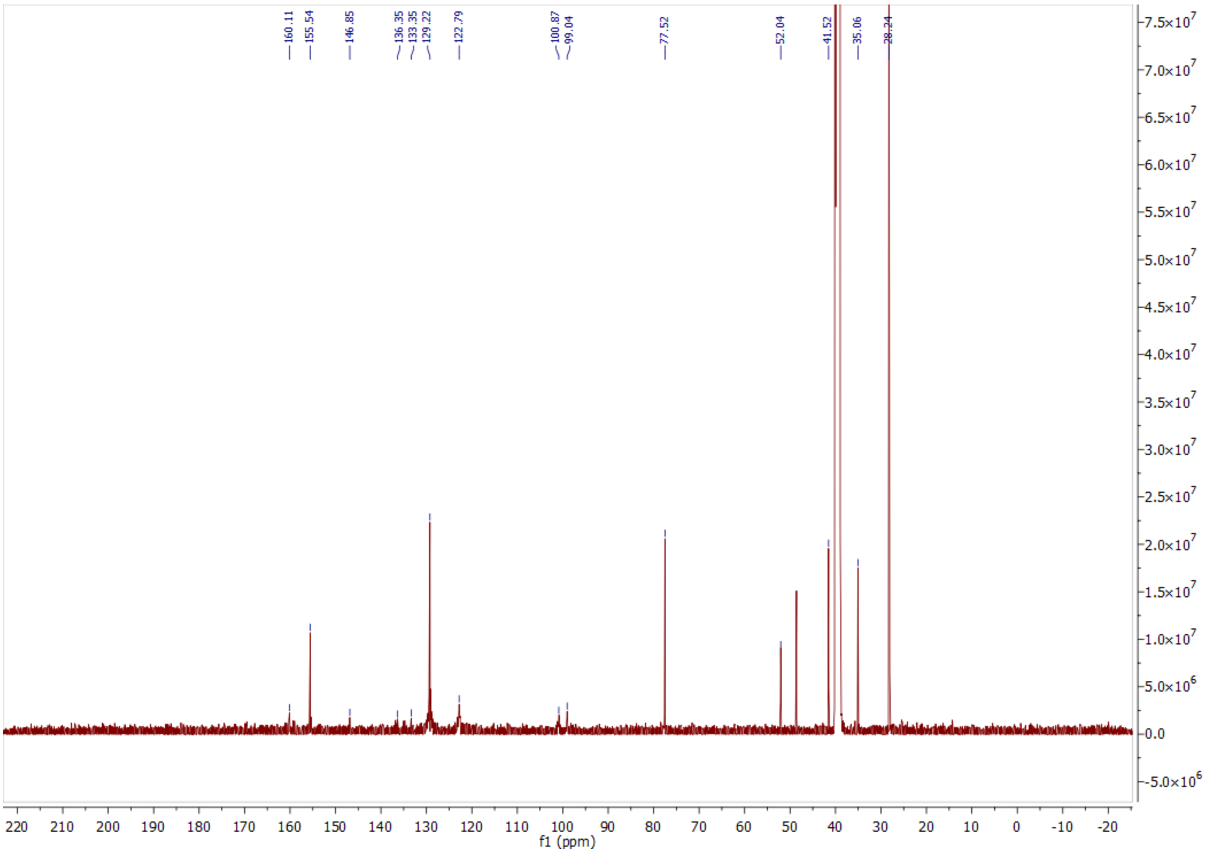

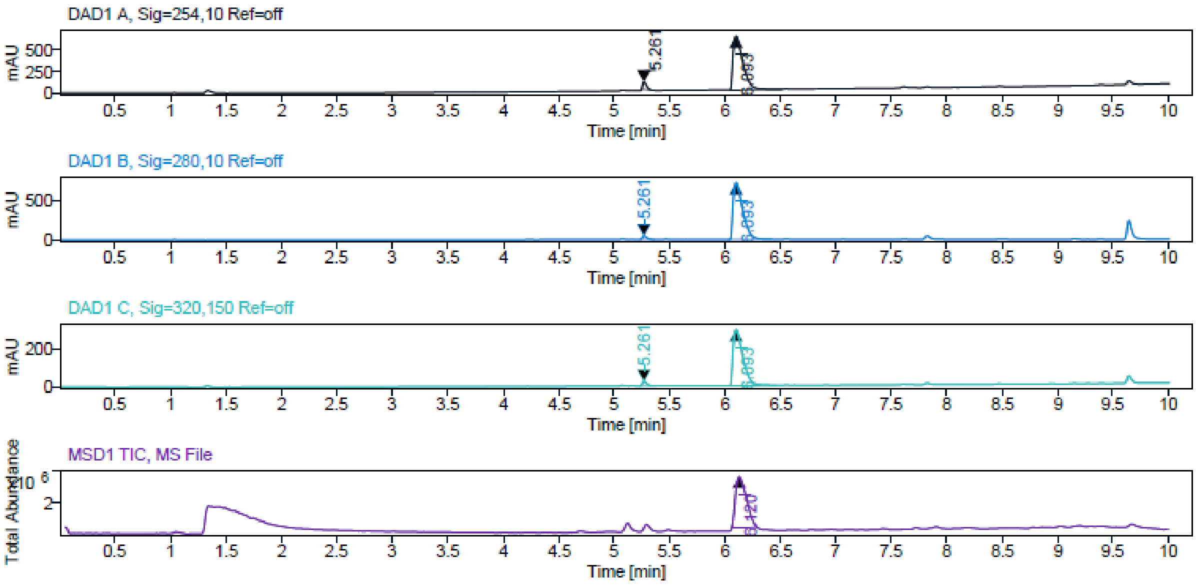

Sample Purity

Signal Description DAD1 A, Sig=254,10 Ref=off

Sample Name	Name	RT	Width	Area	Area%	Height
JA86		5.261	0.037	203.0431	4.92	89.4782
JA86		6.093	0.093	3921.0303	95.08	629.5381

Max Area% 95.077

UV Signal Purity>95% Pass

Signal Description DAD1 B, Sig=280,10 Ref=off

Sample Name	Name	RT	Width	Area	Area%	Height
JA86		5.261	0.040	122.6163	2.71	46.4638
JA86		6.093	0.093	4409.0713	97.29	732.4024

Max Area% 97.294

UV Signal Purity>95% Pass

Signal Description DAD1 C, Sig=320,150 Ref=off

Sample Name	Name	RT	Width	Area	Area%	Height
JA86		5.261	0.041	68.8697	3.56	24.5446
JA86		6.093	0.093	1865.4775	96.44	307.2874

Max Area% 96.440

UV Signal Purity>95% Pass

^{1}H, ^{13}C NMR and HPLC data of compound **21b**.

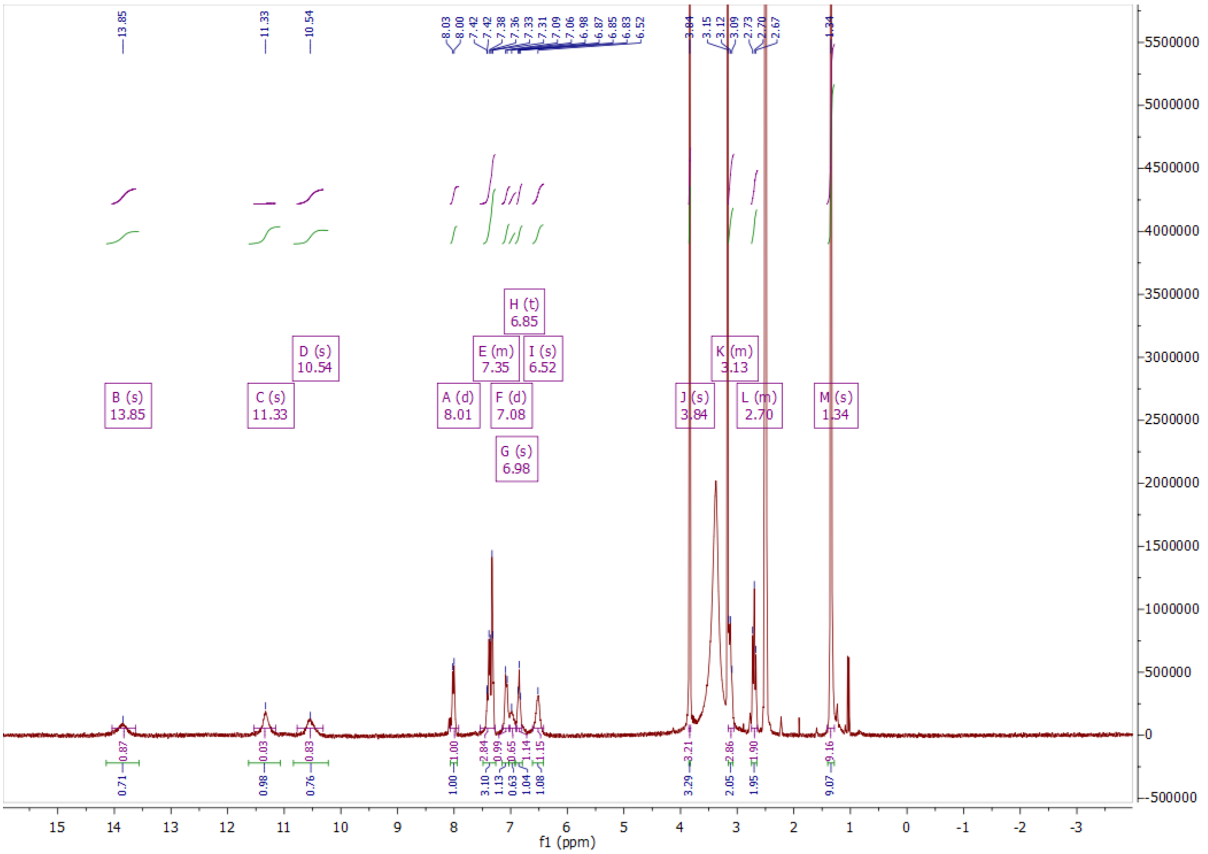

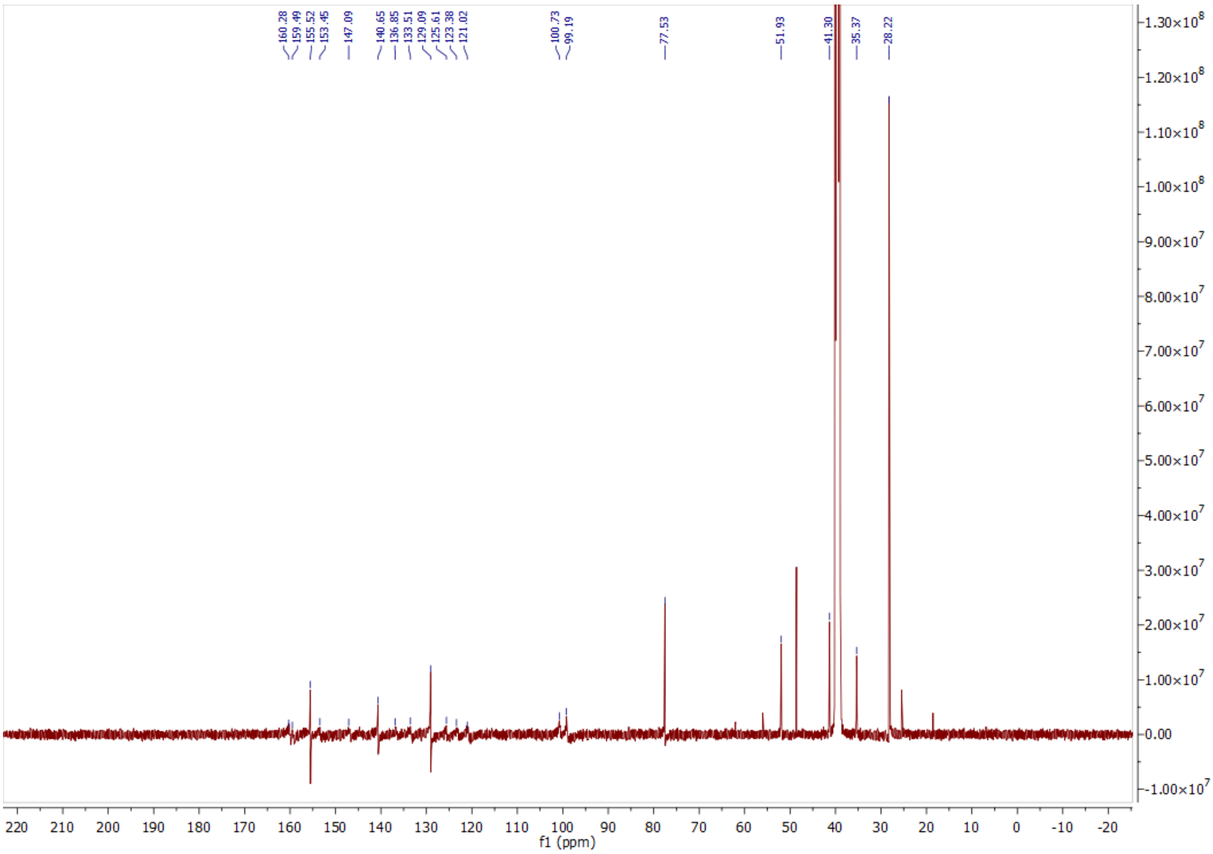

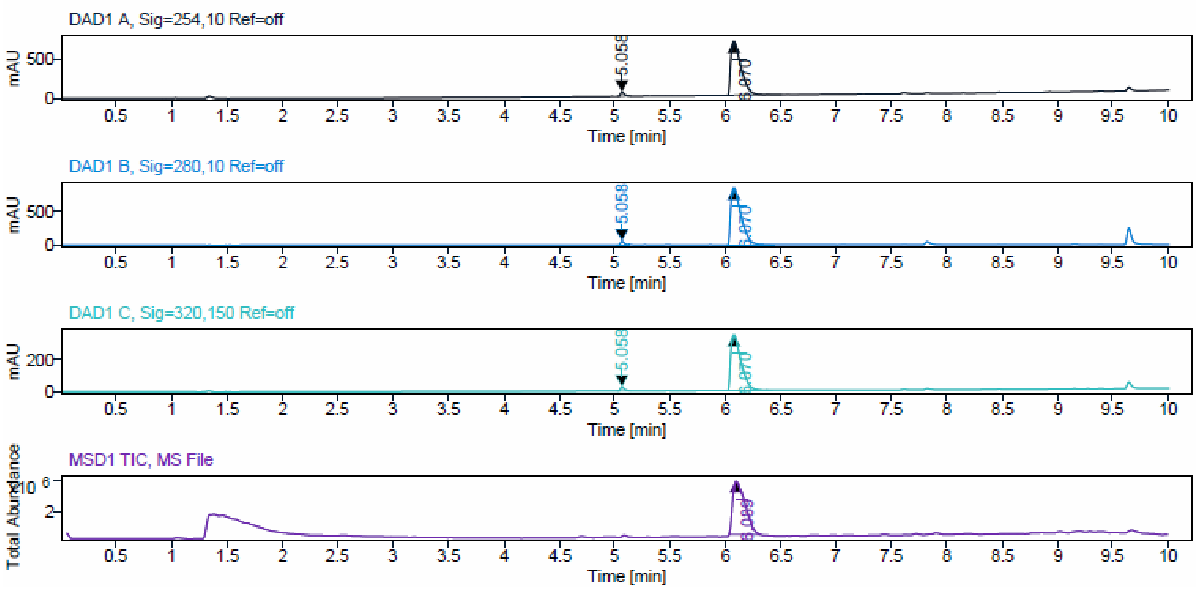

Sample Purity

Signal Description DAD1 A, Sig=254,10 Ref=off

Sample Name	Name	RT	Width	Area	Area%	Height
JA119		5.058	0.034	126.3551	2.73	58.4173
JA119		6.070	0.100	4507.8599	97.27	702.8458

Max Area% 97.273

UV Signal Purity>95% Pass

Signal Description DAD1 B, Sig=280,10 Ref=off

Sample Name	Name	RT	Width	Area	Area%	Height
JA119		5.058	0.033	119.3543	2.15	54.4841
JA119		6.070	0.099	5443.5239	97.85	838.2568

Max Area% 97.854

UV Signal Purity>95% Pass

Signal Description DAD1 C, Sig=320,150 Ref=off

Sample Name	Name	RT	Width	Area	Area%	Height
JA119		5.058	0.035	63.1891	2.70	27.0492
JA119		6.070	0.100	2277.7769	97.30	352.8789

Max Area% 97.301

UV Signal Purity>95% Pass

^{1}H, ^{13}C NMR and HPLC data of compound **21c**.

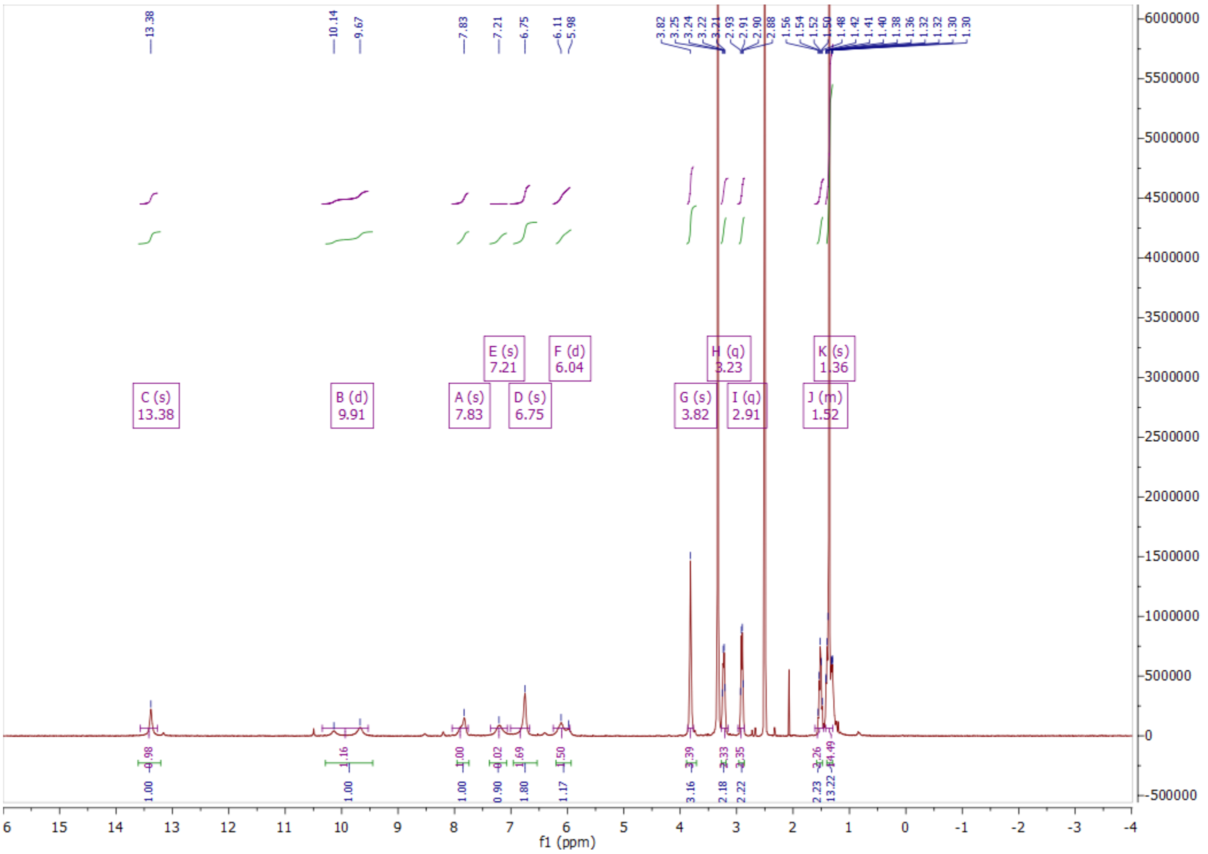

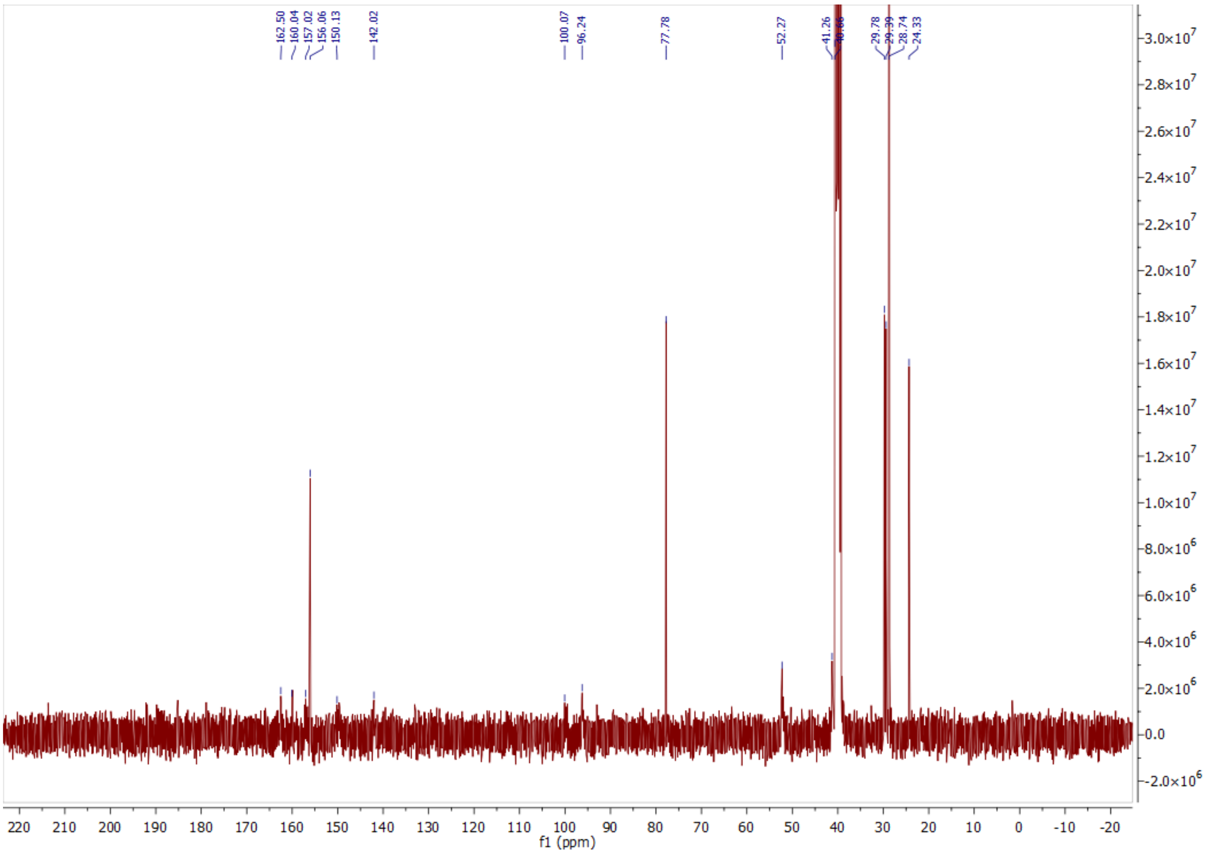

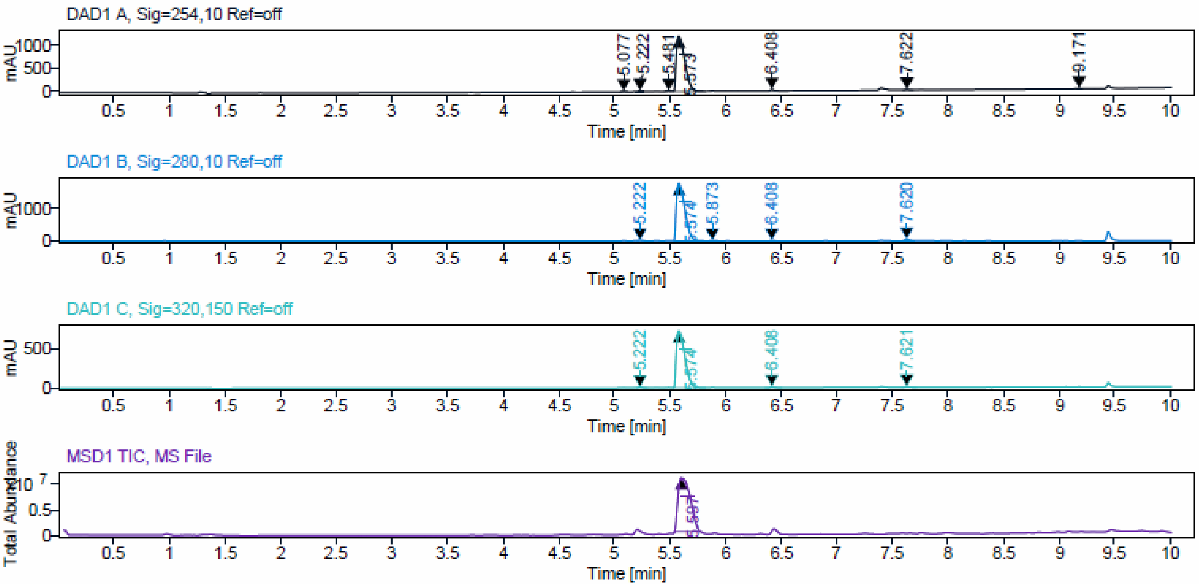

Sample Purity

Signal Description DAD1 A, Sig=254,10 Ref=off

Sample Name	Name	RT	Width	Area	Area%	Height
JA103_prep_f14		5.077	0.045	38.5699	0.59	15.0074
JA103_prep_f14		5.222	0.037	45.0486	0.69	20.1107
JA103_prep_f14		5.481	0.051	35.7459	0.54	12.2971
JA103_prep_f14		5.573	0.085	6298.5713	95.97	1187.9170
JA103_prep_f14		6.408	0.032	66.7007	1.02	31.0731
JA103_prep_f14		7.622	0.051	45.3789	0.69	13.4217
JA103_prep_f14		9.171	0.045	33.1855	0.51	13.6909

Max Area% 95.968

UV Signal Purity>95% Pass

Signal Description DAD1 B, Sig=280,10 Ref=off

Sample Name	Name	RT	Width	Area	Area%	Height
JA103_prep_f14		5.222	0.033	34.3728	0.35	16.0165
JA103_prep_f14		5.574	0.085	9455.1982	97.52	1790.9185
JA103_prep_f14		5.873	0.031	23.3287	0.24	10.3943
JA103_prep_f14		6.408	0.032	34.2773	0.35	14.8231

Sample Name	Name	RT	Width	Area	Area%	Height
JA103_prep_f14		7.620	0.042	148.5687	1.53	53.3182

Max Area%	97.519
UV Signal Purity>95%	Pass
Signal Description	DAD1 C, Sig=320,150 Ref=off

Sample Name	Name	RT	Width	Area	Area%	Height
JA103_prep_f14		5.222	0.033	16.8287	0.44	7.7458
JA103_prep_f14		5.574	0.086	3789.2156	98.13	718.6230
JA103_prep_f14		6.408	0.033	28.5044	0.74	12.9056
JA103_prep_f14		7.621	0.042	26.7143	0.69	9.4558

Max Area%	98.134
UV Signal Purity>95%	Pass

^{1}H, ^{13}C NMR and HPLC data of compound **21d**.

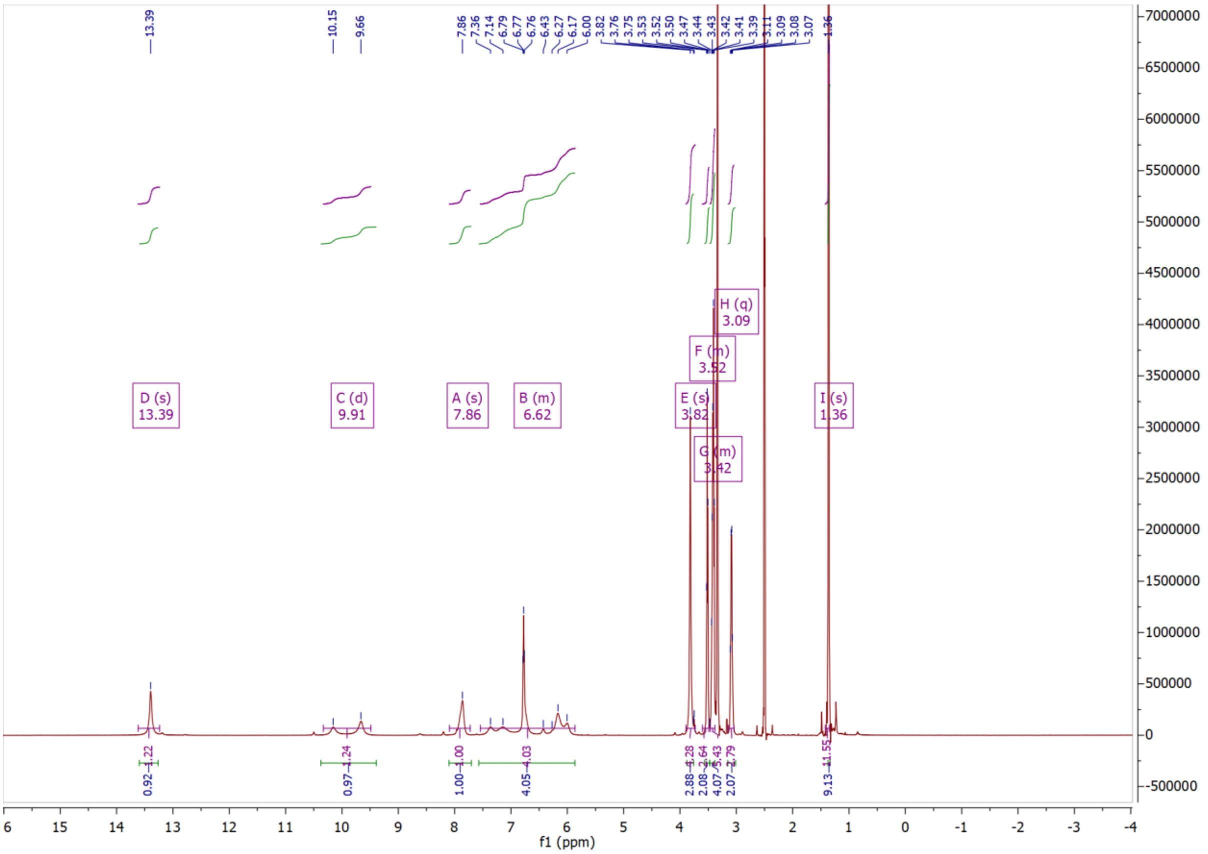

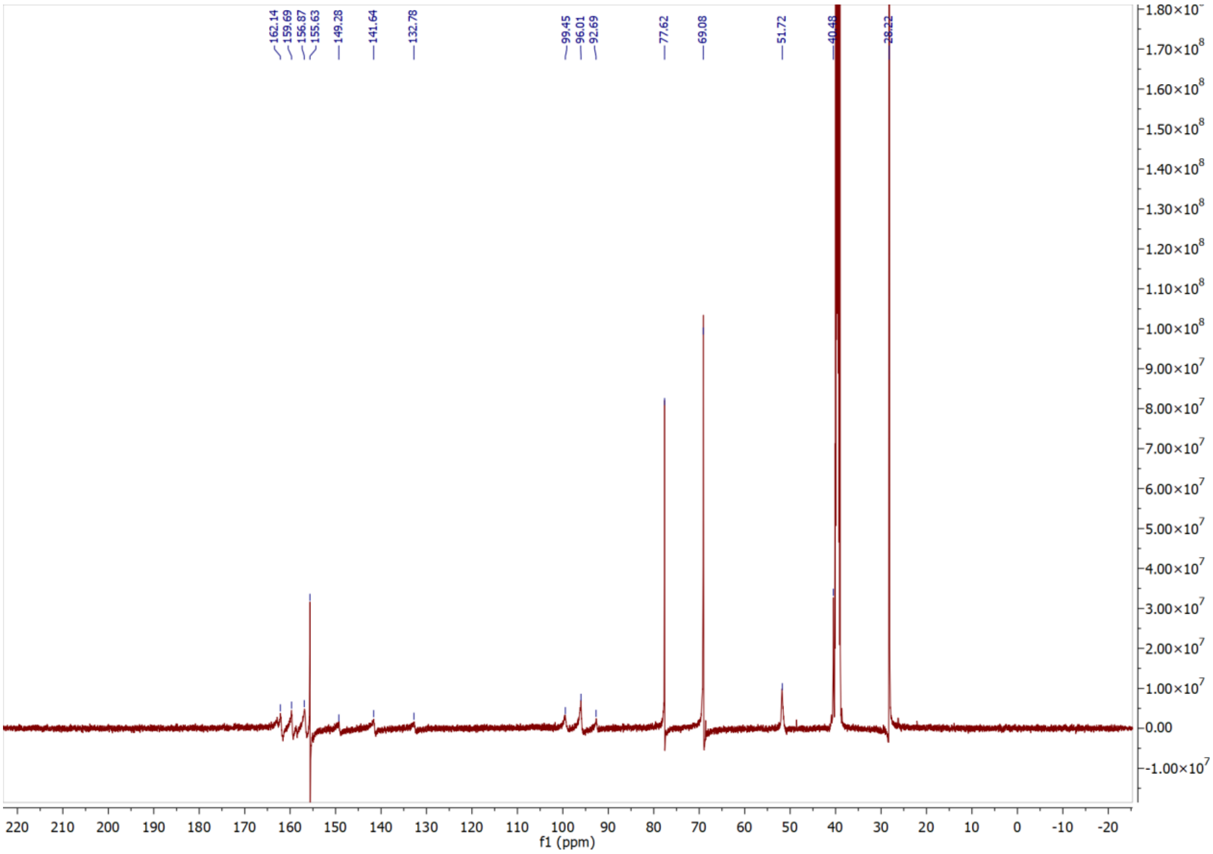

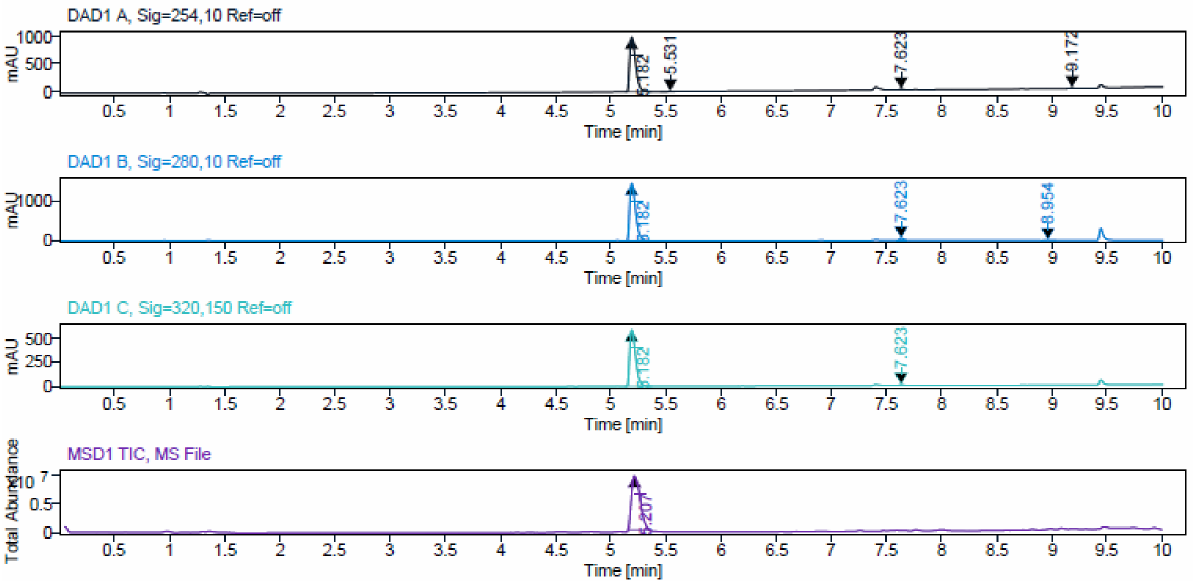

Sample Purity

Signal Description	DAD1 A, Sig=254,10 Ref=off

Sample Name	Name	RT	Width	Area	Area%	Height
JA107_prepF1		5.182	0.059	3622.2283	98.01	971.3520
JA107_prepF1		5.531	0.037	15.2247	0.41	6.1775
JA107_prepF1		7.623	0.043	31.5078	0.85	11.2794
JA107_prepF1		9.172	0.036	26.8967	0.73	11.0198

Max Area%	98.008
UV Signal Purity>95%	Pass
Signal Description	DAD1 B, Sig=280,10 Ref=off

Sample Name	Name	RT	Width	Area	Area%	Height
JA107_prepF1		5.182	0.059	5476.6074	97.28	1467.6888
JA107_prepF1		7.623	0.040	127.1096	2.26	50.6332
JA107_prepF1		8.954	0.038	25.9763	0.46	10.3502

Max Area%	97.281
UV Signal Purity>95%	Pass

Signal Description	DAD1 C, Sig=320,150 Ref=off

Sample Name	Name	RT	Width	Area	Area%	Height
JA107_prepF1		5.182	0.059	2234.7620	98.84	598.5150
JA107_prepF1		7.623	0.043	26.2908	1.16	9.6527

Max Area%	98.837
UV Signal Purity>95%	Pass

^{1}H, ^{13}C NMR and HPLC data of compound **21e**.

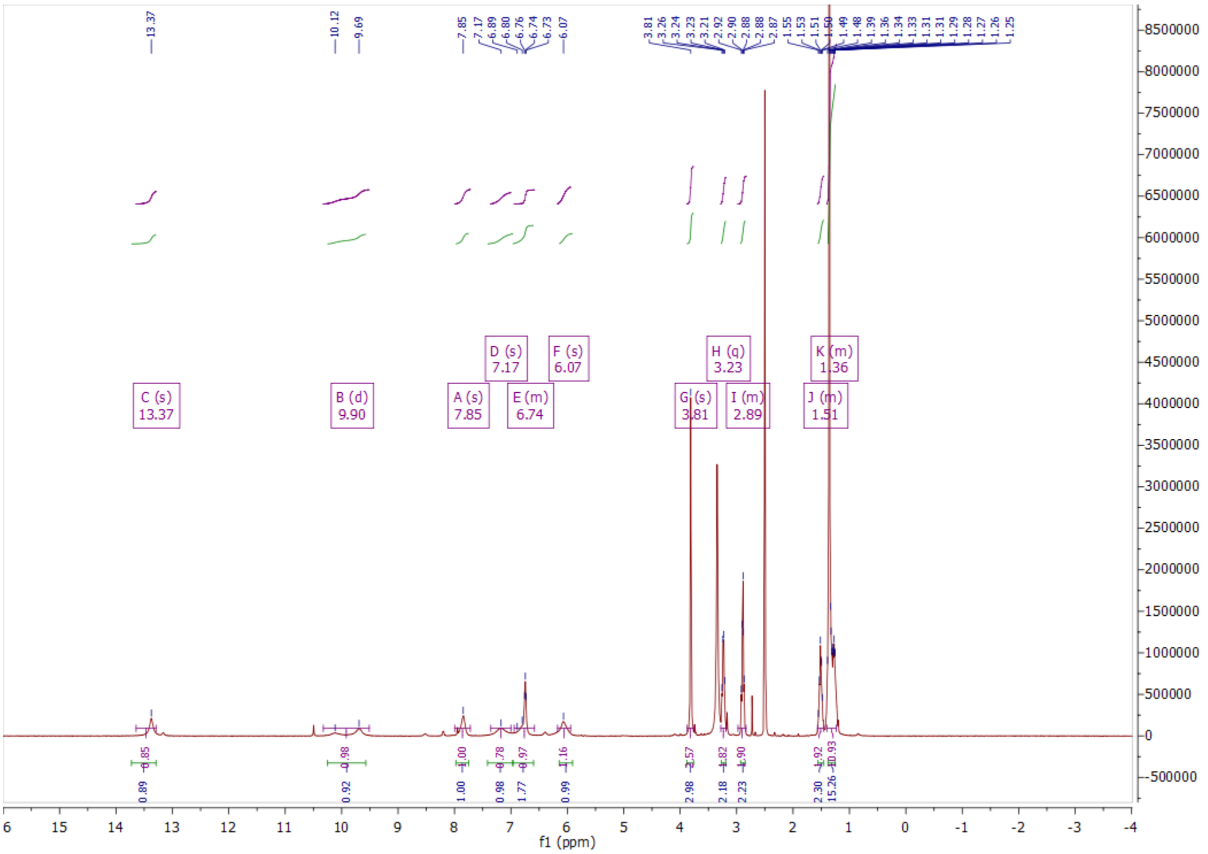

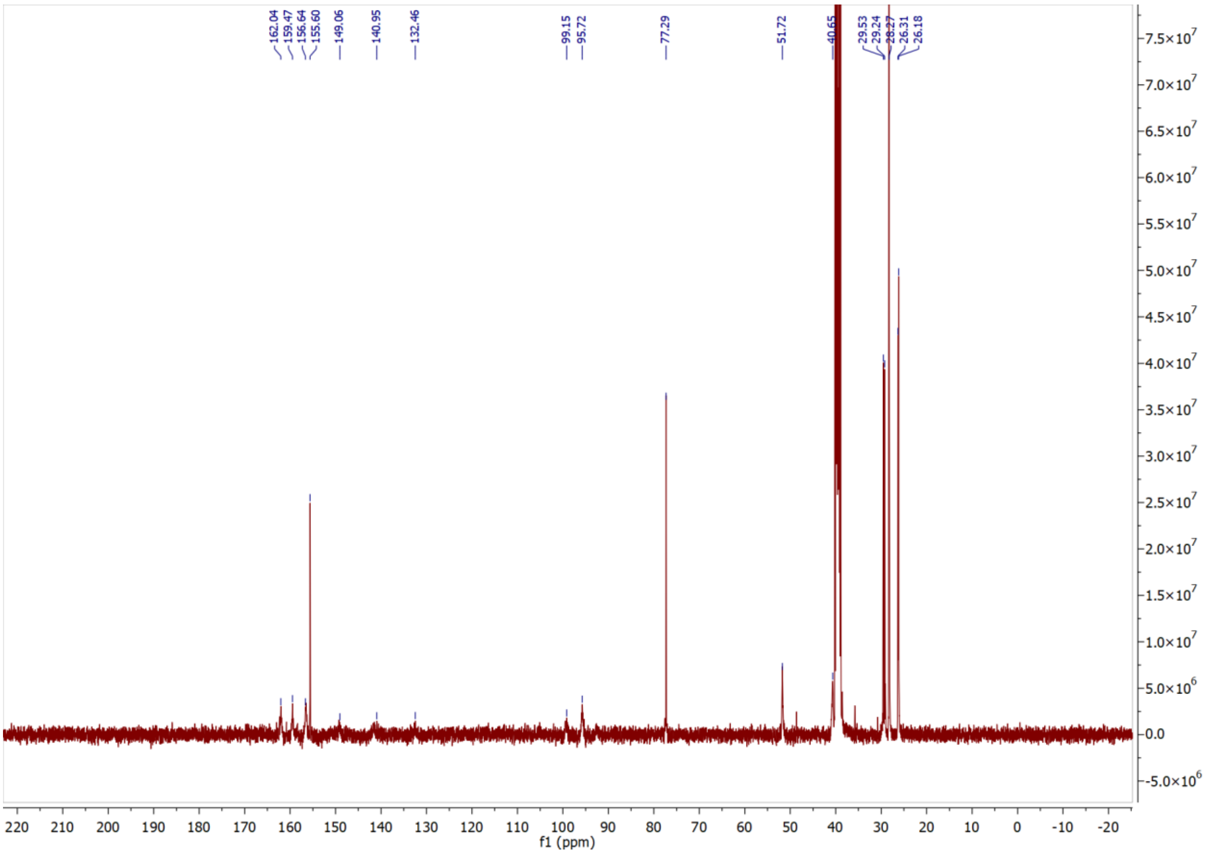

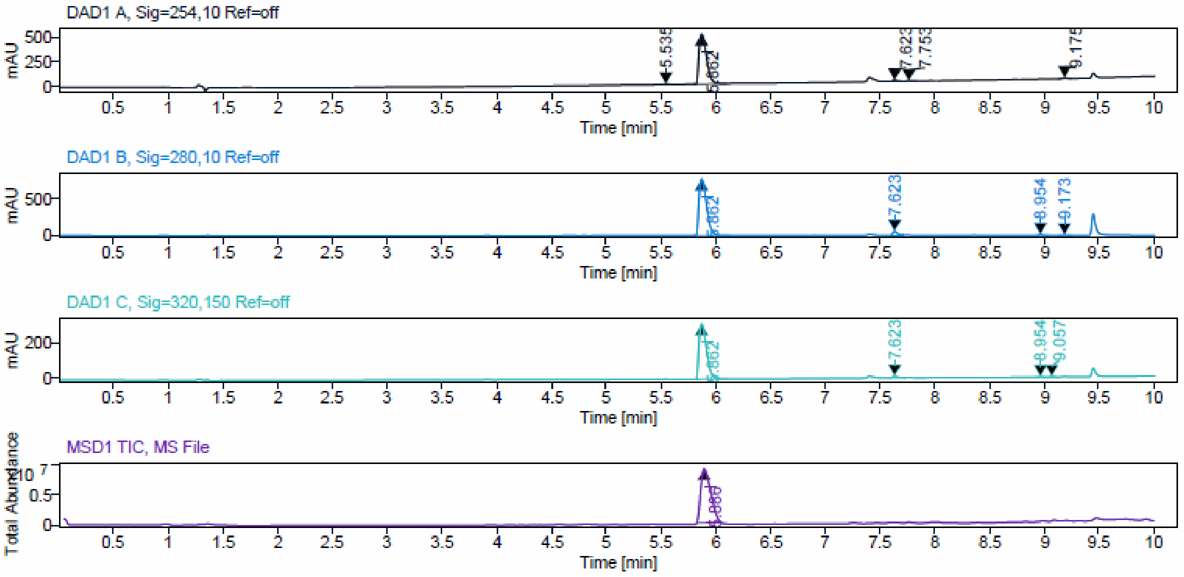

Sample Purity

Signal Description DAD1 A, Sig=254,10 Ref=off

Sample Name	Name	RT	Width	Area	Area%	Height
JA104_prep_F16		5.535	0.043	12.0778	0.49	4.3697
JA104_prep_F16		5.862	0.077	2387.1487	96.52	503.9031
JA104_prep_F16		7.623	0.044	31.3098	1.27	10.9434
JA104_prep_F16		7.753	0.043	12.9726	0.52	4.7900
JA104_prep_F16		9.175	0.038	29.5919	1.20	11.8123

Max Area% 96.525

UV Signal Purity>95% Pass

Signal Description DAD1 B, Sig=280,10 Ref=off

Sample Name	Name	RT	Width	Area	Area%	Height
JA104_prep_F16		5.862	0.075	3681.2483	95.67	775.2848
JA104_prep_F16		7.623	0.042	134.1460	3.49	49.4574
JA104_prep_F16		8.954	0.039	23.7454	0.62	9.2409
JA104_prep_F16		9.173	0.041	8.7145	0.23	2.9845

Max Area% 95.670

UV Signal Purity>95% Pass

Signal Description DAD1 C, Sig=320,150 Ref=off

Sample Name	Name	RT	Width	Area	Area%	Height
JA104_prep_F16		5.862	0.076	1472.0709	97.47	310.0414
JA104_prep_F16		7.623	0.044	26.6122	1.76	9.2569
JA104_prep_F16		8.954	0.041	6.2512	0.41	2.4823
JA104_prep_F16		9.057	0.034	5.3317	0.35	2.4078

Max Area% 97.471

UV Signal Purity>95% Pass

^{1}H, ^{13}C NMR and HPLC data of compound **21f**.

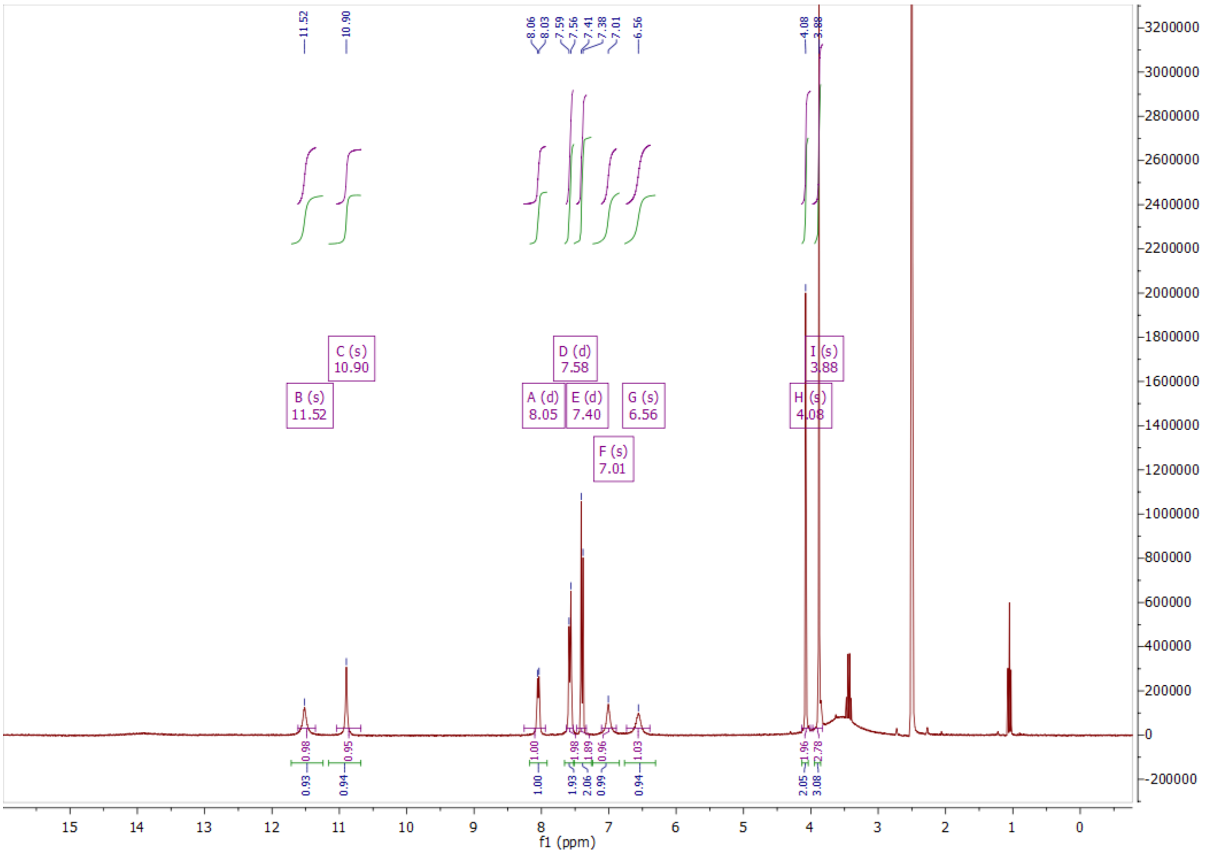

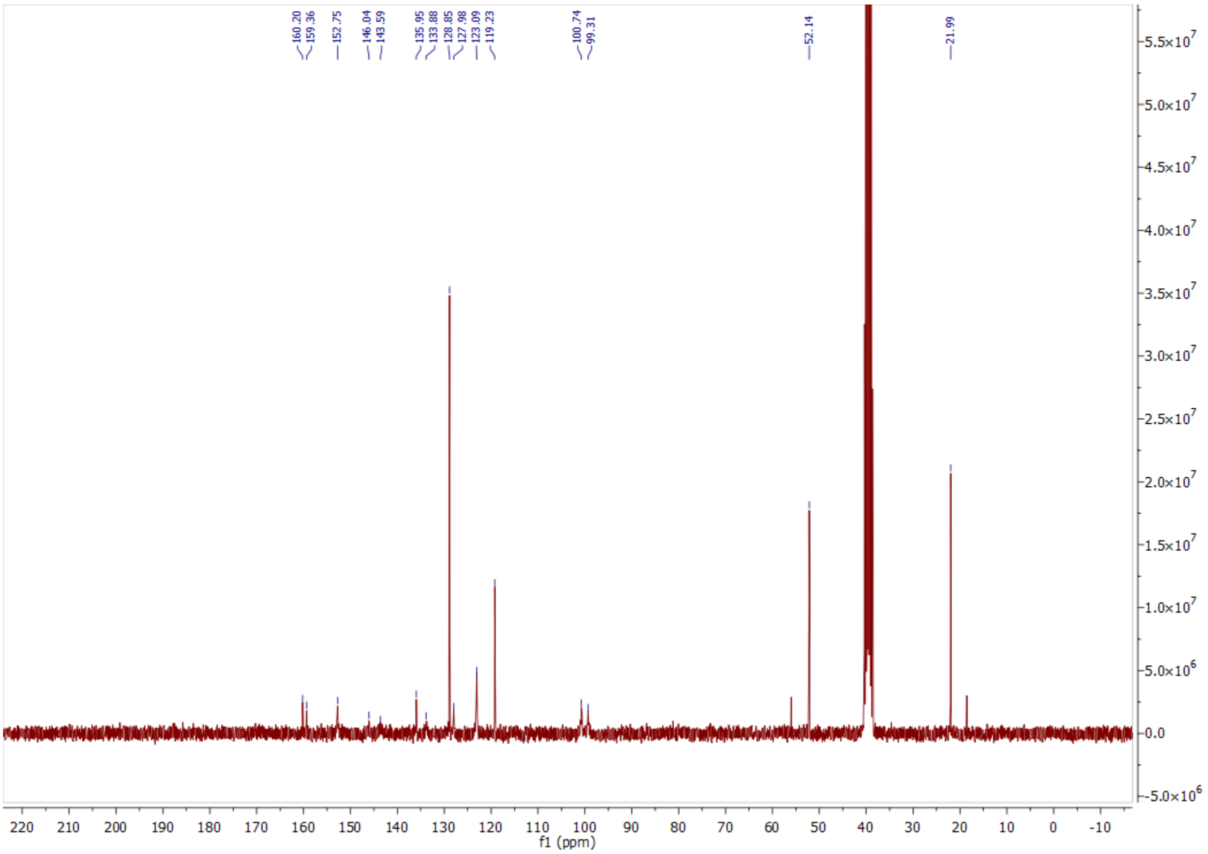

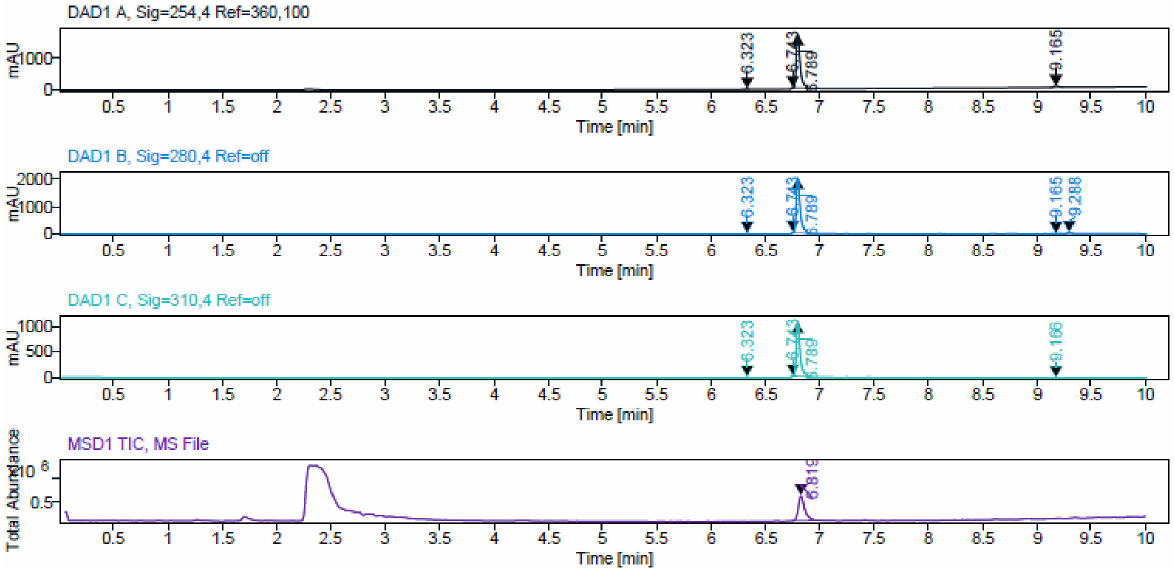

Sample Purity

Signal Description DAD1 A, Sig=254,4 Ref=360,100

Sample Name	Name	RT	Width	Area	Area%	Height
JA295_Feststoff		6.323	0.034	37.3741	0.82	19.1438
JA295_Feststoff		6.743	0.020	31.0171	0.68	26.3949
JA295_Feststoff		6.789	0.037	4313.3276	95.20	1721.5618
JA295_Feststoff		9.165	0.050	149.0728	3.29	48.0904

Max Area% 95.200

UV Signal Purity>95% Pass

Signal Description DAD1 B, Sig=280,4 Ref=off

Sample Name	Name	RT	Width	Area	Area%	Height
JA295_Feststoff		6.323	0.025	21.9729	0.40	15.4297
JA295_Feststoff		6.743	0.018	27.2132	0.50	24.6035
JA295_Feststoff		6.789	0.037	5214.5894	95.81	2069.7368
JA295_Feststoff		9.165	0.058	50.3329	0.92	18.2488
JA295_Feststoff		9.288	0.047	128.3314	2.36	44.2918

Max Area% 95.813

UV Signal Purity>95% Pass

Signal Description DAD1 C, Sig=310,4 Ref=off

Sample Name	Name	RT	Width	Area	Area%	Height
JA295_Feststoff		6.323	0.028	18.5313	0.68	9.5718
JA295_Feststoff		6.743	0.019	17.7016	0.65	15.3087
JA295_Feststoff		6.789	0.037	2693.4287	98.45	1078.4799
JA295_Feststoff		9.166	0.051	6.1711	0.23	1.7122

Max Area% 98.450

UV Signal Purity>95% Pass

^{1}H, ^{13}C NMR and HPLC data of compound **21g**.

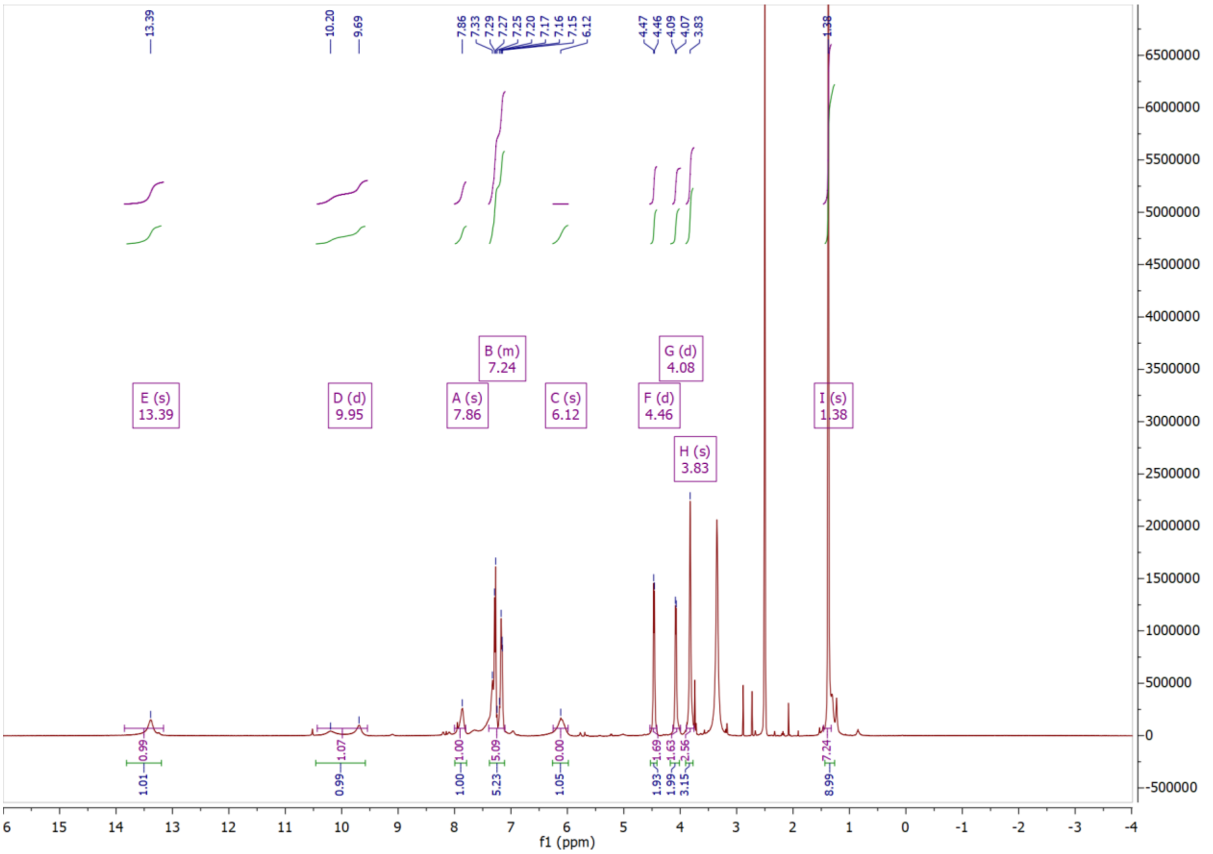

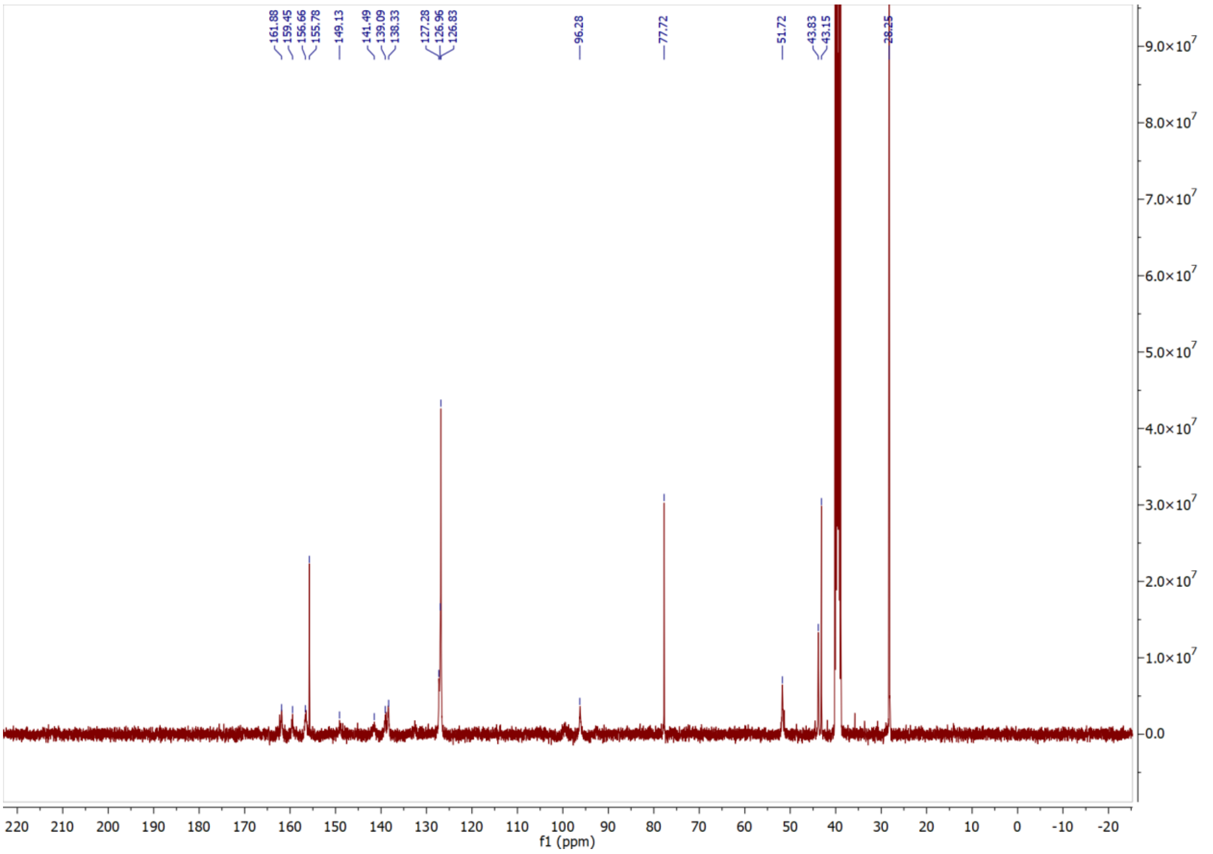

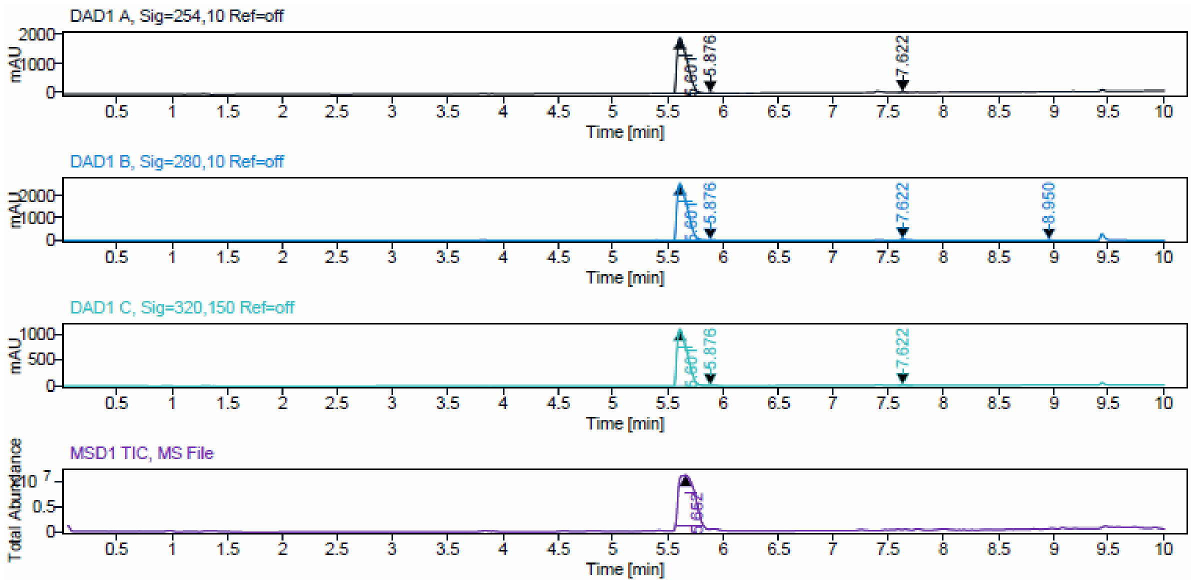

Sample Purity

Signal Description DAD1 A, Sig=254,10 Ref=off

Sample Name	Name	RT	Width	Area	Area%	Height
JA105_prep_f44		5.601	0.106	12181.84 08	99.31	1880.5212
JA105_prep_f44		5.876	0.030	34.6442	0.28	15.1774
JA105_prep_f44		7.622	0.057	50.4412	0.41	15.6378

Max Area% 99.306

UV Signal Purity>95% Pass

Signal Description DAD1 B, Sig=280,10 Ref=off

Sample Name	Name	RT	Width	Area	Area%	Height
JA105_prep_f44		5.601	0.106	17073.10 55	98.66	2607.9143
JA105_prep_f44		5.876	0.029	34.2587	0.20	14.9622
JA105_prep_f44		7.622	0.040	172.3980	1.00	54.7480
JA105_prep_f44		8.950	0.038	25.0231	0.14	10.0462

Max Area% 98.661

UV Signal Purity>95% Pass

Signal Description DAD1 C, Sig=320,150 Ref=off

Sample Name	Name	RT	Width	Area	Area%	Height
JA105_prep_f44		5.601	0.106	7195.5728	99.44	1105.0964
JA105_prep_f44		5.876	0.029	14.1129	0.20	6.1442
JA105_prep_f44		7.622	0.041	26.4374	0.37	9.5805

Max Area% 99.440

UV Signal Purity>95% Pass

^{1}H, ^{13}C NMR and HPLC data of compound **21h**.

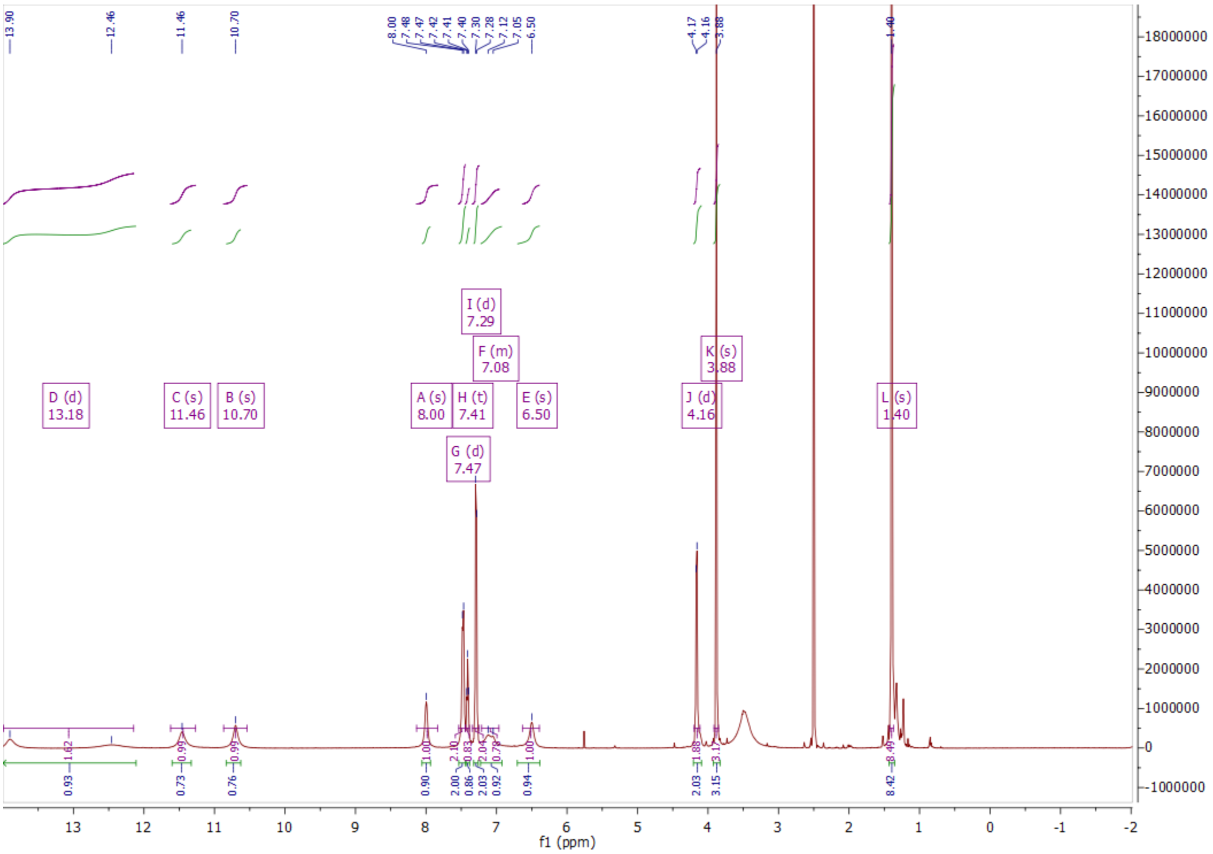

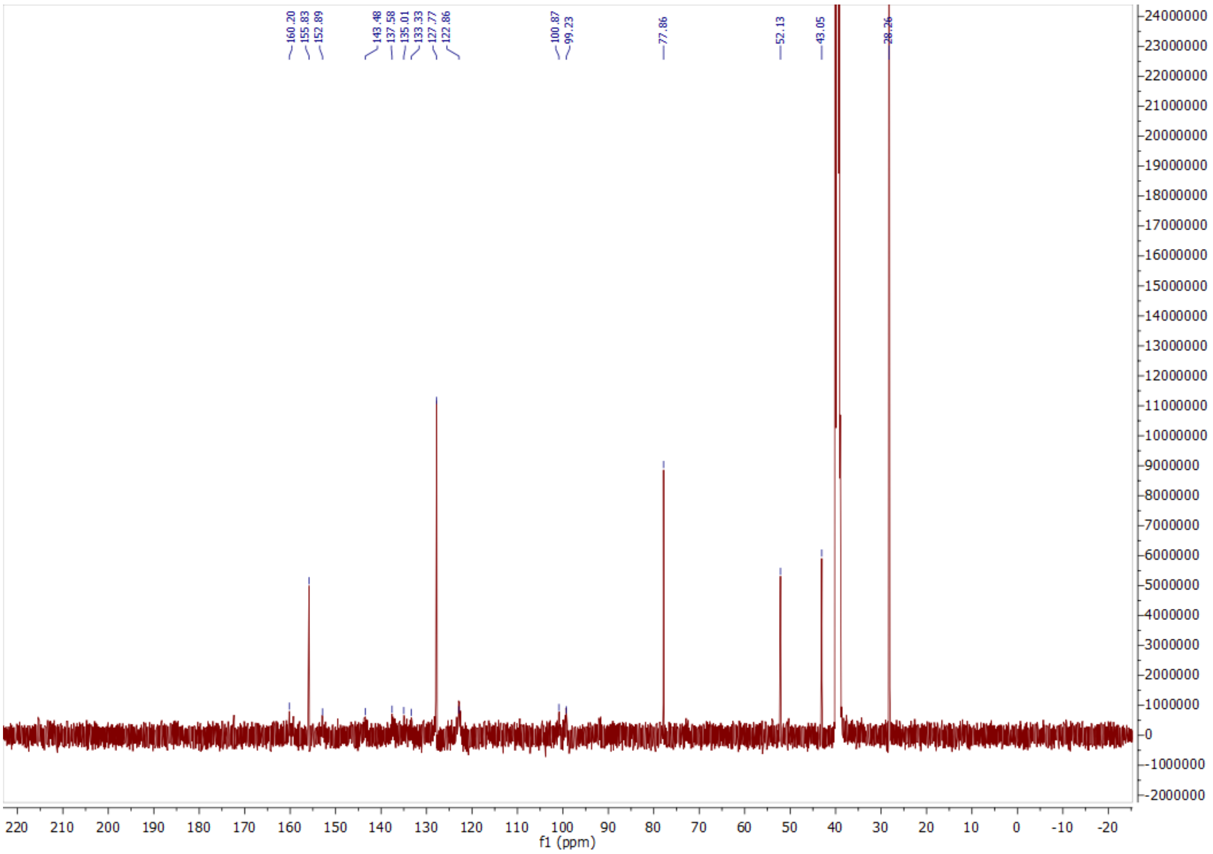

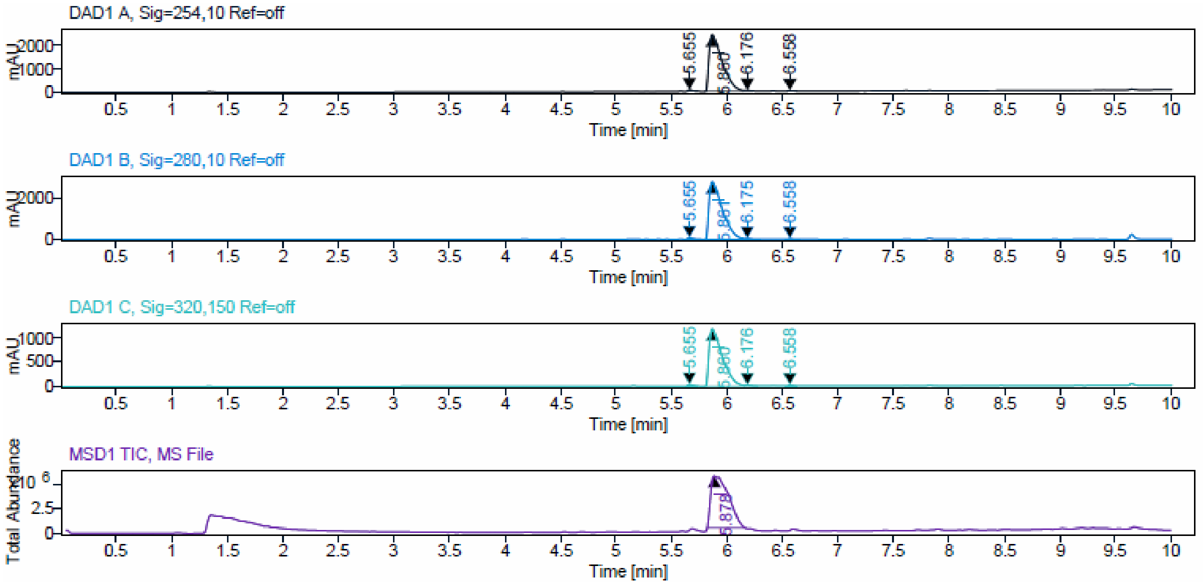

Sample Purity

Signal Description DAD1 A, Sig=254,10 Ref=off

Sample Name	Name	RT	Width	Area	Area%	Height
JA25		5.655	0.047	228.2647	1.11	73.4581
JA25		5.860	0.129	20094.5801	97.97	2433.5793
JA25		6.176	0.066	110.2925	0.54	33.0659
JA25		6.558	0.051	77.4831	0.38	25.5714

Max Area% 97.972

UV Signal Purity>95% Pass

Signal Description DAD1 B, Sig=280,10 Ref=off

Sample Name	Name	RT	Width	Area	Area%	Height
JA25		5.655	0.046	246.5930	1.03	79.9826
JA25		5.861	0.130	23381.5273	97.59	2796.2859
JA25		6.175	0.075	218.0606	0.91	45.3388
JA25		6.558	0.049	111.7577	0.47	34.0448

Max Area% 97.594

UV Signal Purity>95% Pass

Signal Description DAD1 C, Sig=320,150 Ref=off

Sample Name	Name	RT	Width	Area	Area%	Height
JA25		5.655	0.050	113.8086	1.11	35.5694
JA25		5.860	0.129	9936.7734	97.30	1193.9503
JA25		6.176	0.067	91.4905	0.90	22.8523
JA25		6.558	0.050	69.9667	0.69	21.7468

Max Area% 97.304

UV Signal Purity>95% Pass

^{1}H, ^{13}C NMR and HPLC data of compound **21i**.

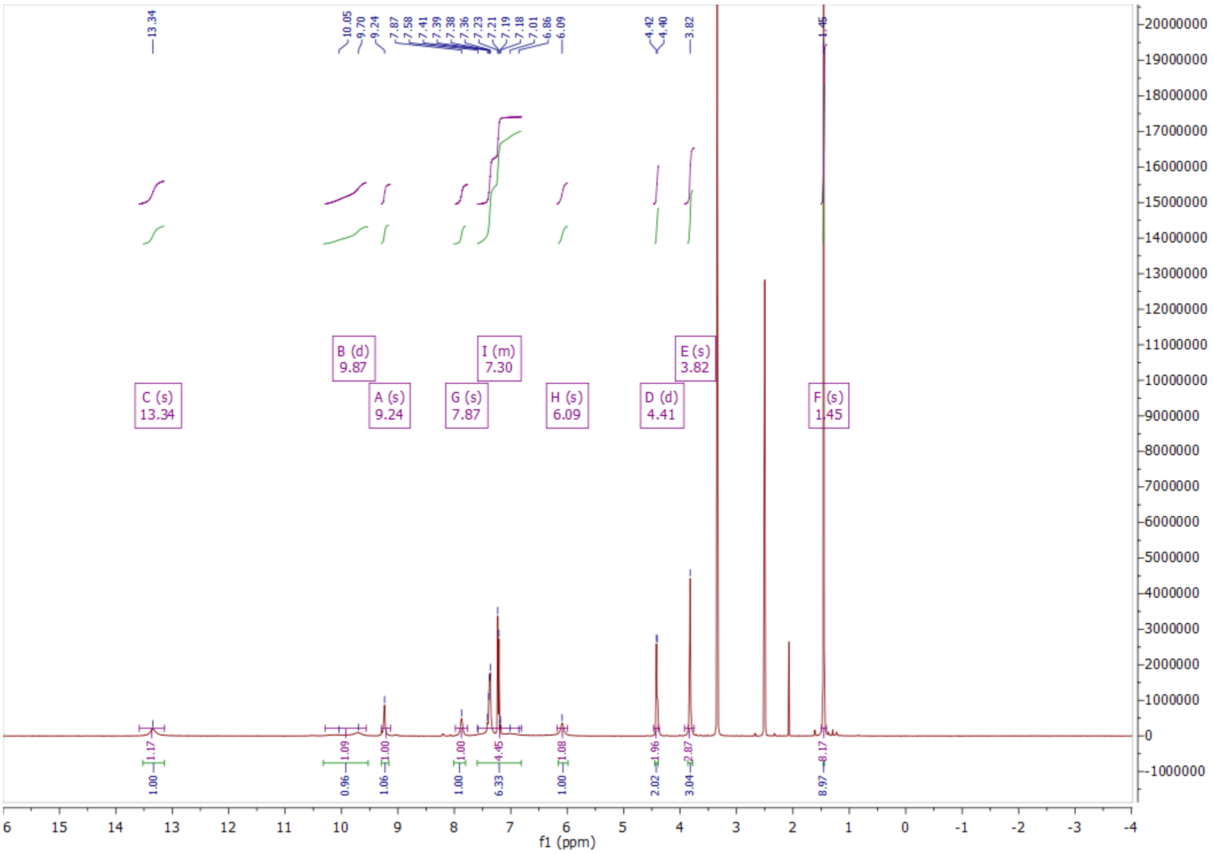

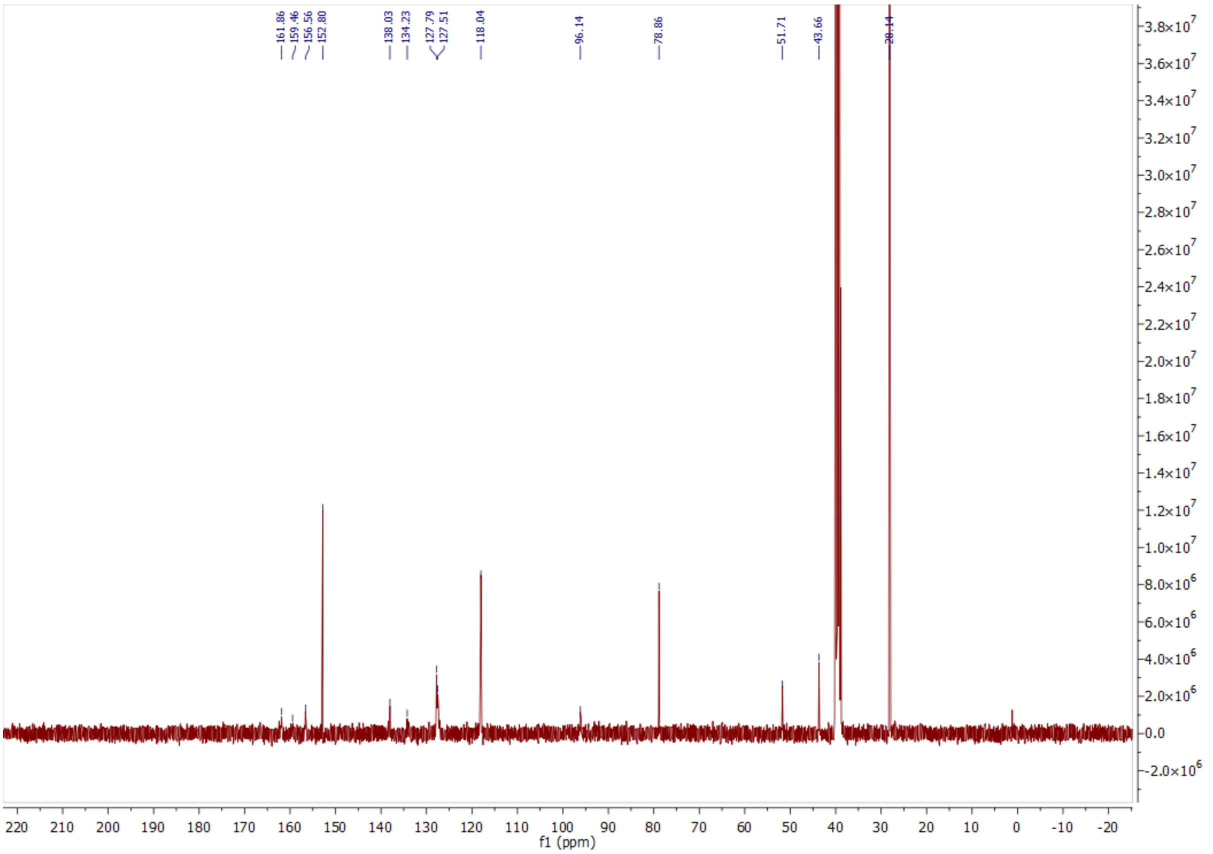

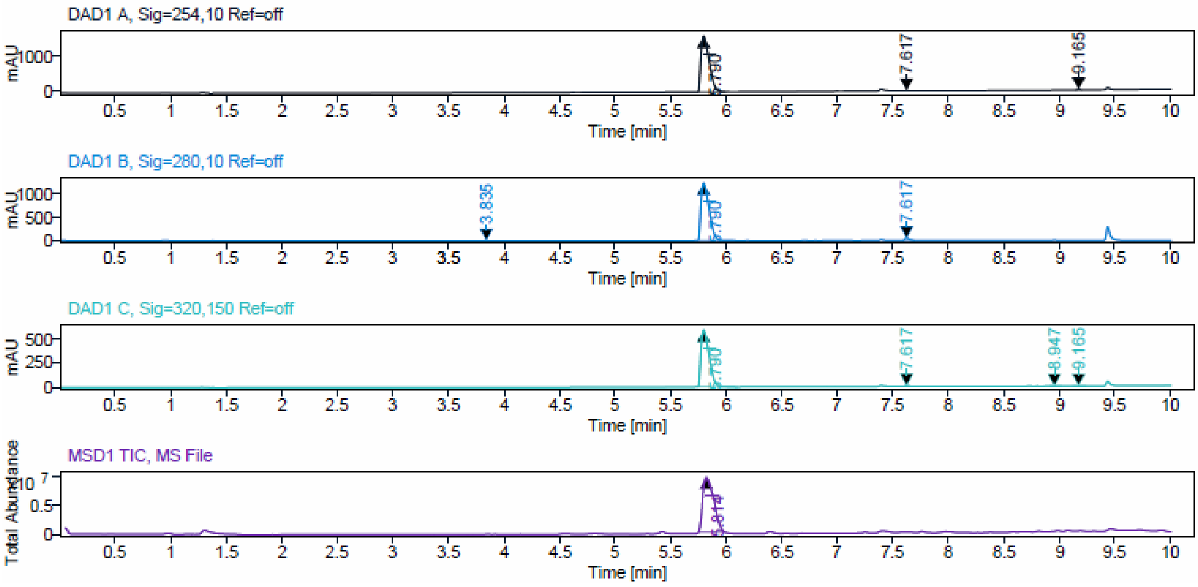

Sample Purity

Signal Description DAD1 A, Sig=254,10 Ref=off

Sample Name	Name	RT	Width	Area	Area%	Height
JA106_F61		5.790	0.084	7655.0088	99.21	1498.8925
JA106_F61		7.617	0.042	31.5055	0.41	11.3986
JA106_F61		9.165	0.042	29.5237	0.38	13.3037

Max Area% 99.209

UV Signal Purity>95% Pass

Signal Description DAD1 B, Sig=280,10 Ref=off

Sample Name	Name	RT	Width	Area	Area%	Height
JA106_F61		3.835	0.042	7.3967	0.11	2.6870
JA106_F61		5.790	0.082	6426.4336	98.03	1264.8546
JA106_F61		7.617	0.038	121.8465	1.86	49.2818

Max Area% 98.029

UV Signal Purity>95% Pass

Signal Description DAD1 C, Sig=320,150 Ref=off

Sample Name	Name	RT	Width	Area	Area%	Height
JA106_F61		5.790	0.082	3055.4397	98.99	599.2926
JA106_F61		7.617	0.036	18.7420	0.61	8.1436
JA106_F61		8.947	0.039	6.7128	0.22	2.7564
JA106_F61		9.165	0.036	5.6560	0.18	2.2742

Max Area% 98.992

UV Signal Purity>95% Pass

^{1}H, ^{13}C NMR and HPLC data of compound **22a**.

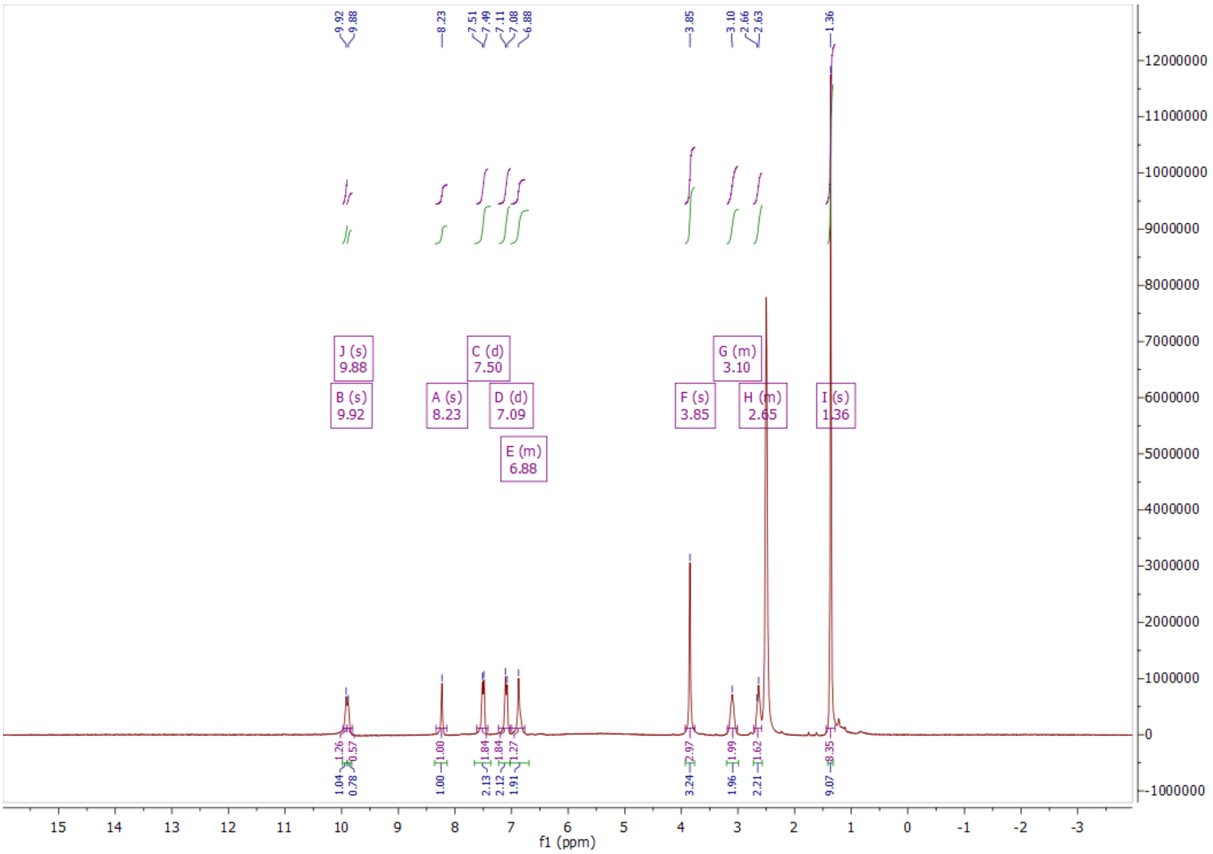

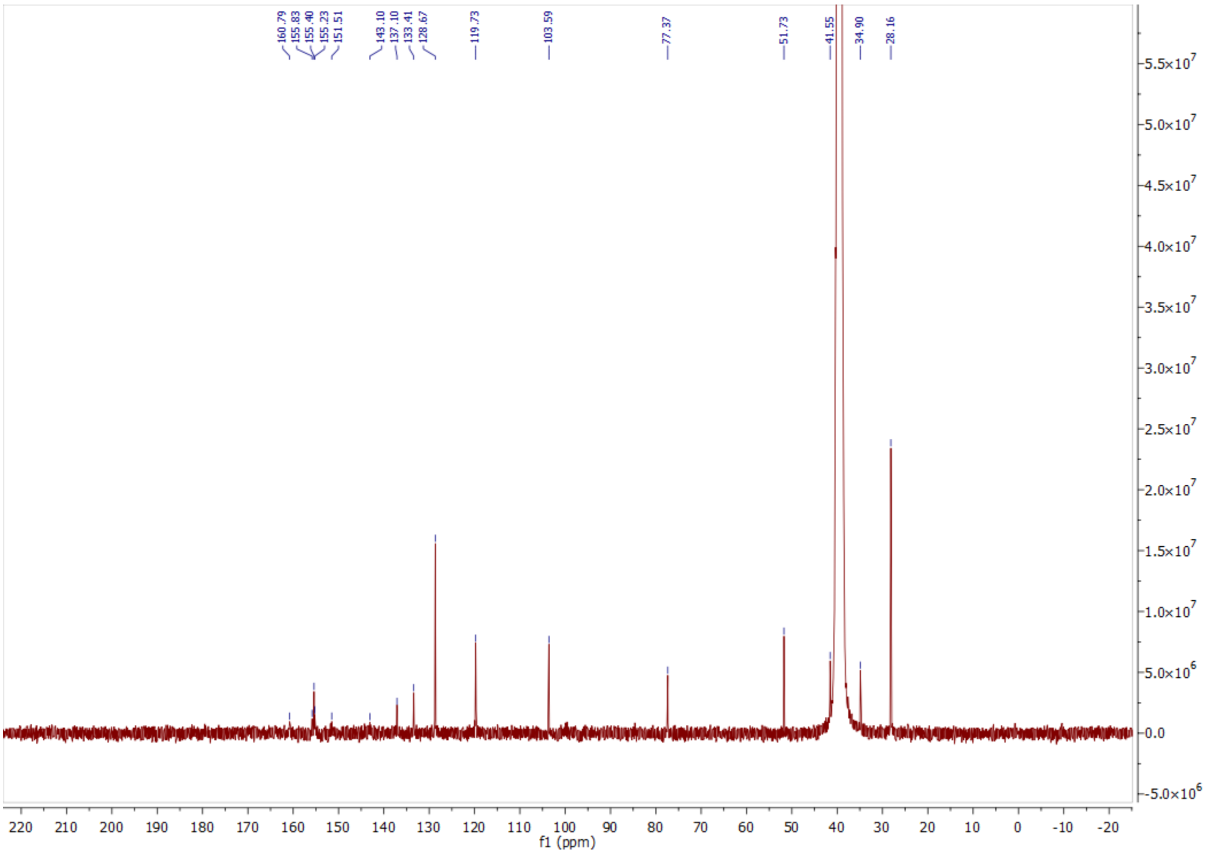

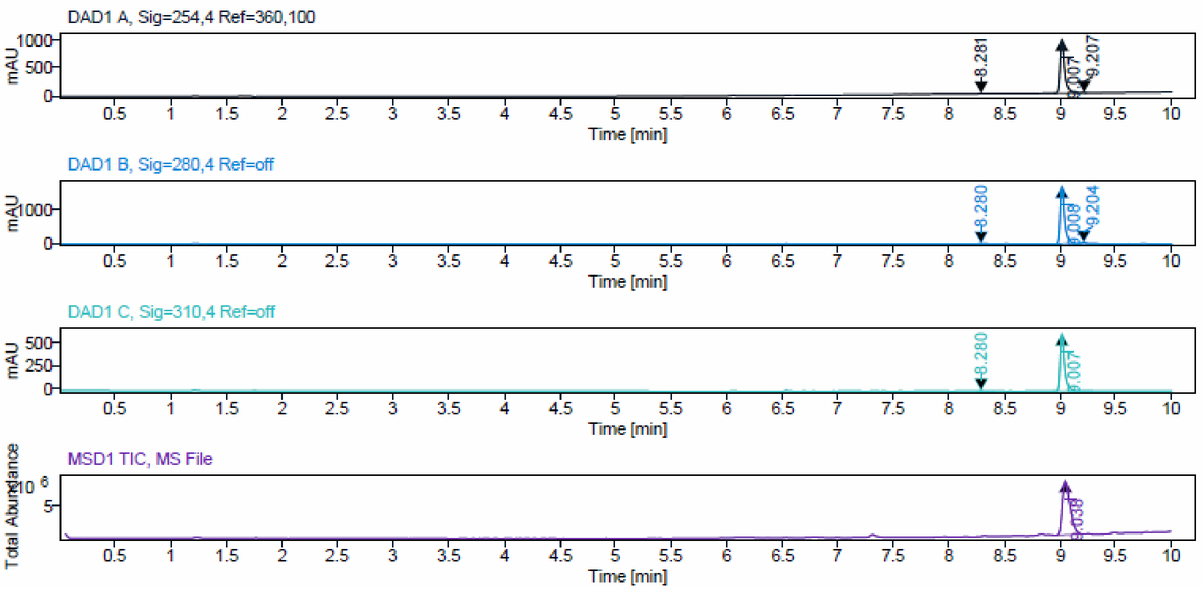

Sample Purity

Signal Description DAD1 A, Sig=254,4 Ref=360,100

Sample Name	Name	RT	Width	Area	Area%	Height
JA219_F17		8.281	0.046	26.9441	0.98	12.2955
JA219_F17		9.007	0.039	2707.9041	98.28	987.3286
JA219_F17		9.207	0.045	20.5357	0.75	8.6404

Max Area% 98.277

UV Signal Purity>95% Pass

Signal Description DAD1 B, Sig=280,4 Ref=off

Sample Name	Name	RT	Width	Area	Area%	Height
JA219_F17		8.280	0.036	43.1173	0.88	16.4284
JA219_F17		9.008	0.039	4741.7402	97.06	1734.8192
JA219_F17		9.204	0.041	100.4228	2.06	36.5106

Max Area% 97.062

UV Signal Purity>95% Pass

Signal Description DAD1 C, Sig=310,4 Ref=off

Sample Name	Name	RT	Width	Area	Area%	Height
JA219_F17		8.280	0.035	16.3519	0.96	6.4223
JA219_F17		9.007	0.038	1693.8707	99.04	617.0402

Max Area% 99.044

UV Signal Purity>95% Pass

^{1}H, ^{13}C NMR and HPLC data of compound **22b**.

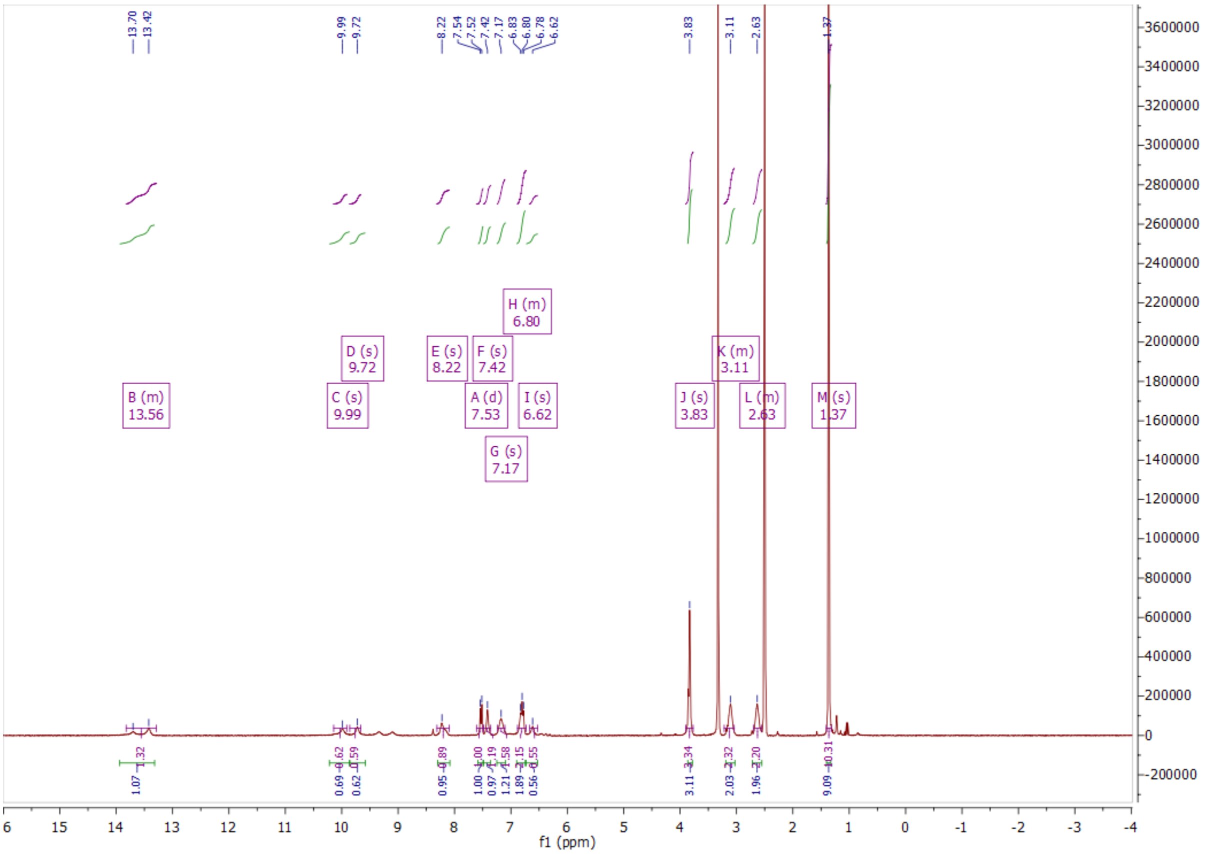

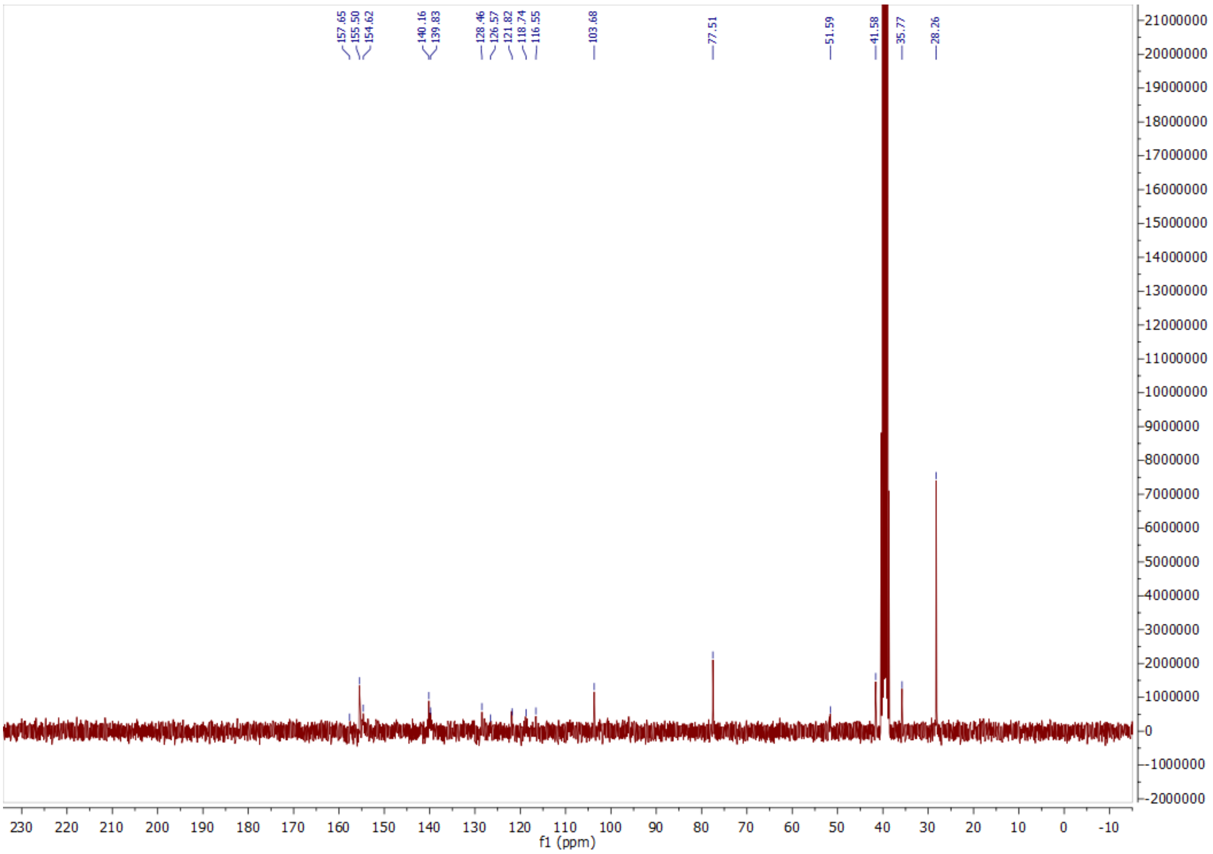

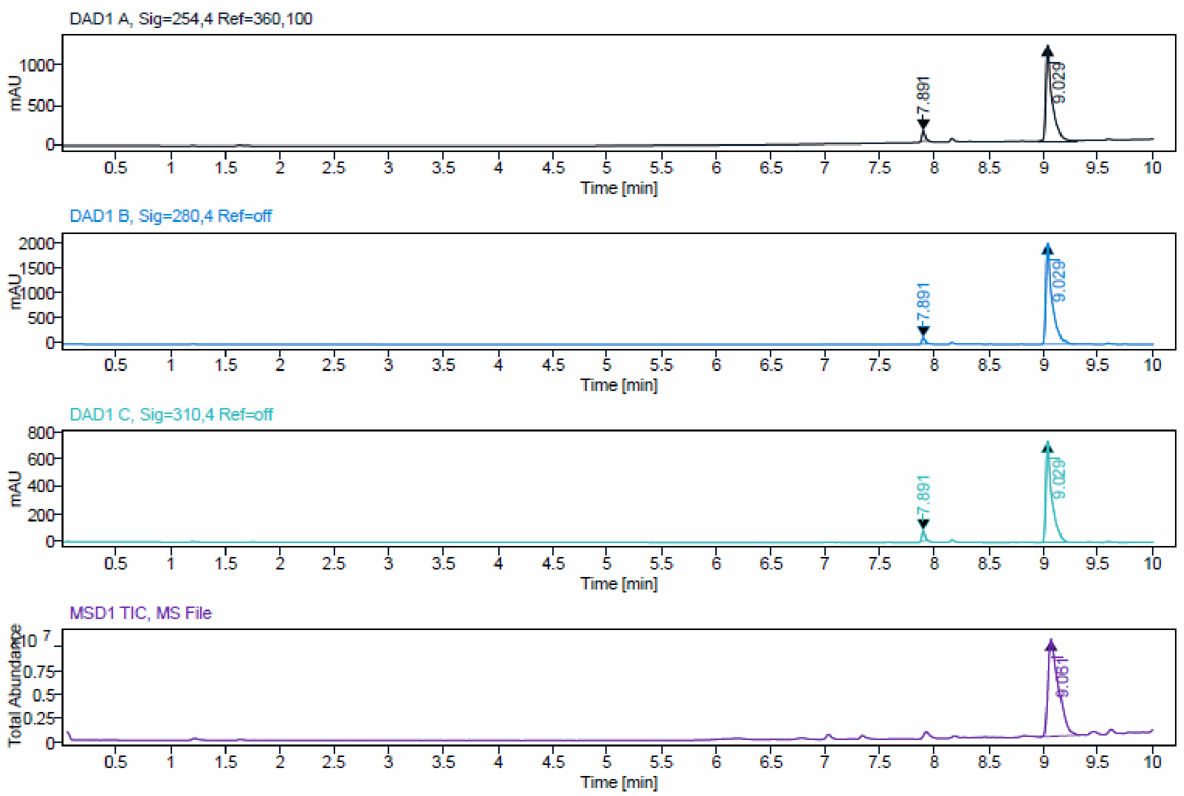

Sample Purity

Signal Description DAD1 A, Sig=254,4 Ref=360,100

Sample Name	Name	RT	Width	Area	Area%	Height
JA216_2_F5		7.891	0.032	257.2323	5.00	128.6893
JA216_2_F5		9.029	0.048	4891.3472	95.00	1214.5306

Max Area% 95.004

UV Signal Purity>95% Pass

Signal Description DAD1 B, Sig=280,4 Ref=off

Sample Name	Name	RT	Width	Area	Area%	Height
JA216_2_F5		7.891	0.033	258.4486	3.17	122.4907
JA216_2_F5		9.029	0.048	7897.1875	96.83	2018.6160

Max Area% 96.831

UV Signal Purity>95% Pass

Signal Description DAD1 C, Sig=310,4 Ref=off

Sample Name	Name	RT	Width	Area	Area%	Height
JA216_2_F5		7.891	0.031	152.4315	4.97	76.1894
JA216_2_F5		9.029	0.047	2912.3542	95.03	752.7323

Max Area% 95.026

UV Signal Purity>95% Pass

^{1}H, ^{13}C NMR and HPLC data of compound **22c**.

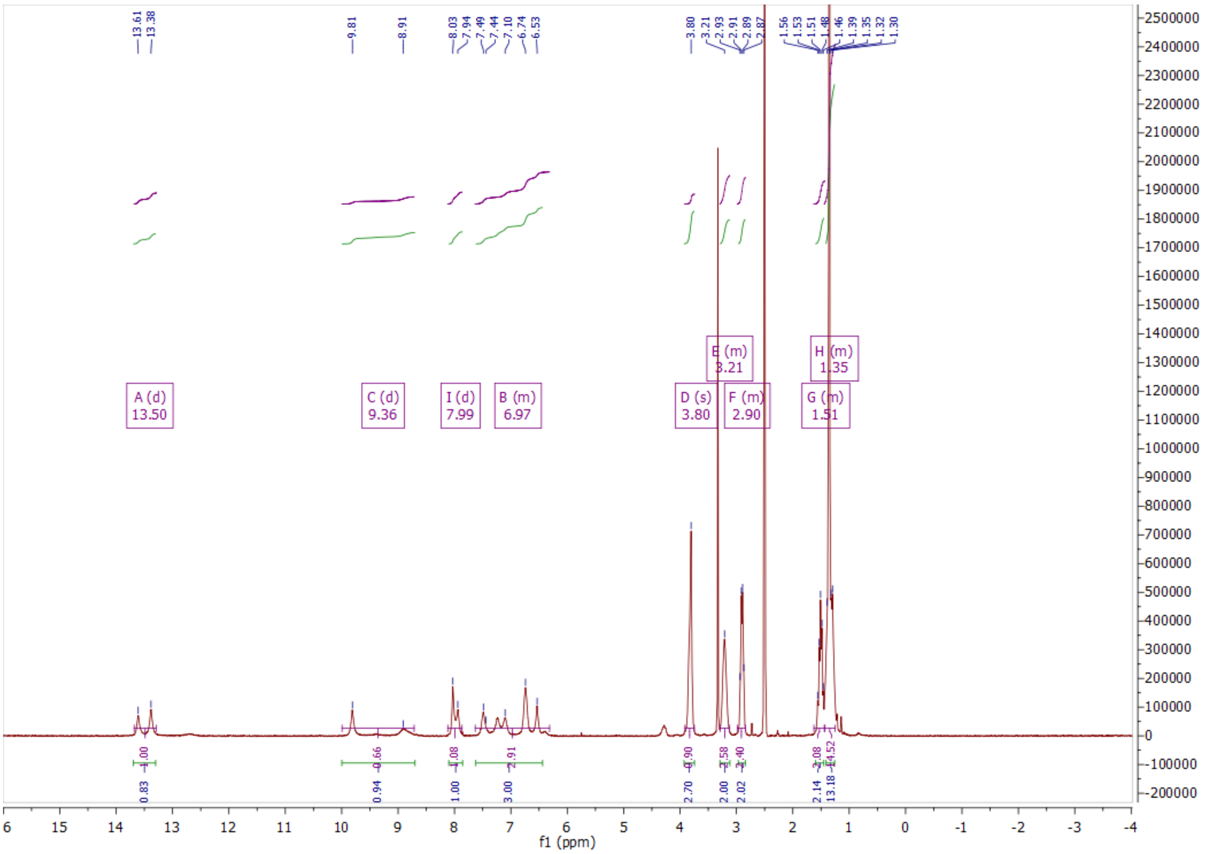

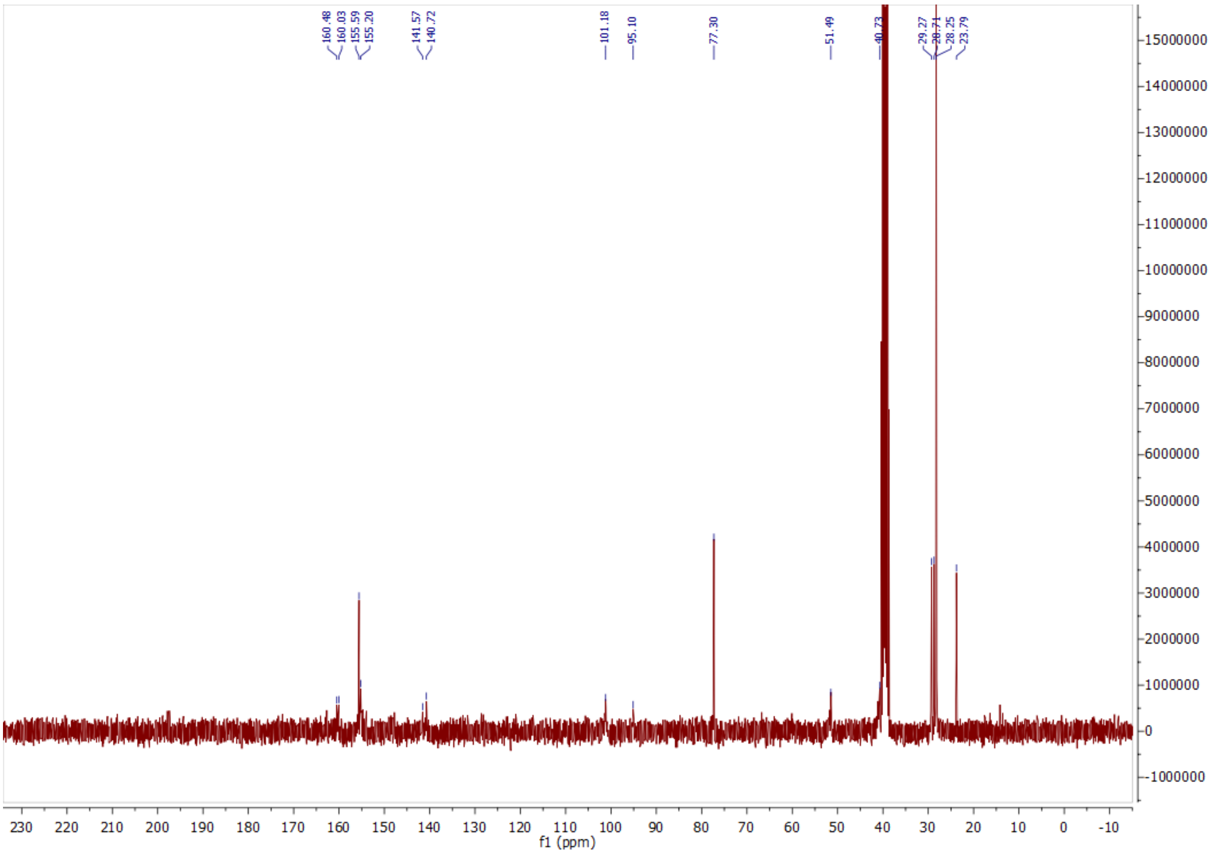

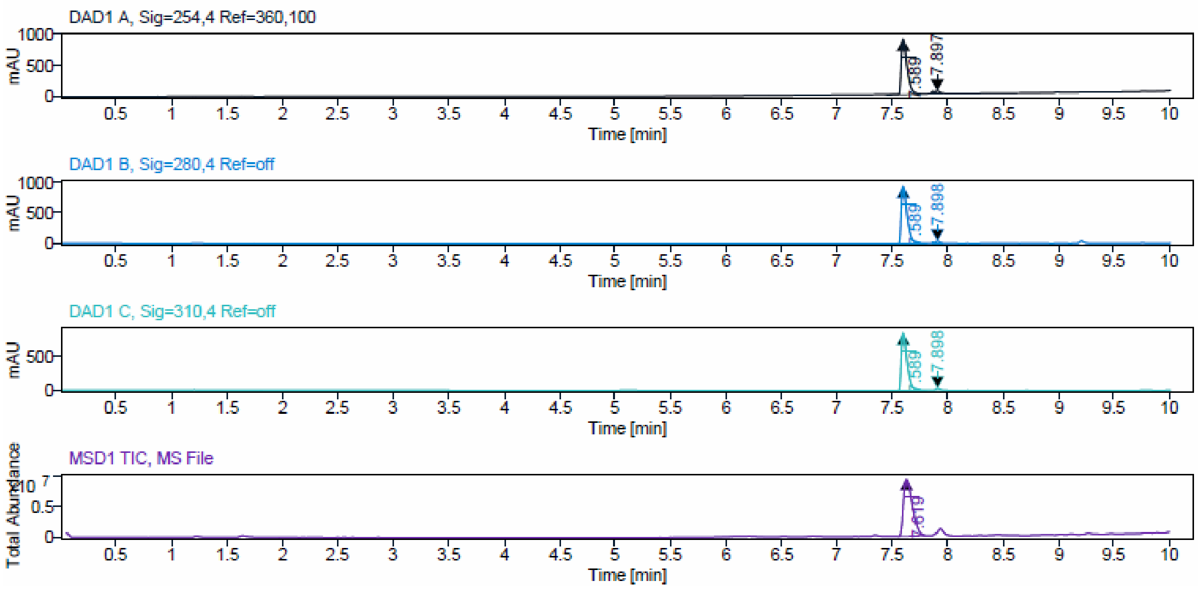

Sample Purity

Signal Description DAD1 A, Sig=254,4 Ref=360,100

Sample Name	Name	RT	Width	Area	Area%	Height
JA229_F12		7.589	0.054	3321.4458	95.04	905.7442
JA229_F12		7.897	0.071	173.3794	4.96	43.8780

Max Area% 95.039

UV Signal Purity>95% Pass

Signal Description DAD1 B, Sig=280,4 Ref=off

Sample Name	Name	RT	Width	Area	Area%	Height
JA229_F12		7.589	0.053	3382.2988	97.46	951.0269
JA229_F12		7.898	0.035	88.3266	2.54	35.7450

Max Area% 97.455

UV Signal Purity>95% Pass

Signal Description DAD1 C, Sig=310,4 Ref=off

Sample Name	Name	RT	Width	Area	Area%	Height
JA229_F12		7.589	0.053	3105.7893	98.19	871.3784
JA229_F12		7.898	0.032	57.3088	1.81	30.3489

Max Area% 98.188

UV Signal Purity>95% Pass

^{1}H, ^{13}C NMR and HPLC data of compound **22d**.

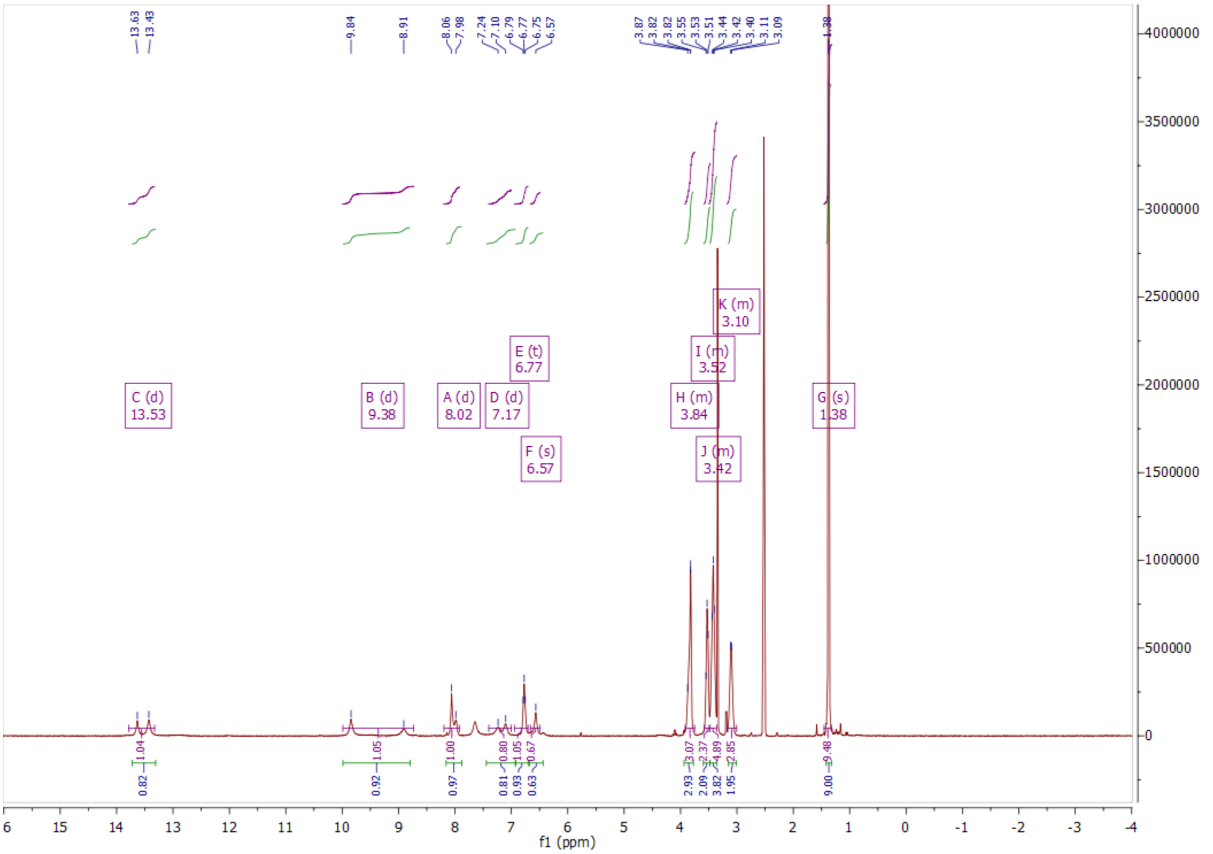

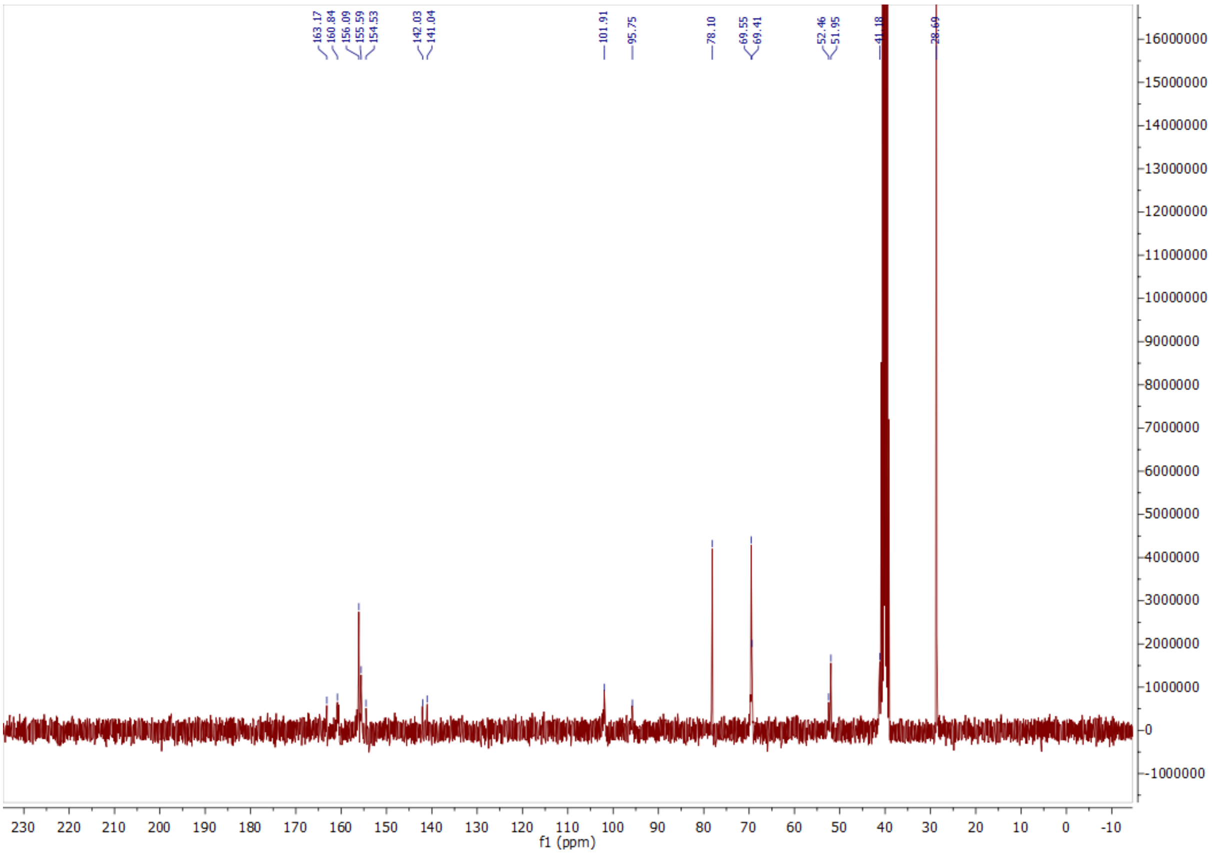

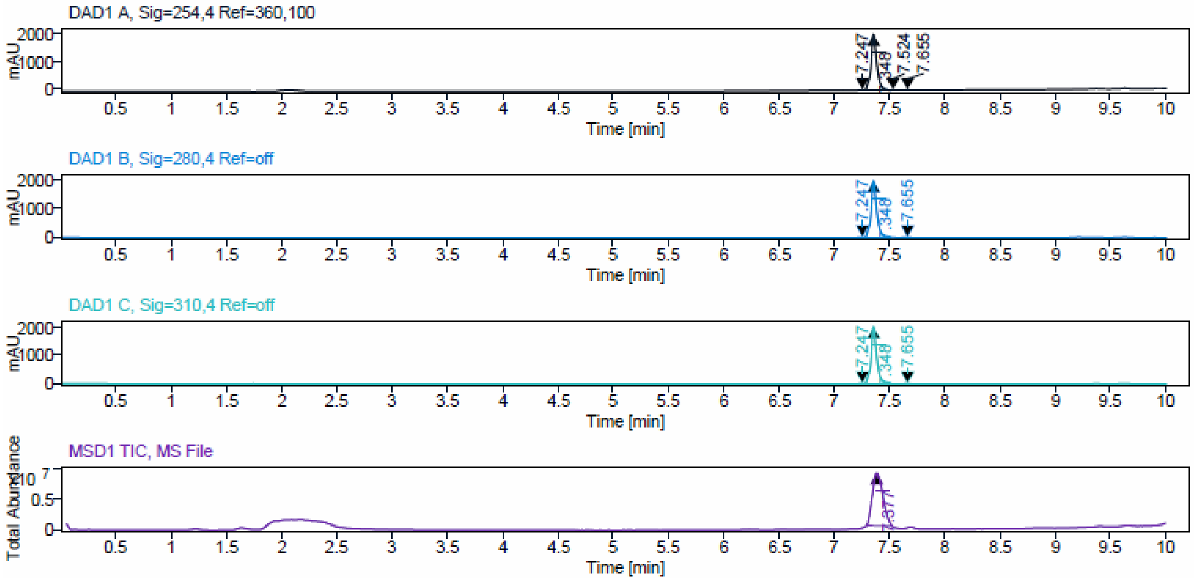

Sample Purity

Signal Description DAD1 A, Sig=254,4 Ref=360,100

Sample Name	Name	RT	Width	Area	Area%	Height
JA244_product		7.247	0.018	6.6651	0.10	6.2215
JA244_product		7.348	0.049	6960.0249	99.43	1960.5637
JA244_product		7.524	0.033	15.4476	0.22	7.8229
JA244_product		7.655	0.031	17.9934	0.26	9.7974

Max Area% 99.427

UV Signal Purity>95% Pass

Signal Description DAD1 B, Sig=280,4 Ref=off

Sample Name	Name	RT	Width	Area	Area%	Height
JA244_product		7.247	0.041	55.2211	0.75	18.9209
JA244_product		7.348	0.047	7226.4063	98.36	2043.6461
JA244_product		7.655	0.045	65.2849	0.89	14.6890

Max Area% 98.360

UV Signal Purity>95% Pass

Signal Description DAD1 C, Sig=310,4 Ref=off

Sample Name	Name	RT	Width	Area	Area%	Height
JA244_product		7.247	0.040	47.7897	0.64	17.4729
JA244_product		7.348	0.047	7309.6895	98.63	2069.8733
JA244_product		7.655	0.042	53.9669	0.73	14.0873

Max Area% 98.627

UV Signal Purity>95% Pass

^{1}H, ^{13}C NMR and HPLC data of compound **22e**.

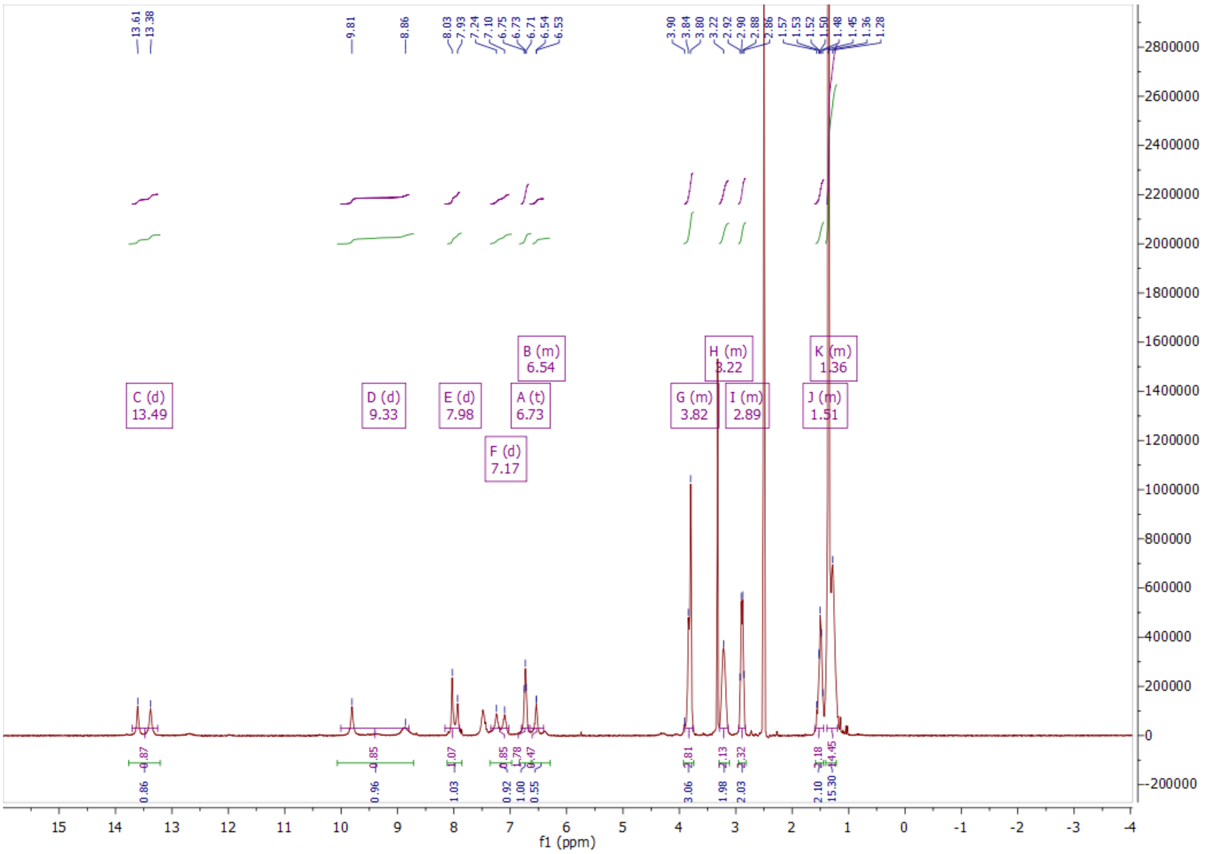

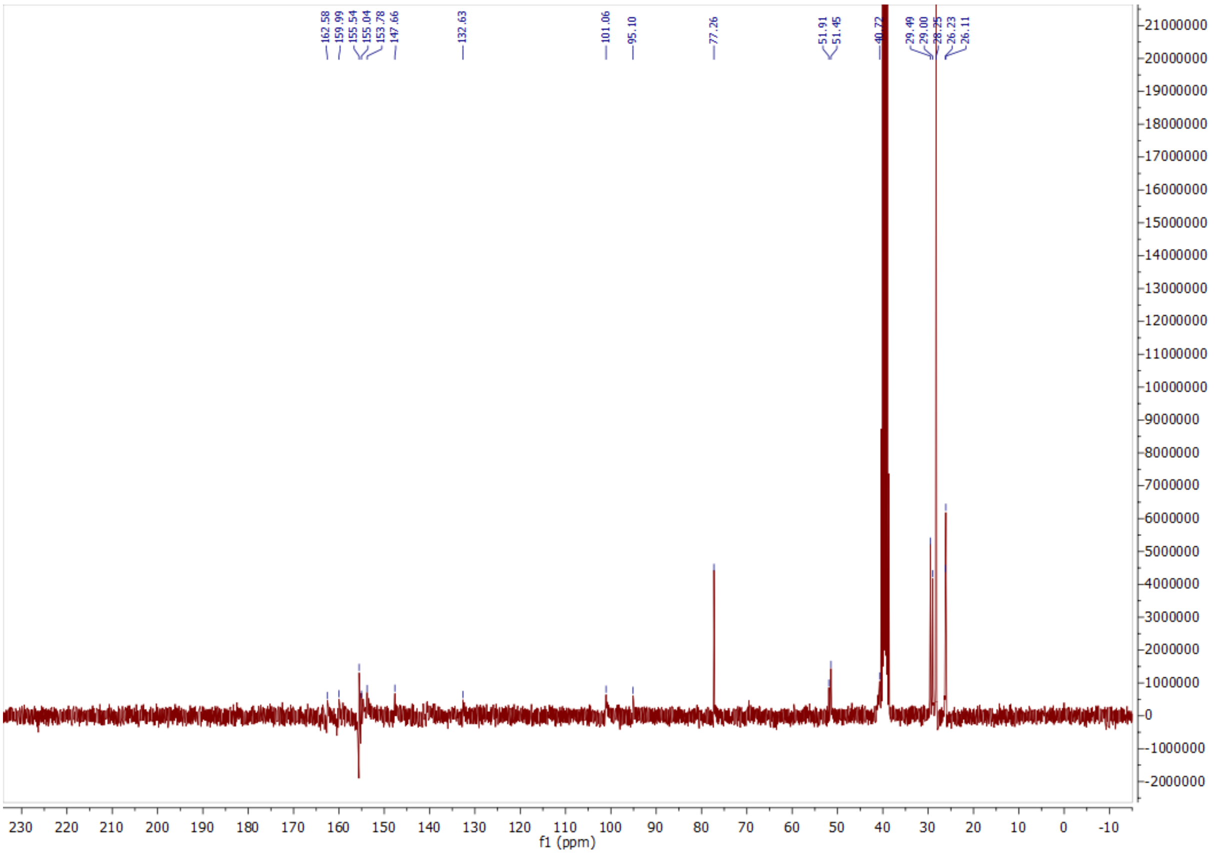

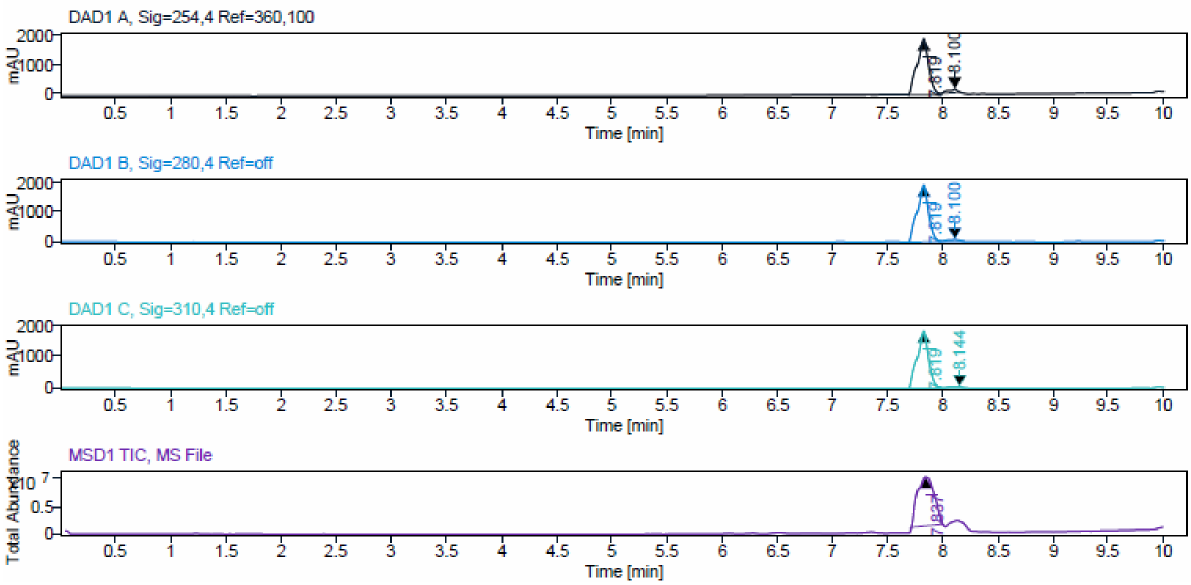

Sample Purity

Signal Description DAD1 A, Sig=254,4 Ref=360,100

Sample Name	Name	RT	Width	Area	Area%	Height
JA242_F10		7.819	0.115	13908.89 06	95.12	1891.8716
JA242_F10		8.100	0.102	713.2922	4.88	125.9100

Max Area% 95.122

UV Signal Purity>95% Pass

Signal Description DAD1 B, Sig=280,4 Ref=off

Sample Name	Name	RT	Width	Area	Area%	Height
JA242_F10		7.819	0.110	14103.97 56	96.49	1974.7263
JA242_F10		8.100	0.139	513.7810	3.51	70.9866

Max Area% 96.485

UV Signal Purity>95% Pass

Signal Description DAD1 C, Sig=310,4 Ref=off

Sample Name	Name	RT	Width	Area	Area%	Height
JA242_F10		7.819	0.110	13242.50 20	98.30	1838.7095
JA242_F10		8.144	0.137	228.7905	1.70	33.6280

Max Area% 98.302

UV Signal Purity>95% Pass

^{1}H, ^{13}C NMR and HPLC data of compound **23a**.

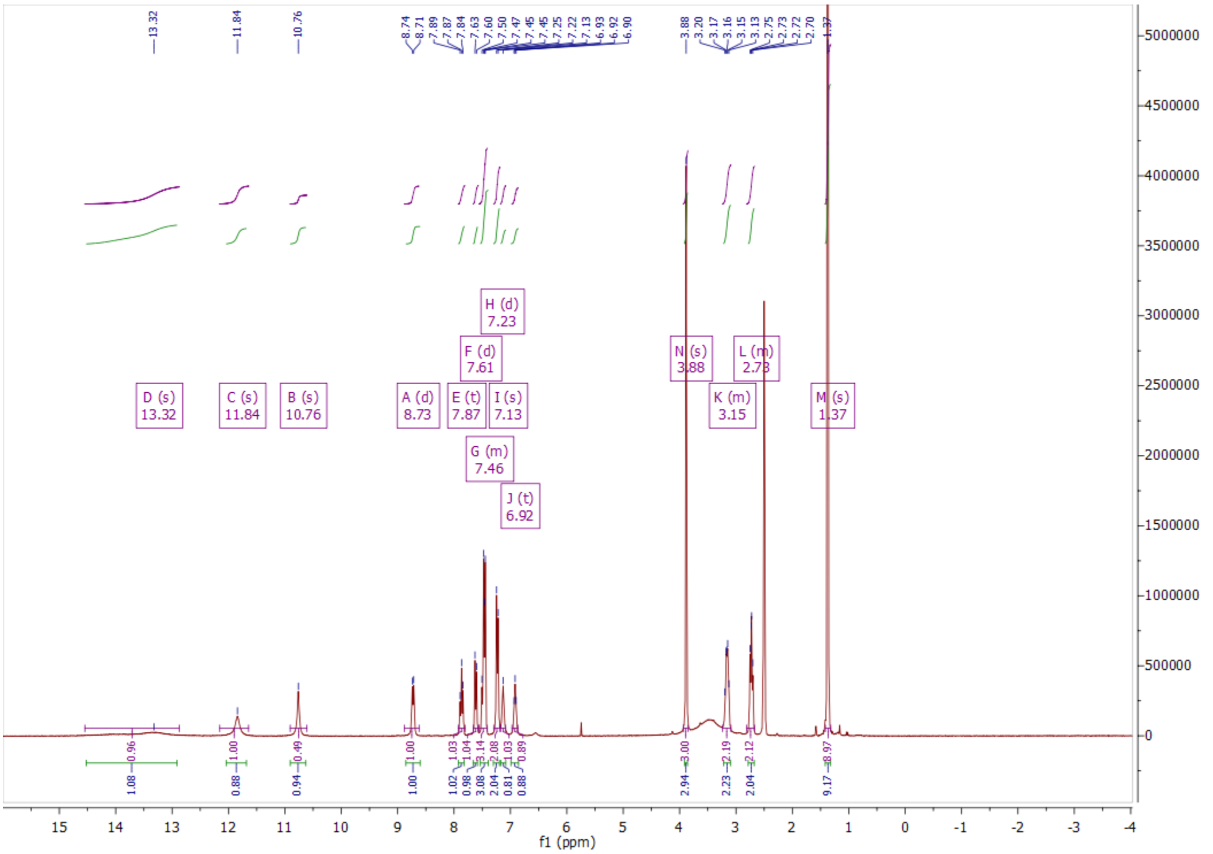

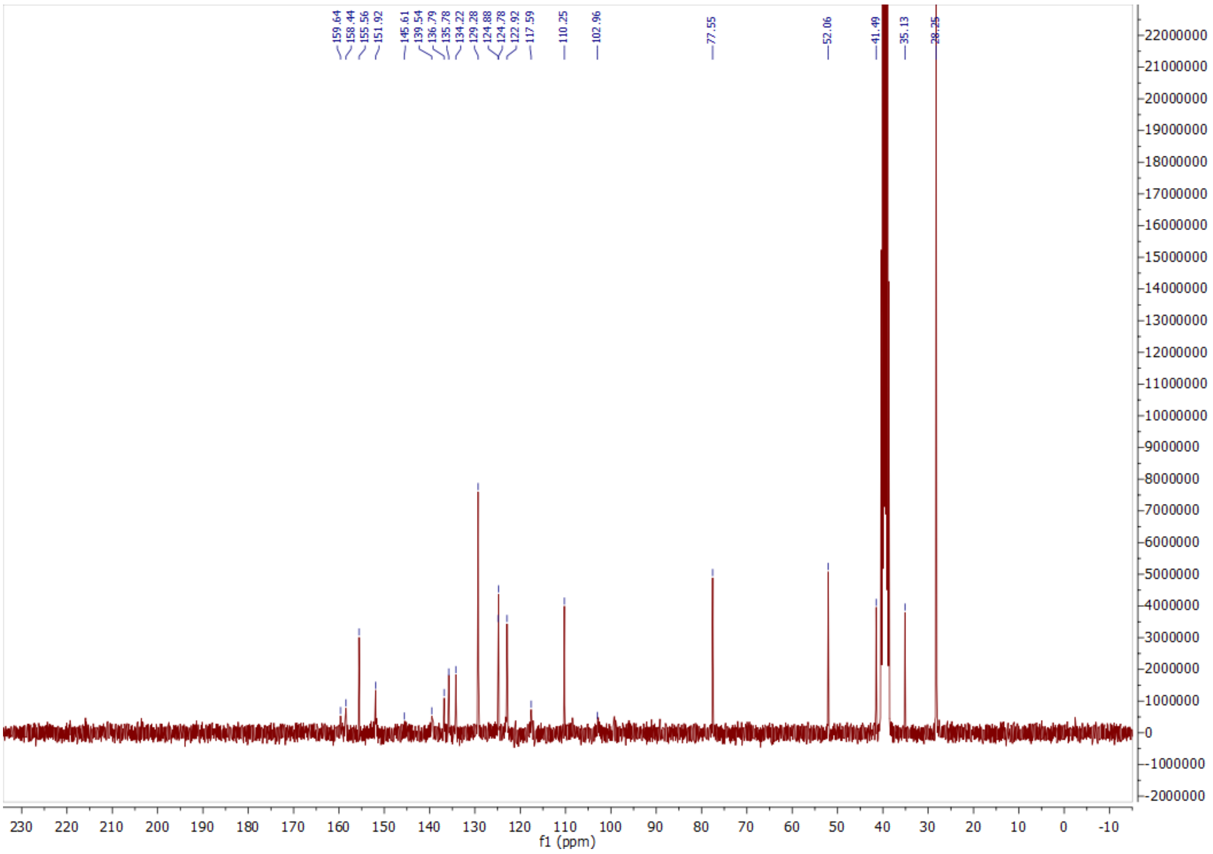

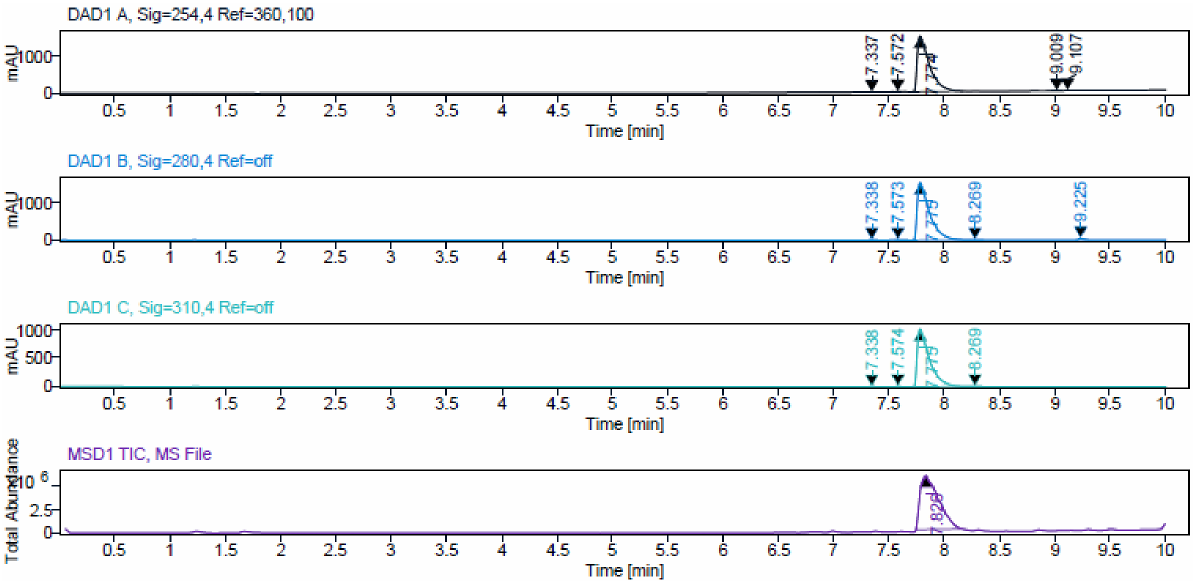

Sample Purity

Signal Description DAD1 A, Sig=254,4 Ref=360,100

Sample Name	Name	RT	Width	Area	Area%	Height
JA282_F15		7.337	0.054	18.5845	0.16	6.4313
JA282_F15		7.572	0.053	43.8318	0.38	12.9010
JA282_F15		7.774	0.113	11492.2168	98.66	1489.1780
JA282_F15		9.009	0.069	46.4684	0.40	12.3748
JA282_F15		9.107	0.074	47.3371	0.41	13.1516

Max Area% 98.659

UV Signal Purity>95% Pass

Signal Description DAD1 B, Sig=280,4 Ref=off

Sample Name	Name	RT	Width	Area	Area%	Height
JA282_F15		7.338	0.045	23.2447	0.20	6.7086
JA282_F15		7.573	0.037	22.7132	0.19	8.0980
JA282_F15		7.775	0.111	11714.4355	98.69	1518.0446
JA282_F15		8.269	0.046	18.0723	0.15	5.9514
JA282_F15		9.225	0.047	91.2506	0.77	32.6501

Max Area% 98.692

UV Signal Purity>95% Pass

Signal Description DAD1 C, Sig=310,4 Ref=off

Sample Name	Name	RT	Width	Area	Area%	Height
JA282_F15		7.338	0.045	16.0702	0.20	4.6109
JA282_F15		7.574	0.039	17.9199	0.22	5.7650
JA282_F15		7.775	0.111	8134.1978	99.42	1057.8473
JA282_F15		8.269	0.047	13.2664	0.16	4.1943

Max Area% 99.422

UV Signal Purity>95% Pass

^{1}H, ^{13}C NMR and HPLC data of compound **23b**.

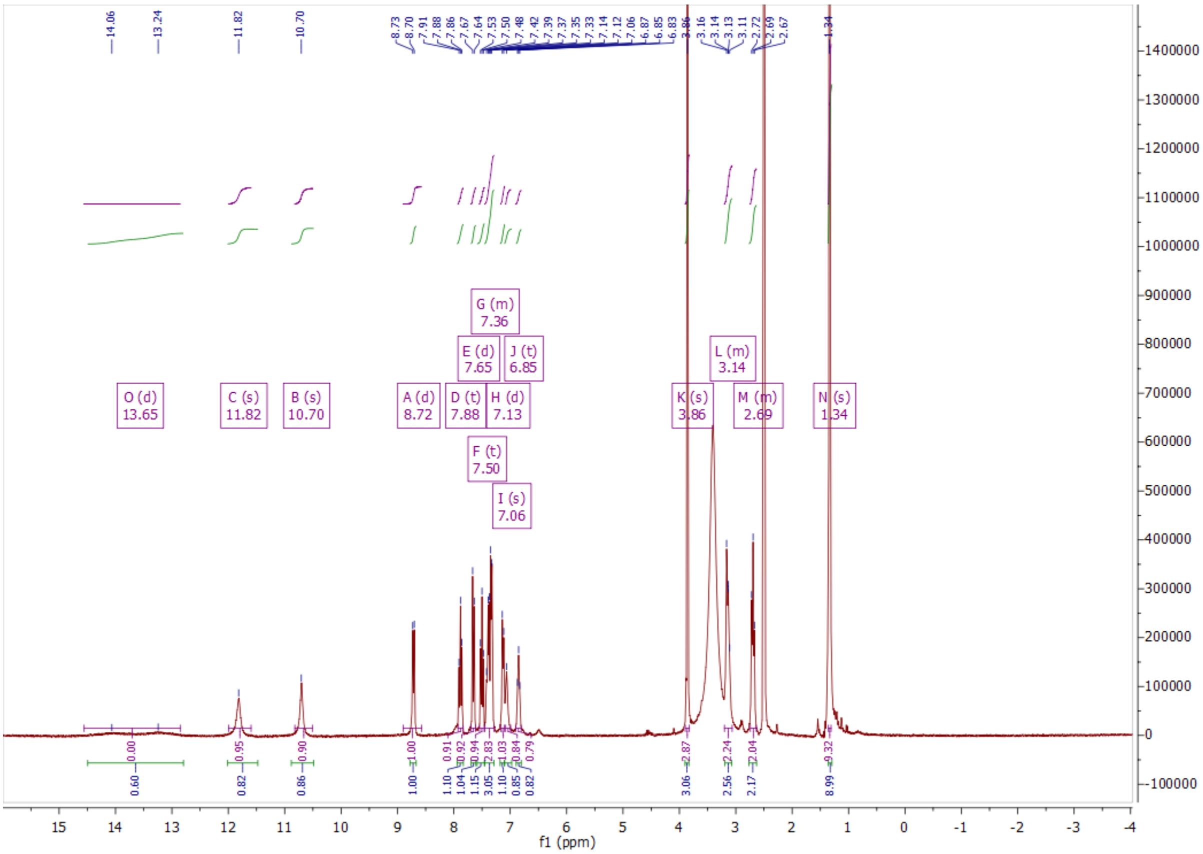

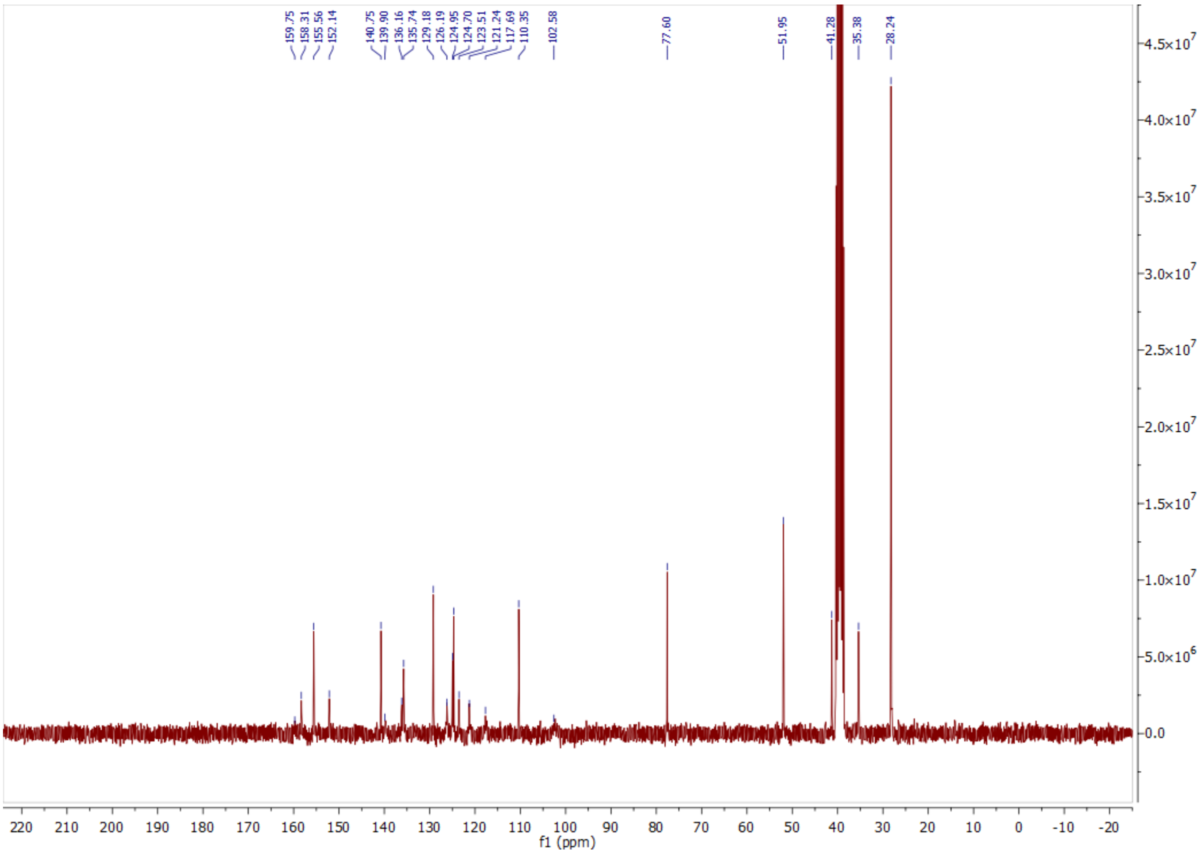

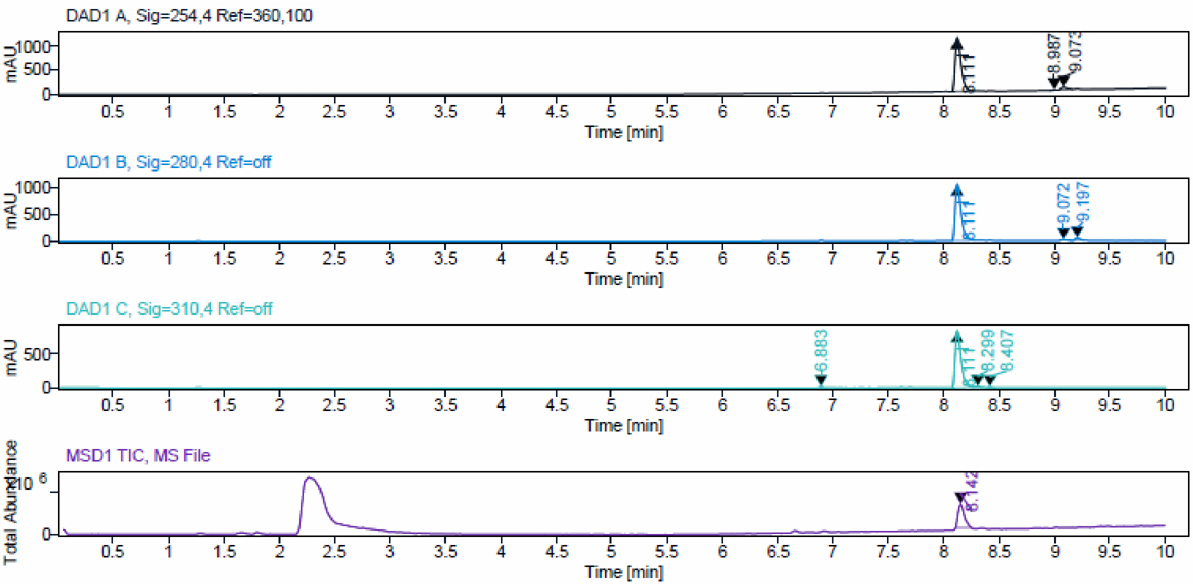

Sample Purity

Signal Description DAD1 A, Sig=254,4 Ref=360,100

Sample Name	Name	RT	Width	Area	Area%	Height
JA285_F7		8.111	0.057	4212.3657	95.26	1127.8860
JA285_F7		8.987	0.046	22.7012	0.51	8.2231
JA285_F7		9.073	0.065	186.7537	4.22	48.2658

Max Area% 95.263

UV Signal Purity>95% Pass

Signal Description DAD1 B, Sig=280,4 Ref=off

Sample Name	Name	RT	Width	Area	Area%	Height
JA285_F7		8.111	0.057	3988.7537	95.70	1060.9926
JA285_F7		9.072	0.064	54.3462	1.30	14.3521
JA285_F7		9.197	0.050	124.8755	3.00	41.3984

Max Area% 95.700

UV Signal Purity>95% Pass

Signal Description DAD1 C, Sig=310,4 Ref=off

Sample Name	Name	RT	Width	Area	Area%	Height
JA285_F7		6.883	0.033	28.6834	0.89	13.9921
JA285_F7		8.111	0.057	3146.4600	97.92	836.7382
JA285_F7		8.299	0.046	17.4013	0.54	5.8589
JA285_F7		8.407	0.044	20.7846	0.65	7.1484

Max Area% 97.919

UV Signal Purity>95% Pass

^{1}H, ^{13}C NMR and HPLC data of compound **23c**.

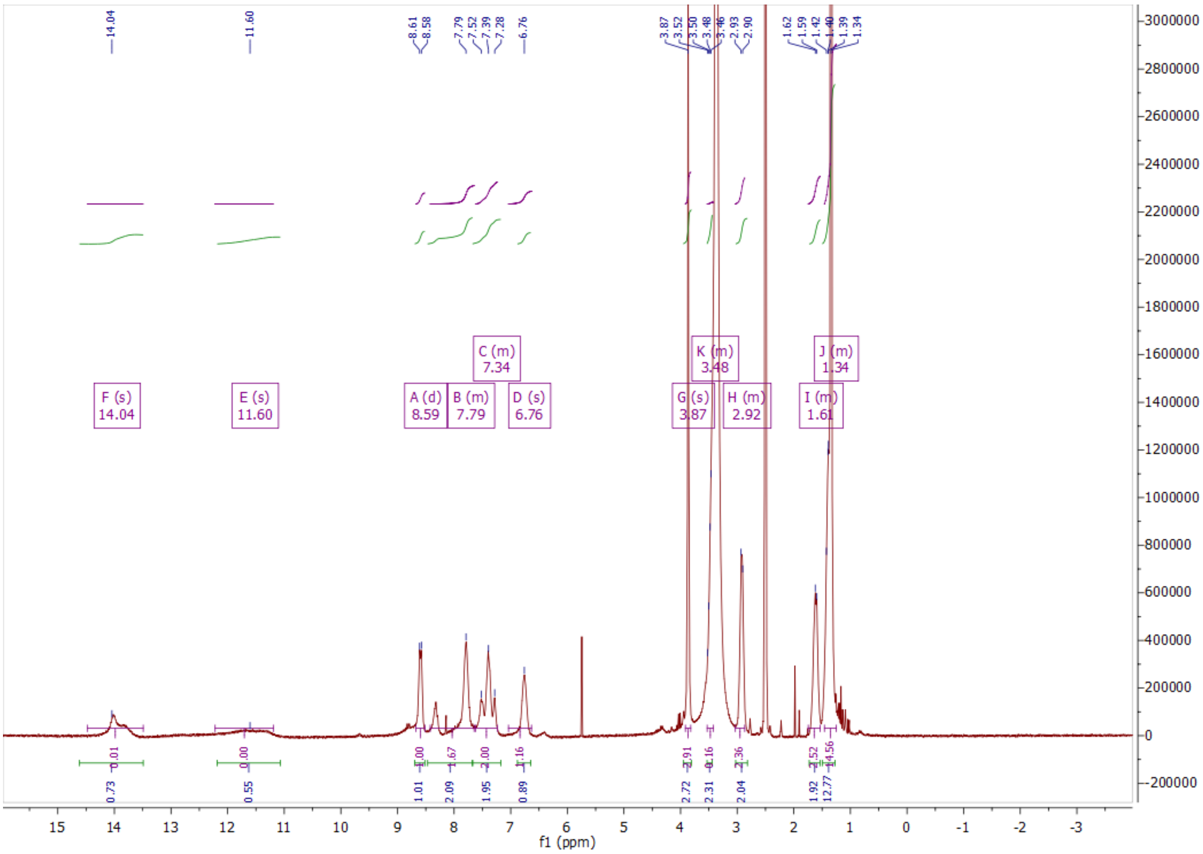

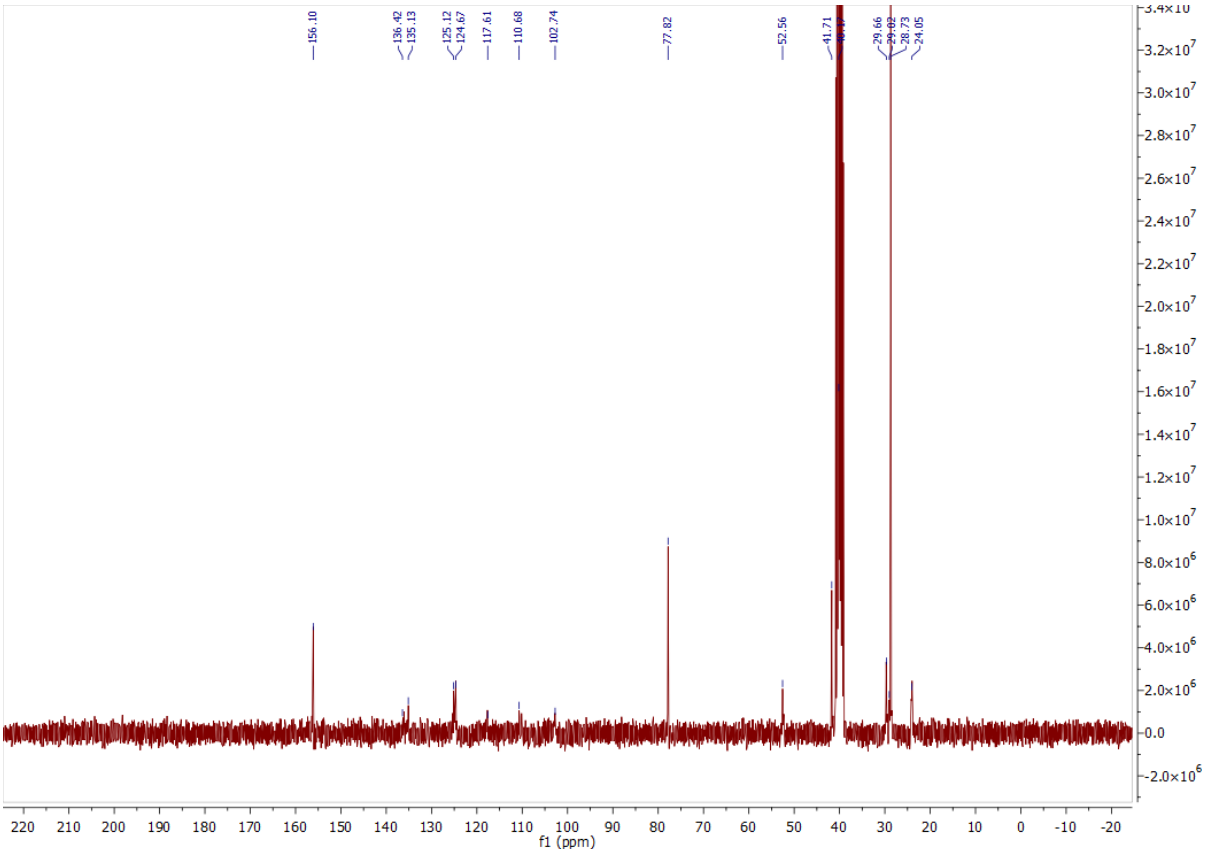

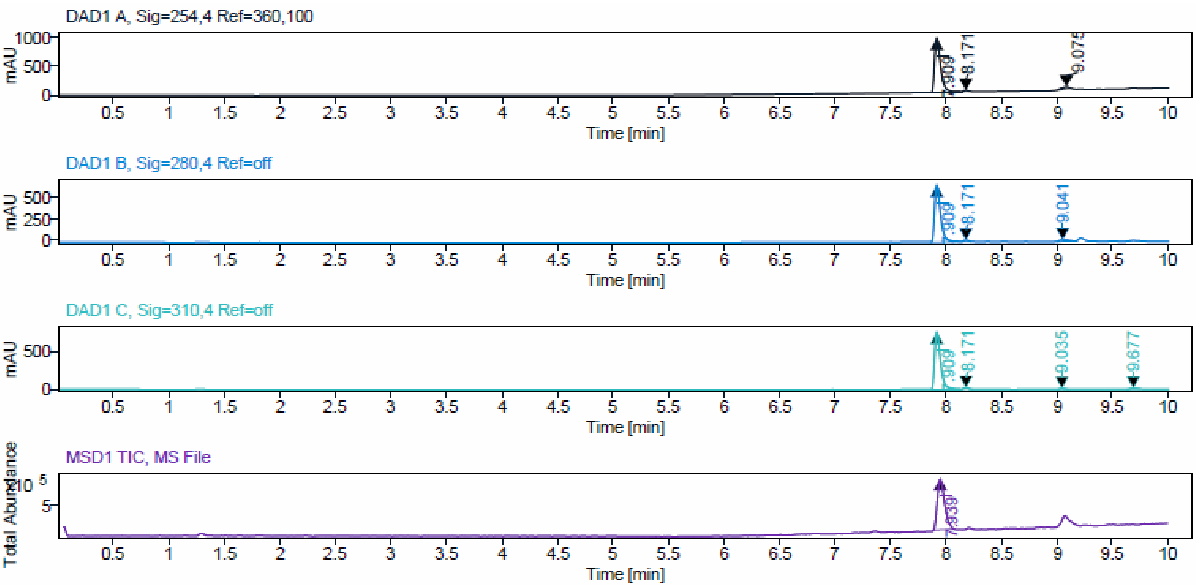

Sample Purity

Signal Description DAD1 A, Sig=254,4 Ref=360,100

Sample Name	Name	RT	Width	Area	Area%	Height
JA286_2_F6		7.909	0.056	3739.5625	95.11	977.5157
JA286_2_F6		8.171	0.035	36.5721	0.93	17.3514
JA286_2_F6		9.075	0.069	155.7726	3.96	34.1628

Max Area% 95.108

UV Signal Purity>95% Pass

Signal Description DAD1 B, Sig=280,4 Ref=off

Sample Name	Name	RT	Width	Area	Area%	Height
JA286_2_F6		7.909	0.054	2451.2275	95.22	661.6134
JA286_2_F6		8.171	0.042	38.3186	1.49	15.1009
JA286_2_F6		9.041	0.092	84.6018	3.29	17.6666

Max Area% 95.225

UV Signal Purity>95% Pass

Signal Description DAD1 C, Sig=310,4 Ref=off

Sample Name	Name	RT	Width	Area	Area%	Height
JA286_2_F6		7.909	0.054	2791.7505	95.86	764.8190
JA286_2_F6		8.171	0.041	43.9157	1.51	17.2489
JA286_2_F6		9.035	0.053	34.8448	1.20	11.1154
JA286_2_F6		9.677	0.067	41.8919	1.44	11.1434

Max Area% 95.857

UV Signal Purity>95% Pass

^{1}H, ^{13}C NMR and HPLC data of compound **24**.

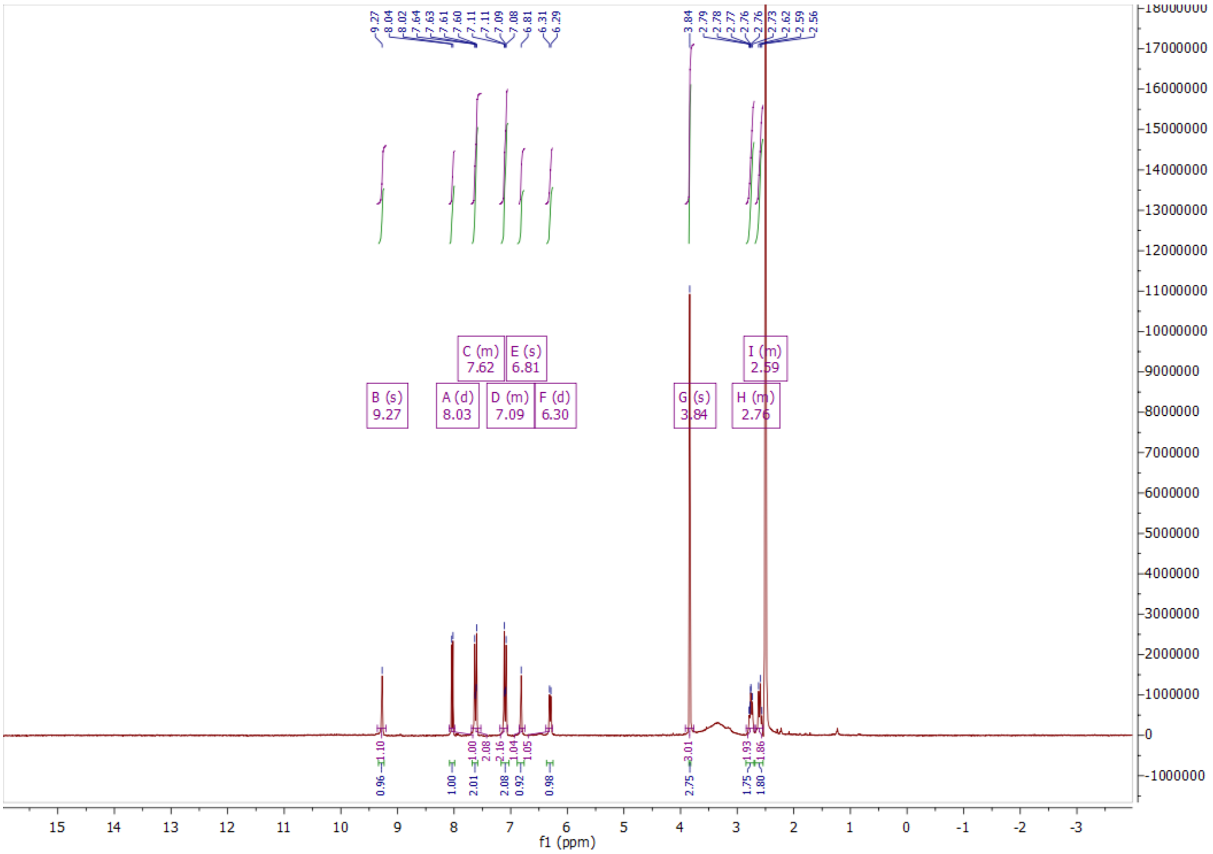

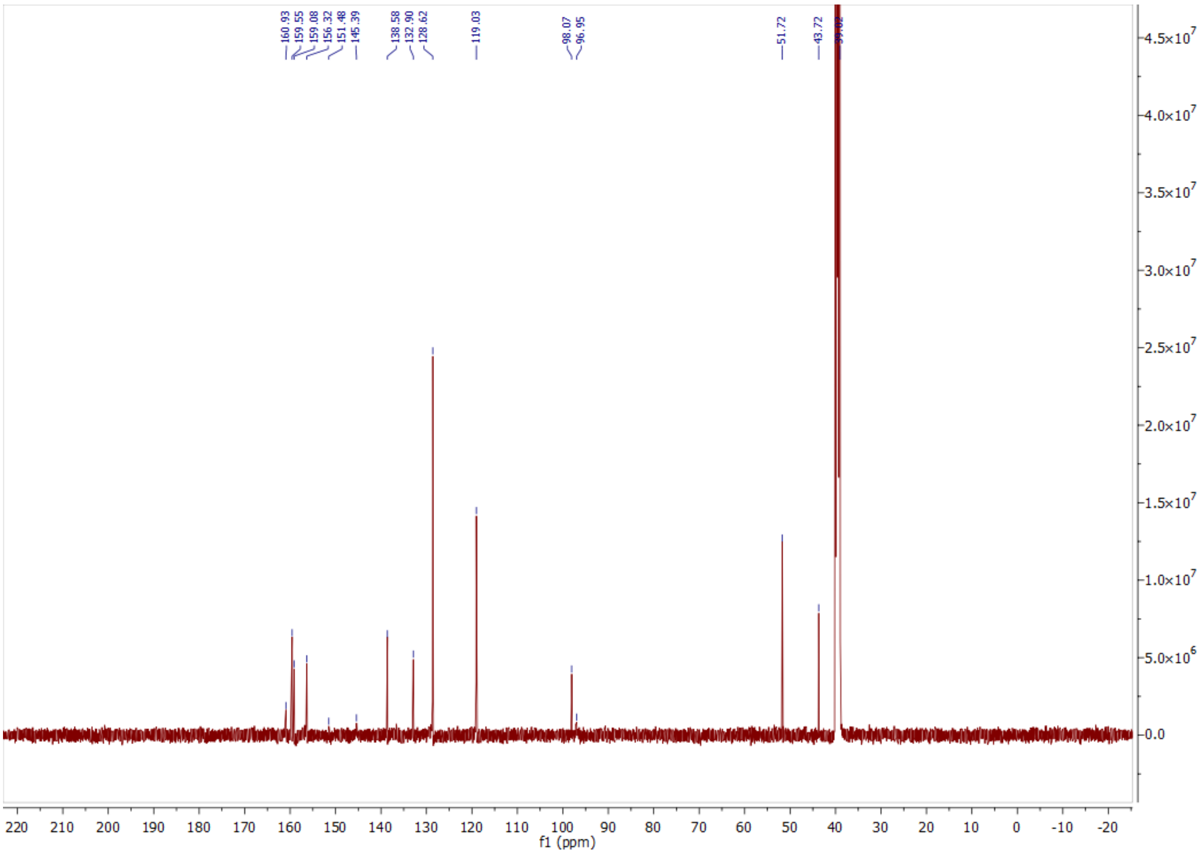

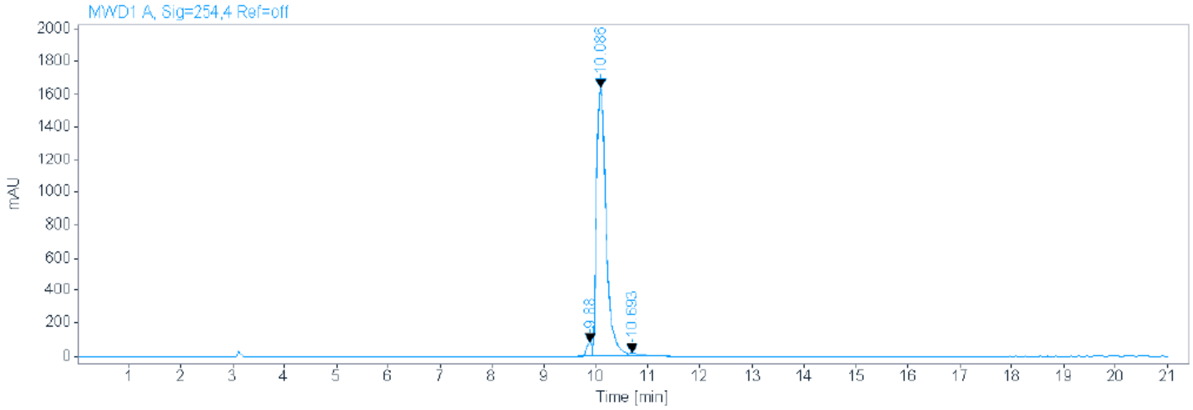

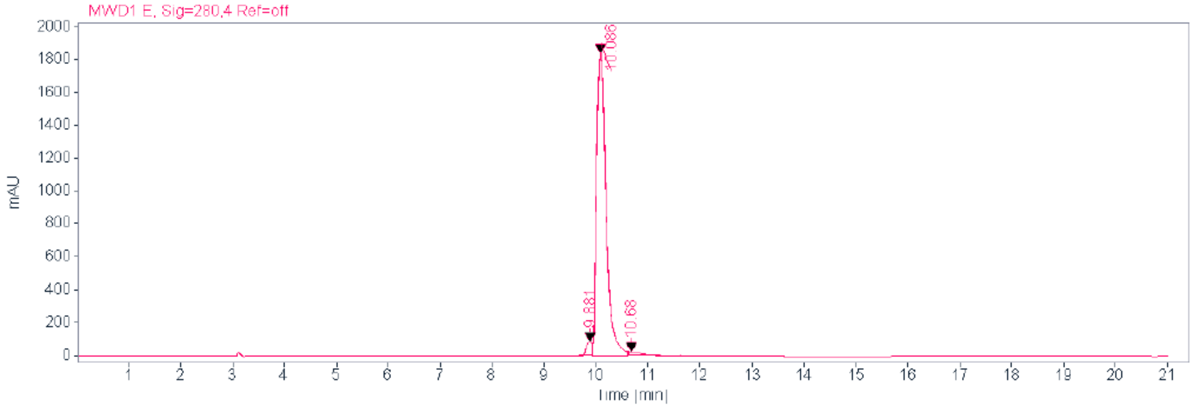

Signal: MSD1 TIC, MS File

RT [min]	Type	Width [min]	Area	Height	Area%	Name
10.284	BB	0.3672	127534464.0000	5424947.5000	100.0000	
		Sum	127534464.0000			

Signal: MSD2 TIC, MS File

RT [min]	Type	Width [min]	Area	Height	Area%	Name
10.249	MM	0.3418	4109865.2500	200428.5781	100.0000	
		Sum	4109865.2500			

Signal: MWD1 A, Sig=254,4 Ref=off

RT [min]	Type	Width [min]	Area	Height	Area%	Name
9.880	MM	0.1284	597.8953	77.6385	2.7330	
10.086	MM	0.2122	20872.6133	1639.6437	95.4110	
10.693	MM	0.3305	406.0184	20.4763	1.8560	
		Sum	21876.5270			

Signal: MWD1 E, Sig=280,4 Ref=off

RT [min]	Type	Width [min]	Area	Height	Area%	Name
9.881	BV	0.1347	667.7483	84.9856	2.7196	
10.086	MM	0.2119	23396.1289	1840.2412	95.2884	
10.680	MM	0.3344	489.0952	24.3793	1.9920	
		Sum	24552.9724			

[1]H and HPLC data of compound **25**.

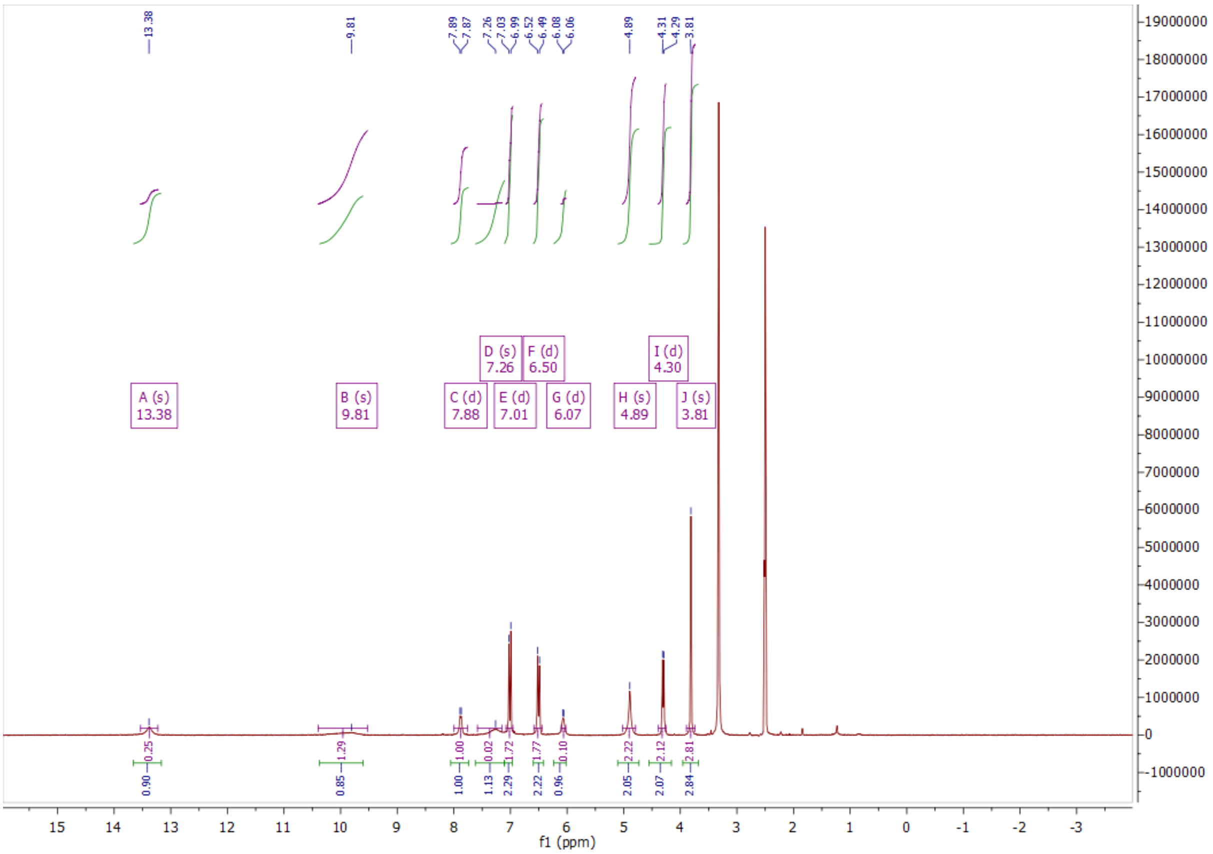

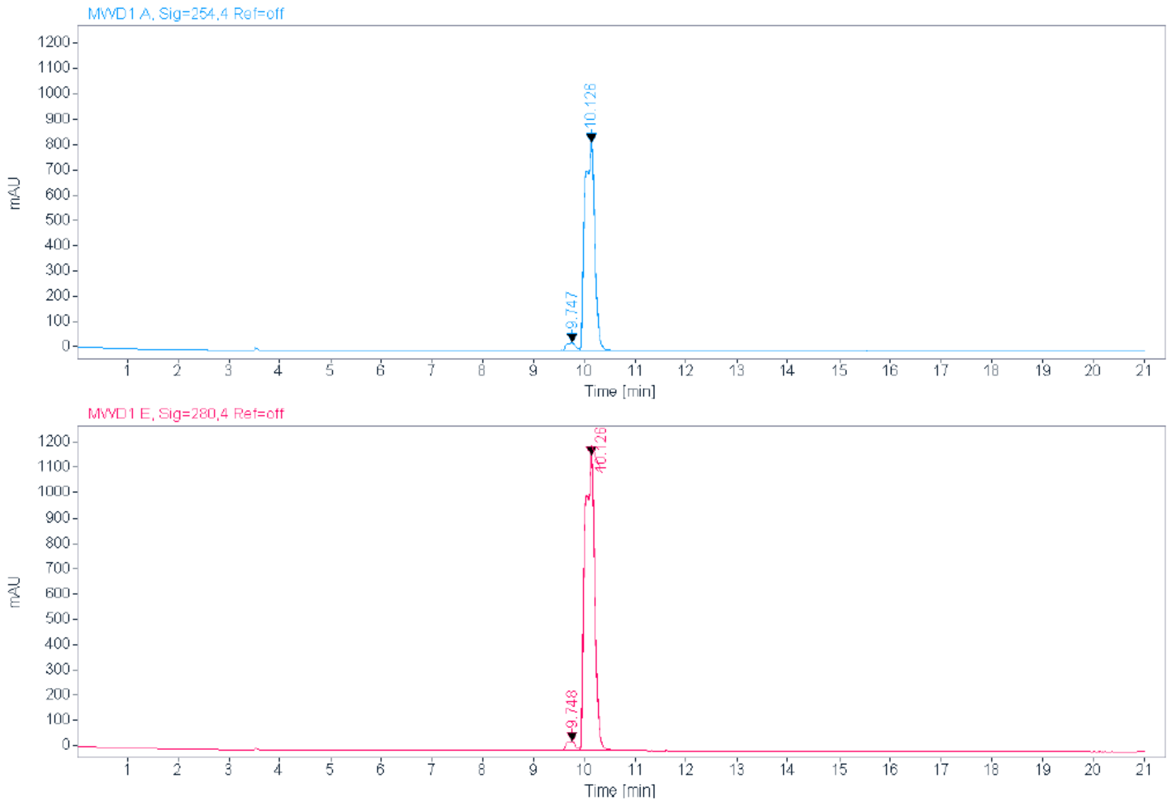

Signal: MSD1 TIC, MS File

RT [min]	Type	Width [min]	Area	Height	Area%	Name
10.279	MM	0.3313	69807984.0000	3512338.7500	100.0000	
		Sum	69807984.0000			

Signal: MSD2 TIC, MS File

RT [min]	Type	Width [min]	Area	Height	Area%	Name
10.279	MM	0.3334	3910586.0000	195473.2500	100.0000	
		Sum	3910586.0000			

Signal: MWD1 A, Sig=254,4 Ref=off

RT [min]	Type	Width [min]	Area	Height	Area%	Name
9.747	MF	0.2056	378.7932	30.7116	3.3358	
10.126	FM	0.2229	10976.5176	820.6157	96.6642	
		Sum	11355.3107			

Signal: MWD1 E, Sig=280,4 Ref=off

RT [min]	Type	Width [min]	Area	Height	Area%	Name
9.748	MF	0.2034	434.8949	35.6284	2.7139	
10.126	FM	0.2230	15589.8066	1165.2881	97.2861	
		Sum	16024.7015			

^{1}H and ^{13}C NMR data of compound **32**.

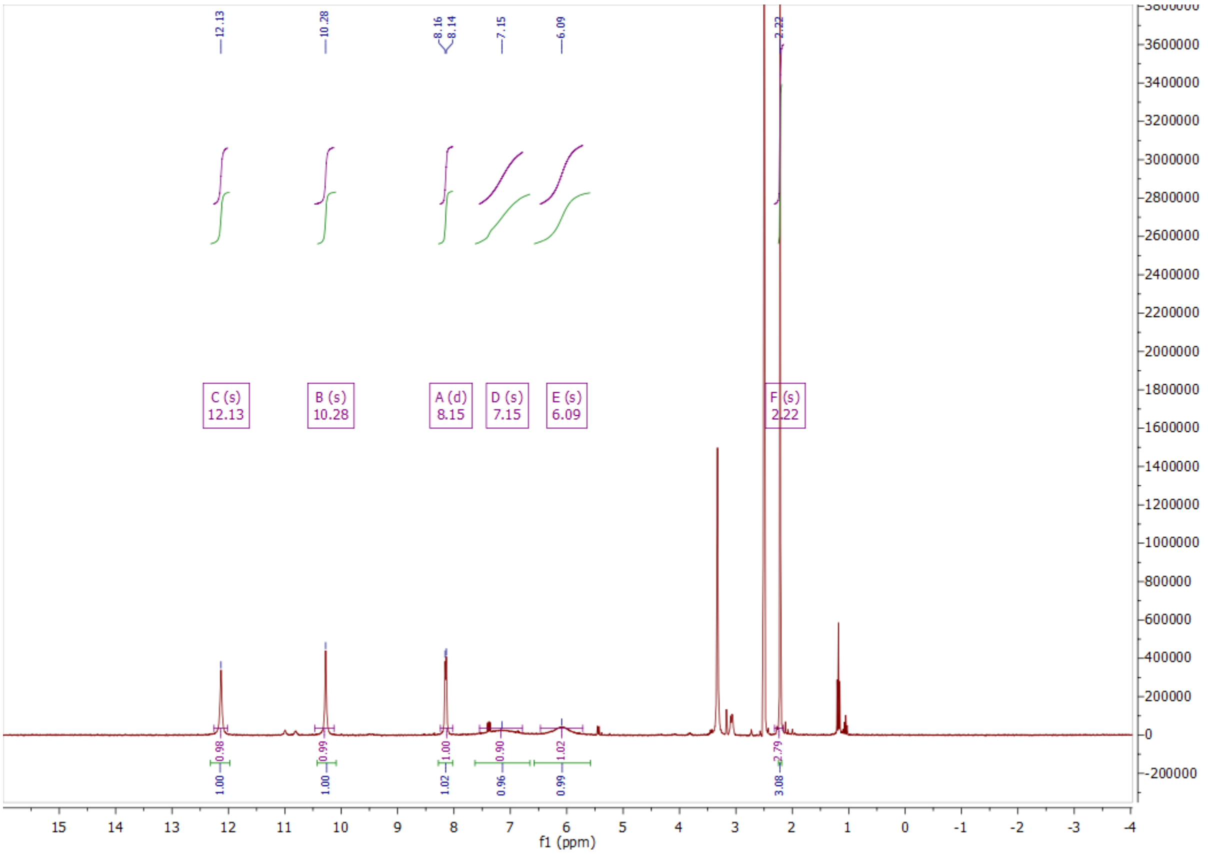

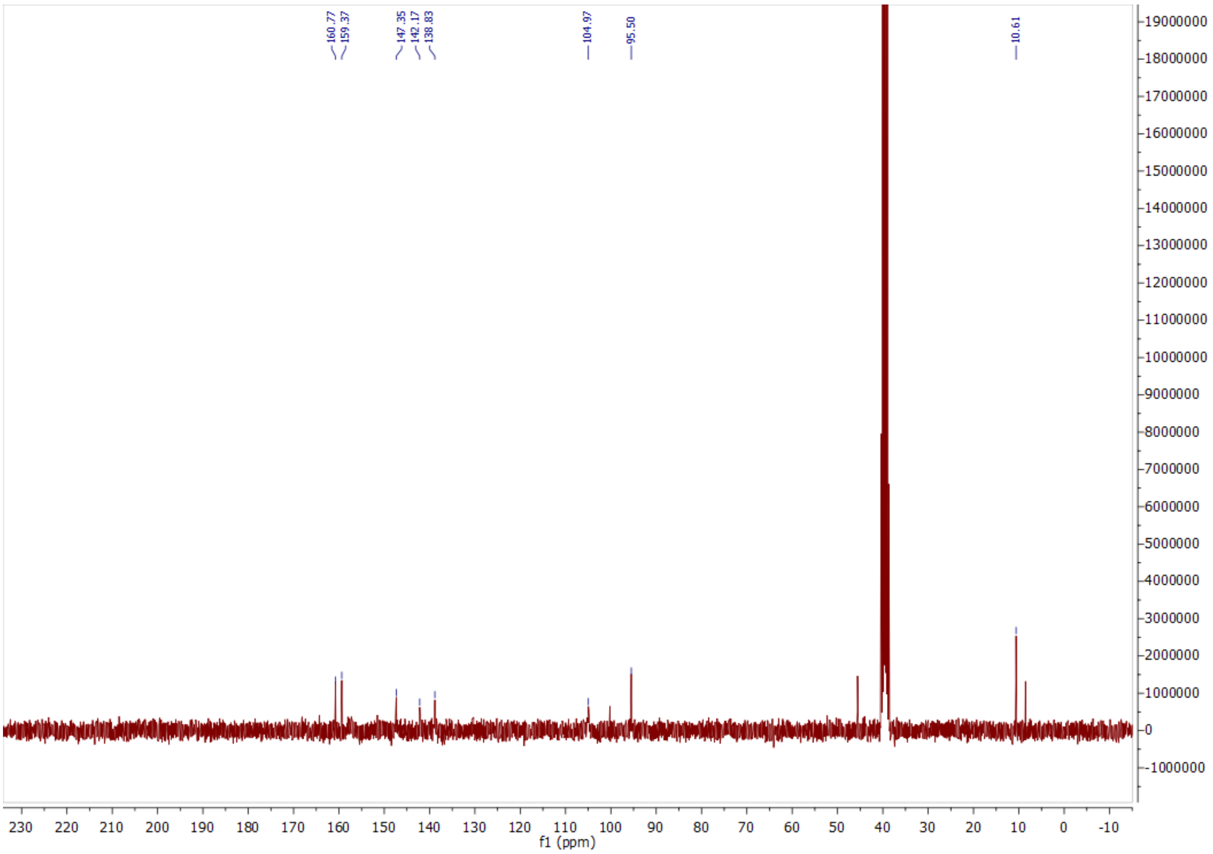

^{1}H and ^{13}C NMR data of compound **33**.

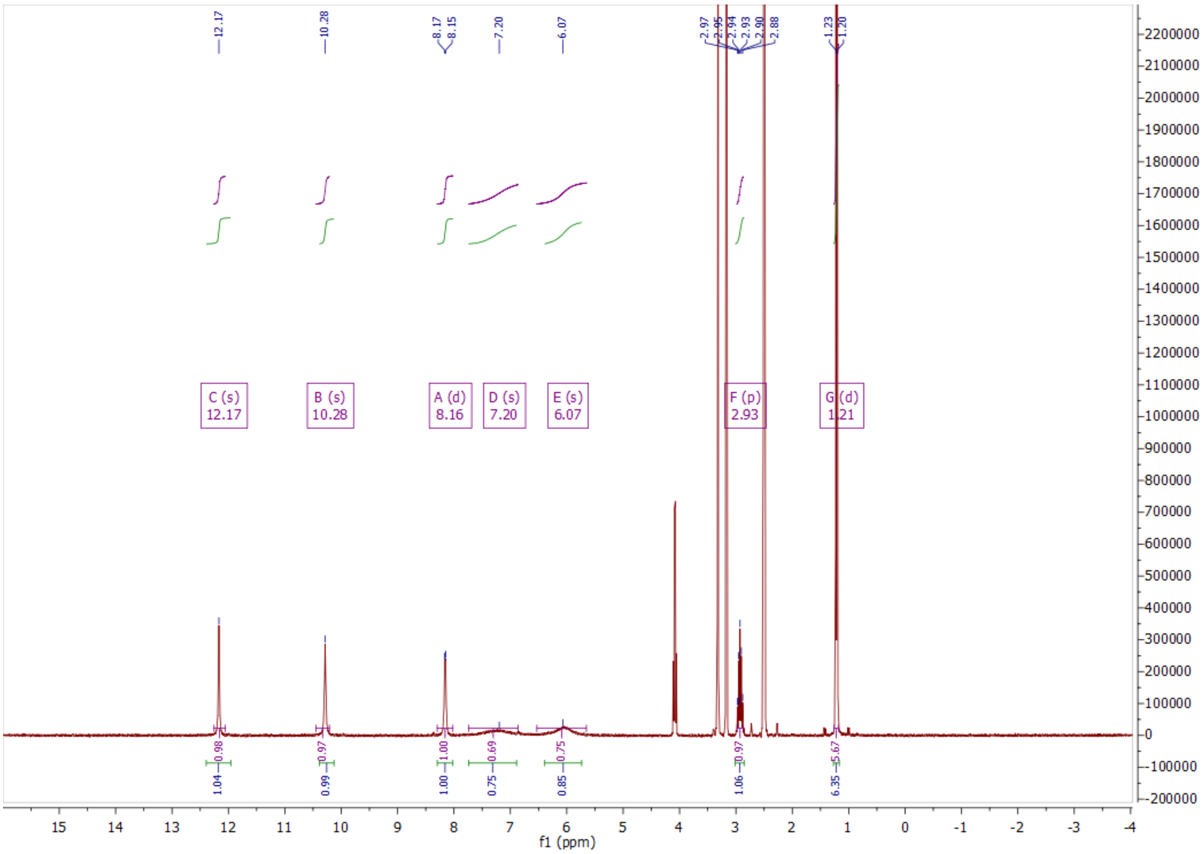

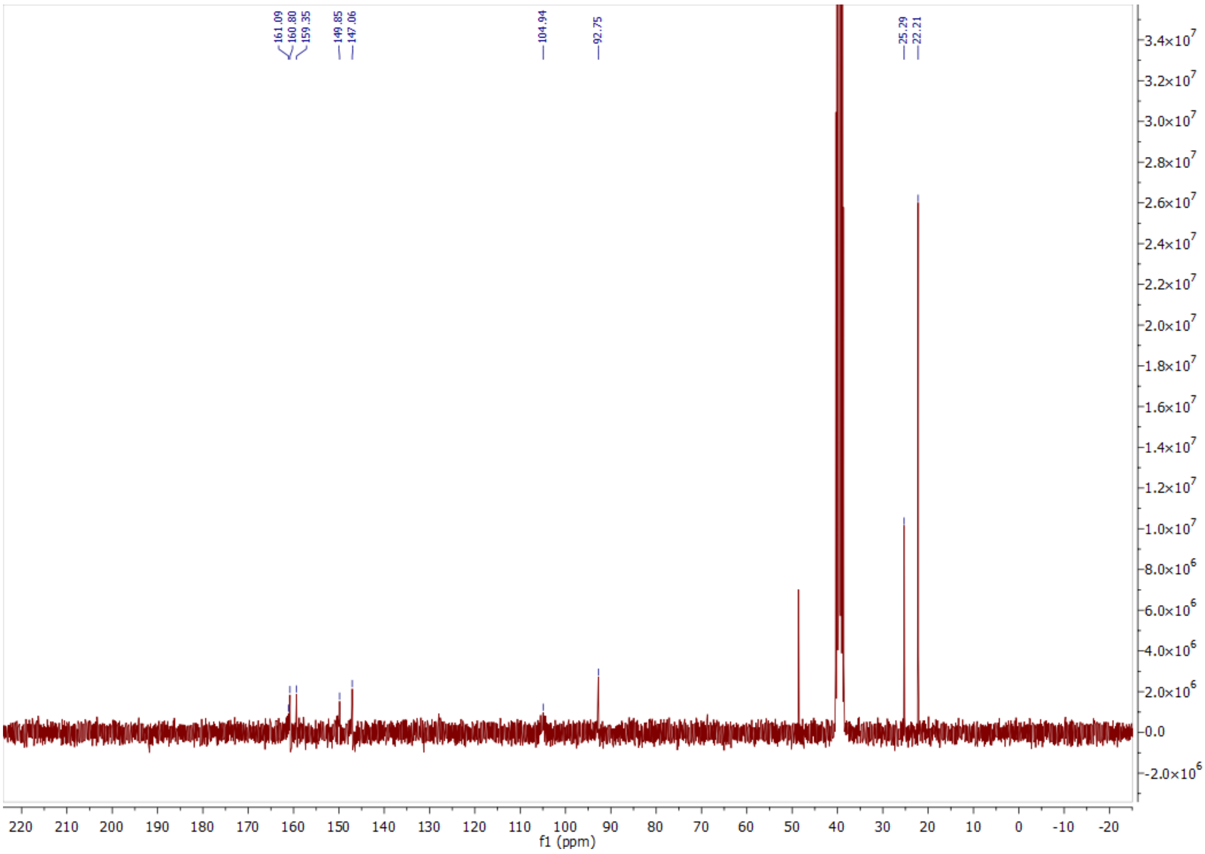

^{1}H and ^{13}C NMR data of compound **34**.

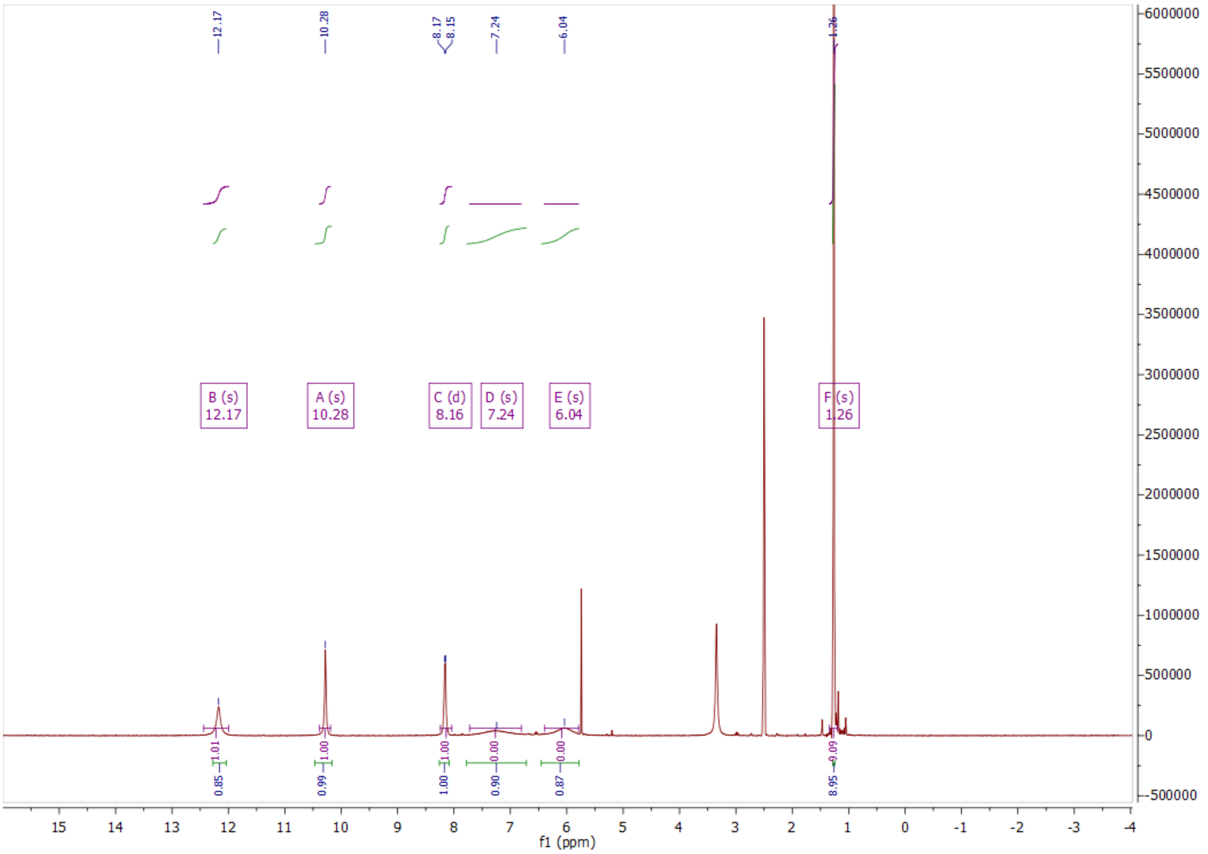

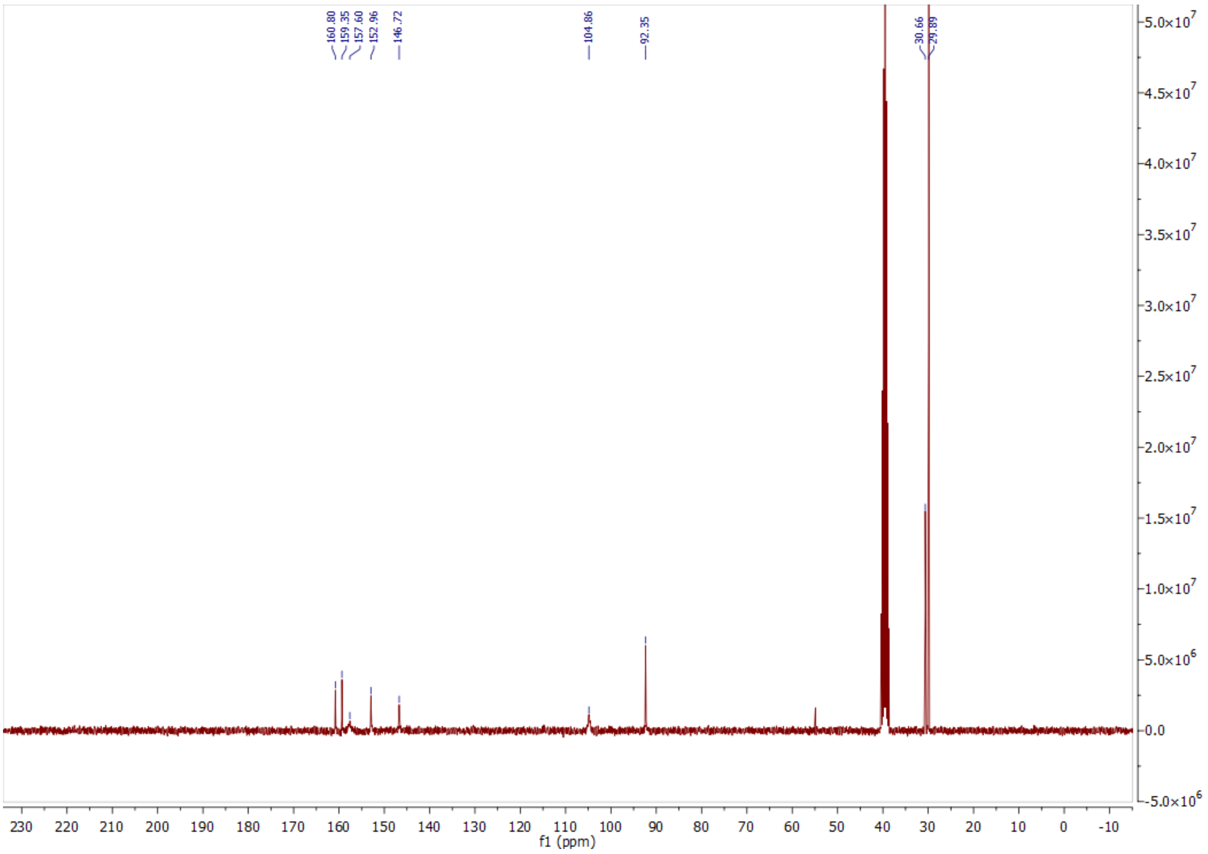

^{1}H and ^{13}C NMR data of compound **35**.

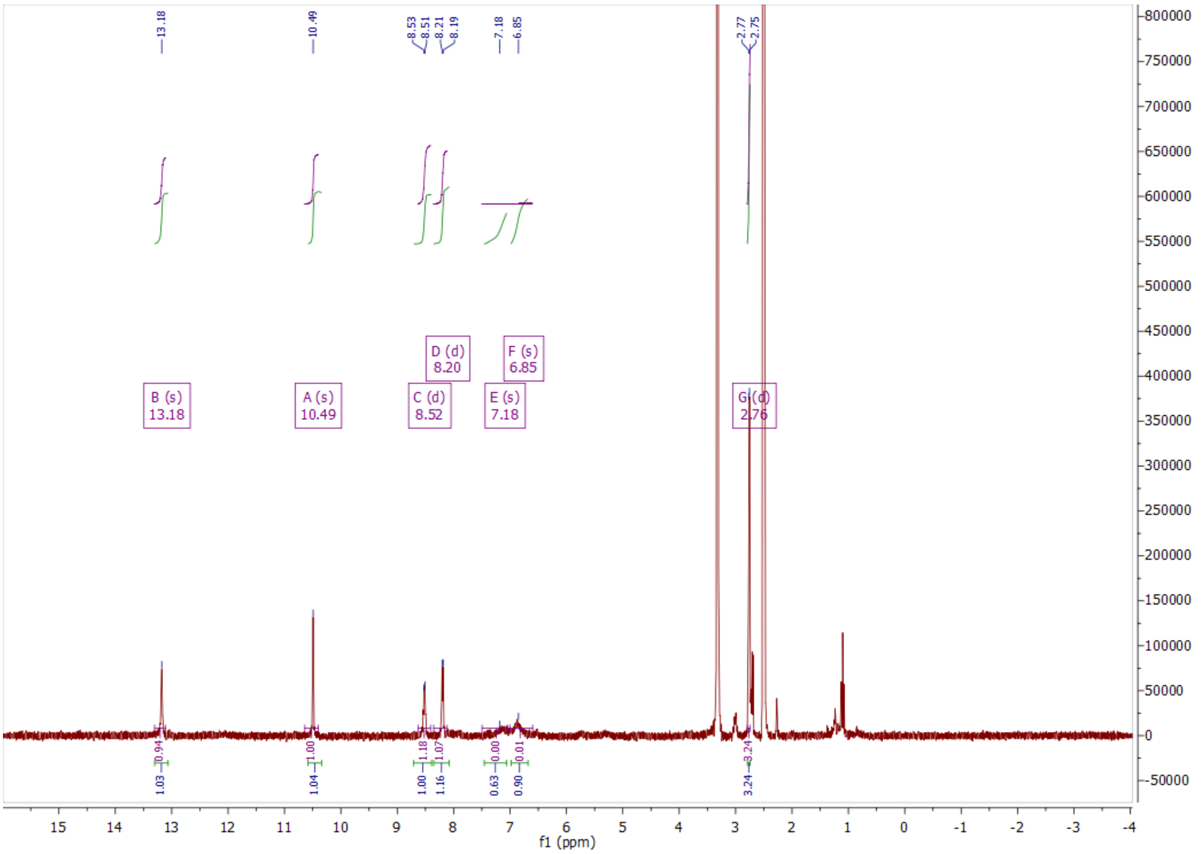

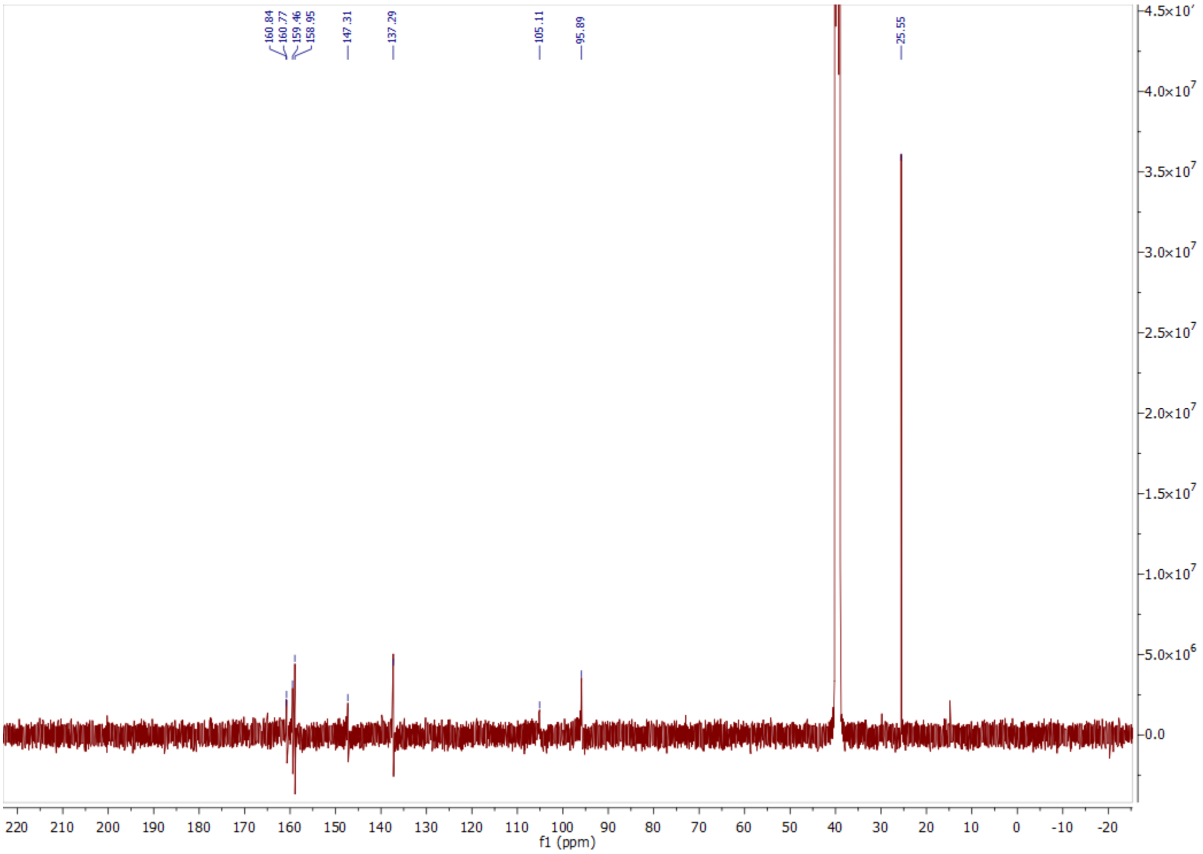

^{1}H and ^{13}C NMR data of compound **37**.

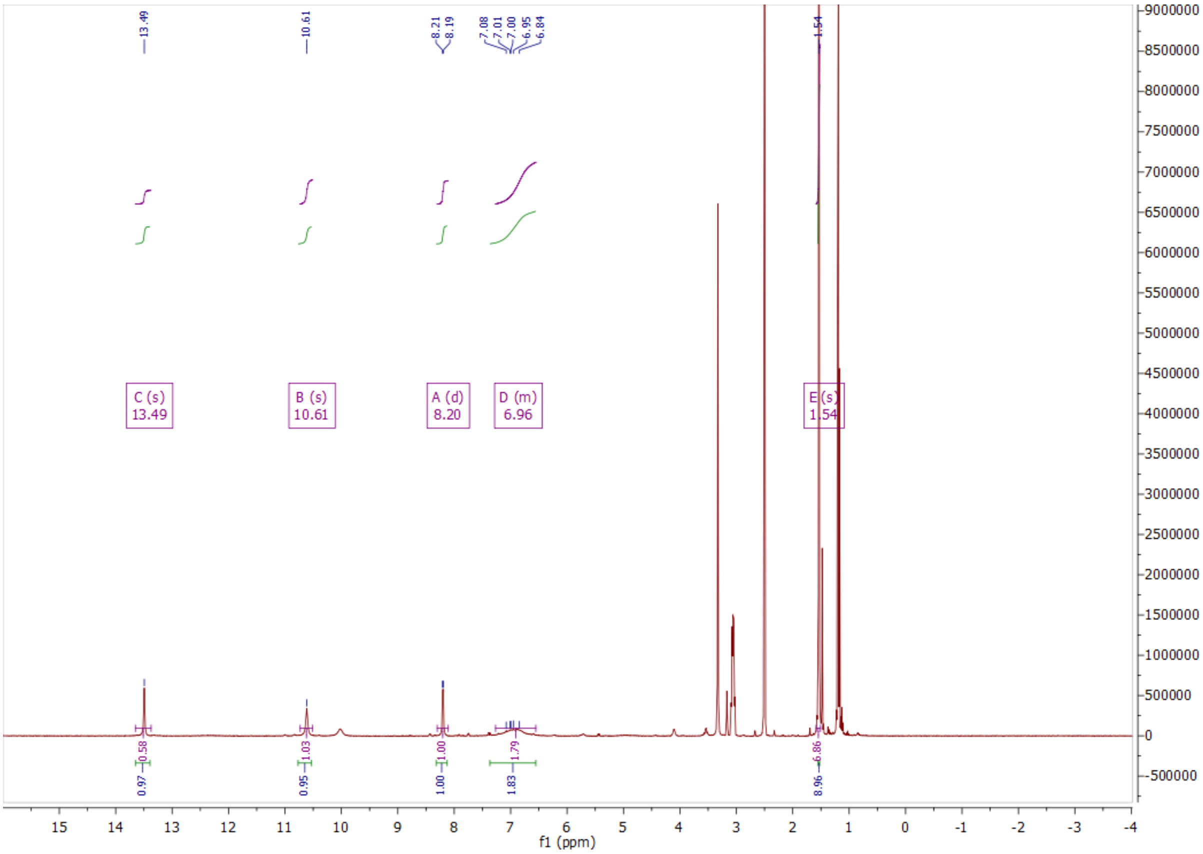

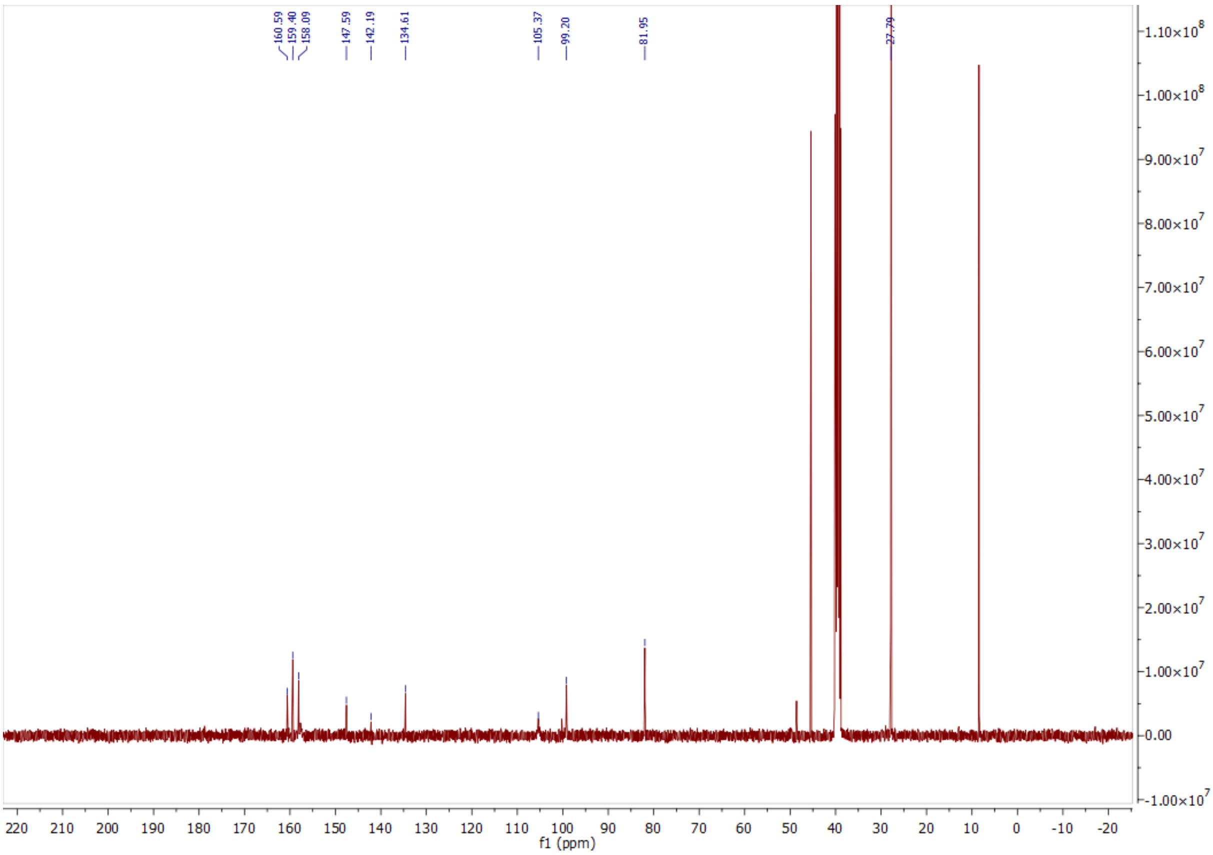

^{1}H, ^{13}C NMR and HPLC data of compound **38a**.

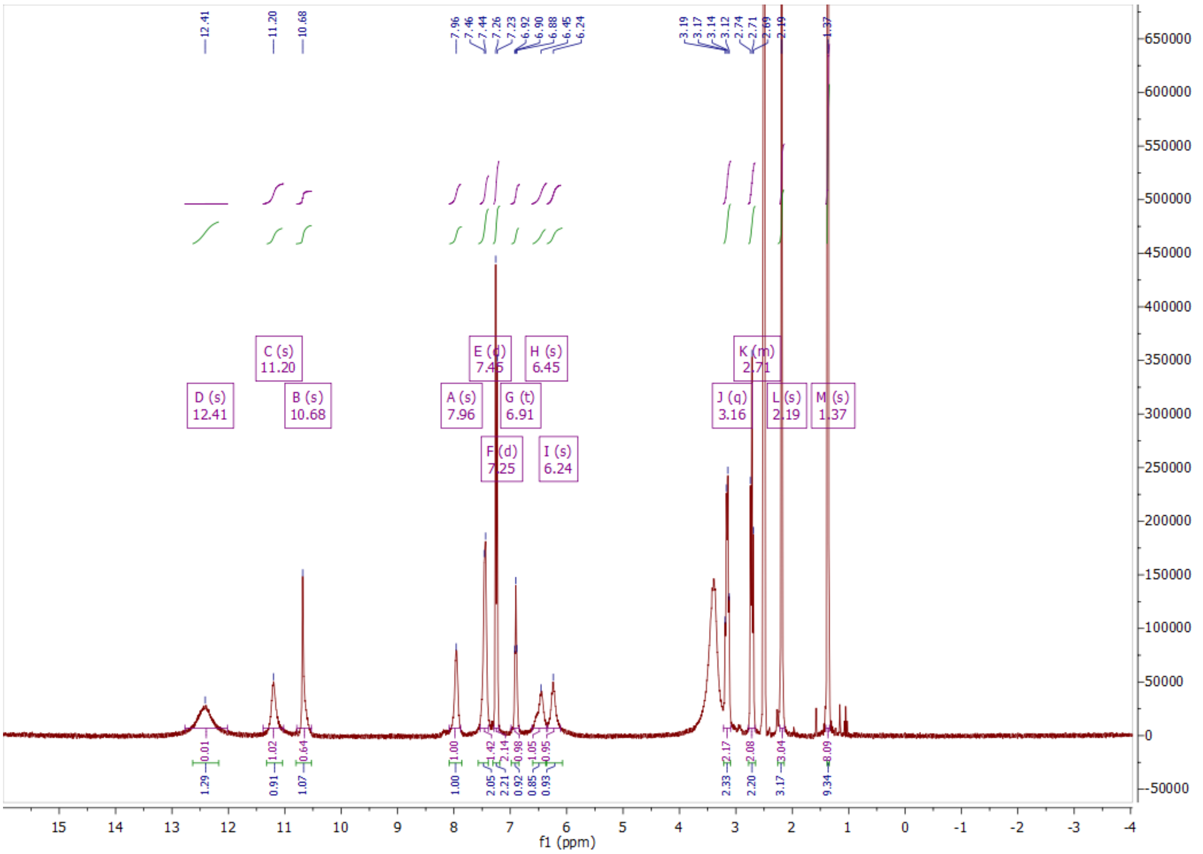

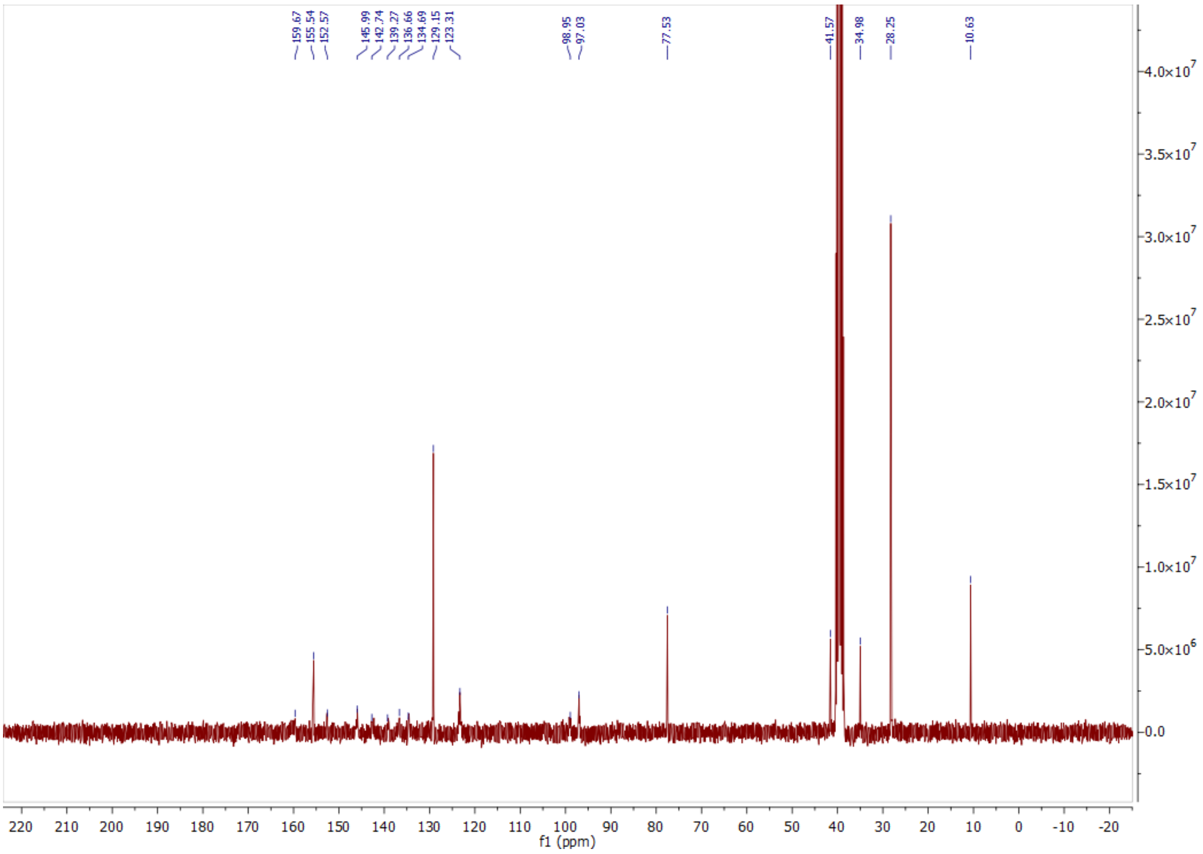

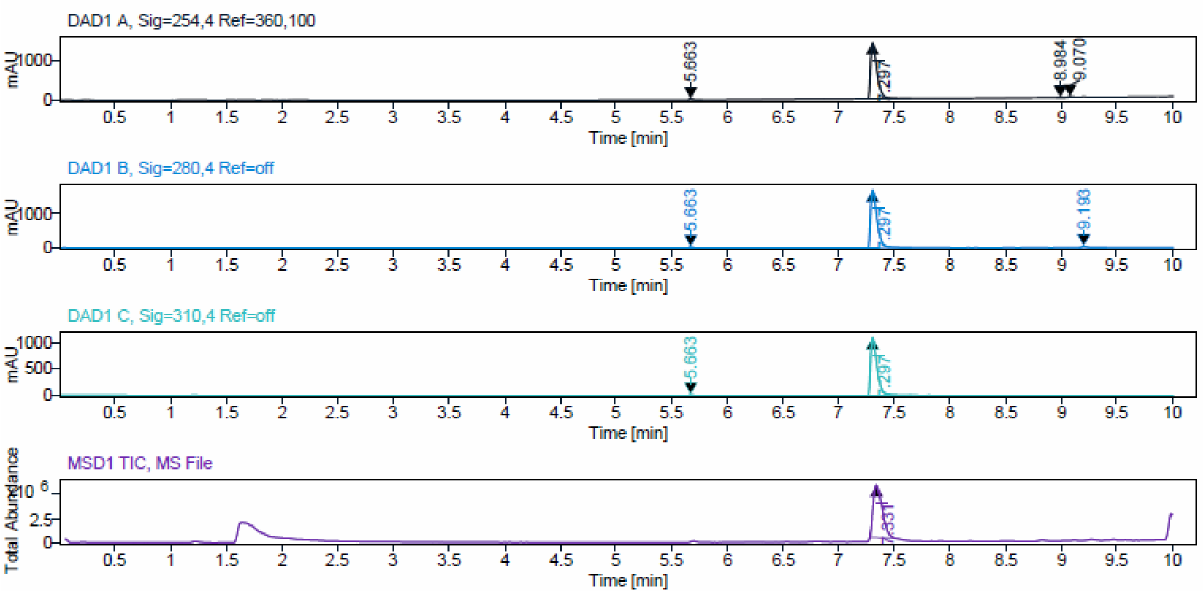

Sample Purity

Signal Description DAD1 A, Sig=254,4 Ref=360,100

Sample Name	Name	RT	Width	Area	Area%	Height
JA269_Feststoff		5.663	0.025	49.1302	0.85	26.7319
JA269_Feststoff		7.297	0.060	5665.3062	97.71	1414.4751
JA269_Feststoff		8.984	0.039	8.0375	0.14	4.1229
JA269_Feststoff		9.070	0.060	75.8784	1.31	21.5400

Max Area% 97.705

UV Signal Purity>95% Pass

Signal Description DAD1 B, Sig=280,4 Ref=off

Sample Name	Name	RT	Width	Area	Area%	Height
JA269_Feststoff		5.663	0.025	58.0753	0.80	30.9809
JA269_Feststoff		7.297	0.060	7042.7925	97.23	1756.3060
JA269_Feststoff		9.193	0.043	142.6468	1.97	46.1915

Max Area% 97.229

UV Signal Purity>95% Pass

Signal Description DAD1 C, Sig=310,4 Ref=off

Sample Name	Name	RT	Width	Area	Area%	Height
JA269_Feststoff		5.663	0.025	34.1099	0.76	17.9979
JA269_Feststoff		7.297	0.060	4446.4482	99.24	1108.9093

Max Area% 99.239

UV Signal Purity>95% Pass

^{1}H, ^{13}C NMR and HPLC data of compound **38b**.

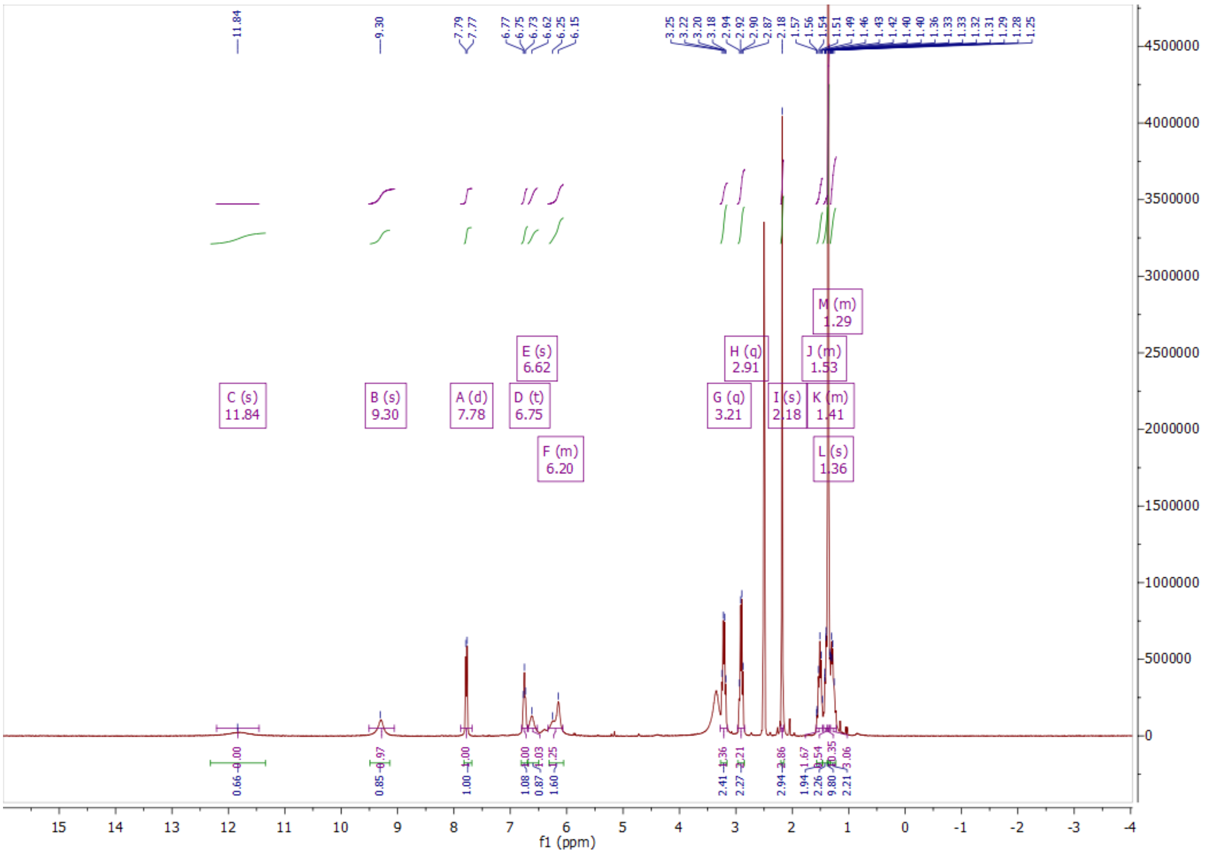

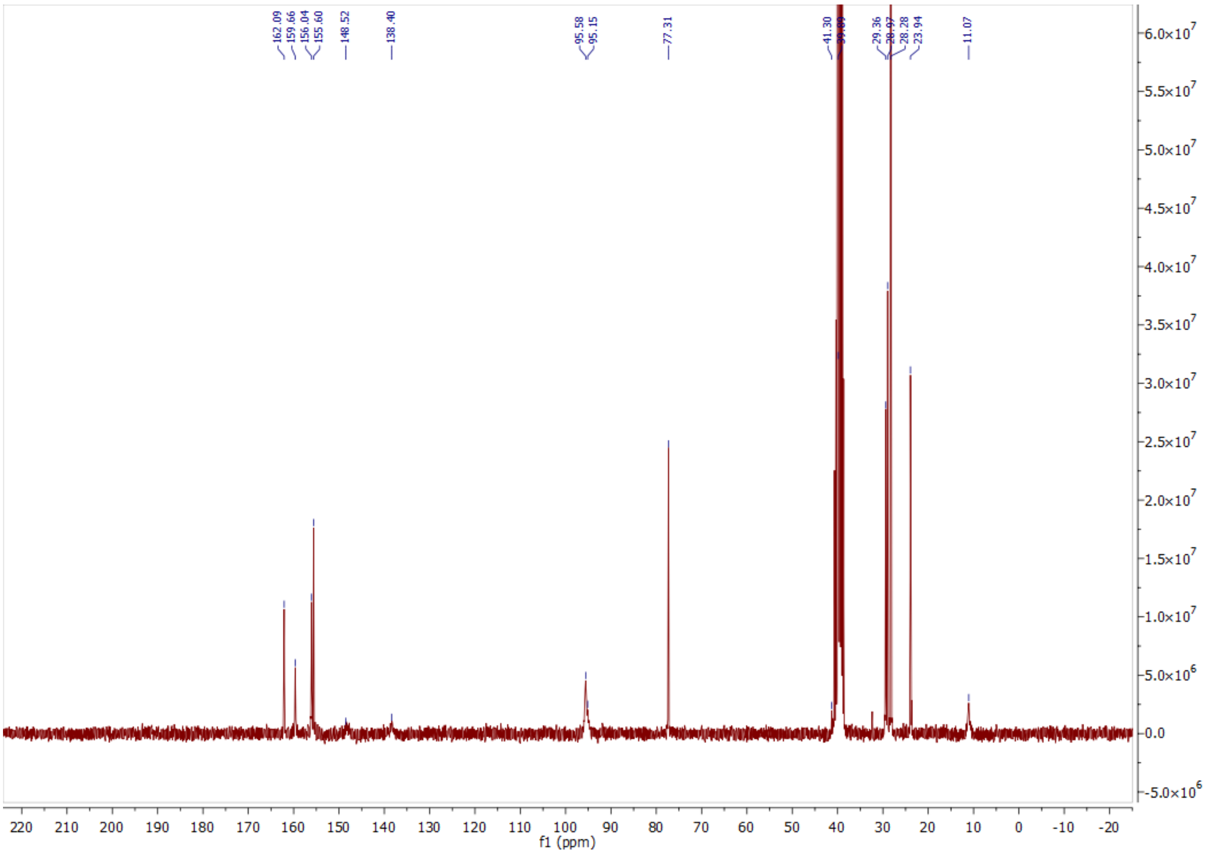

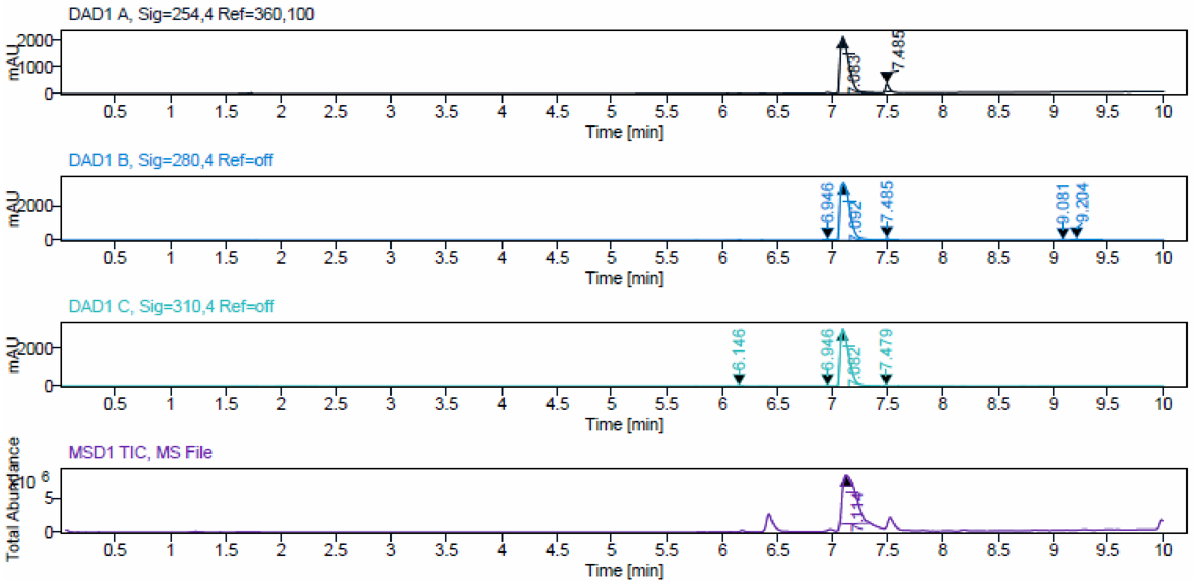

Sample Purity

Signal Description DAD1 A, Sig=254,4 Ref=360,100

Sample Name	Name	RT	Width	Area	Area%	Height
JA274_F14		7.083	0.086	11962.3975	95.07	2129.5413
JA274_F14		7.485	0.035	620.1653	4.93	289.2501

Max Area% 95.071

UV Signal Purity>95% Pass

Signal Description DAD1 B, Sig=280,4 Ref=off

Sample Name	Name	RT	Width	Area	Area%	Height
JA274_F14		6.946	0.034	90.2678	0.42	34.5130
JA274_F14		7.092	0.097	21126.9648	97.36	3391.2566
JA274_F14		7.485	0.041	316.6327	1.46	104.0925
JA274_F14		9.081	0.065	13.8192	0.06	3.4643
JA274_F14		9.204	0.044	153.0932	0.71	46.0402

Max Area% 97.356

UV Signal Purity>95% Pass

Signal Description DAD1 C, Sig=310,4 Ref=off

Sample Name	Name	RT	Width	Area	Area%	Height
JA274_F14		6.146	0.051	84.2527	0.49	34.1474
JA274_F14		6.946	0.034	35.4767	0.21	14.0715
JA274_F14		7.082	0.085	17015.8066	99.02	3086.3950
JA274_F14		7.479	0.086	48.1190	0.28	9.4774

Max Area% 99.023

UV Signal Purity>95% Pass

^{1}H, ^{13}C NMR and HPLC data of compound **39a**.

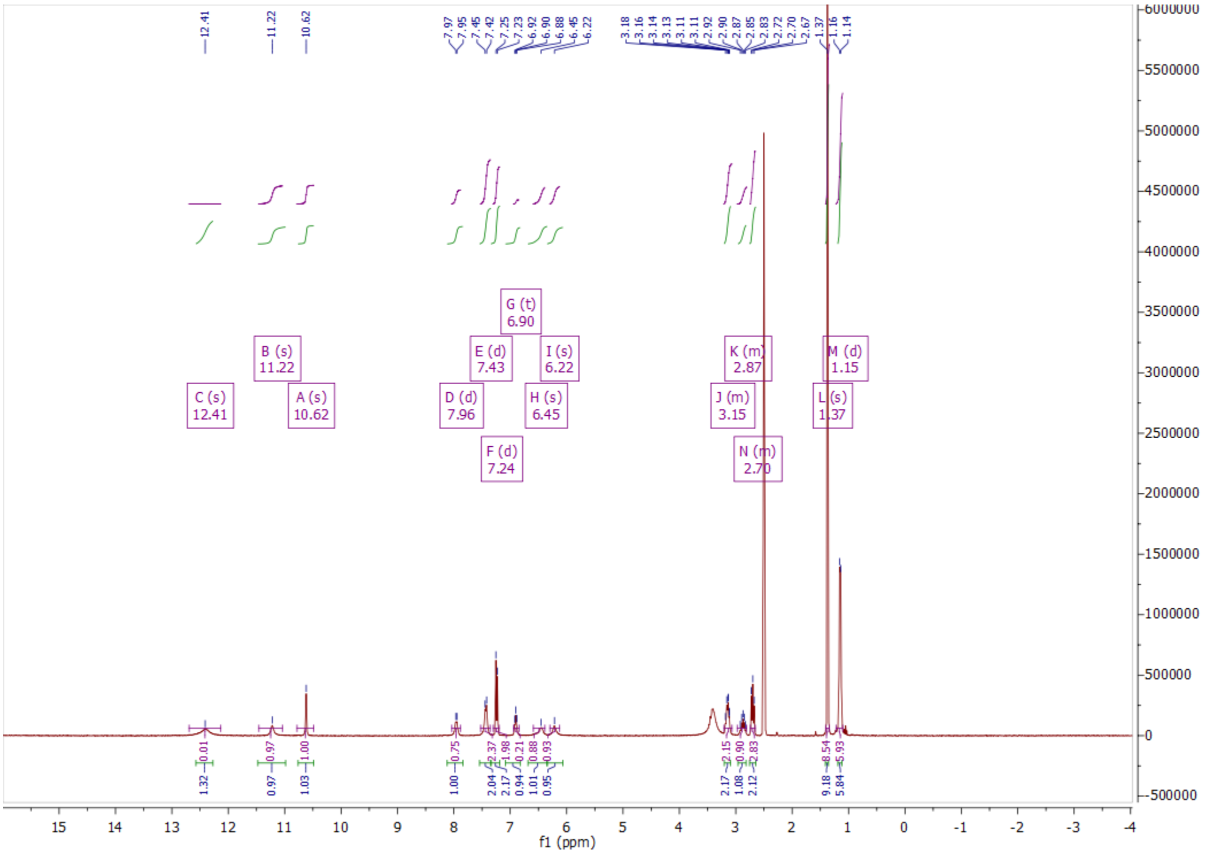

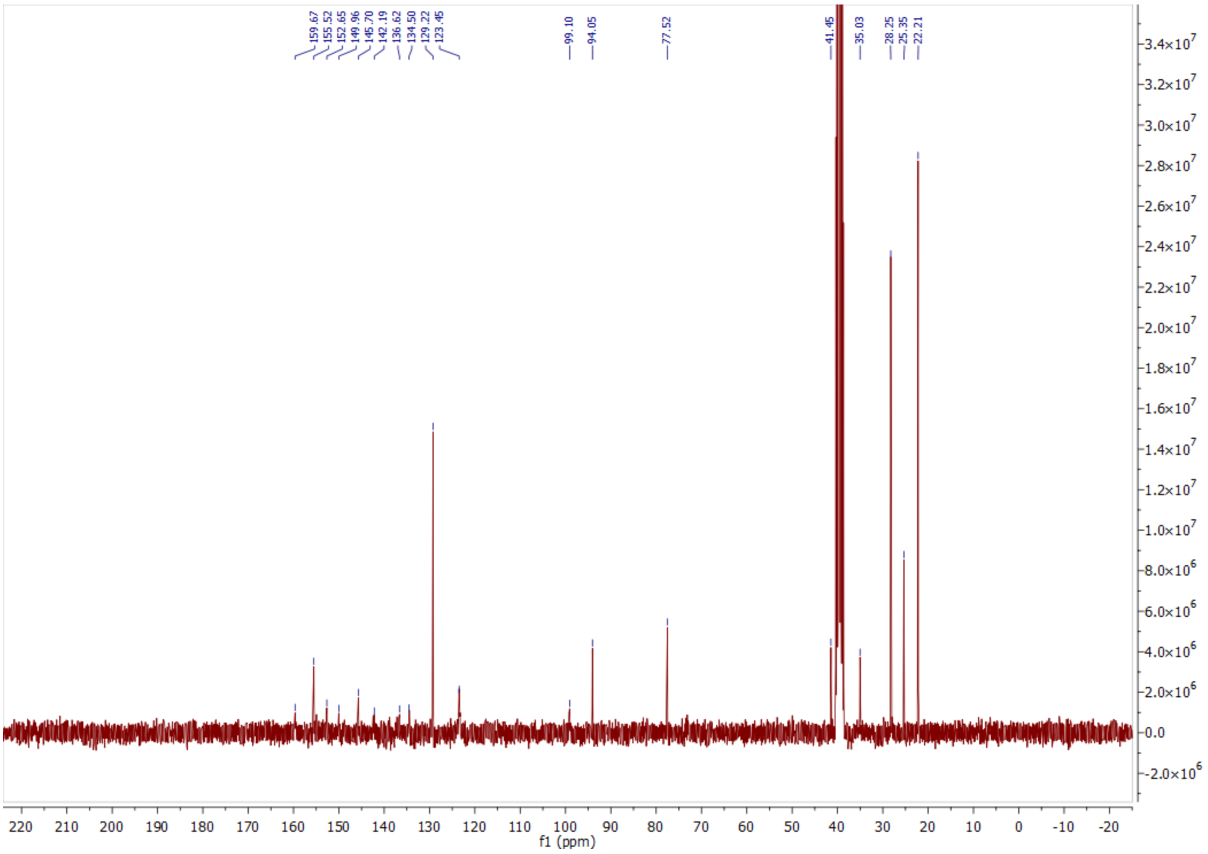

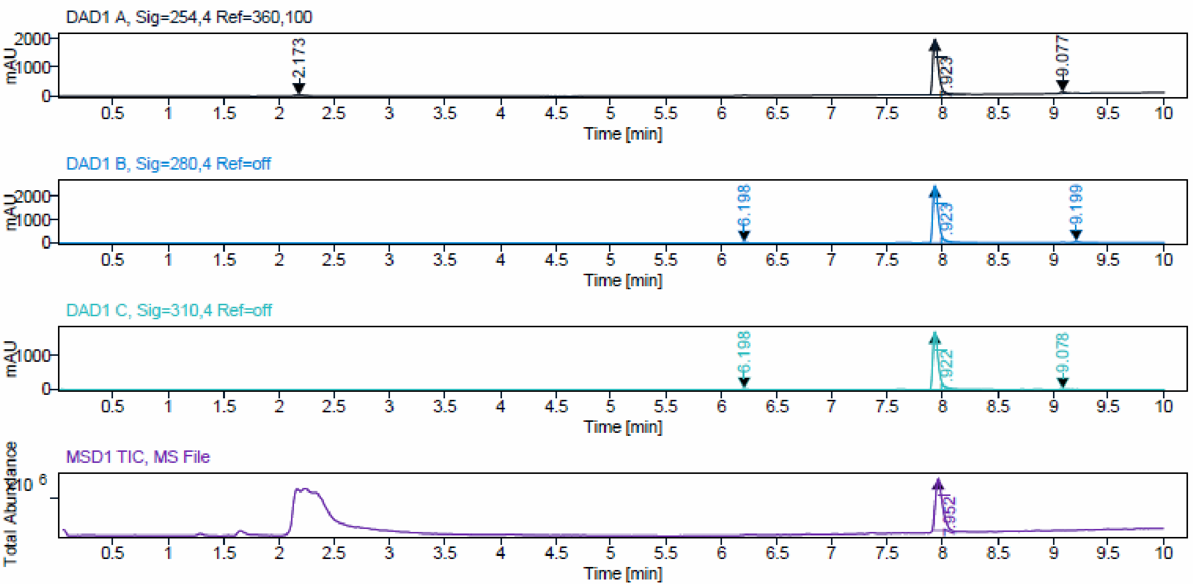

Sample Purity

Signal Description DAD1 A, Sig=254,4 Ref=360,100

Sample Name	Name	RT	Width	Area	Area%	Height
JA287_Feststoff		2.173	0.095	140.0351	1.94	25.7437
JA287_Feststoff		7.923	0.050	6910.7554	95.50	1987.5983
JA287_Feststoff		9.077	0.070	185.4305	2.56	50.0716

Max Area% 95.502

UV Signal Purity>95% Pass

Signal Description DAD1 B, Sig=280,4 Ref=off

Sample Name	Name	RT	Width	Area	Area%	Height
JA287_Feststoff		6.198	0.040	73.2381	0.82	25.3868
JA287_Feststoff		7.923	0.050	8692.6367	97.66	2527.7795
JA287_Feststoff		9.199	0.050	135.4978	1.52	39.5107

Max Area% 97.655

UV Signal Purity>95% Pass

Signal Description DAD1 C, Sig=310,4 Ref=off

Sample Name	Name	RT	Width	Area	Area%	Height
JA287_Feststoff		6.198	0.040	47.0880	0.81	16.2155
JA287_Feststoff		7.922	0.049	5732.7109	99.08	1680.5092
JA287_Feststoff		9.078	0.062	6.4065	0.11	1.6052

Max Area% 99.075

UV Signal Purity>95% Pass

^{1}H, ^{13}C NMR and HPLC data of compound **39b**.

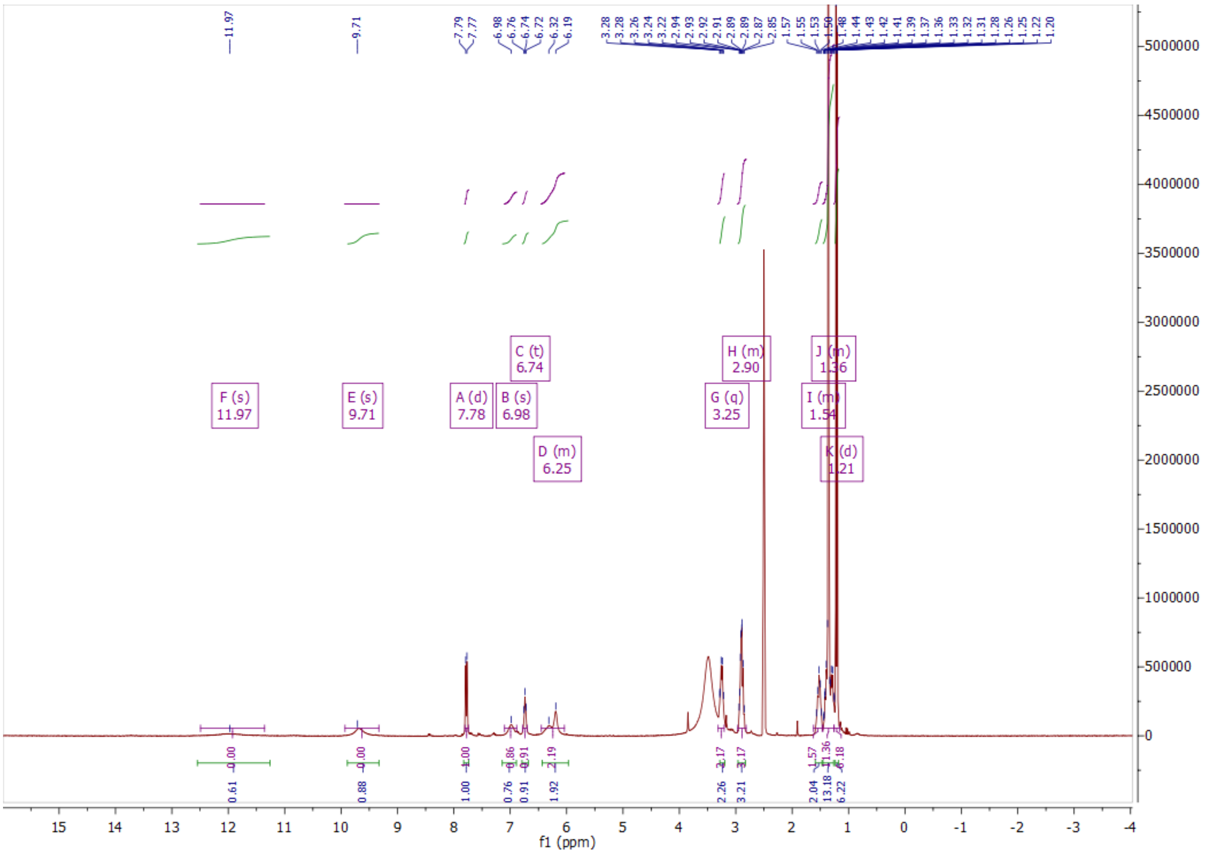

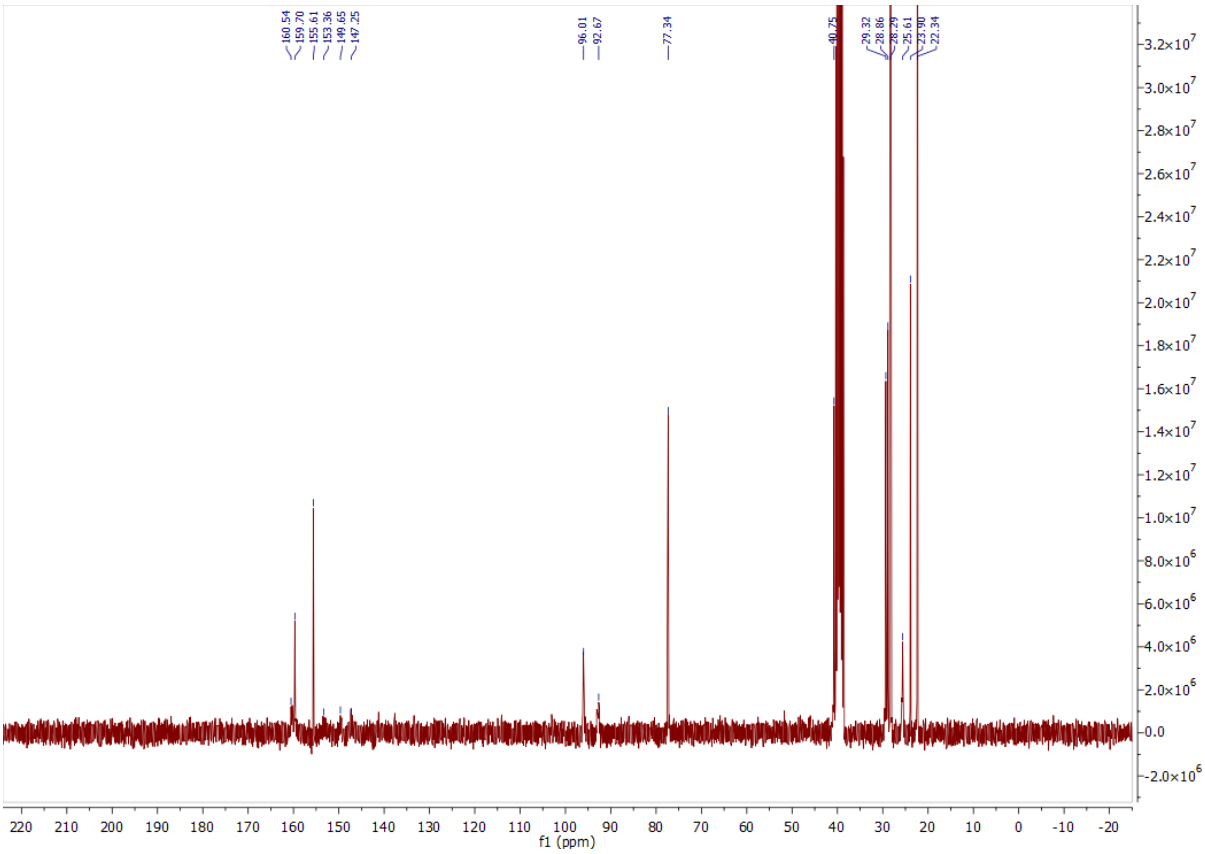

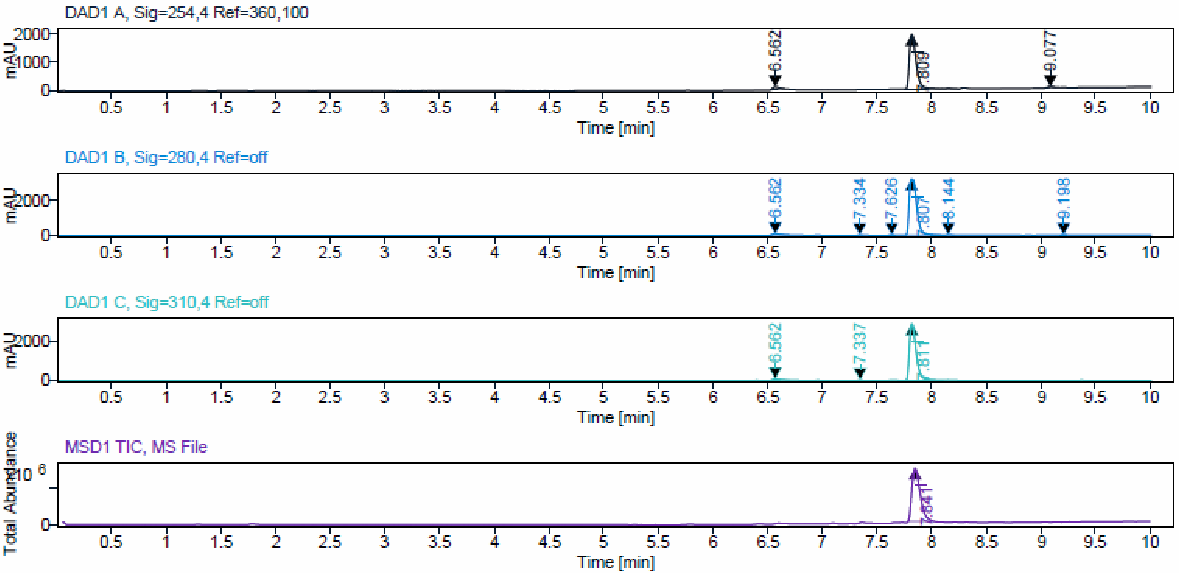

Sample Purity

Signal Description DAD1 A, Sig=254,4 Ref=360,100

Sample Name	Name	RT	Width	Area	Area%	Height
JA288_F12-14		6.562	0.077	359.5660	3.97	88.8404
JA288_F12-14		7.809	0.063	8615.5664	95.02	1984.7686
JA288_F12-14		9.077	0.056	91.9962	1.01	31.0333

Max Area% 95.020

UV Signal Purity>95% Pass

Signal Description DAD1 B, Sig=280,4 Ref=off

Sample Name	Name	RT	Width	Area	Area%	Height
JA288_F12-14		6.562	0.083	408.7689	2.54	86.3401
JA288_F12-14		7.334	0.034	47.6861	0.30	23.6398
JA288_F12-14		7.626	0.042	61.5826	0.38	27.4725
JA288_F12-14		7.807	0.072	15443.0850	96.05	3216.3123
JA288_F12-14		8.144	0.049	56.7902	0.35	22.0381
JA288_F12-14		9.198	0.043	59.6309	0.37	25.5464

Max Area% 96.054

UV Signal Purity>95% Pass

Signal Description DAD1 C, Sig=310,4 Ref=off

Sample Name	Name	RT	Width	Area	Area%	Height
JA288_F12-14		6.562	0.092	383.3391	2.93	71.3822
JA288_F12-14		7.337	0.045	60.5519	0.46	21.8484
JA288_F12-14		7.811	0.063	12659.7813	96.61	2981.6008

Max Area% 96.612

UV Signal Purity>95% Pass

^{1}H, ^{13}C NMR and HPLC data of compound **40a**.

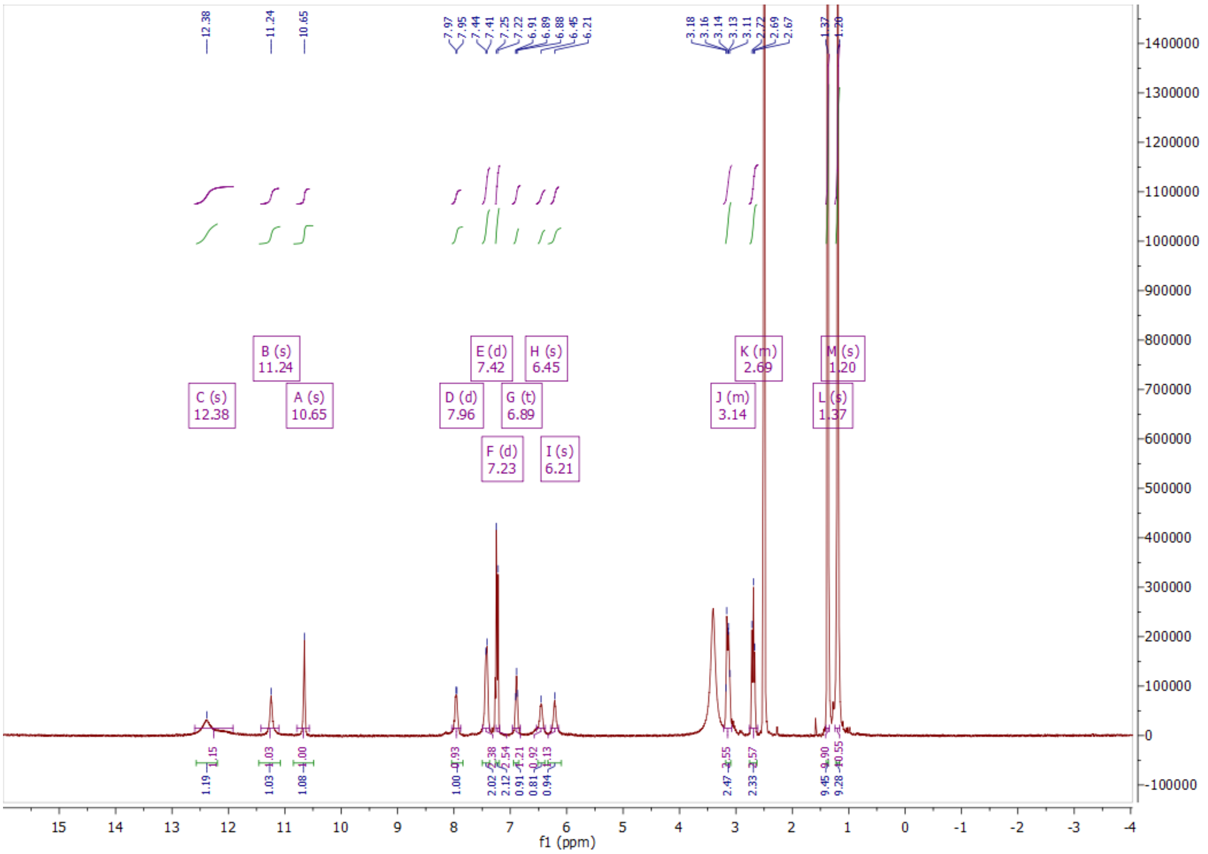

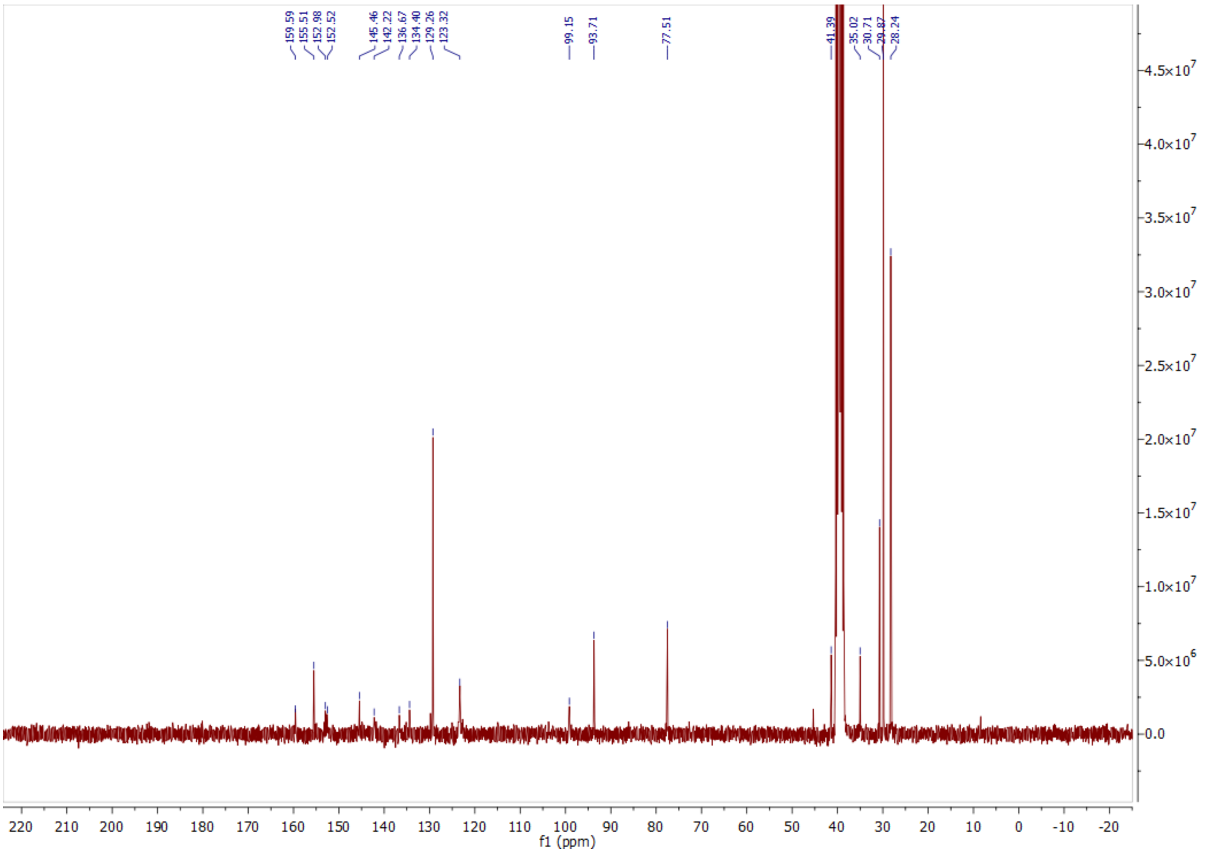

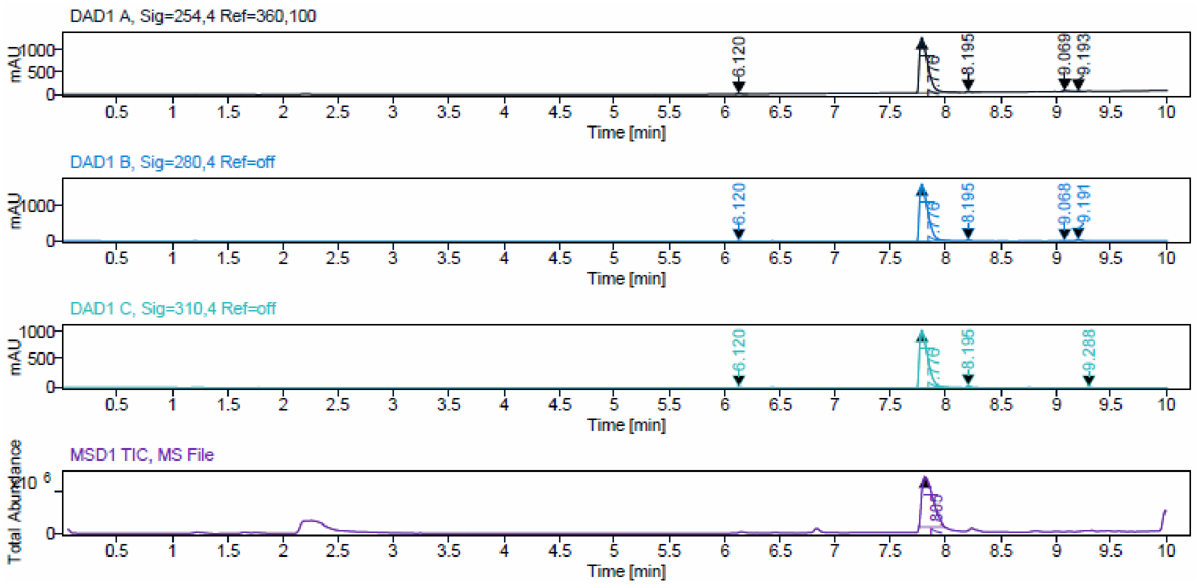

Sample Purity

Signal Description DAD1 A, Sig=254,4 Ref=360,100

Sample Name	Name	RT	Width	Area	Area%	Height
JA270		6.120	0.037	29.3321	0.43	12.1442
JA270		7.776	0.081	6570.1865	97.12	1244.2864
JA270		8.195	0.034	70.6574	1.04	27.1058
JA270		9.069	0.059	74.3764	1.10	21.1875
JA270		9.193	0.041	20.3194	0.30	7.9297

Max Area% 97.122

UV Signal Purity>95% Pass

Signal Description DAD1 B, Sig=280,4 Ref=off

Sample Name	Name	RT	Width	Area	Area%	Height
JA270		6.120	0.036	37.6890	0.43	14.8377
JA270		7.776	0.080	8435.6387	97.36	1598.6654
JA270		8.195	0.030	54.3853	0.63	29.6436
JA270		9.068	0.055	20.4446	0.24	6.0127
JA270		9.191	0.042	116.5159	1.34	43.7447

Max Area% 97.357

UV Signal Purity>95% Pass

Signal Description DAD1 C, Sig=310,4 Ref=off

Sample Name	Name	RT	Width	Area	Area%	Height
JA270		6.120	0.035	24.8144	0.43	9.5936
JA270		7.776	0.080	5636.7827	97.82	1065.5066
JA270		8.195	0.034	82.3680	1.43	30.7904
JA270		9.288	0.039	18.2703	0.32	6.6721

Max Area% 97.823

UV Signal Purity>95% Pass

^{1}H, ^{13}C NMR and HPLC data of compound **40b**.

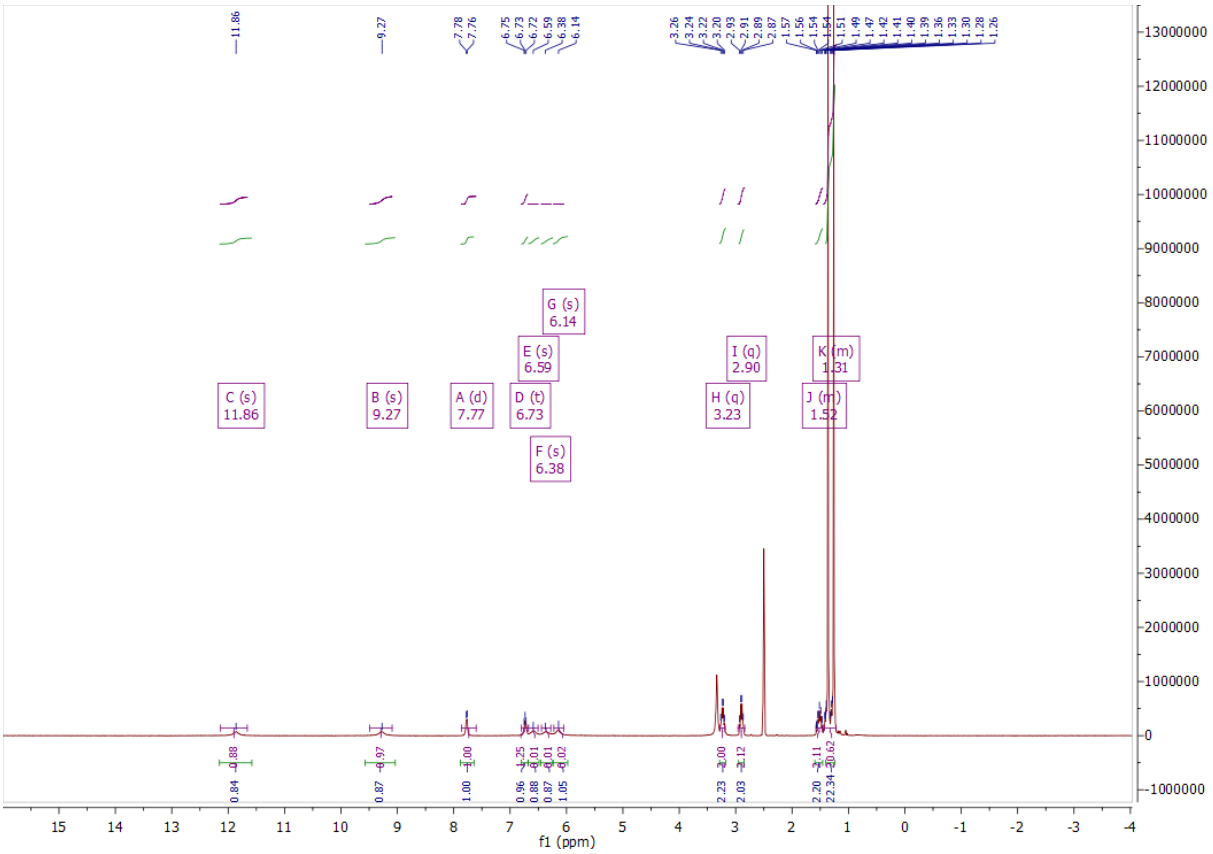

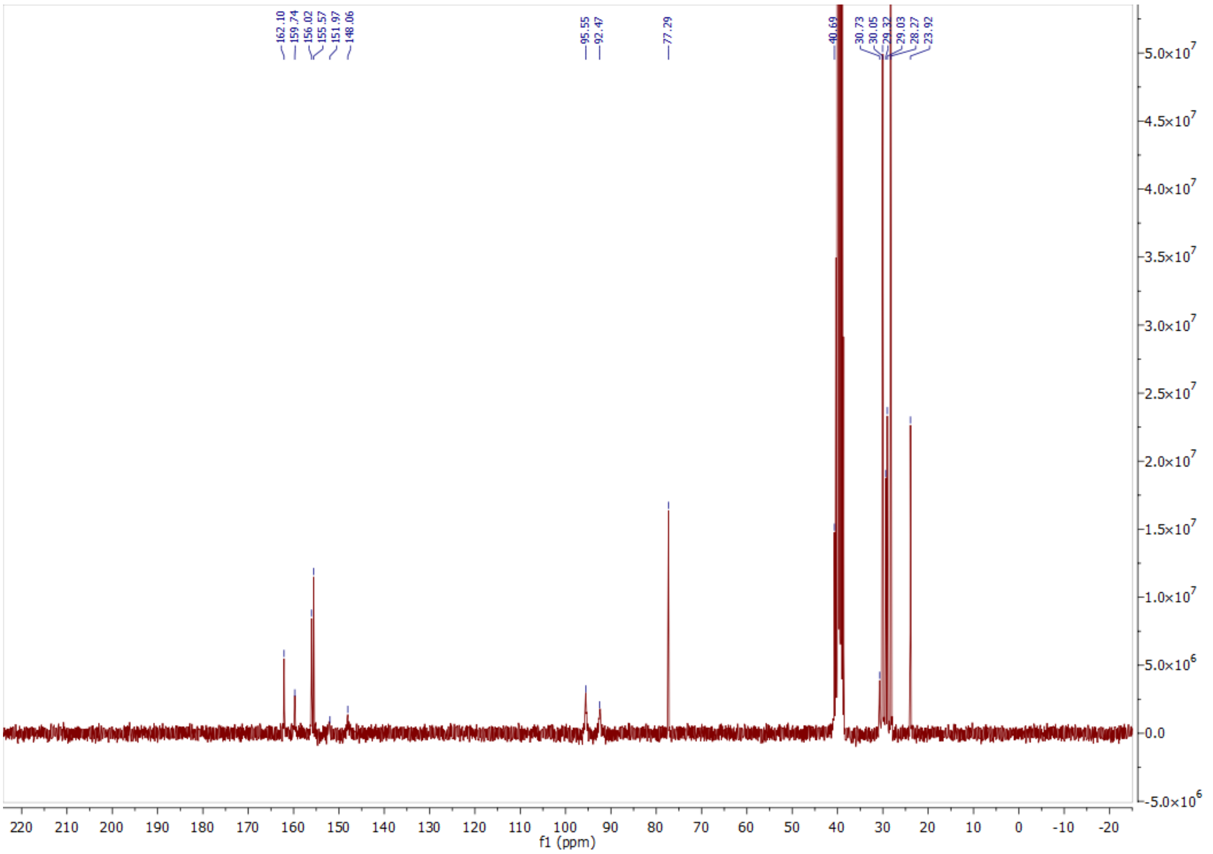

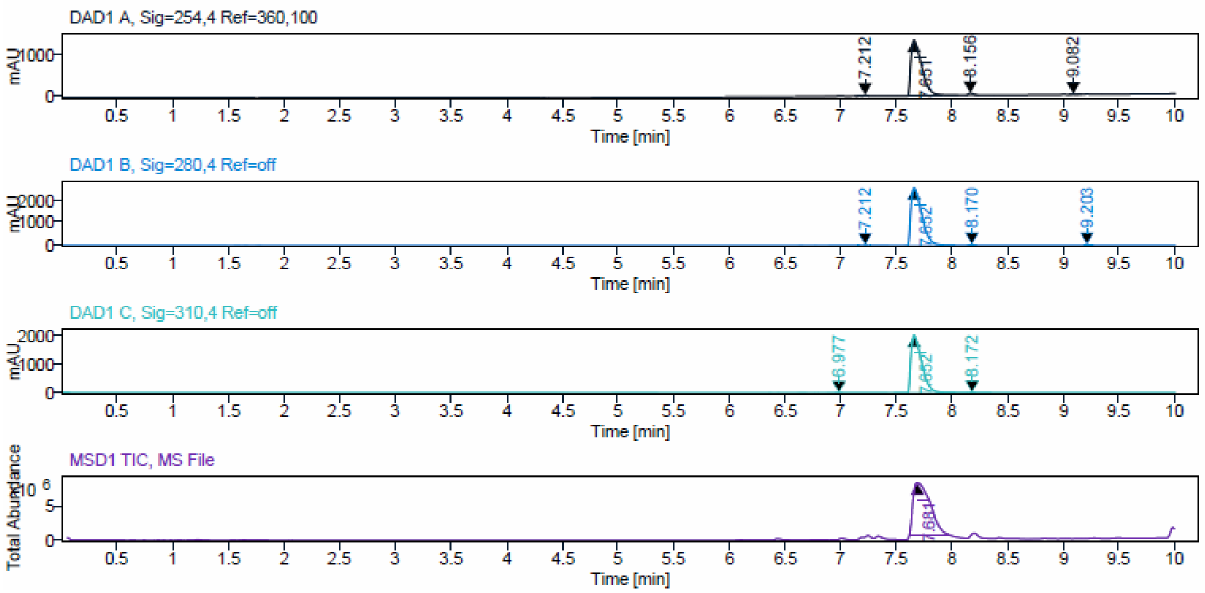

Sample Purity

Signal Description DAD1 A, Sig=254,4 Ref=360,100

Sample Name	Name	RT	Width	Area	Area%	Height
JA275_F12		7.212	0.037	80.7077	0.89	22.9511
JA275_F12		7.651	0.105	8757.3848	96.84	1311.1350
JA275_F12		8.156	0.054	148.5106	1.64	44.7173
JA275_F12		9.082	0.100	56.2100	0.62	14.3488

Max Area% 96.844

UV Signal Purity>95% Pass

Signal Description DAD1 B, Sig=280,4 Ref=off

Sample Name	Name	RT	Width	Area	Area%	Height
JA275_F12		7.212	0.039	58.1602	0.32	15.2083
JA275_F12		7.652	0.107	17866.1797	98.68	2593.3777
JA275_F12		8.170	0.043	91.6798	0.51	33.6842
JA275_F12		9.203	0.039	88.7779	0.49	37.6789

Max Area% 98.682

UV Signal Purity>95% Pass

Signal Description DAD1 C, Sig=310,4 Ref=off

Sample Name	Name	RT	Width	Area	Area%	Height
JA275_F12		6.977	0.032	15.1017	0.11	7.0030
JA275_F12		7.652	0.105	14017.4668	99.46	2056.5493
JA275_F12		8.172	0.044	61.0053	0.43	15.7799

Max Area% 99.460

UV Signal Purity>95% Pass

^{1}H, ^{13}C NMR and HPLC data of compound **40c**.

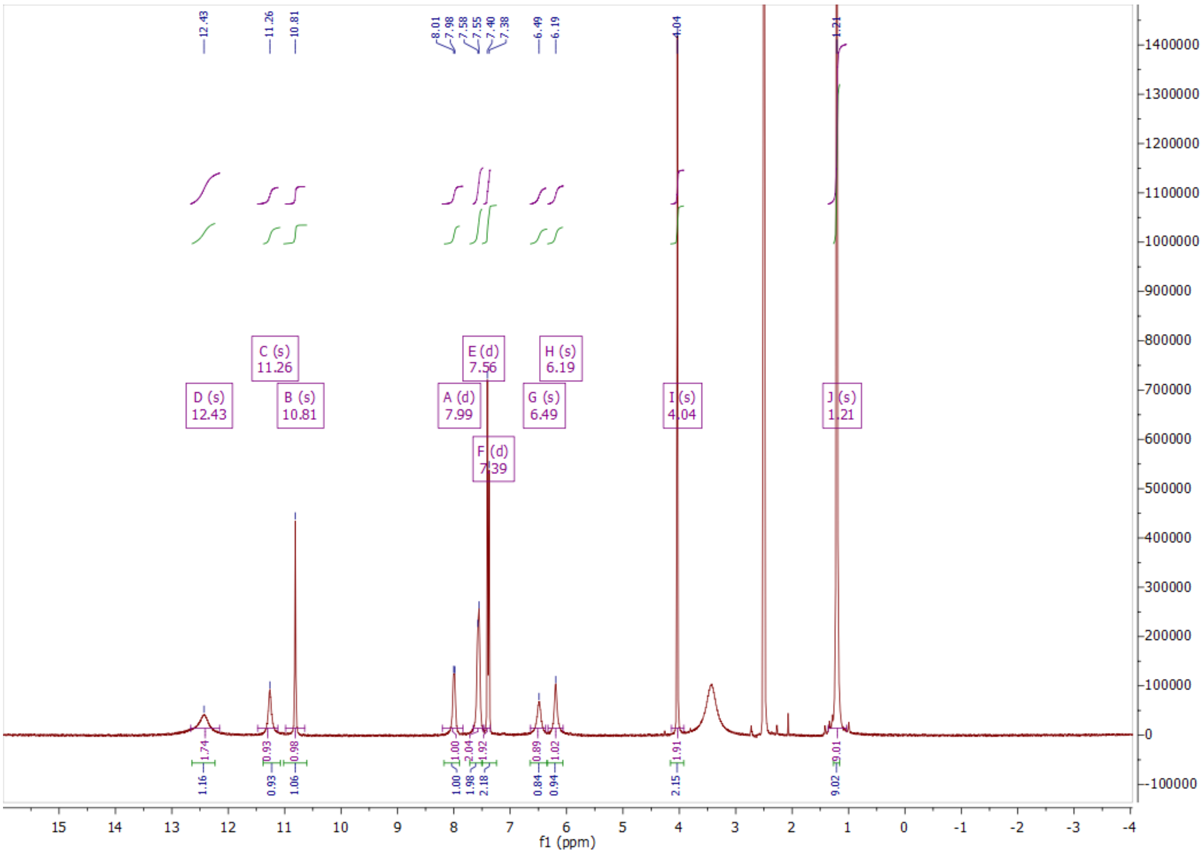

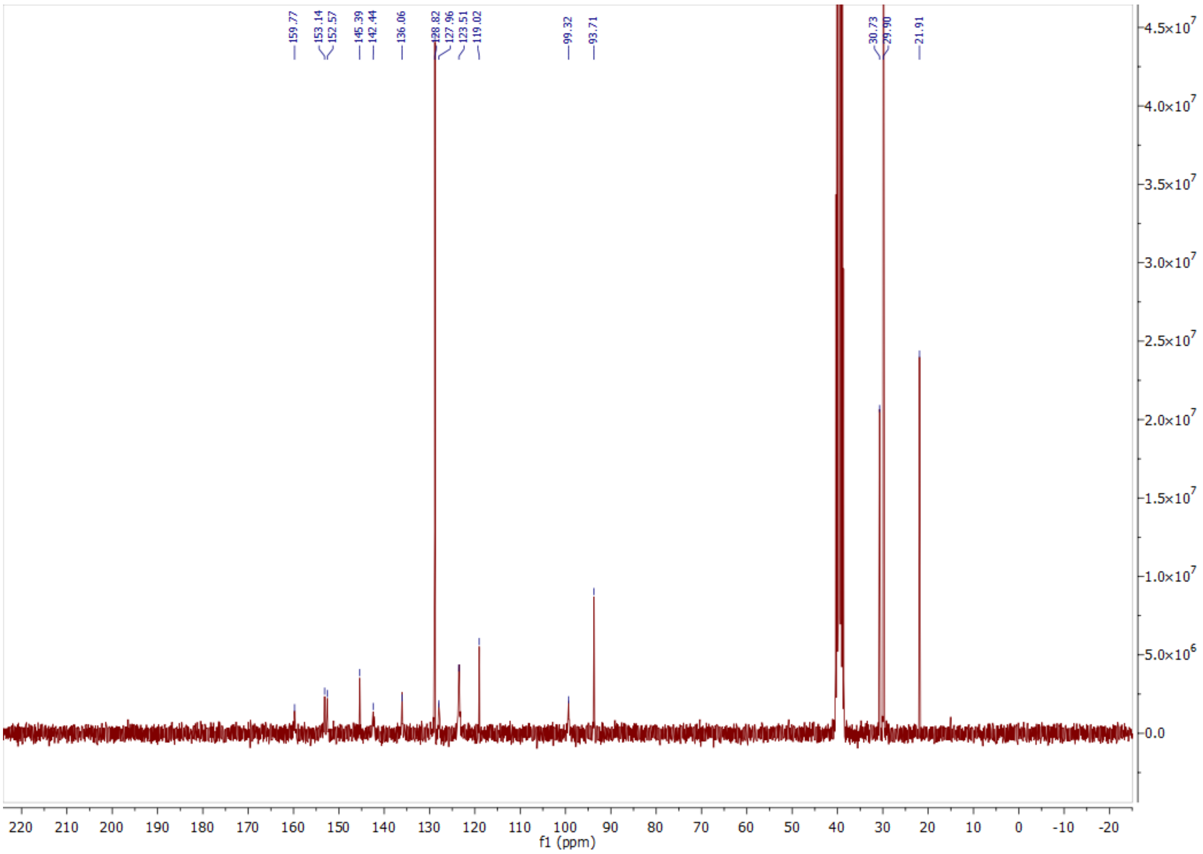

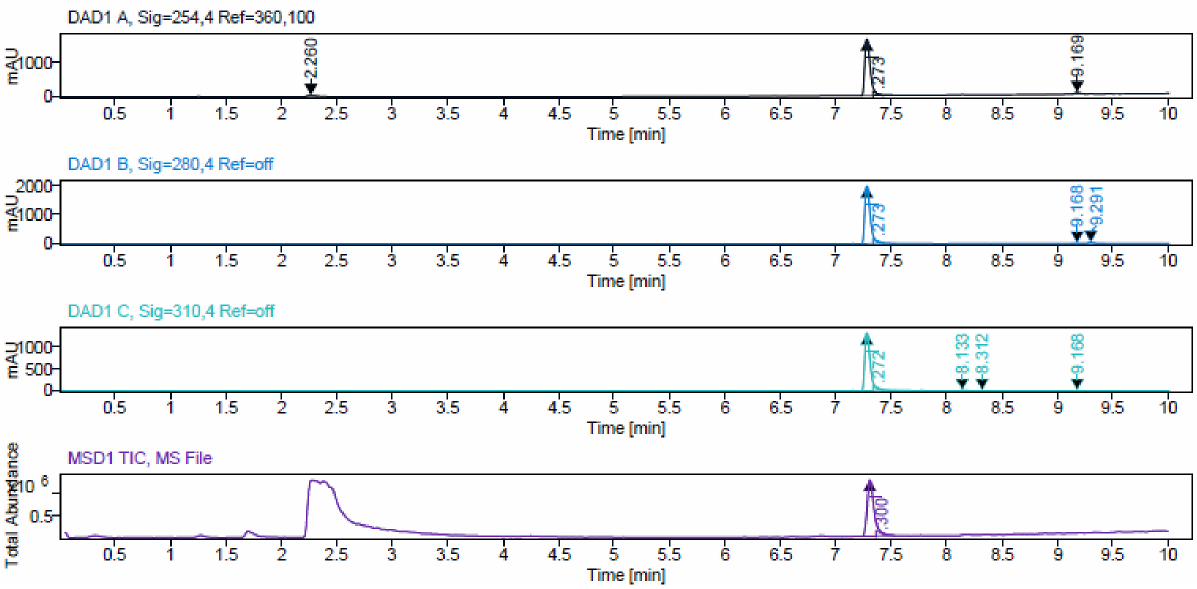

Sample Purity

Signal Description DAD1 A, Sig=254,4 Ref=360,100

Sample Name	Name	RT	Width	Area	Area%	Height
JA296_gew		2.260	0.083	125.4498	2.17	28.1138
JA296_gew		7.273	0.049	5515.3525	95.39	1651.4106
JA296_gew		9.169	0.055	140.8497	2.44	41.8234

Max Area% 95.394

UV Signal Purity>95% Pass

Signal Description DAD1 B, Sig=280,4 Ref=off

Sample Name	Name	RT	Width	Area	Area%	Height
JA296_gew		7.273	0.049	6727.2666	97.21	2031.8427
JA296_gew		9.168	0.056	43.6122	0.63	12.8363
JA296_gew		9.291	0.056	149.3973	2.16	41.2774

Max Area% 97.211

UV Signal Purity>95% Pass

Signal Description DAD1 C, Sig=310,4 Ref=off

Sample Name	Name	RT	Width	Area	Area%	Height
JA296_gew		7.272	0.048	4439.2456	99.33	1344.7618
JA296_gew		8.133	0.063	18.2635	0.41	4.4087
JA296_gew		8.312	0.047	5.2892	0.12	1.6603
JA296_gew		9.168	0.058	6.3300	0.14	1.6265

Max Area% 99.331

UV Signal Purity>95% Pass

^{1}H, ^{13}C NMR and HPLC data of compound **41a**.

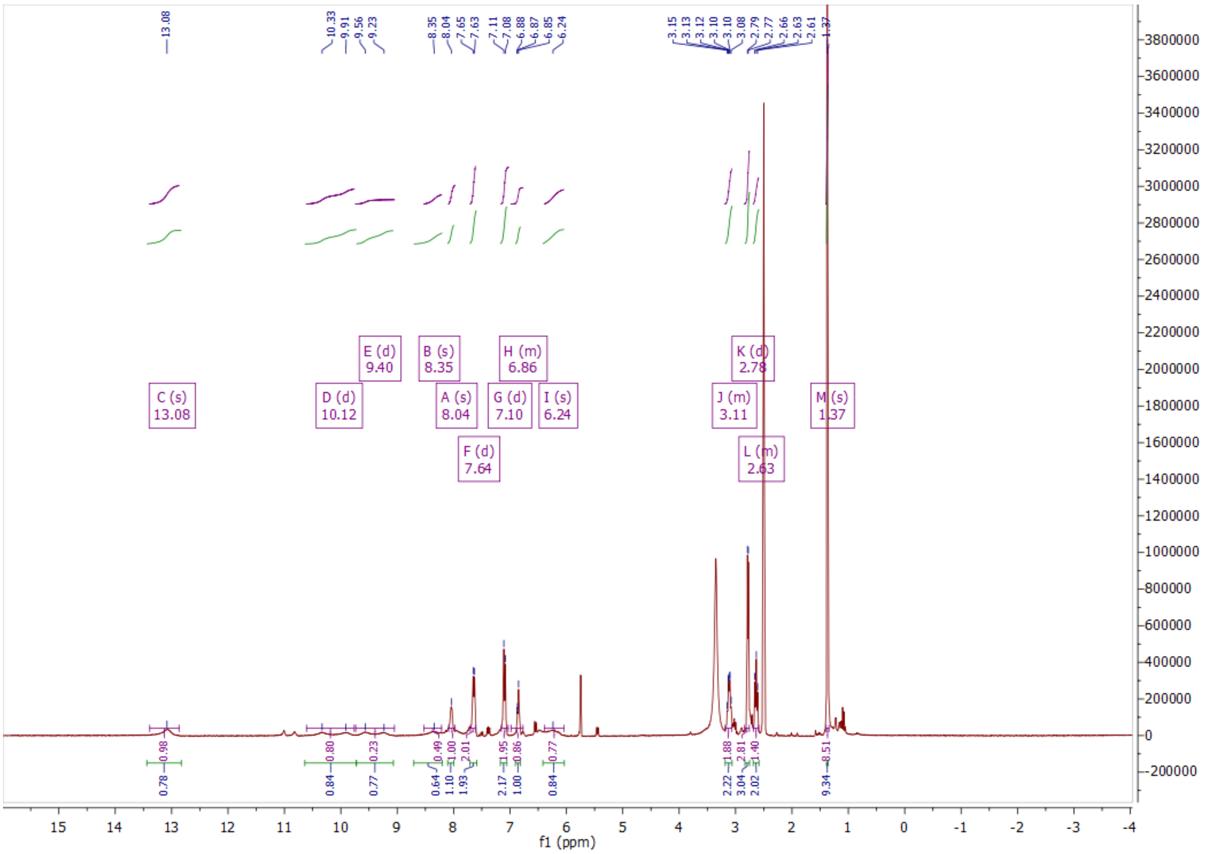

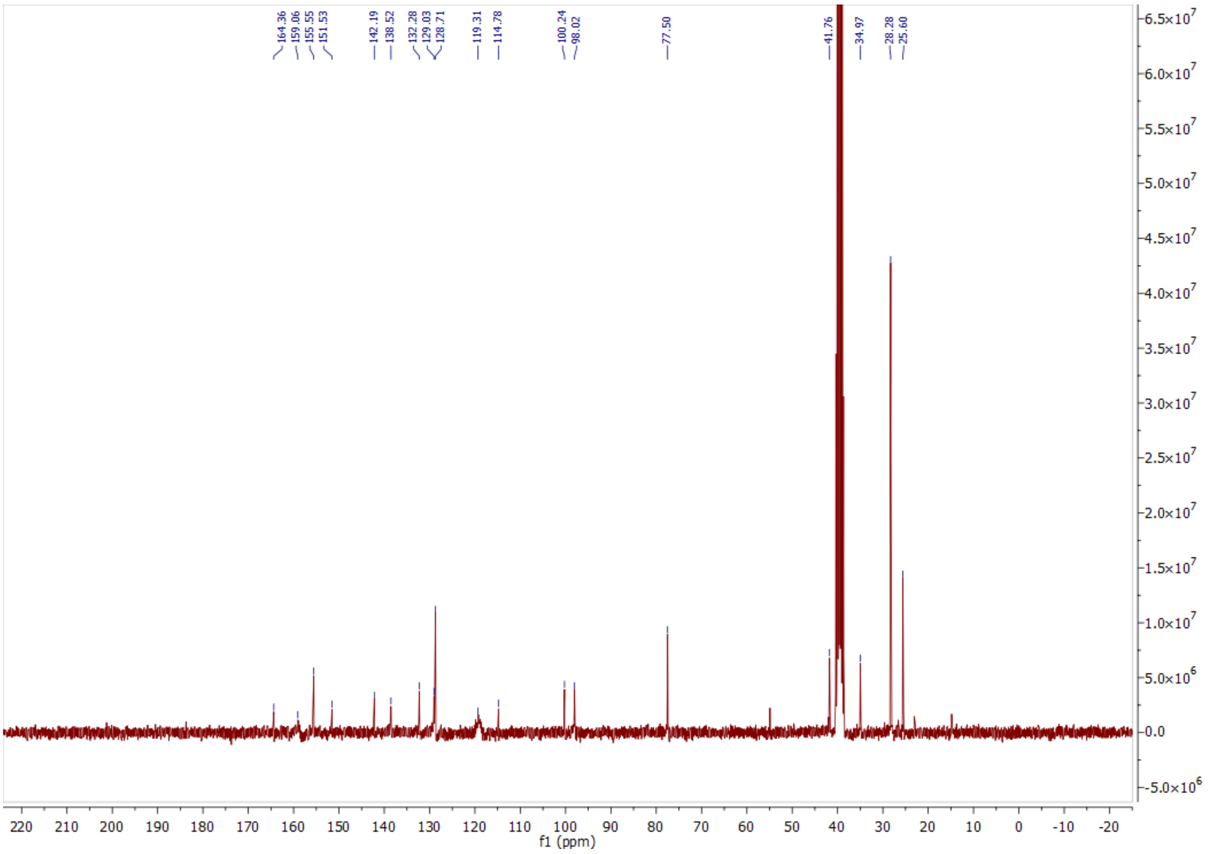

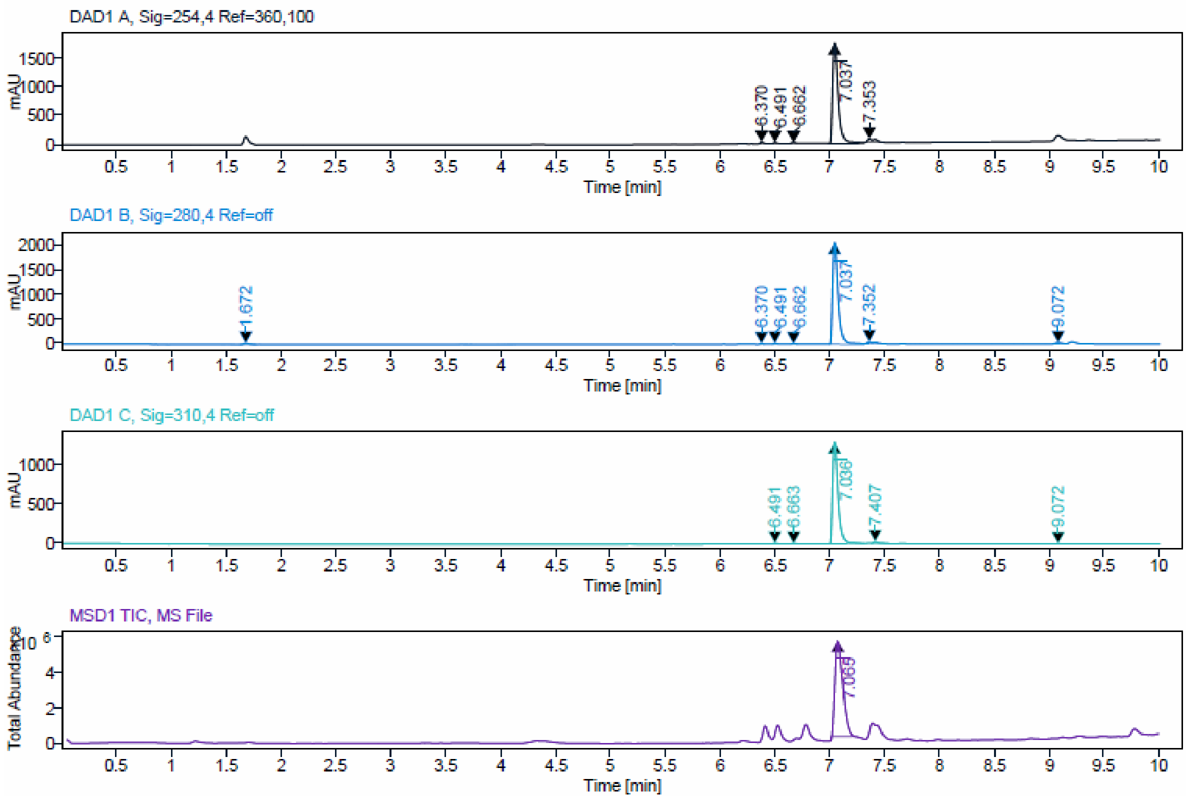

Sample Purity

Signal Description DAD1 A, Sig=254,4 Ref=360,100

Sample Name	Name	RT	Width	Area	Area%	Height
JA314_F5_gew		6.370	0.027	31.1355	0.47	21.0812
JA314_F5_gew		6.491	0.028	21.8895	0.33	14.3091
JA314_F5_gew		6.662	0.032	26.1582	0.39	14.8105
JA314_F5_gew		7.037	0.052	6338.6504	95.52	1759.8859
JA314_F5_gew		7.353	0.043	218.0476	3.29	49.4386

Max Area% 95.521

UV Signal Purity>95% Pass

Signal Description	DAD1 B, Sig=280,4 Ref=off

Sample Name	Name	RT	Width	Area	Area%	Height
JA314_F5_gew		1.672	0.050	91.4392	1.19	26.9358
JA314_F5_gew		6.370	0.030	19.9674	0.26	11.6932
JA314_F5_gew		6.491	0.032	30.5953	0.40	16.8101
JA314_F5_gew		6.662	0.031	21.9173	0.29	11.9362
JA314_F5_gew		7.037	0.052	7313.4629	95.22	2066.3850
JA314_F5_gew		7.352	0.091	145.2905	1.89	36.6547
JA314_F5_gew		9.072	0.056	57.9037	0.75	18.5854

Max Area%	95.220
UV Signal Purity>95%	Pass
Signal Description	DAD1 C, Sig=310,4 Ref=off

Sample Name	Name	RT	Width	Area	Area%	Height
JA314_F5_gew		6.491	0.037	26.4131	0.57	9.8582
JA314_F5_gew		6.663	0.036	18.6254	0.40	7.3411
JA314_F5_gew		7.036	0.052	4479.9907	97.11	1287.3610
JA314_F5_gew		7.407	0.044	73.6294	1.60	20.7778
JA314_F5_gew		9.072	0.072	14.5269	0.31	3.0419

Max Area%	97.113
UV Signal Purity>95%	Pass

^{1}H, ^{13}C NMR and HPLC data of compound **41b**.

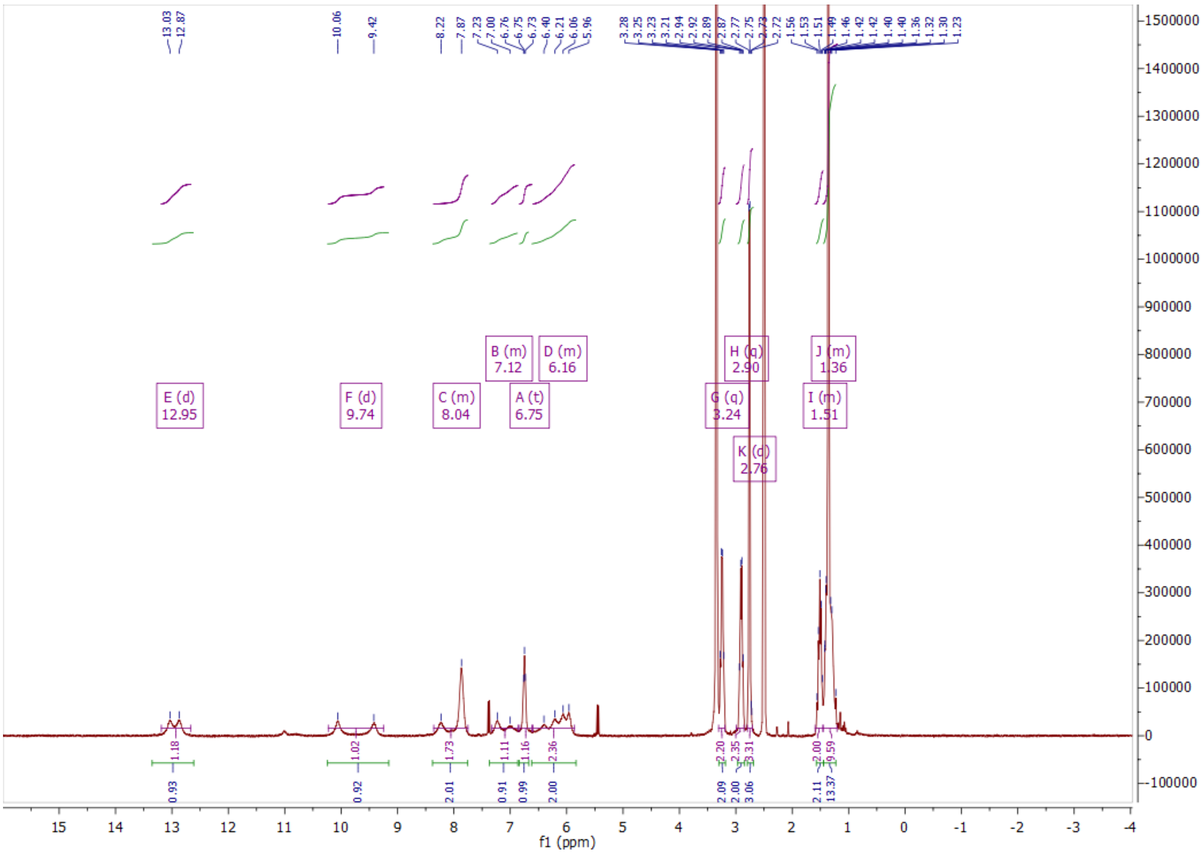

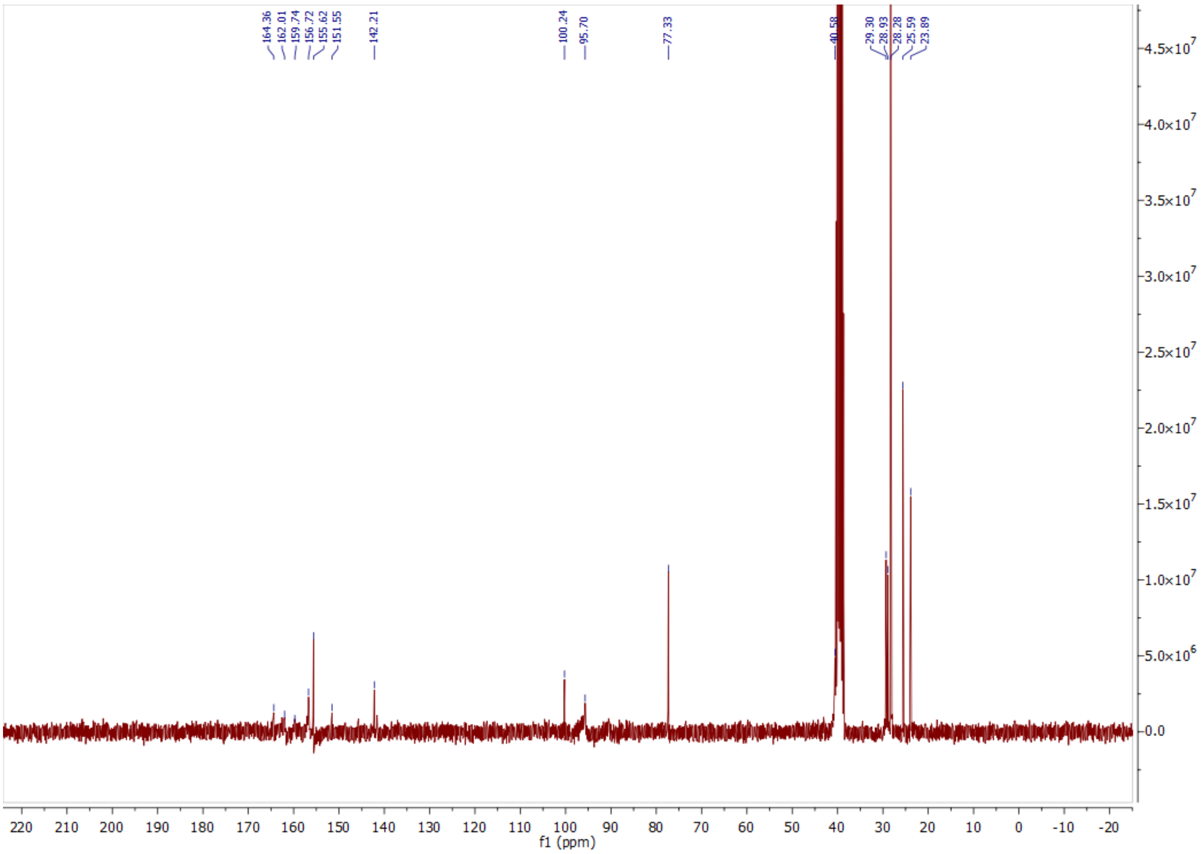

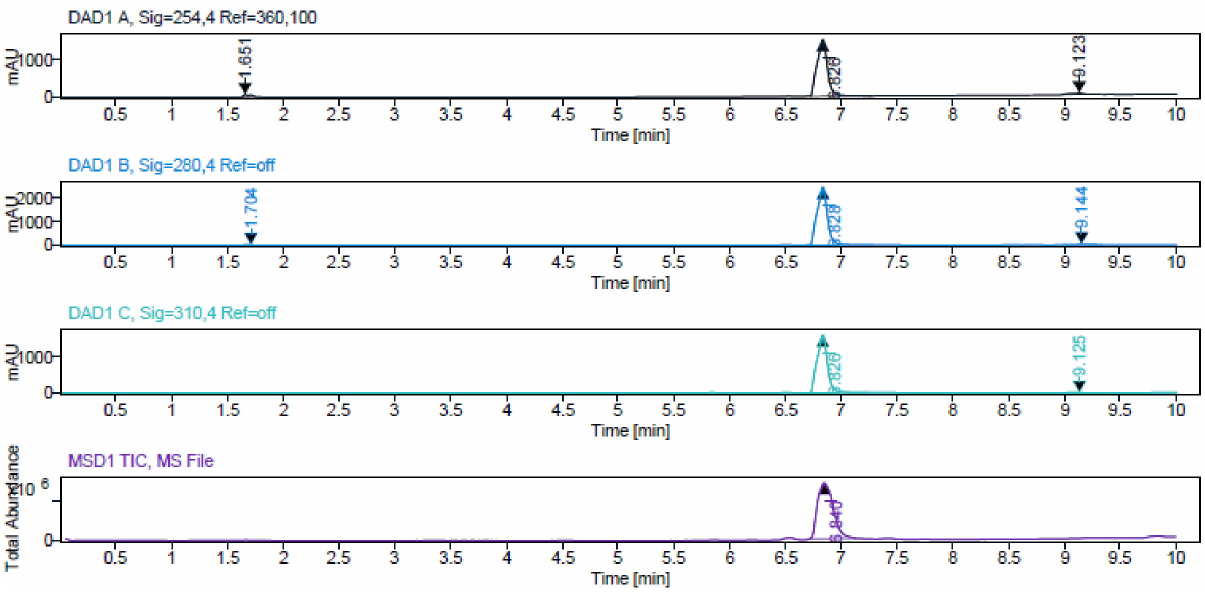

Sample Purity

Signal Description DAD1 A, Sig=254,4 Ref=360,100

Sample Name	Name	RT	Width	Area	Area%	Height
JA316_gew		1.651	0.086	237.8882	2.37	58.5248
JA316_gew		6.826	0.102	9522.5215	95.02	1490.9647
JA316_gew		9.123	0.147	260.7821	2.60	30.6708

Max Area% 95.024

UV Signal Purity>95% Pass

Signal Description DAD1 B, Sig=280,4 Ref=off

Sample Name	Name	RT	Width	Area	Area%	Height
JA316_gew		1.704	0.088	36.2562	0.23	8.5621
JA316_gew		6.828	0.101	15536.6641	98.29	2436.4849
JA316_gew		9.144	0.183	234.4920	1.48	21.0569

Max Area% 98.287

UV Signal Purity>95% Pass

Signal Description DAD1 C, Sig=310,4 Ref=off

Sample Name	Name	RT	Width	Area	Area%	Height
JA316_gew		6.826	0.102	10291.1426	99.88	1605.3280
JA316_gew		9.125	0.171	12.0582	0.12	1.2114

Max Area% 99.883

UV Signal Purity>95% Pass

^{1}H, ^{13}C NMR and HPLC data of compound **41c**.

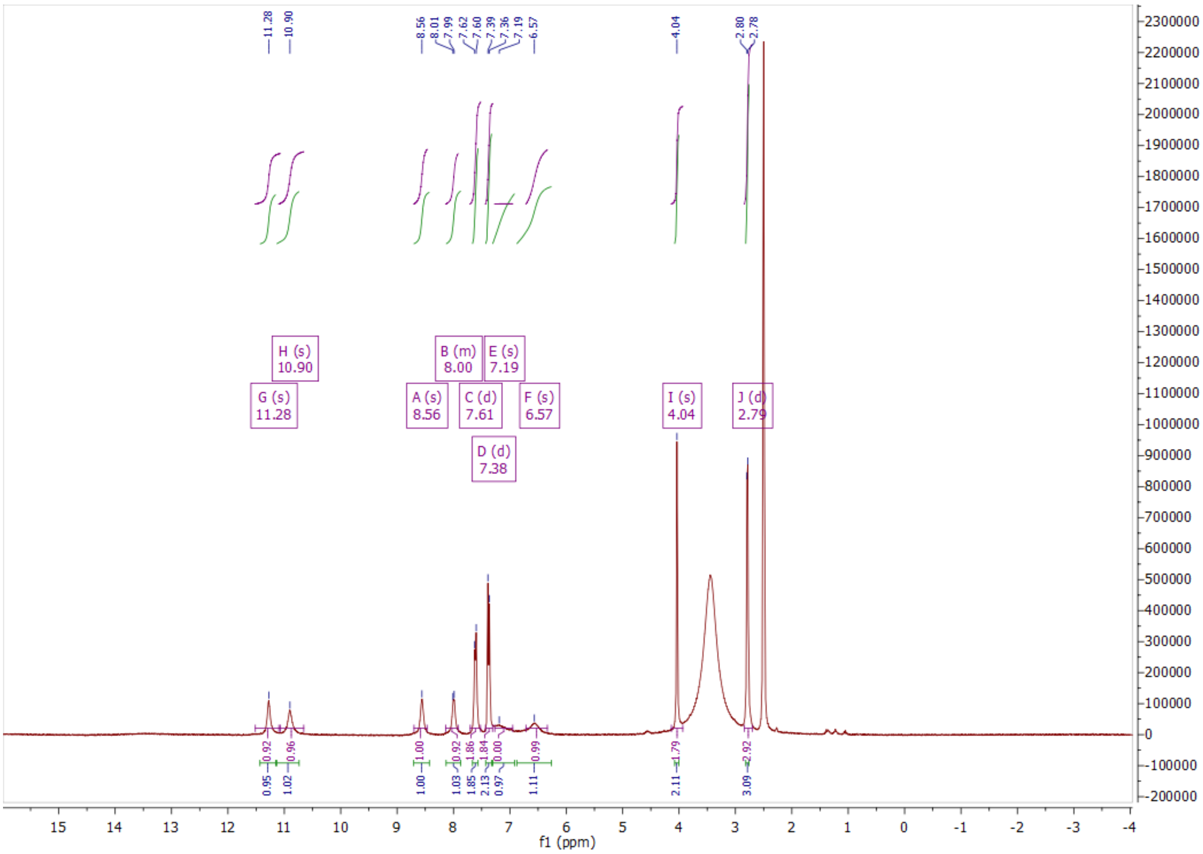

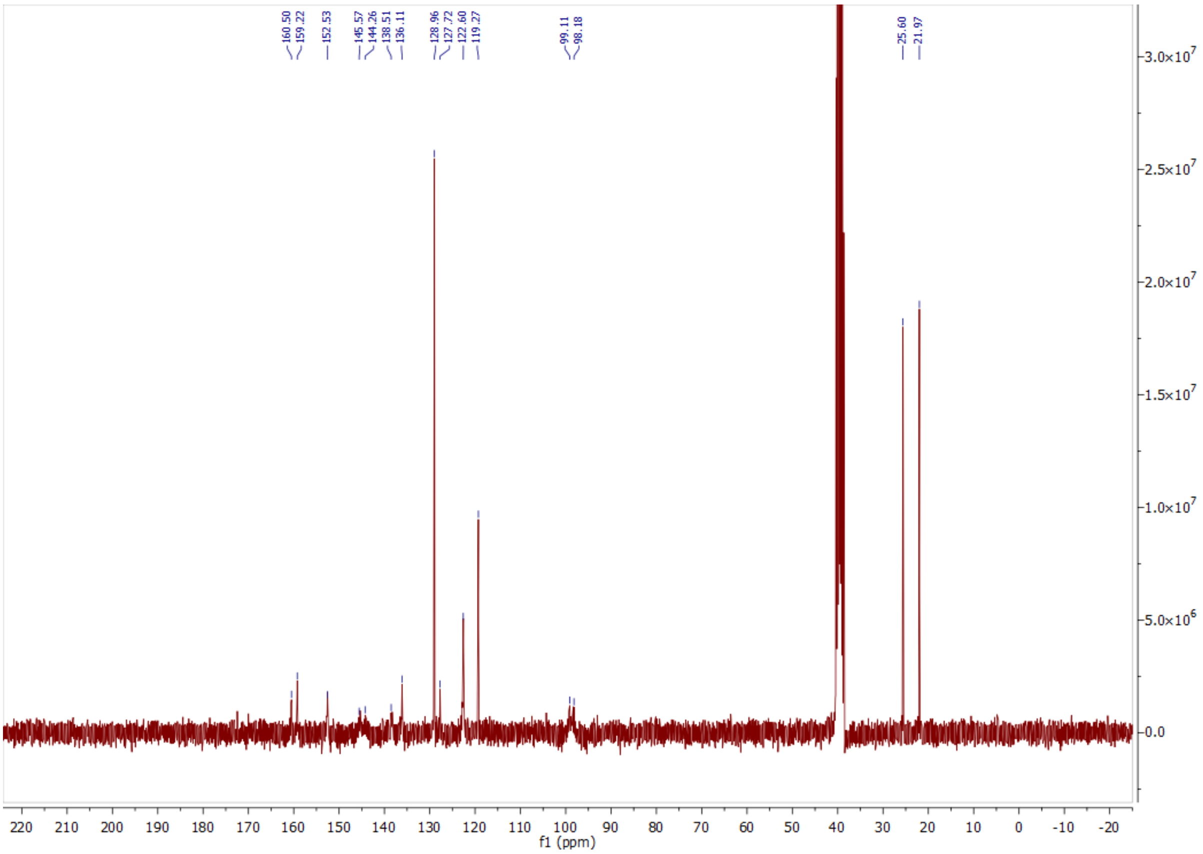

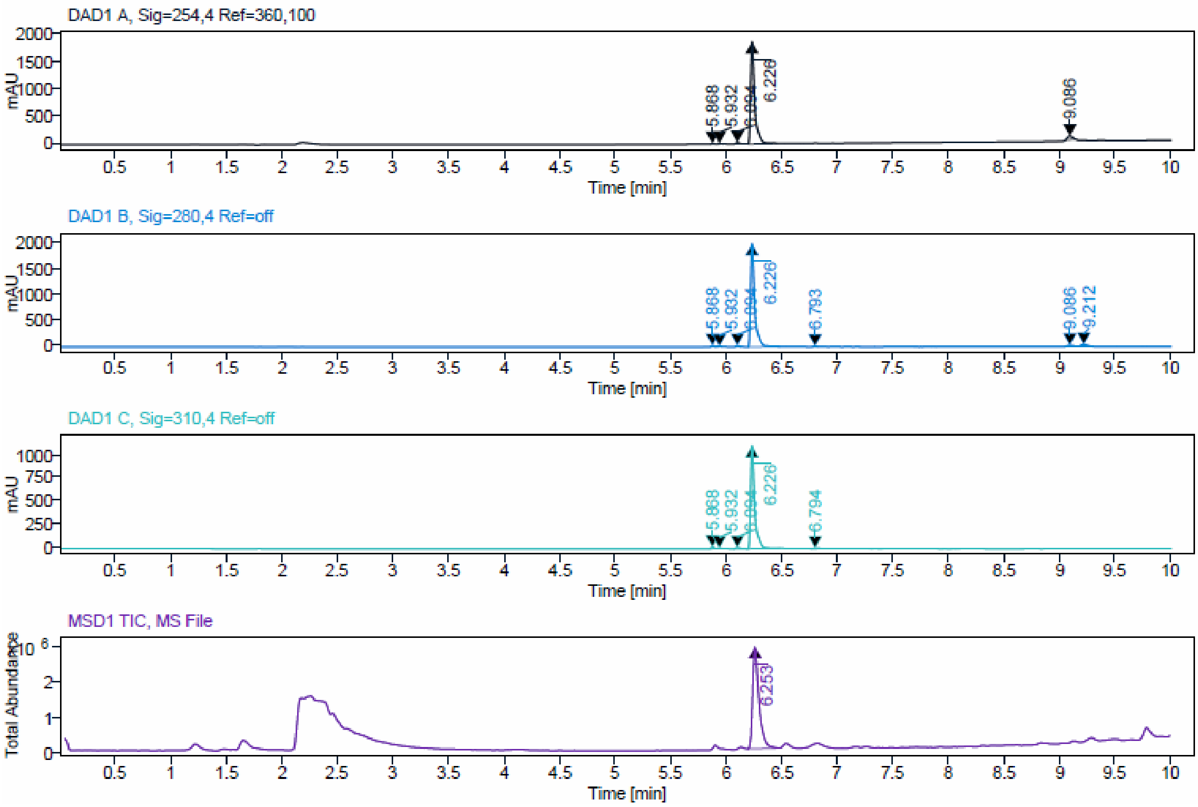

Sample Purity

Signal Description DAD1 A, Sig=254,4 Ref=360,100

Sample Name	Name	RT	Width	Area	Area%	Height
JA315_Feststoff		5.868	0.023	11.8542	0.23	9.0758
JA315_Feststoff		5.932	0.024	14.0073	0.27	10.2022
JA315_Feststoff		6.094	0.028	33.6666	0.65	21.1828
JA315_Feststoff		6.226	0.037	4911.0620	95.09	1864.5328
JA315_Feststoff		9.086	0.056	193.9945	3.76	61.8013

Max Area% 95.091

UV Signal Purity>95% Pass

Signal Description DAD1 B, Sig=280,4 Ref=off

Sample Name	Name	RT	Width	Area	Area%	Height
JA315_Feststoff		5.868	0.025	36.0247	0.65	23.2383
JA315_Feststoff		5.932	0.022	11.8471	0.21	8.9383
JA315_Feststoff		6.094	0.028	36.9698	0.67	22.0486
JA315_Feststoff		6.226	0.036	5270.0771	95.25	2005.1448
JA315_Feststoff		6.793	0.034	12.6696	0.23	7.3899
JA315_Feststoff		9.086	0.058	62.7860	1.13	18.7597
JA315_Feststoff		9.212	0.055	102.7732	1.86	31.7571

Max Area% 95.246

UV Signal Purity>95% Pass

Signal Description DAD1 C, Sig=310,4 Ref=off

Sample Name	Name	RT	Width	Area	Area%	Height
JA315_Feststoff		5.868	0.027	31.1603	1.04	18.0765
JA315_Feststoff		5.932	0.024	10.1894	0.34	6.7228
JA315_Feststoff		6.094	0.033	33.6390	1.12	14.2018
JA315_Feststoff		6.226	0.036	2894.2144	96.58	1106.0254
JA315_Feststoff		6.794	0.073	27.4460	0.92	6.5380

Max Area% 96.582

UV Signal Purity>95% Pass

^{1}H, ^{13}C NMR and HPLC data of compound **42b**.

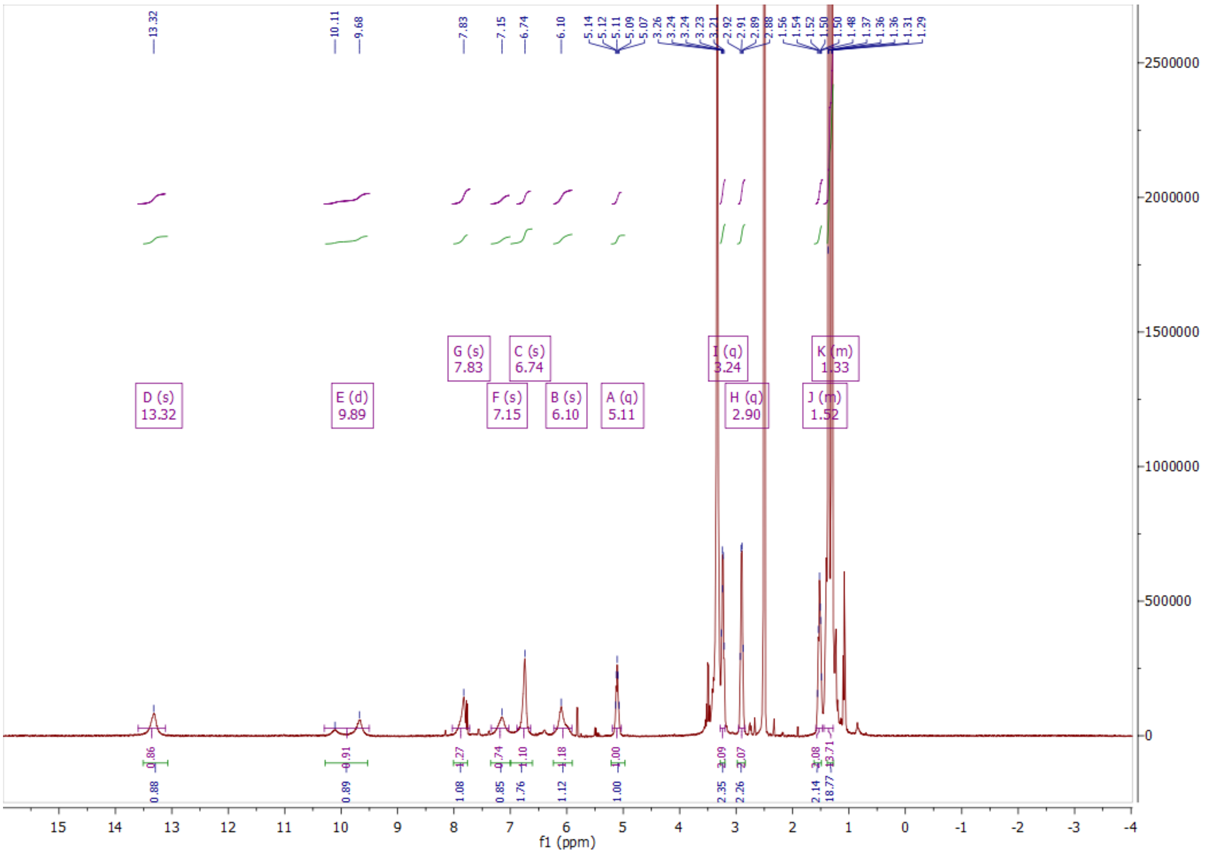

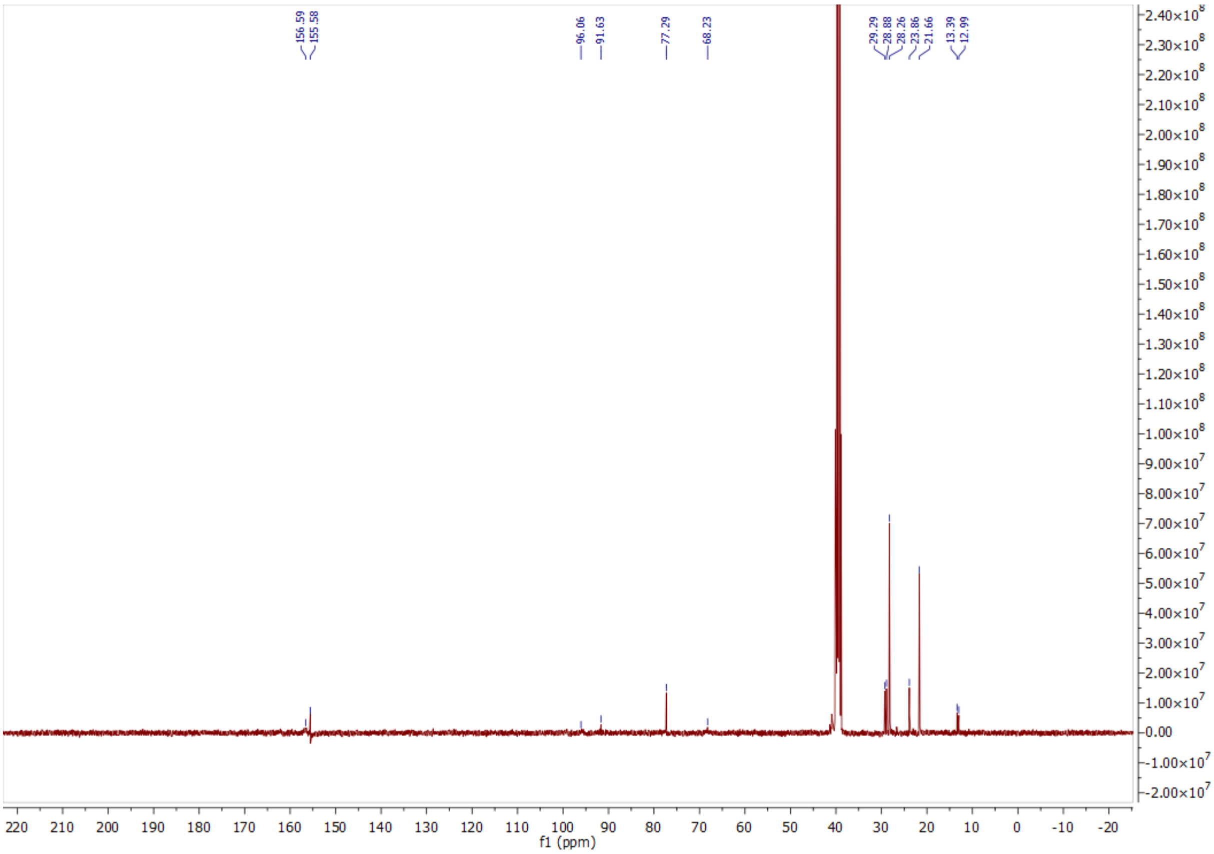

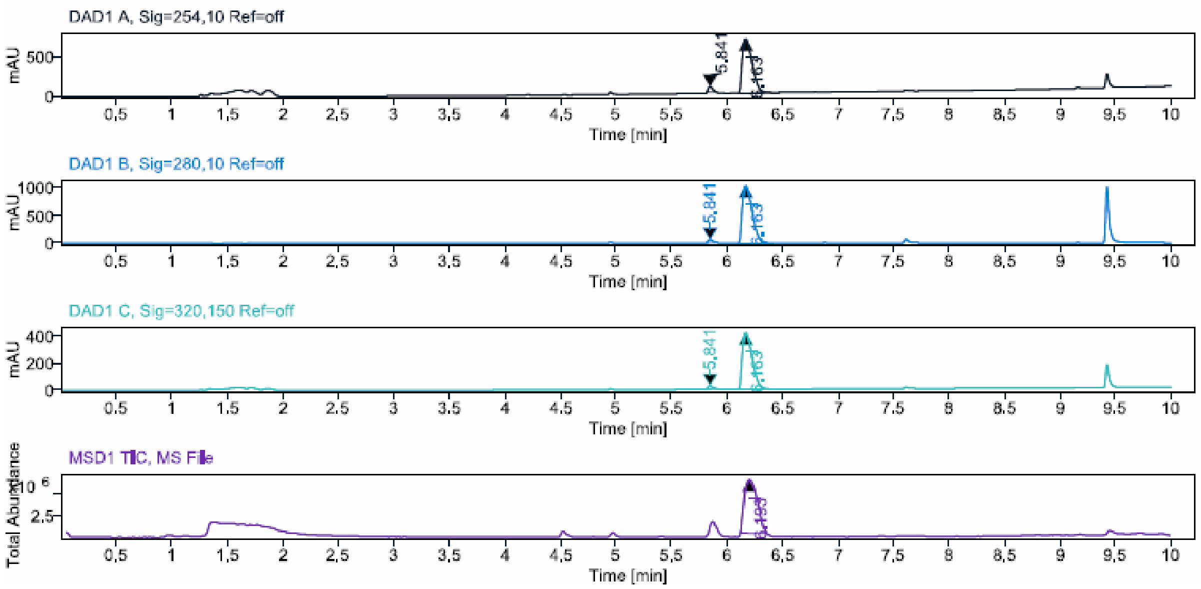

Sample Purity

Signal Description DAD1 A, Sig=254,10 Ref=off

Sample Name	Name	RT	Width	Area	Area%	Height
JA401_F20-3_gew		5,841	0,048	213,1942	4,71	77,7819
JA401_F20-3_gew		6.163	0.099	4310.3647	95.29	694.2296

Max Area% 95,287

UV Signal Purity>95% Pass

Signal Description DAD1 B, Sig=280,10 Ref=off

Sample Name	Name	RT	Width	Area	Area%	Height
JA401_F20-3_gew		5,841	0,049	161,7128	2,39	58,1263
JA401_F20-3_gew		6.163	0.098	6605.2827	97.61	1068.1763

Max Area% 97,610

UV Signal Purity>95% Pass

Signal Description DAD1 C, Sig=320,150 Ref=off

Sample Name	Name	RT	Width	Area	Area%	Height
JA401_F20-3_gew		5,841	0,048	65,2982	2,47	23,3064
JA401_F20-3_gew		6,163	0,098	2576,2925	97,53	425,1884

Max Area% 97.528

UV Signal Purity>95% Pass

^{1}H, ^{13}C NMR and HPLC data of compound **42d**.

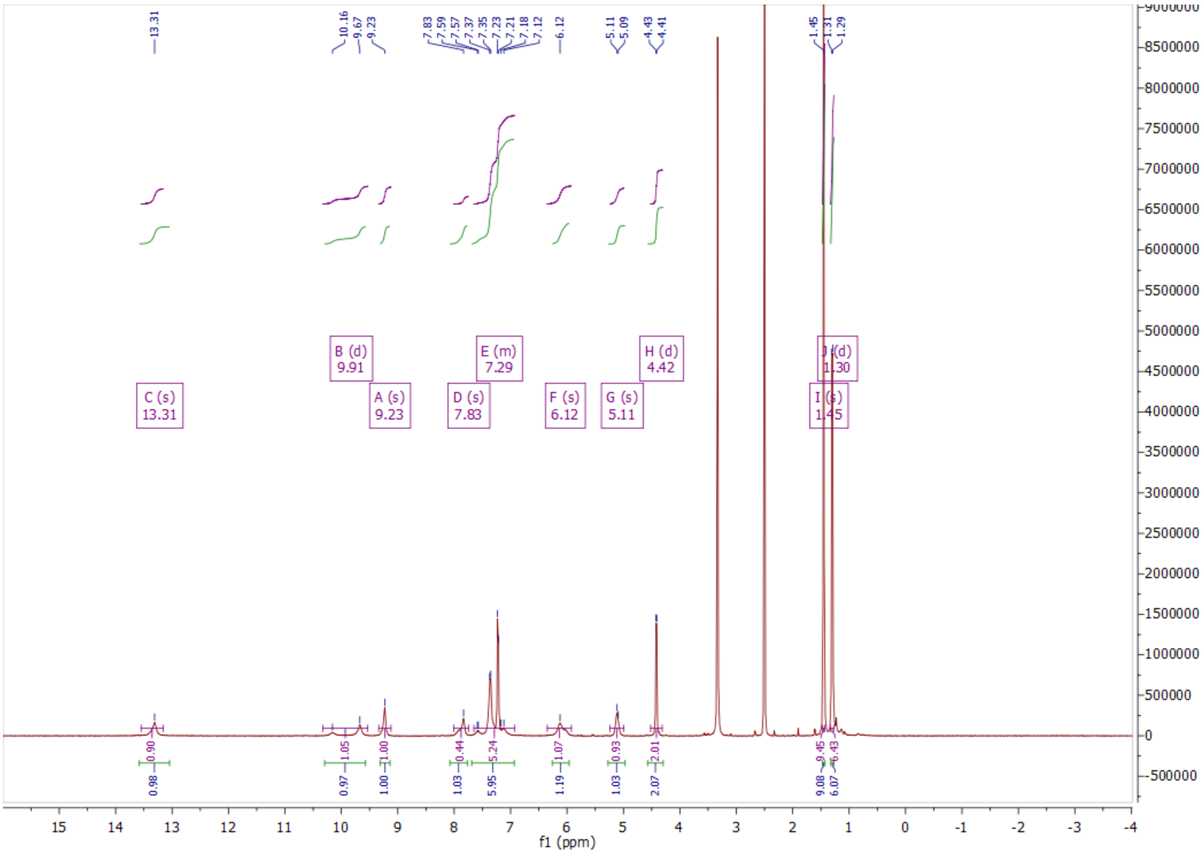

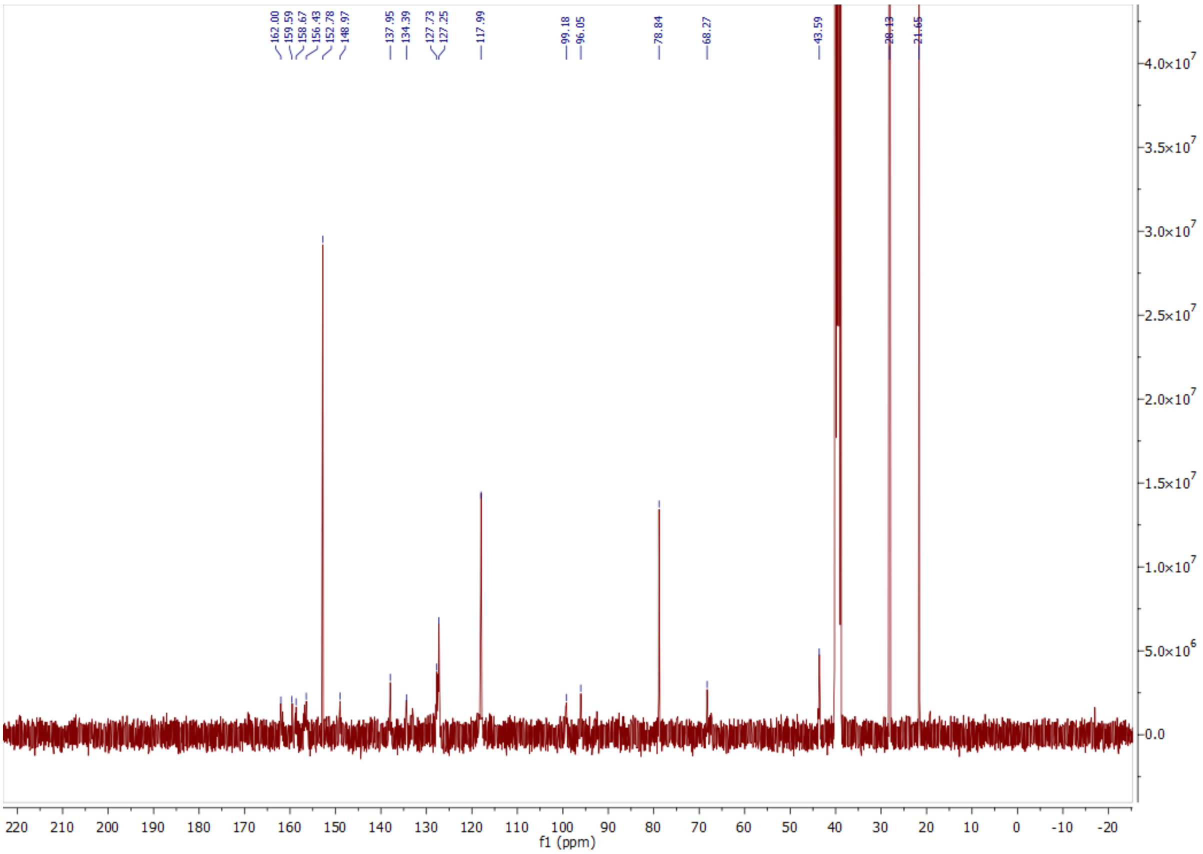

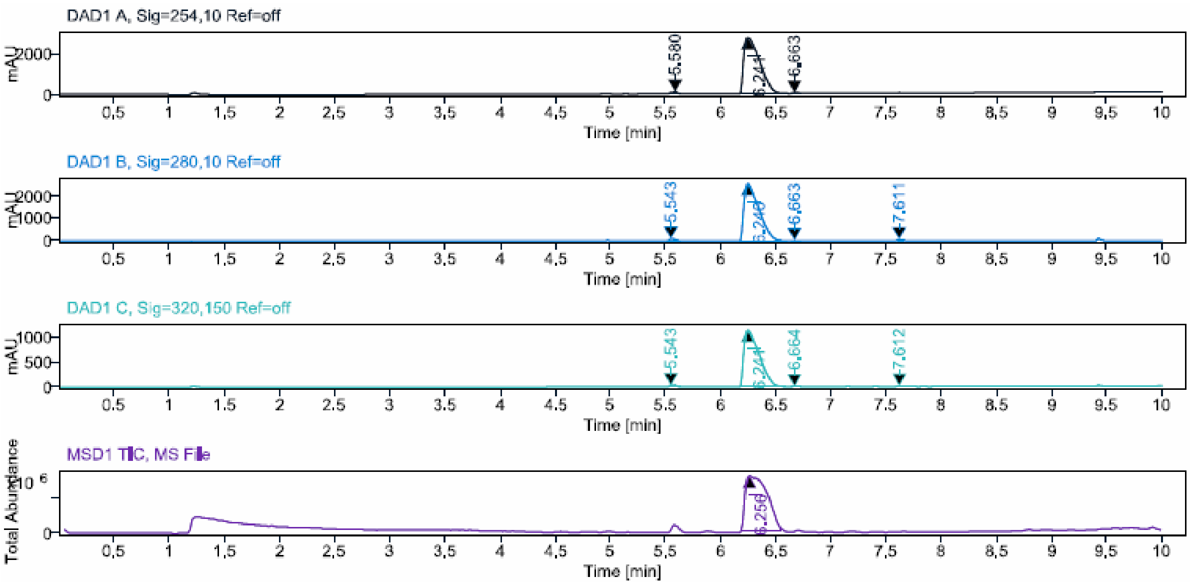

Sample Purity

Signal Description DAD1 A, Sig=254,10 Ref=off

Sample Name	Name	RT	Width	Area	Area%	Height
JA400_F23-30_gew		5,580	0,034	297,0102	1,06	104,3446
JA400_F23-30_gew		6,241	0,165	27510,92 38	98,48	2733,2893
JA400_F23-30_gew		6.663	0.065	126.9736	0.45	35.0586

Max Area% 98,482

UV Signal Purity>95% Pass

Signal Description DAD1 B, Sig=280,10 Ref=off

Sample Name	Name	RT	Width	Area	Area%	Height
JA400_F23-30_gew		5,543	0,065	355,3736	1,44	94,8632
JA400_F23-30_gew		6,240	0,151	24087,23 63	97,91	2548,1602
JA400_F23-30_gew		6,663	0,082	78,0396	0,32	18,1591
JA400_F23-30_gew		7.611	0.027	81.2656	0.33	53.6894

Max Area% 97.908

UV Signal Purity>95% Pass

Signal Description DAD1 C, Sig=320,150 Ref=off

Sample Name	Name	RT	Width	Area	Area%	Height
JA400_F23-30_gew		5,543	0,071	131,4802	1,14	33,7244
JA400_F23-30_gew		6.241	0.157	11352,55 27	98.23	1164.3856
JA400_F23-30_gew		6.664	0.075	34.0731	0.29	8.6266
JA400_F23-30_gew		7.612	0.056	39.2604	0.34	14.2551

Max Area% 98,228

UV Signal Purity>95% Pass

^{1}H, ^{13}C NMR and HPLC data of compound **43b**.

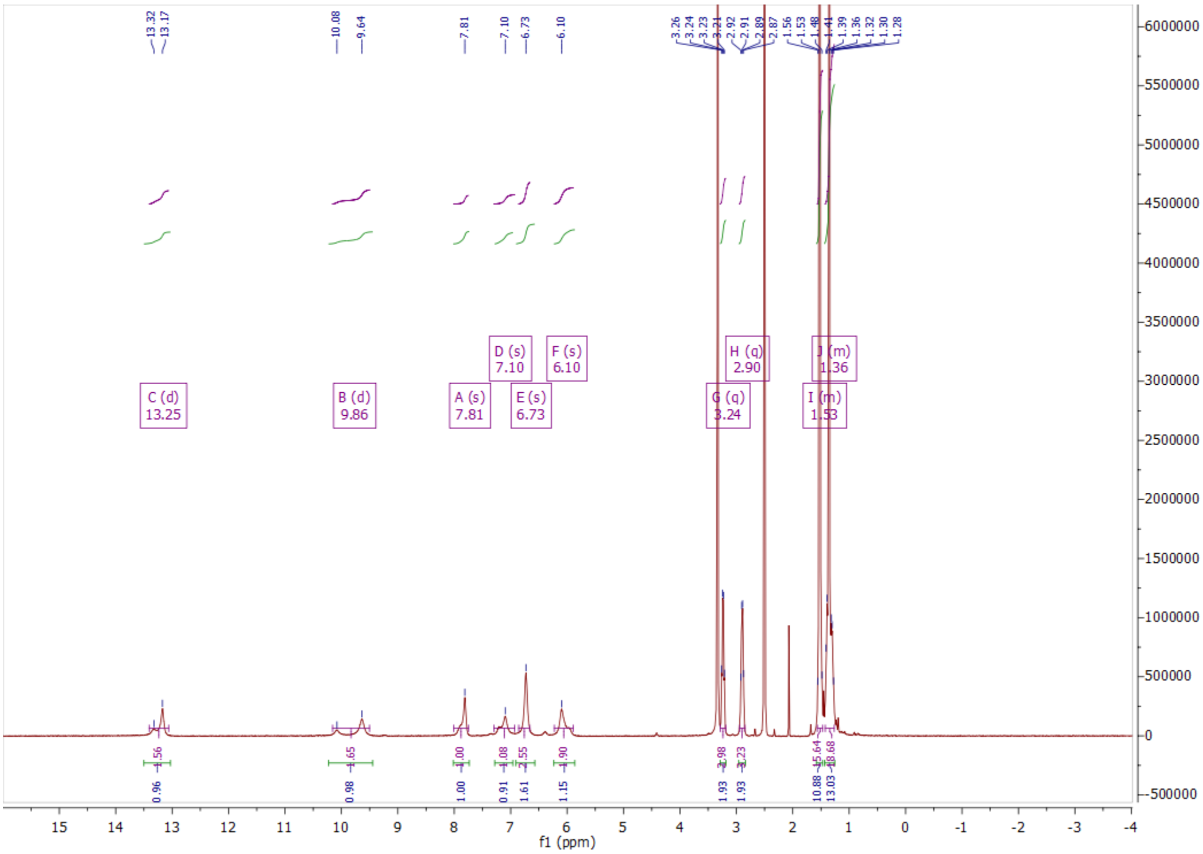

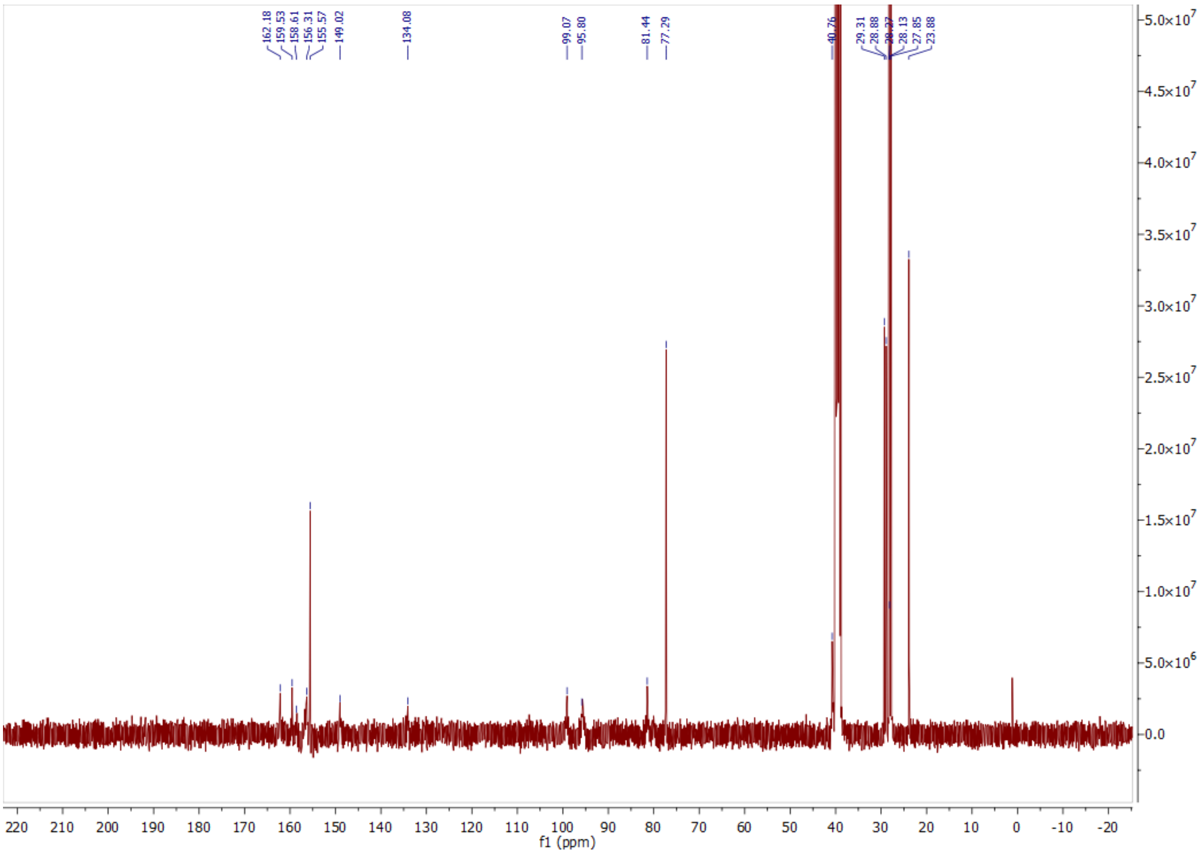

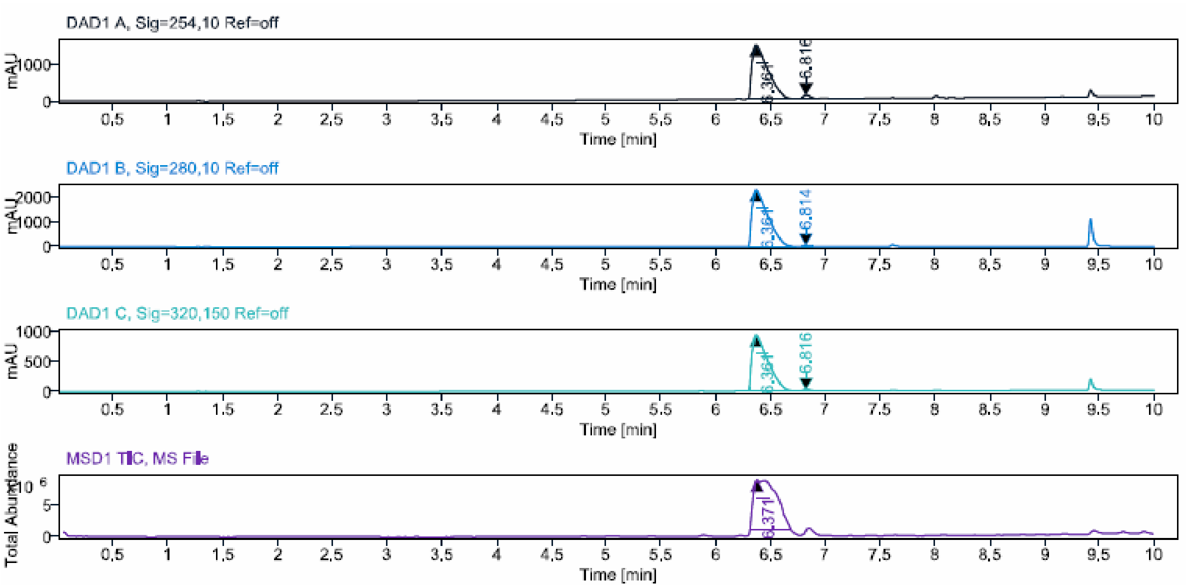

Sample Purity

Signal Description DAD1 A, Sig=254,10 Ref=off

Sample Name	Name	RT	Width	Area	Area%	Height
JA402_rp_F8		6.361	0.158	15098.88 67	97.42	1515.8259
JA402_rp_F8		6.816	0.062	400.4716	2.58	107.1271

Max Area% 97.416

UV Signal Purity>95% Pass

Signal Description DAD1 B, Sig=280,10 Ref=off

Sample Name	Name	RT	Width	Area	Area%	Height
JA402_rp_F8		6.361	0.159	22989.56 45	99.59	2303.4490
JA402_rp_F8		6.814	0.053	95.7846	0.41	28.1562

Max Area% 99.585

UV Signal Purity>95% Pass

Signal Description DAD1 C, Sig=320,150 Ref=off

Sample Name	Name	RT	Width	Area	Area%	Height
JA402_rp_F8		6.361	0.159	9302.6260	98.98	933.1728
JA402_rp_F8		6.816	0.063	95.7069	1.02	22.7253

Max Area% 98.982

UV Signal Purity>95% Pass

^{1}H, ^{13}C NMR and HPLC data of compound **43d**.

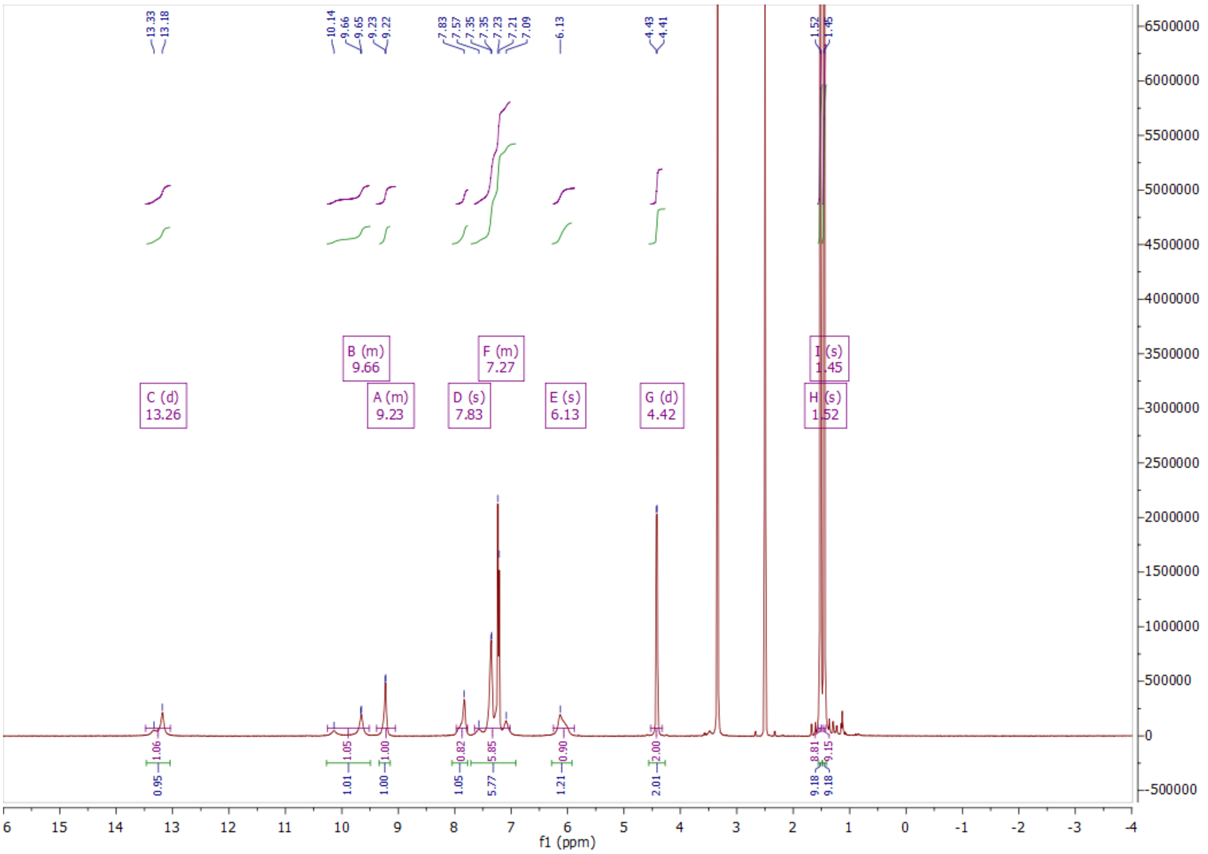

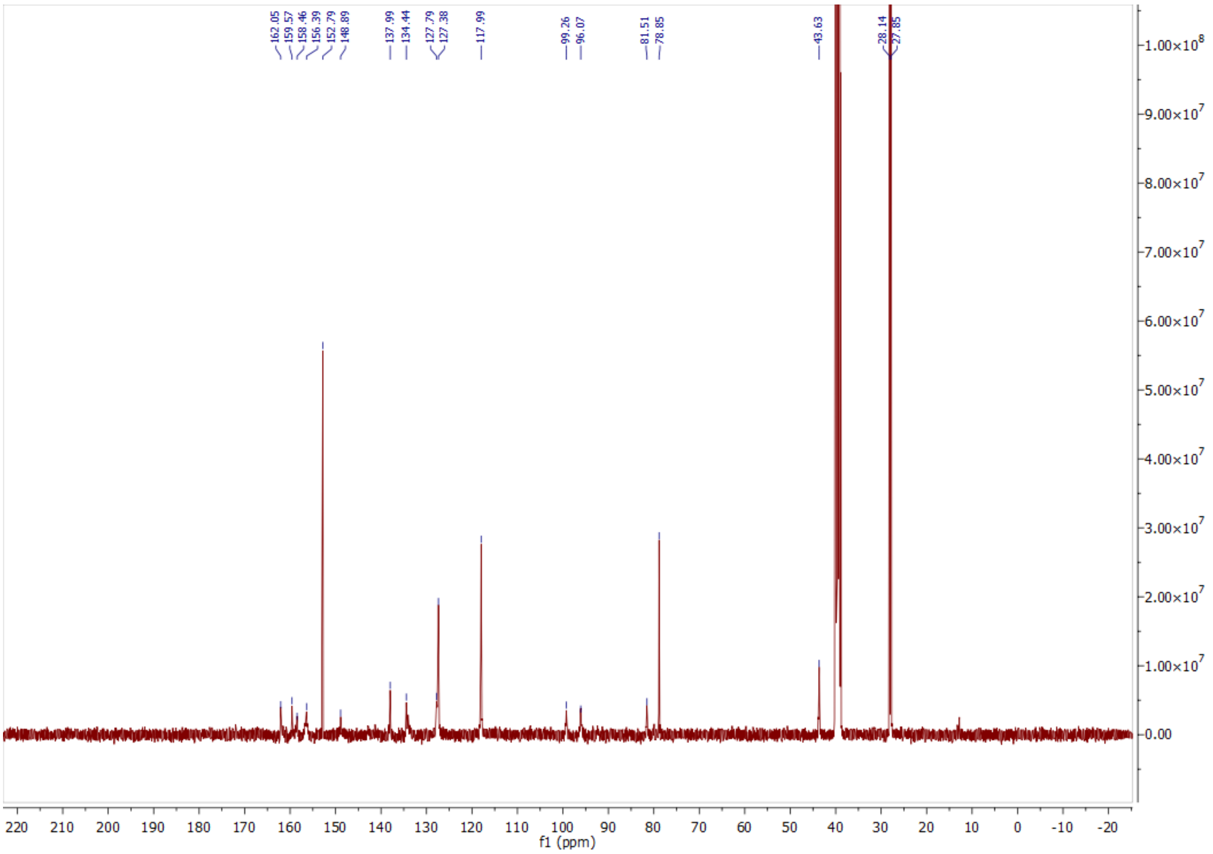

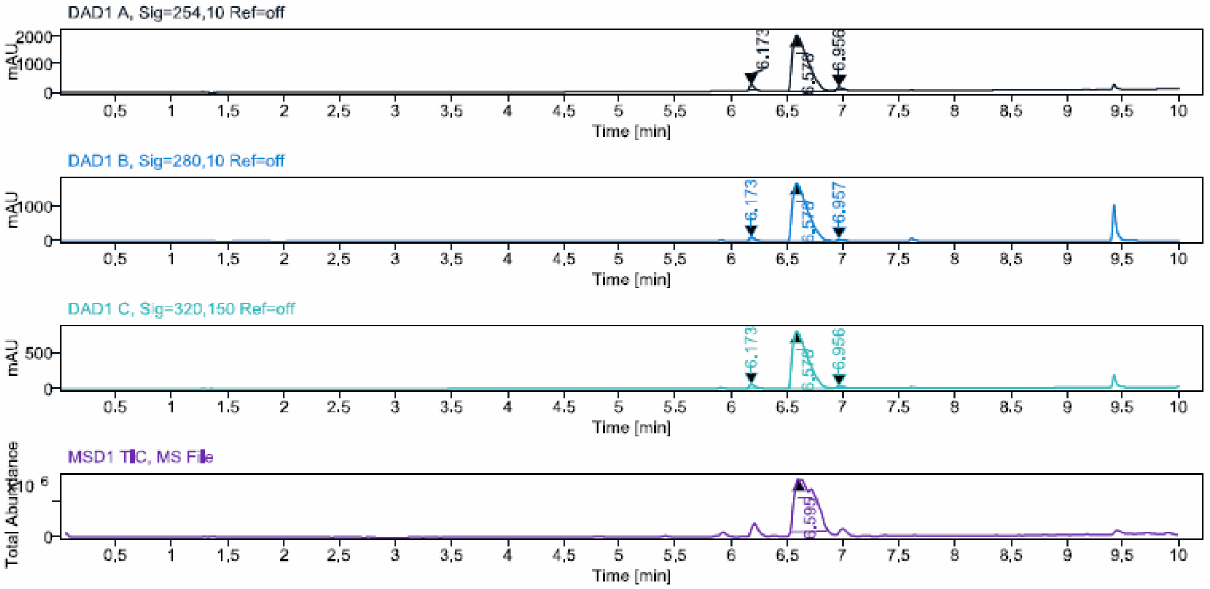

Sample Purity

Signal Description DAD1 A, Sig=254,10 Ref=off

Sample Name	Name	RT	Width	Area	Area%	Height
JA397_rp2_F8		6,173	0,052	627,0422	3,05	201,3510
JA397_rp2_F8		6,578	0,153	19615,33 40	95,39	2057,7278
JA397_rp2_F8		6.956	0.056	321.3210	1.56	99.1156

Max Area% 95,388

UV Signal Purity>95% Pass

Signal Description DAD1 B, Sig=280,10 Ref=off

Sample Name	Name	RT	Width	Area	Area%	Height
JA397_rp2_F8		6,173	0,052	279,9376	1,71	93,2231
JA397_rp2_F8		6,578	0,148	15957,24 22	97,62	1716,4701
JA397_rp2_F8		6,957	0,058	108,7941	0,67	35,6585

Max Area% 97,622

UV Signal Purity>95% Pass

Signal Description DAD1 C, Sig=320,150 Ref=off

Sample Name	Name	RT	Width	Area	Area%	Height
JA397_rp2_F8		6,173	0,055	156,1059	1,98	51,0682
JA397_rp2_F8		6,578	0,151	7630,3564	96,67	815,0915
JA397_rp2_F8		6,956	0,066	106,3827	1,35	25,6990

Max Area% 96,674

UV Signal Purity>95% Pass
